# Design and Synthesis of Novel HIV-1 NNRTIs with Bicyclic Cores and with Improved Physicochemical Properties

**DOI:** 10.1021/acs.jmedchem.2c01574

**Published:** 2023-01-18

**Authors:** Ladislav Prener, Ondřej Baszczyňski, Martin M. Kaiser, Martin Dračínský, George Stepan, Yu-Jen Lee, Boris Brumshtein, Helen Yu, Petr Jansa, Eric B. Lansdon, Zlatko Janeba

**Affiliations:** † Institute of Organic Chemistry and Biochemistry of the Czech Academy of Sciences, Flemingovo nám. 2, Prague 6 160 00, Czech Republic; ‡ Department of Organic Chemistry, Faculty of Science, Charles University, Hlavova 8, Prague 2 128 43, Czech Republic; § Gilead Sciences Inc., 333 Lakeside Drive, Foster City, California 94404, United States

## Abstract

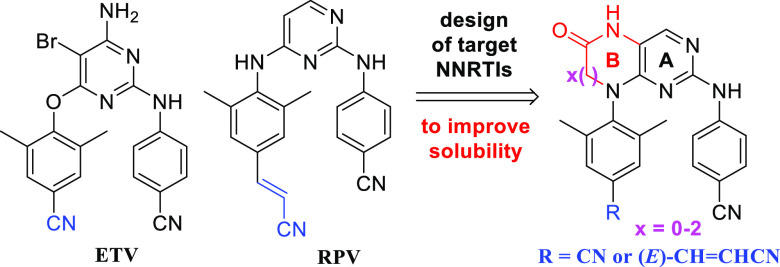

Non-nucleoside reverse transcriptase inhibitors (NNRTIs) represent cornerstones of current regimens for treatment of human immunodeficiency virus type 1 (HIV-1) infections. However, NNRTIs usually suffer from low aqueous solubility and the emergence of resistant viral strains. In the present work, novel bicyclic NNRTIs derived from etravirine (ETV) and rilpivirine (RPV), bearing modified purine, tetrahydropteridine, and pyrimidodiazepine cores, were designed and prepared. Compounds **2**, **4**, and **6** carrying the acrylonitrile moiety displayed single-digit nanomolar activities against the wild-type (WT) virus (EC_50_ = 2.5, 2.7, and 3.0 nM, respectively), where the low nanomolar activity was retained against HXB2 (EC_50_ = 2.2–2.8 nM) and the K103N and Y181C mutated strains (fold change, 1.2–6.7×). Most importantly, compound **2** exhibited significantly improved phosphate-buffered saline solubility (10.4 μM) compared to ETV and RPV (≪1 μM). Additionally, the binding modes of compounds **2**, **4**, and **6** to the reverse transcriptase were studied by X-ray crystallography.

## Introduction

Reverse transcriptase (RT) of human immunodeficiency virus (HIV) is essential for viral replication due to its responsibility for the conversion of viral RNA genome into DNA.^1^ Thus, RT represents an important and established target among anti-HIV-1 therapies to treat acquired immune deficiency syndrome.^2−5^ RT contains two distinct druggable sites: the polymerase catalytic site that is available for nucleoside reverse transcriptase inhibitors (NRTIs) and their acyclic nucleoside phosphonate analogues (ANPs), and the allosteric binding site available for non-NRTIs (NNRTIs).^6,7^ The aforementioned drug classes play a crucial role in modern highly active antiretroviral therapy, a regimen based on a combination of at least two classes of antiretroviral drugs.^8^

The main advantages of NNRTIs are their lack of requirement for metabolic activation, low nanomolar potency, and noncompetitive kinetics of binding to RT. Clinical use of first-generation NNRTIs, nevirapine and delavirdine, was limited by their low genetic barrier to resistance and toxicity.^2,9,10^ Efavirenz is a component of the first once daily single-tablet regimen to treat HIV-1; however, efavirenz-based regimens have a high rate of central nervous system side effects, which affect the overall tolerability.^11^ These issues with first-generation NNRTIs were partly circumvented in second-generation inhibitors, including the diarylpyrimidine (DAPY) structural family, namely etravirine (ETV) and rilpivirine (RPV), and lately the 2-pyridinone derivative doravirine (DOR).^12,13^

NNRTIs target an allosteric binding pocket which is situated about 10 Å from the polymerase catalytic site. Although NNRTIs include a wide range of structurally diverse scaffolds, their interactions with RT show similar binding modes. A “butterfly-like” model with a hydrophilic center (“body”) and two hydrophobic moieties (“wings” or “arms”) is a typical conformation of the first-generation NNRTIs. Alternatively, the RT-bound conformation of the DAPY family resembles a “horseshoe” or “U” shape. The inherent flexibility of ETV and RPV has been hypothesized to allow them to adapt to the binding pocket and provide the conformational adaptability to evade resistance against a wide range of mutations.^14,15^ Common NNRTI resistance mutations include Y181C and K103N. These mutations continue to show a high amount of transmitted drug resistance.^16^

**Figure 1:**
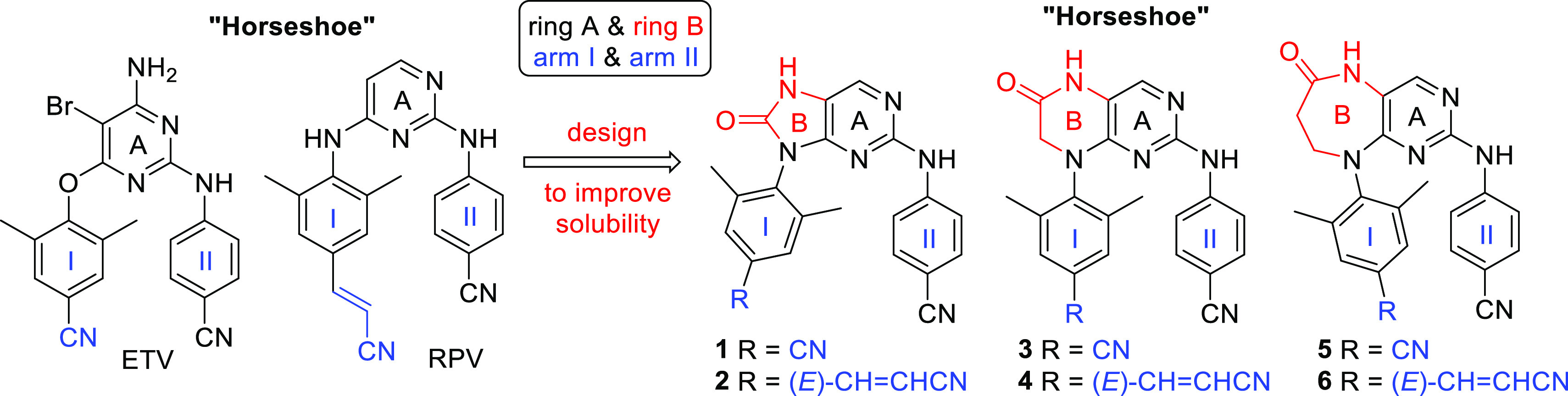
Design of novel bicyclic DAPY NNRTIs **1**–**6** derived from ETV and RPV.

The DAPY class typically suffers from poor aqueous solubility, which can contribute to low bioavailability and drug-like properties. Hence, the development of novel DAPY-like inhibitors with high potency against the WT virus and resistant viral strains, as well as improved aqueous solubility, is highly desirable.^17−24^ Numerous publications^17,25−37^ and reviews^12,38,39^ on DAPY NNRTIs have been recently published. In several cases, another (hetero)cycle was attached to the pyrimidine central core of molecules to form fused bicyclic NNRTIs in order to explore the entrance channel of the NNRTI binding pocket.^25,28,35,37^ In our current work, we decided to substitute the pyrimidine core of ETV and RPV (Figure 1) with modified bicyclic cores in order to introduce an additional polar moiety, which should result in increased aqueous solubility. Furthermore, the second ring (ring B) of the bicyclic cores may also prove advantageous from the conformational viewpoint: it is expected to lock the inhibitors in the desirable U-shape to introduce potency (especially for the six-membered analogues),^40^ while not being aromatic, it still keeps certain flexibility to accommodate changes in the binding pocket (to preserve the resistance profile). Since the size of ring B can, to some extent, influence the mutual position of the two lateral hydrophobic arms, analogues containing five-membered (compounds **1** and **2**), six-membered (**3** and **4**), and seven-membered (**5** and **6**) rings were designed (Figure 1).

## Results and Discussion

### Chemistry

We started with the synthesis of bicyclic NNRTIs containing the six-membered ring, that is compounds **3** and **4** (Figure 1), since such compounds were expected to have the appropriate “horseshoe” conformation and, thus, to be the most potent inhibitors of the whole series.

**Scheme 1:**
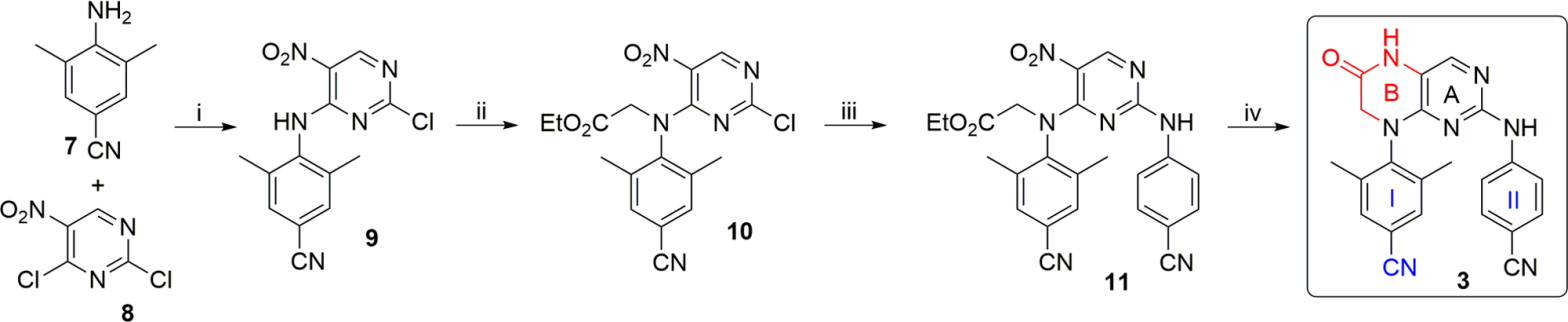
Synthetic Route for ETV Analogue **3** Reagents and conditions: (i) DIPEA, dioxane, 50 °C, 18 h, 55%; (ii) NaH, 0 °C, 15 min, then ethyl bromoacetate, DMF, 50 °C, 16 h, 21%; (iii) 4-aminobenzonitrile, *i*-PrOH, 150 °C (MW), 30 min, 54%; (iv) Fe/HCl, MeOH/H_2_O (4/1), 50 °C, 16 h, 27%.

First, compound **3** bearing the cyano group on arm I, similarly to ETV, was prepared in four steps from commercially available 4-amino-3,5-dimethylbenzonitrile (**7**, Scheme 1). Treatment of **7** with pyrimidine derivative **8** and DIPEA in dioxane afforded diarylamine **9** (55% yield), which was then alkylated with ethyl bromoacetate in DMF to give glycinate **10** in a 21% yield. Subsequent introduction of arm II using 4-aminobenzonitrile under microwave conditions gave intermediate **11** (54%), which was reductively cyclized with Fe/HCl to afford the target compound **3** in a 27% yield.

**Scheme 2:**
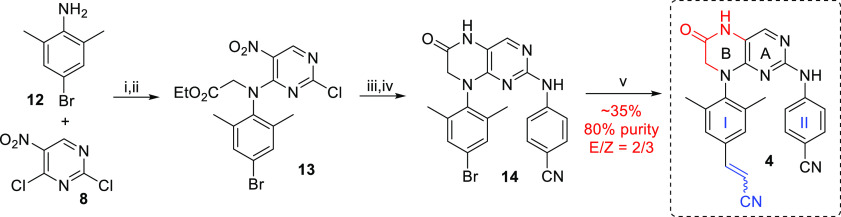
Unsuccessful Synthetic Route for RPV Analogue **4** Reagents and conditions: (i) DIPEA, dioxane, 60 °C, 3 h; (ii) NaH, 0 °C, 15 min, then ethyl bromoacetate, DMF, 50 °C, 18 h, 57% (two steps); (iii) 4-aminobenzonitrile, *i*-PrOH, 150 °C, 30 min; (iv) Fe, HCl, MeOH/H_2_O (4/1), 50 °C, 16 h, 8% (two steps) or Ra–Ni, H_2_, MeOH, 50 °C, 18 h, 14% (two steps); (v) acrylonitrile, Pd[P(*t*-Bu_3_)]_2_ (1 equiv), TEA, DMF, 100 °C, 8 h, ∼35% (80% purity), *E*/*Z* = 2/3.

We decided to prepare the six-membered ring RPV analogue, that is compound **4** (Figure 1), in a similar manner to compound **3**. Thus, treatment of 4-bromo-2,6-dimethylaniline (**12**) with pyrimidine **8** and DIPEA in dioxane, followed by alkylation with ethyl bromoacetate in DMF, afforded compound **13** in a 57% yield over two steps (Scheme 2). Subsequent introduction of arm II using 4-aminobenzonitrile and reductive cyclization with either Fe/HCl or H_2_/Ra–Ni gave the bicyclic intermediate **14** in 8 and 14% yields (two steps), respectively. Our initial approach to the bicyclic RPV analogue, that is compound **4**, relied on an introduction of the sensitive cyanovinyl moiety *via* Mizoroki–Heck coupling between acrylonitrile and aryl bromide **14**. However, the cyanovinylation of **14** by Mizoroki–Heck coupling proved to be inefficient. For example, using the reaction conditions previously used for the preparation of RPV^41^ afforded a complex reaction mixture with less than 10% of the desired product **4** [determined by ultra-performance liquid chromatography–mass spectrometry (UPLC–MS)]. Ensuing screening of some 20 alternative reaction conditions for Mizoroki–Heck reaction (various catalysts and catalyst loadings, various reaction temperatures) led to a slight improvement only: when bulky Pd[P(*t*-Bu_3_)]_2_ was used stoichiometrically in DMF with TEA as a base, desired product **4** was obtained in a 35% yield with 80% purity. Furthermore, isolated product **4** was an inseparable mixture of *E*- and *Z*-isomers (2/3) in favor of the undesired *Z*-isomer. An attempted isomerization of the cyanovinyl double bond (using I_2_, hv) was not successful. It became apparent that a new synthetic route for target compound **4**, as well as other bicyclic RPV analogues, was required.

Preliminary biological data obtained for target compound **3** and for bromo intermediate **14** revealed low nanomolar activity against the WT virus, high potency against Y181C and K103N mutated strains, and also significantly improved aqueous solubility.^42^ Encouraged by these promising data, we decided to finish the easier-to-make ETV series first.

**Scheme 3:**
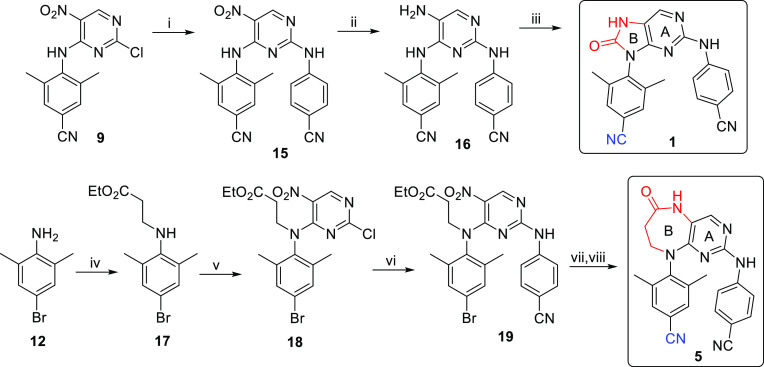
Synthetic Route for ETV Analogues **1** and **5** Reagents and conditions: (i) 4-aminobenzonitrile, *i*-PrOH, 150 °C (MW), 1 h, 62%; (ii) SnCl_2_·2H_2_O, EtOH, 60 °C, 18 h, 82%; (iii) CDI, DCM, 25 °C, 2 h, 71%; (iv) ethyl 3-bromopropionate, KI, K_2_CO_3_, MeCN, 65 °C, 5 d, 46%; (v) 2,4-dichloro-5-nitropyrimidine (**8**), DIPEA, dioxane, 70 °C, 8 h, 17%; (vi) 4-aminobenzonitrile, *i*-PrOH, 150 °C (MW), 1.5 h, 55%; (vii) Zn(CN)_2_, Pd(*t*-Bu_3_P)_2_ (30 mol %), DMF, 110 °C, 2 h; (viii) SnCl_2_·2H_2_O, Sc(OTf)_3_ (20 mol %), EtOH, 50 °C, 24 h, 54% (two steps).

The synthesis of five-membered ETV analogue **1** (Figure 1) started with the treatment of intermediate **9** with 4-aminobenzonitrile in *i*-PrOH to give compound **15** in a 62% yield (Scheme 3). The nitro group of **15** was then reduced by tin(II) chloride (to give amine **16** in an 82% yield), followed by the closure of the five-membered ring by treatment with carbonyldiimidazole (CDI)^43^ in DCM to give the desired compound **1** (Scheme 3).

In order to synthesize the seven-membered ETV analogue **5** (Figure 1), starting aniline **12** was alkylated with ethyl 3-bromopropionate (to give **17** in a 46% yield), followed by nucleophilic aromatic substitution with 2,4-dichloro-5-nitropyrimidine to afford compound **18** in a 17% yield (Scheme 3). The reaction of **18** with 4-aminobenzonitrile in *i*-PrOH gave the intermediate **19** in a 55% yield. Aryl bromide **19** was then converted into the corresponding nitrile by palladium-catalyzed cyanation, and subsequent reductive cyclization with tin(II) chloride and Sc(OTf)_3_ gave the desired pyrimidodiazepin-6-one analogue of ETV, compound **5** (Scheme 3), in a 54% yield over two steps.

**Figure 2:**
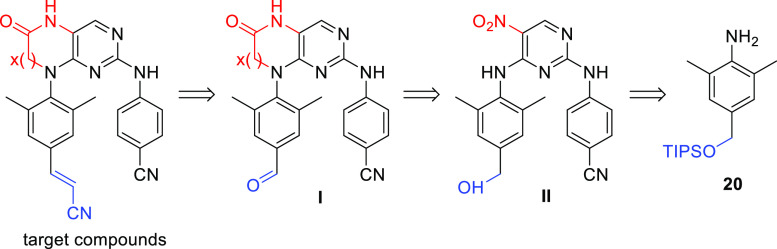
Second-generation retrosynthetic approach for bicyclic RPV-like NNRTIs leading to key intermediate **20**.

Our next goal was to develop a successful synthetic route toward the bicyclic RPV analogues **2**, **4**, and **6** (Figure 1). The installation of the cyanovinyl moiety using Mizoroki–Heck cyanovinylation on compound **14** (Scheme 2) in the last step of the first-generation synthesis failed. Thus, in the second-generation synthesis, the Horner-Wadsworth-Emmons reaction using (cyanomethyl)phosphonate and corresponding aldehyde intermediates **I** (Figure 2) was chosen for the introduction of the sensitive cyanovinyl moiety in the late stage of the novel synthetic approach. The desired aldehydes **I** and benzyl alcohols **II** can be prepared from compound **20** (Figure 2), which was identified as a suitable precursor for the preparation of the target bicyclic RPV analogues.

**Scheme 4:**
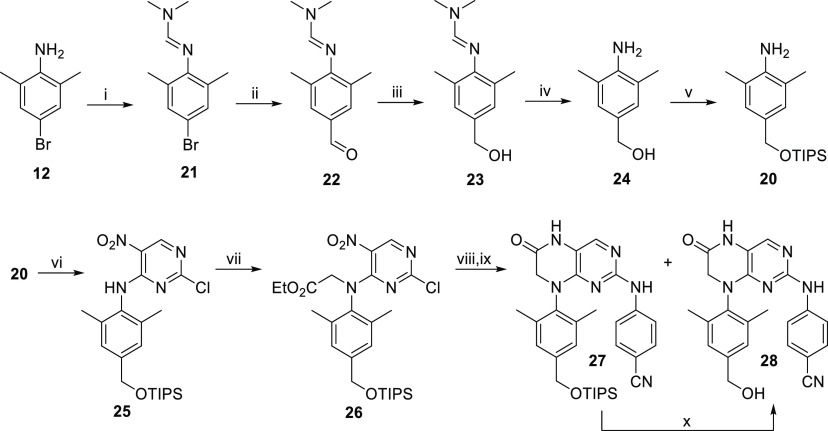
Synthetic Route for Intermediate **20** and for Hydroxymethyl Derivative **28** Reagents and conditions: (i) Vilsmeier-Haack-Arnold reagent, DCM, 25 °C, 1 h, 98%; (ii) *n*-BuLi, −78 °C, 45 min, then DMF, THF, −78 to 0 °C, 1 h; (iii) NaBH_4_, MeOH, 25 °C, 1.5 h, 81% (two steps); (iv) LiOH, 2-aminoethanol, H_2_O/*i*-PrOH (4:1), reflux, 20 h, 75%; (v) TIPSCl, imidazole, DMF, 25 °C, 6 h, 89%; (vi) 2,4-dichloro-5-nitropyrimidine (**8**), DIPEA, dioxane, 60 °C, 1.5 h, 88%; (vii) NaH, 0 °C, 20 min, then ethyl bromoacetate, DMF, 55 °C, 22 h, 66%; (viii) 4-aminobenzonitrile, *i*-PrOH, 150 °C (MW), 1.5 h; (ix) SnCl_2_·2H_2_O, Sc(OTf)_3_ (20 mol %), EtOH, 50 °C, 18 h, 30% of **27** and 13% of **28** (two steps); (x) TBAF, THF, 0 to 25 °C, 4 h, 82%.

Synthesis of precursor **20** (Scheme 4) started with the treatment of 4-bromo-2,6-dimethylaniline (**12**) with the Vilsmeier-Haack-Arnold reagent to give dimethylformimidamide **21**(44,45) in a quantitative yield. Compound **21** was then formylated using *n*-butyllithium/DMF (to yield the aldehyde **22**(44)) and subsequent reduction of the aldehyde group with NaBH_4_ in MeOH gave the corresponding alcohol **23** in an 81% yield over two steps. The removal of the protecting group with LiOH/2-aminoethanol afforded amino alcohol **24** (75%),^45^ which was then readily silylated under standard conditions to provide the desired aniline **20**.

Aniline **20** (Scheme 4) was then treated with 2,4-dichloro-5-nitropyrimidine (**8**) and DIPEA in dioxane to give secondary amine **25** (88%),^43,46^ which was then alkylated by ethyl bromoacetate to yield glycinate **26** in a 66% yield. Nucleophilic aromatic substitution with 4-aminobenzonitrile was performed on compound **26** under MW-assisted heating to introduce arm II, followed by reductive cyclization with tin(II) chloride as a reducing agent and with a catalytic amount of scandium(III) trifluoromethanesulfonate to afford tetrahydropteridine derivatives **27** and **28** in 30 and 13% yields (over two steps), respectively. The triisopropylsilyl (TIPS) protecting group was then removed with TBAF in order to convert derivative **27** to **28** in an 82% yield.

**Scheme 5:**
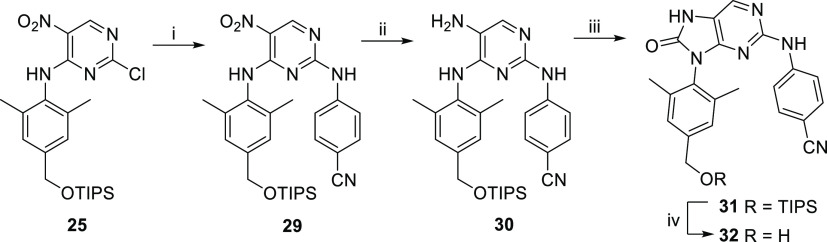
Synthetic Route for Hydroxymethyl Derivative **32** Reagents and conditions: (i) 4-aminobenzonitrile, *i*-PrOH, 150 °C (MW), 1 h, 57%; (ii) SnCl_2_·2H_2_O, EtOH, 50 °C, 30 h, 89%; (iii) CDI, DCM, 25 °C, 2 h, 91%; (iv) TBAF, THF, 0 to 25 °C, 4 h, 87%.

Intermediate **25** was heated with 4-aminobenzonitrile under MW-assisted conditions to give compound **29** in a 57% yield (Scheme 5). The nitro group of **29** was reduced with tin(II) chloride to yield amine **30** (89%), which then underwent a cyclization with CDI^43^ to give five-membered intermediate **31** in a 91% yield. Finally, the TIPS protecting group was removed from derivative **31** using TBAF to give benzyl alcohol **32** in an 87% yield.

All attempts to alkylate intermediates **20** and **25** with ethyl 3-bromopropionate, methyl acrylate, 3-oxopropanoate, or 3,3-diethoxypropanoate failed, and also an attempted hydrolysis—Arndt-Eistert homologation sequence on compound **26** was futile. Thus, we decided to introduce the propionate moiety (*en route* to target seven-membered analogue **6**, Figure 1) earlier in the synthesis.

**Scheme 6:**
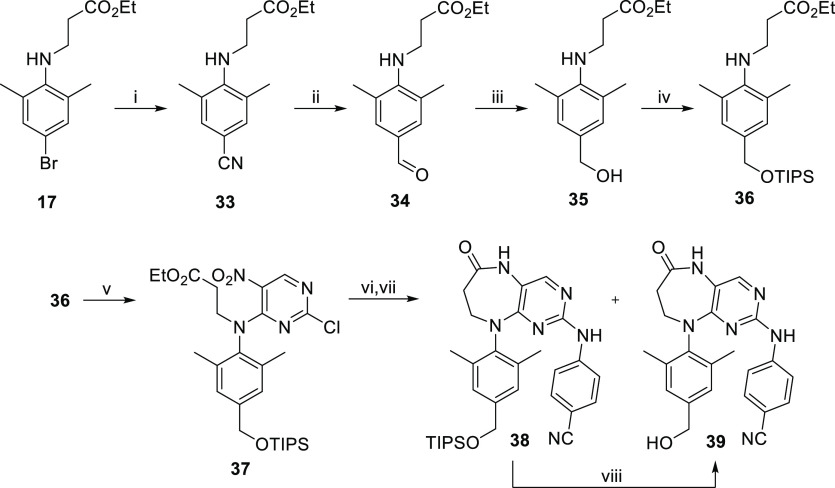
Synthetic Route for Hydroxymethyl Derivative **39** Reagents and conditions: (i) Zn(CN)_2_, Pd(*t*-Bu_3_P)_2_ (13.5 mol %), DMF, 100 °C, 5 h, 87%; (ii) HCO_2_H, Ra–Ni, 80 °C, 15 min, 75%; (iii) NaBH_4_, EtOH, 25 °C, 45 min, 85%; (iv) TIPSCl, AgNO_3_, pyridine, 25 °C, 4.5 h, 89%; (v) 2,4-dichloro-5-nitropyrimidine (**8**), DIPEA, dioxane, 55 °C, 5 h, 52%; (vi) 4-aminobenzonitrile, *i*-PrOH, 150 °C (MW), 2 h; (vii) SnCl_2_·2H_2_O, Sc(OTf)_3_ (20 mol %), EtOH, 50–60 °C, 20 h, 17% of **38** and 20% of **39** (two steps); (viii) TBAF, THF, 0 to 25 °C, 4 h, 86%.

Hence, the above-prepared intermediate **17** (Scheme 3) was selected as a suitable starting material. Since an attempted direct introduction of the formyl moiety by lithiation/trapping with DMF failed due to the retro-aza-Michael addition, compound **17** was subjected to a palladium-catalyzed cyanation, giving aryl nitrile **33** in an 87% yield (Scheme 6). The nitrile **33** was then reduced by aqueous formic acid over Ra–Ni to yield aldehyde **34** (75%), which was further reduced with NaBH_4_ to the corresponding alcohol **35** in an 85% yield. Surprisingly, silylation of alcohol **35** under common reaction conditions (*e*.*g*., TIPSCl and imidazole in DMF or TIPSOTf and 2,6-lutidine in DCM) failed. Fortunately, alcohol **35** afforded silyl ether **36** on treatment with TIPSCl and silver nitrate in pyridine in an excellent yield.^47^

Silyl ether **36** then reacted with 2,4-dichloro-5-nitropyrimidine (**8**) to yield β-alaninate **37** (Scheme 6). Microwave-assisted nucleophilic aromatic substitution introduced the 4-aminobenzonitrile arm, and subsequent reductive cyclization gave pyrimidodiazepines **38** and **39** in 17 and 20% yields, respectively. Finally, the triisopropylsilyl protecting group of **38** was removed by TBAF to give the desired alcohol **39** in an 86% yield.

**Scheme 7:**
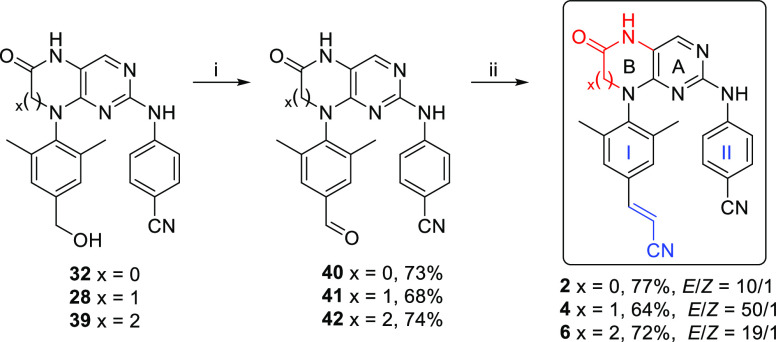
Synthetic Route for Target RPV-like Analogues **2**, **4**, and **6** Reagents and conditions: (i) DMSO, Py.SO_3_, DIPEA, THF, −10 °C, 30 min; (ii) diisopropyl (cyanomethyl)phosphonate, *n*-BuLi, 0 to 25 °C, 30 min, then aldehyde, DME, 25 °C, 1.5 h.

With the advanced intermediates **28**, **32**, and **39** in hand, oxidation of the benzyl alcohols to the corresponding aldehydes, followed by the Horner-Wardsworth-Emmons reaction, remained to be performed in order to obtain RPV analogues **2**, **4**, and **6** (Figure 1), respectively. The oxidation was optimized using compound **28**. The use of activated MnO_2_ in DMF at 25 °C led to the oxidation of benzyl alcohol to the corresponding aldehyde but also resulted in a hydroxylation of α-position of the lactam moiety (determined by 2D NMR and UPLC–MS). This finding is in accordance with the literature,^48^ where a similar system was oxidized with MnO_2_ in a DMSO/H_2_O mixture at an elevated temperature, yielding the corresponding bis-lactam. An attempted oxidation of **28** with Dess-Martin periodinane in NaHCO_3_-buffered DCM at 0 °C gave an inseparable mixture of the desired product **41** (Scheme 7) and the same hydroxylated by-product as during the previous oxidation with MnO_2_ (determined by UPLC–MS). Oxidation of **28** with pyridinium chlorochromate in DCM at 0 °C did not lead to the desired compound **41**, and the main product was identified as the corresponding carboxylic acid (UPLC–MS). However, employing Parikh–Doering conditions led to a clean formation of desired aldehyde **41** in a 68% yield (Scheme 7). Similarly, aldehydes **40** and **42** were prepared in high yields (73 and 74%, respectively) from benzyl alcohols **32** and **39**, respectively. Finally, the Horner–Wardsworth–Emmons reaction of aldehyde **41** with deprotonated diisopropyl (cyanomethyl)phosphonate in DME afforded the target tetrahydropteridine analogue **4** as a 50/1 mixture of *E*/*Z* isomers in a 64% yield. Analogously, target compounds **2** (*E*/*Z* = 10/1, 77%) and **6** (*E*/*Z* = 19/1, 72%) were prepared from the aldehyde intermediates **40** and **42**, respectively.

**Figure 3:**
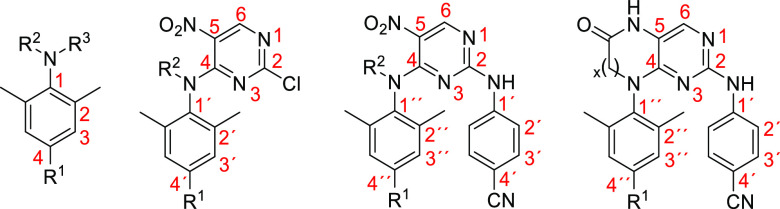
Numbering system for prepared compounds.

The numbering system used for description of NMR spectra of the prepared compounds is depicted in Figure 3.

### Biology

#### Anti-HIV-1 Biological Evaluation

The prepared compounds were tested for their anti-HIV-1 activity (Table 1). A viral cytopathic effect assay in MT-4 cells with HIV-1 IIIB virus was used to determine compound potency (EC_50_, 50% cell viability) in protecting virus induced cell killing. Compound cytotoxicity was assessed in MT-4 cells in the absence of virus (CC_50_, 50% cytotoxicity). The synthetic intermediates with modified arm I, that is, compounds **28**, **32**, **39**, and **40**–**42**, did not exhibit appreciable potency against the WT virus and, thus, were not profiled further. The designed compounds bearing the cyano group (ETV analogues **1**, **3**, and **5**) and cyanovinyl moiety (RPV analogues **2**, **4**, and **6**) at arm I were further profiled with HXB2 wild-type virus, K103N, and Y181C mutations to assess Fold-Change (FC) in potency against these resistance mutations. The bicyclic derivatives **2**, **4,** and **6** with the cyanovinyl arm I were equipotent (half-maximal effective concentration, EC_50_ = 2.2–2.8 nM) against WT/HXB2 and, in general, were more potent than their cyano arm I comparators **1**, **3**, and **5** (Table 1). ETV contains bromo and amino substitutions on the DAPY core. These substitutions have previously been shown to alter the position of ETV compared to RPV in the pocket.^15^ Likely, with the smaller cyano arm I in **1**, **3**, and **5**, the cores could be further elaborated to optimize the compound position in the pocket and to improve potency. The six-membered bicyclic analogue **3** was the most potent from the cyano-arm I series (ETV analogues), with EC_50_ values of 8.1–8.6 nM against both wild-type viruses. Interestingly, six-membered bicyclic RPV analogue **4** retained the best potency against K103N (FC 1.2x) and Y181C (FC 2.0x) mutations compared to the other compounds tested.

**Table 1:** Anti-HIV-1 Evaluation of Target Compounds **1**–**6** and of the Key Intermediates

						solubility (μM)			
comp	EC_50_ MT4 (nM)^a^	CC_50_ MT4 (μM)^b^	EC_50_ HXB2 (nM)^c^	EC_50_K103N (nM) (FC)^d^	EC_50_Y181C (nM) (FC)^d^	PBS^e^	HCl	*P*_app_^f^ (10^–6^ cm/s)	HLM^g^ CL_int_, _pred_ (L/h/kg)
ETV	2.4	7.3	2.5	2.3 (0.9)	11.1 (4.8)	<1	<1	3.8	0.25
RPV	1.0	6.5	1.2	1.5 (1.3)	3.8 (3.2)	<1	>100	4.6	0.86
1	62.3	43.7	73.7	229.7 (3.1)	241.2 (3.3)	6.6	73.2	4.9	0.53
2	2.5	23.3	2.2	4.5 (2.0)	10.6 (4.8)	10.4	61.2	5.6	1.0
3	8.6	>50	8.1	45.2 (5.6)	30.2 (3.7)	2.3	>100	4.1	1.1
4	2.7	42.4	2.4	2.8 (1.2)	4.9 (2.0)	1.8	6.5	4.2	0.98
5	43.4	>50.0	29.4	70.7 (2.4)	198.1 (6.7)	1.4	87.3	3.5	0.93
6	3.0	25.8	2.8	4.8 (1.7)	18.3 (6.5)	2.3	63.3	3.1	0.72
28	78.9	41.6	n.d.	n.d.	n.d.	n.d.	n.d.	n.d.	n.d.
32	174.2	39.7	n.d.	n.d.	n.d.	n.d.	n.d.	n.d.	n.d.
39	197.6	15.9	n.d.	n.d.	n.d.	n.d.	n.d.	n.d.	n.d.
40	209.1	>50	n.d.	n.d.	n.d.	n.d.	n.d.	n.d.	n.d.
41	76.7	14.2	n.d.	n.d.	n.d.	n.d.	n.d.	n.d.	n.d.
42	94.3	5.0	n.d.	n.d.	n.d.	n.d.	n.d.	n.d.	n.d.

a EC_50_: concentration of the compound that protects 50% of MT-4 cells against HIV-1-induced cell killing.

b CC_50_: concentration of the compound that reduces the viability of the cell cultures by 50%.

c EC_50_: concentration of the compound that protects 50% of cells against HIV-1-HXB2-induced cell killing.

d Compound potency against HIV-1 HBX2 variants (EC_50_). Ratio of compound potency against HIV-1 HXB2 K103N or Y181C variant and parental HXB2 virus, expressed in fold change (FC).

e PBS—phosphate-buffered saline.

f *P*_app_—apparent permeability coefficient.

g HLM—human liver microsomes; n.d.—not determined.

#### Drug-like Properties

By creating the B ring in the core of DAPY analogues and introducing a polar moiety, improvements in solubility at pH 7 were observed compared to ETV and RPV. The compounds bearing five-membered ring had modestly higher solubility than those with six- and seven-membered rings. Surprisingly, compound **4** had markedly reduced solubility at pH 2 compared to the other compounds.

Compounds were further tested in a Madin–Darby Canine Kidney (MDCK) assay to evaluate their potential for oral absorption. ETV and RPV have moderate permeability (*P*_app_) in the MDCK assay, with RPV being slightly better than ETV. The improved pH 7 solubility of compounds **1**–**6** did not have a substantial effect on *P*_app_. Additionally, there was no trend for better permeability when comparing arm I versus arm II when matched with the same core. The size of the B ring had the greatest influence on *P*_app_ with the trend being five-membered > six-membered > seven-membered rings (Table 1).

Next, microsome stability was profiled in purified human liver microsomes (HLM). In general, the predicted intrinsic clearance (CL_int, pred_) was moderate and comparable to RPV (Table 1).

#### X-ray Crystallography Studies

**Figure 4:**
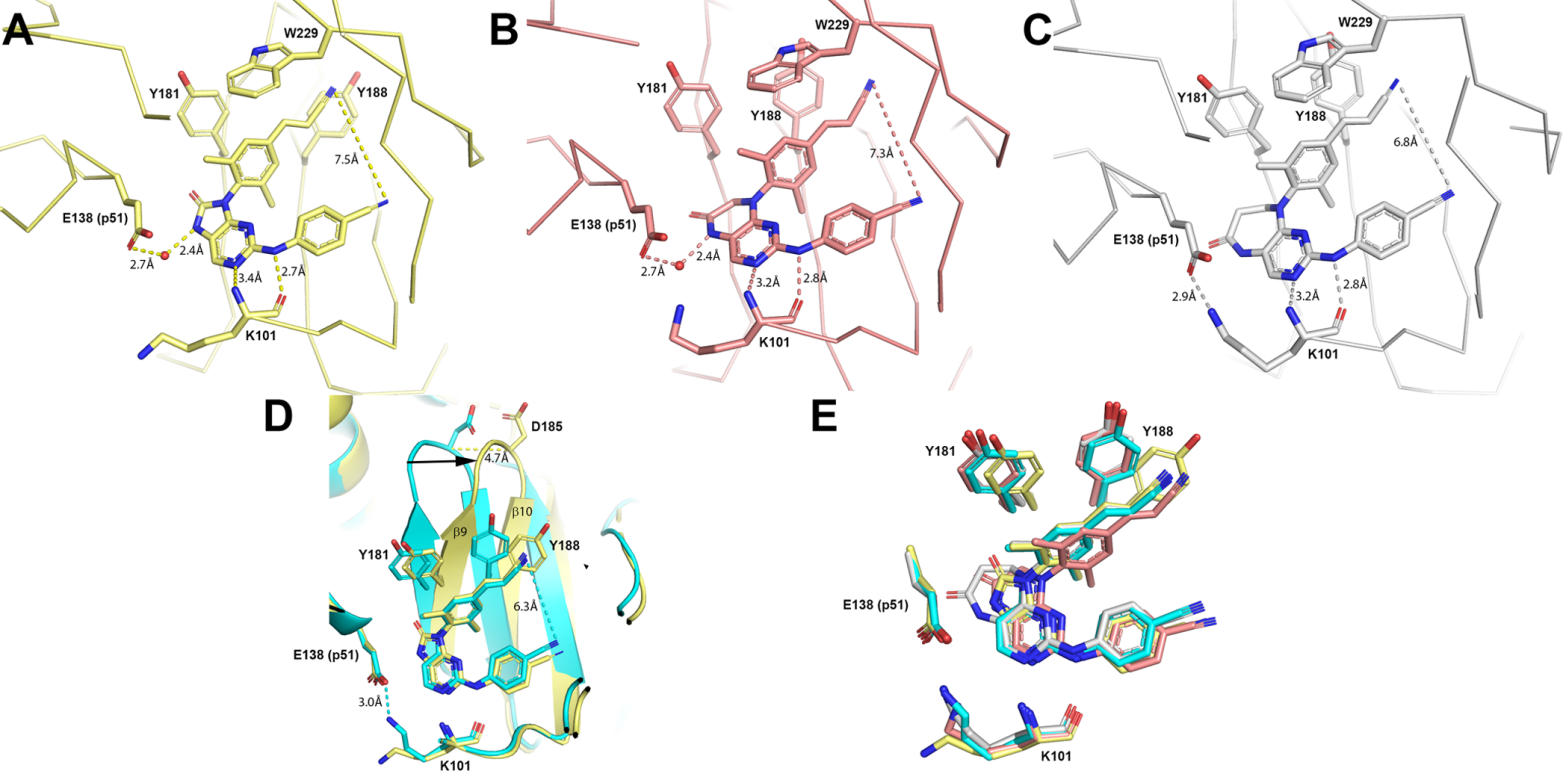
Co-crystal structure of HIV-1 reverse transcriptase (RT) with (A) **2** (yellow, PDB: 8FCC), (B) **4** (light red, PDB: 8FCD), and (C) **6** (gray, PDB: 8FCE). Dashed lines show hydrogen bonds. (D) Comparison between **2** and RPV (cyan, PDB code: 3MEE). Residue Y188 moves significantly in response to the binding of **2**, which culminates in D185 moving 4.7 Å compared to the RPV-bound structure. (E) Overall binding modes of **2**, **4**, and **6** with relevant amino acid side chains shown.

HIV-1 RT was co-crystallized with cyanovinyl compounds **2**, **4**, and **6** to determine the effect of the size of ring B on binding in the NNRTI pocket (Figure 4 and Table S1). In general, changing the core size produced the same binding mode between compounds, that is the positioning of the core is comparable, which locates arms I and II in the same place in the pocket (Figure 4). All three compounds maintain a hydrogen bond between the aniline nitrogen and the K101 backbone carbonyl (Figure 4A–C). The potential for a second hydrogen bond between the pyrimidine core nitrogen and the K101 backbone nitrogen varies between structures. This distance is longest with the five-membered ring at 3.4 Å, suggesting that it likely does not contribute significantly to binding affinity. The six- and seven-membered structures show this distance to be 3.2 Å, which is somewhat long for a hydrogen bond but implies some contribution to binding affinity. Due to an opening and solvent-accessible area around the core, there is water observed interacting with ring B in the five- and six-membered structures of compounds **2** and **4**, but not with the seven-membered ring B of compound **6**. This water is further coordinated by E138 from the p51 subunit. Previously, E138 was shown to form a salt bridge with K101 when RPV was bound in the pocket (Figure 4D). In these structures, this salt bridge is disrupted for the five- and six-membered ring B but is present for the seven-membered ring B (Figure 4A–C). Thus, it appears that E138 can either form a water-mediated bond to the NNRTI core or a salt bridge to K101 but not both. E138K is a known resistance mutation to RPV and ETR that is often observed with M184I; however, it confers only 2–3 fold resistance in cell culture.^49^ The implications of whether E138 forms a bond with a water or cross-subunit salt bridge are unclear.

The size of ring B changes the distance between arms I and II. As measured from the terminal nitrogens in arms I and II, the five-membered ring B produces a distance of 7.5 Å (Figure 4A) compared to RPV (PDB code: 3MEE) at 6.3 Å (Figure 4D). Increasing the ring size subsequently leads to shorter distances, closer to RPV, where the six- and seven-membered rings are 7.3 and 6.8 Å apart, respectively (Figure 4B,C).

The co-crystal structure with compound **2** shows the most dramatic change in protein conformation. Notably, the side chain of Y188 rotates away from Y181 and sits more parallel to the cyanovinyl (Figure 4D,E). To allow this shift, sheets β9 and β10 move, resulting in the Cα atom of D185 moving 4.7 Å compared to the RPV bound structure (Figure 4D). Because of this, several amino acid residues become disordered at the periphery of the pocket, markedly from residues 217 to 222. These protein movements are possibly due to the position of the carbonyl in ring B and/or the wider distance between the arms in the horseshoe shape. To accommodate the carbonyl, the Cβ atom in Y181 moves 1.2 Å compared to the position with RPV bound. The measured distance from the carbonyl oxygen to the Cβ is 3.5 Å. Taken together, this illustrates the flexibility that is possible with wild-type RT.

## Conclusions

A series of novel DAPY-based NNRTIs were designed based on an addition of a second non-aromatic ring to the pyrimidine core. These bicyclic NNRTIs contained either a five-membered (purine core), six-membered (tetrahydropteridine core), or seven-membered ring (pyrimidodiazepine core), and either a cyano group (etravirine analogues) or a cyanovinyl moiety (rilpivirine analogues) on arm I (*i.e.*, the *o*,*o*-dimethylphenyl moiety). The synthesis of target compounds *via* key aldehyde intermediates **I** was developed and optimized, using the Horner-Wardsworth-Emmons reaction in the last synthetic step. The aldehyde functionality in intermediates **I** offers a convenient handle for future diversification of arm I and, thus, an entry into a broader SAR study of the bicyclic NNRTIs.

All six target compounds proved to be potent antiviral agents targeting HIV-1 reverse transcriptase. Especially the cyanovinyl derivatives, that is RPV analogues **2**, **4**, and **6**, displayed single-digit nanomolar activities against the wild-type virus (EC_50_ = 2.6–3.0 nM) and low nanomolar activity against the K103N and Y181C mutated strains (Fold-Change 1.2–6.7x). All final compounds also exhibited improved phosphate-buffered saline solubility (1.4–10.4 μM) compared to ETV and RPV (≪1 μM), where the five-membered RPV analogue had the highest solubility of 10.4 μM. Furthermore, HIV-1 RT was co-crystallized with all cyanovinyl compounds, that is, derivatives **2**, **4**, and **6**. The structures reveal that as the size of ring B changes, the distance between arms I and II changes too. Additionally, since ring B is non-aromatic and somewhat flexible, all three cyanovinyl derivatives revealed analogous binging to the RT pocket and similar anti-HIV-1 potency (EC_50_ = 2.6–3.0 nM). The attachment of the second ring to the pyrimidine core of DAPY-based NNRTIs demonstrates successful structural modifications with improved drug-like properties of the inhibitors, and the prepared compounds represent an important starting point for the future design of novel anti-HIV-1 agents.

## Experimental Section

### Chemistry

#### General Information

Unless otherwise stated, all reactions were performed under an argon atmosphere utilizing standard Schlenk techniques. Glassware was dried by heating with a heat gun under high vacuum. Solvents were evaporated at 40 °C/2 mbar. Dry tetrahydrofuran was distilled from lithium aluminum hydride pellets. Acetonitrile, dimethylformamide, and dioxane were degassed by bubbling with Ar for 30 min and dried by passing through activated alumina columns in the Pure Solv system (Innovative Technology, Inc.). Ethanol and isopropanol were distilled from 3 Å molecular sieves. Other dry solvents were purchased from commercial suppliers (Sigma-Aldrich, Acros Organics). Reagents were purchased from the following commercial vendors: Sigma-Aldrich, Merck, Acros Organics, Fluorochem, Alfa Aesar, and Fluka. Microwave experiments were performed in 10 or 30 mL vials with a CEM discover (Explorer) apparatus operating at the frequency of 2.45 GHz with continuous irradiation power from 0 to 300 W. Reaction progress was routinely monitored by analytical TLC on silica gel-precoated aluminum plates with fluorescent indicator (Merck 60 F_254_) and/or UPLC–MS on a Waters Delta 600 chromatography system consisting of Waters UPLC H-class Core System (column Waters Acquity UPLC BEH C18 1.7 mm, 2.1 × 100 mm), Waters Acquity UPLC PDA detector, and mass spectrometer Waters SQD2. LC method A was used (eluent H_2_O modified with 0.1% formic acid/MeCN modified with 0.1% formic acid, gradient 0—100%, run-length 7 min) unless LC method B was used as noted (eluent H_2_O/MeCN, gradient 0—100%, run-length 7 min) and the MS method (ESI^+^ and/or ESI^–^, cone voltage = 30 V, mass detector range 100–1000 Da) was used. Flash chromatography was performed on a Teledyne ISCO CombiFlash Rf^+^ automated chromatography system using FLUKA silica gel 60 Å (230–400 mesh) for normal phase chromatography and RediSep RF Gold C18 Aq columns (20–40 μm, TELEDYNE ISCO) for reverse-phase chromatography. Preparative TLC (pTLC) was performed on Preparative UNIPLATES from ANALTECH (20 × 20 cm, 2 mm thickness, Catalog number 02015). The high-resolution mass spectra were measured on an LTQ Orbitrap XL spectrometer (Thermo Fisher Scientific) using ESI^+^, EI^+^, or CI^+^ ionization. NMR spectra were measured on Bruker Advance III HD 400, 500, and 600 instruments: ^1^H NMR spectra at 400.1, 499.9, and 600.1 MHz, respectively, and ^13^C NMR spectra at 100.6 MHz, 125.7 MHz, and 150.9 MHz, respectively. ^1^H NMR spectra are reported relative to the residual solvent (δ = 7.26 for CHCl_3_ and δ = 2.50 for DMSO) and ^13^C NMR spectra are referenced relative to the solvent signal (δ = 77.16 for CDCl_3_ and δ = 39.70 for DMSO-*d*_6_). APT, COSY, HSQC, and HMBC spectra were used to aid full assignment of NMR signals. The numbering system used for the description of the NMR spectra of the prepared compounds is depicted in Figure 3.

Data for ^1^H NMR spectra are reported as follows: chemical shift in ppm (multiplicity, coupling constant in Hz, integration, assignment) and for ^13^C NMR spectra as follows: chemical shift in ppm (assignment). Multiplicity and qualifier abbreviations are as follows: s = singlet, d = doublet, t = triplet, q = quartet, m = multiplet, and b = broad. All compounds are >95% pure by HPLC analysis.

### Synthetic Procedures

#### General Procedure for Introduction of Arm II (Method A)

Corresponding chloropyrimidine (1 equiv) and 4-aminobenzonitrile (1.1 equiv) were suspended in *i*-PrOH (0.08 M, degassed by bubbling with Ar for 15 min) in a microwave vial. The vial was sealed and heated to 150 °C for 0.5–1.5 h in the microwave reactor. The volatiles were removed, and the residue was purified by flash chromatography.

#### General Procedure for Introduction of Arm II followed by Reductive Cyclization (Method B)

Corresponding chloropyrimidine (1 equiv) and 4-aminobenzonitrile (1.1 equiv) were suspended in *i*-PrOH (0.1 M, degassed by bubbling with Ar for 15 min) in a microwave vial. The vial was sealed and heated to 150 °C for 2 h in the microwave reactor. The volatiles were removed, and the residue was purified by flash chromatography (50 g C18-SiO_2_, 0–100% MeOH/H_2_O, min. 5 column volumes (CV) 100% MeOH) fractions containing the desired nitriles were combined, evaporated, and used directly in the next step. The obtained nitriles, SnCl_2_ (5 equiv) and Sc(OTf)_3_ (0.2 equiv), were dissolved in EtOH (0.01 M) and H_2_O (10 equiv) was added. The reaction mixture was heated to 50 °C for 18–20 h. The volatiles were evaporated and the residue purified by flash chromatography.

#### General Procedure for Reduction of the Nitro Group with SnCl_2_ (Method C)

The corresponding nitropyrimidine (1 equiv) and SnCl_2_ (10 equiv) were dissolved in EtOH (0.05 M) and H_2_O (20 equiv), and the mixture was heated to 60 °C for 18 h. The volatiles were evaporated, and the residue was purified by flash chromatography (50 g C18-SiO_2_, 0–100% MeOH/H_2_O, min. 5 CV 100% MeOH) to give the amine.

#### General Procedure for Cyclization with CDI (Method D)

To the corresponding amine (1 equiv) dissolved in DCM (0.075 M) was portionwise added CDI (2.2 equiv) at 25 °C. The mixture was stirred at 25 °C for 2 h. Work-up is given for individual compounds.

#### General Procedure for Removal of the TIPS Protecting Group (Method E)

To the corresponding silyl ether (1 equiv) dissolved in THF (0.04 M) cooled to 0 °C (ice/water bath) was added TBAF (1 M in THF, 1.2 equiv), and the cooling bath was removed. The mixture was stirred at 25 °C for 4 h. The volatiles were evaporated, and the residue was purified by flash chromatography (50 g C18-SiO_2_, 0–100% MeOH/H_2_O) to give the corresponding alcohol.

#### General Procedure for Parikh–Doering Oxidation (Method F)

The corresponding alcohol (1 equiv), DMSO (20 equiv), and DIPEA (5 equiv) were dissolved in THF (0.025 M), and the mixture was cooled to −10 °C (NaCl/ice/water bath). Sulfur trioxide pyridine complex (14 equiv) was added in a spatulatipwise manner over 10 min, and the mixture was stirred at −10 °C for an additional 20 min, after which time UPLC–MS indicated full consumption of the starting material. The reaction was quenched by H_2_O (1 mL), allowed to warm to 25 °C, and the volatiles were evaporated. The residue was purified by flash chromatography (30 g C18-SiO_2_, 0–100% MeCN/H_2_O, loaded in 1 mL DMF) to give the corresponding aldehyde.

#### General Procedure for Horner–Wadsworth–Emmons Olefination (Method G)

To diisopropyl (cyanomethyl)phosphonate (1.5 equiv) in DME (0.02 M) was added *n*-BuLi (1.6 M in hexane, 2 equiv) at 0 °C, and the mixture was allowed to warm to 25 °C for 30 min. Then the corresponding neat aldehyde (1 equiv) was added, and the mixture was stirred at 25 °C for 90 min. The reaction was quenched with H_2_O (0.3 mL), the volatiles were evaporated, and the residue was purified by flash chromatography (30 g C18-SiO_2_, 0–100%MeCN/H_2_O, loaded in 1 mL DMF) to give the corresponding acrylate.

##### 4-{2-[(4-Cyanophenyl)amino]-8-oxo-8,9-dihydro-7*H*-purin-9-yl}-3,5-dimethylbenzonitrile (**1**)

From amine **16** (25 mg, 0.07 mol) according to Method D. Water (0.3 mL) was added, and the volatiles were evaporated. The residue was purified by flash chromatography (30 g of C18-SiO_2_, 0–100% MeCN/H_2_O) to give **1** (19 mg, 71%) as a white solid. ^**1**^**H NMR (500 MHz, DMSO-*d***_**6**_**): δ** 11.56 (bs, 1H, 5-NH), 9.99 (bs, 1H, 2-NH), 8.20 (s, 1H, H6), 7.91 (m, 2H, H2′), 7.81 (s, 2H, H3″), 7.65 (m, 2H, H3′), 2.12 (s, 6H, CH_3_). ^**13**^**C NMR (126 MHz, DMSO-*d***_**6**_**): δ** 154.18 (C2 or C4), 151.50 (CO), 150.66 (C2 or C4), 145.49 (C1′), 139.30 (C2″), 135.37 (C1″), 134.25 (C6), 133.13 (C3′), 132.25 (C3″), 119.94 (4′-**C**N), 118.50 (4″-**C**N), 117.67 (C2′), 117.02 (C5), 112.45 (C4″), 101.69 (C4′), 17.52 (CH_3_). **HRMS-ESI**^**+**^**[M** + **Na]**^**+**^**:** calcd, 404.1230; found, 404.1232.

##### 4-[(9-{4-[(1*E*)-2-Cyanoeth-1-en-1-yl]-2,6-dimethylphenyl}-8-oxo-8,9-dihydro-7*H*-purin-2-yl)amino]benzonitrile (**2**)

From aldehyde **40** (10 mg, 0.03 mmol) according to Method G, afforded acrylate **2** (8.2 mg, 77.4%, *E*/*Z* = 10/1) as a white solid. ^**1**^**H NMR (500 MHz, DMSO-*d***_**6**_**): δ** 11.50 (bs, 1H, 5-NH), 9.98 (s, 1H, 2-NH), 8.18 (s, 1H, H6), 7.92 (m, 2H, H2′), 7.66 (d, *J* = 16.7, 1H, 4″-C**H**=CH), 7.64 (m, 2H, H3′), 7.56 (s, 2H, H3″), 6.54 (d, *J* = 16.7, 1H, 4″-CH=C**H**), 2.07 (s, 6H, CH_3_). ^**13**^**C NMR (126 MHz, DMSO-*d***_**6**_**): δ** 154.15 (C2 or C4), 151.95 (CO), 151.04 (C2 or C4), 149.98 (4″-**C**H=CH), 145.57 (C1′), 138.01 (C2″), 134.92 (C4″), 133.89 (C6), 133.11 (C3′), 133.03 (C1″), 127.78 (C3″), 119.96 (4′-**C**N), 118.91 (CH-**C**N), 117.61 (C2′), 117.16 (C5), 101.58 (C4′), 98.35 (4″-CH=**C**H), 17.77 (CH_3_). **HRMS-ESI**^**+**^**[M** + **Na]**^**+**^**:** calcd, 430.1387; found, 430.1383. Note: NMR spectra given for the E-isomer only.

##### 4-(2-((4-Cyanophenyl)amino)-6-oxo-6,7-dihydropteridin-8(5*H*)-yl)-3,5-dimethylbenzonitrile (**3**)

Glycinate **11** (311 mg, 0.66 mmol) and iron powder (370 mg, 6.6 mmol) were suspended in MeOH/water 4:1 (30 mL). One drop of concentrated hydrochloric acid was added, and the mixture was heated to 50 °C overnight. The mixture was filtered, volatiles were evaporated, and the crude residue was purified by HPLC (Phenomenex Gemini 10u, C18, 250 × 21.2 mm, 10 mL/min, 50–100% MeCN/H_2_O) to afford compound **3** (70 mg, 27%). ^**1**^**H NMR (500 MHz, DMSO-*d***_**6**_**): δ** 10.79 (bs, 1H, 5-NH), 9.57 (s, 1H, 2-NH), 7.78 (s, 2H, H3″), 7.71 (s, 1H, H6), 7.49–7.42 (m, 2H, H2′), 7.39–7.34 (m, 2H, H3′), 4.30 (s, 2H, CH_2_), 2.21 (s, 6H, CH_3_). ^**13**^**C NMR (126 MHz, DMSO-*d***_**6**_**): δ** 161.70 (CON), 154.83 (C2), 149.13 (C4), 145.72 (C1′), 142.98 (C1″), 139.18 (C6), 139.00 (C2″), 132.71 (C3″ or C3′), 132.70 (C3″ or C3′), 120.00 (4′-CN), 118.86 (4″-CN), 117.42 (C2′), 113.12 (C5), 111.10 (C4″), 101.24 (C4′), 50.25 (CH_2_), 17.56 (CH_3_). **HRMS-ESI**^**+**^**[M** + **Na]**^**+**^**:** calcd, 396.1567; found, 396.1563.

##### 4-[(8-{4-[(1*E*)-2-Cyanoeth-1-en-1-yl]-2,6-dimethylphenyl}-6-oxo-5,6,7,8-tetrahydropteridin-2-yl)amino]benzonitrile (**4**)

From aldehyde **41** (10 mg, 0.03 mmol) according to Method G, afforded acrylate **4** (6.8 mg, 64.3%, *E*/*Z* = 50/1) as a white solid. ^**1**^**H NMR (401 MHz, DMSO-*d***_**6**_**): δ** 10.75 (bs, 1H, 5-NH), 9.54 (bs, 1H, 1′-NH), 7.69 (d, *J* = 16.7, 1H, 4″-CH), 7.69 (s, 1H, H6), 7.55 (s, 2H, H3″), 7.48 (m, 2H, H2′), 7.31 (m, 2H, H3′), 6.53 (d, *J* = 16.7, 1H, 4″-CH=C**H**), 4.29 (s, 2H, NCH_2_), 2.18 (s, 6H, CH_3_). ^**13**^**C NMR (126 MHz, DMSO-*d***_**6**_**): δ** 161.71 (CON), 154.86 (C2), 150.31 (4″-**C**H), 149.33 (C4), 145.75 (C1′), 140.77 (C1″), 138.85 (C6), 137.48 (C2″), 133.73 (C4″), 132.64 (C3′), 128.28 (C3″), 119.96 (4′-**C**N), 119.09 (CH-**C**N), 117.39 (C2′), 113.04 (C5), 101.05 (C4′), 97.48 (4″-CH=**C**H), 50.52 (NCH_2_), 17.76 (CH_3_). **HRMS-ESI**^**+**^**[M** + **Na]**^**+**^**:** calcd, 444.1543; found, 444.1544.

##### 4-{2-[(4-Cyanophenyl)amino]-6-oxo-5*H*,6*H*,7*H*,8*H*,9*H*-pyrimido[4,5-*b*][1,4]diazepin-9-yl}-3,5-dimethylbenzonitrile (**5**)

To nitrile **19** (16 mg, 0.03 mmol) and zinc cyanide (10.6 mg, 0.09 mmol) in DMF (1 mL) was added Pd(*t*-Bu_3_P)_2_ (4.6 mg, 0.009 mmol), and the mixture was heated to 110 °C until UPLC–MS indicated full consumption of the starting material (2 h). DMF was evaporated and the residue purified by flash chromatography (30 g of C18-SiO_2_, 50–100% MeCN/H_2_O). Fractions containing the product were evaporated, and the residue was used directly in the next step. The crude intermediate was dissolved in EtOH (3 mL), and SnCl_2_ (28.2 mg, 0.15 mmol), Sc(OTf)_3_ (2.9 mg, 0.006 mmol), and water (0.015 mL) were added. The resulting mixture was heated to 50 °C for 24 h. The volatiles were evaporated, and the residue was purified by flash chromatography (30 g of C18-SiO_2_, 0–100% MeCN/H_2_O) to give **5** (6.5 mg, 54% over two steps) as a white solid. ^**1**^**H NMR (500 MHz, DMSO-*d***_**6**_**): δ** 9.74 (bs, 1H, 2-NH), 9.72 (bs, 1H, 5-NH), 7.95 (s, 1H, H6), 7.83 (s, 2H, H3″), 7.23 (m, 2H, H3′), 7.10 (m, 2H, H2′), 3.83 (m, 2H, NCH_2_), 2.91 (m, 2H, COCH_2_), 2.15 (s, 6H, CH_3_). ^**13**^**C NMR (126 MHz, DMSO-*d***_**6**_**): δ** 172.38 (CON), 154.67 (C2), 152.55 (C4), 149.65 (C6), 148.45 (C1″), 145.36 (C1′), 138.11 (C2″), 132.71 (C3″), 132.38 (C3′), 119.82 (4′-**C**N), 118.90 (4″-**C**N), 117.00 (C2′), 111.51 (C5), 110.15 (C4″), 101.26 (C4′), 47.51 (NCH_2_), 36.69 (CO**C**H_2_), 17.62 (CH_3_). **HRMS-ESI**^**+**^**[M** + **H]**^**+**^**:** calcd, 410.1724; found, 410.1718.

##### 4-[(9-{4-[(1*E*)-2-Cyanoeth-1-en-1-yl]-2,6-dimethylphenyl}-6-oxo-5*H*,6*H*,7*H*,8*H*,9*H*-pyrimido[4,5-*b*][1,4]diazepin-2-yl)amino]benzonitrile (**6**)

From aldehyde **42** (10 mg, 0.024 mmol) according to Method G, afforded acrylate **6** (7.6 mg, 72%, *E*/*Z* = 19/1) as a white solid. ^**1**^**H NMR (500 MHz, DMSO-*d***_**6**_**): δ** 9.70 (s, 1H, 2-NH), 9.69 (s, 1H, 5-NH), 7.92 (s, 1H, H6), 7.75 (d, *J* = 16.7, 1H, 4″-CH), 7.58 (s, 2H, H3″), 7.13 (s, 4H, H3′ and H2′), 6.56 (d, *J* = 16.7, 1H, CHCN), 3.83 (m, 2H, NCH_2_), 2.91 (m, 2H, COCH_2_), 2.12 (s, 6H, CH_3_). ^**13**^**C NMR (126 MHz, DMSO-*d***_**6**_**): δ** 172.42 (CON), 154.74 (C2), 152.76 (C4), 150.39 (4″-**C**H), 149.48 (C6), 146.44 (C1″), 145.40 (C1′), 136.69 (C2″), 133.00 (C4″), 132.41 (C3′), 128.34 (C3″), 119.83 (4′-**C**N), 119.09 (CH**C**N), 117.08 (C2′), 111.42 (C5), 101.11 (C4′), 97.06 (**C**HCN), 47.80 (NCH_2_), 36.73 (CO**C**H_2_), 17.91 (CH_3_). **HRMS-ESI**^**+**^**[M** + **H]**^**+**^**:** calcd, 436.1880; found, 436.1876.

##### 4-[(2-Chloro-5-nitropyrimidin-4-yl)amino]-3,5-dimethylbenzonitrile (**9**)

To 2,6-dichloro-5-nitropyrimidine **8** (665 mg, 3.4 mmol) dissolved in dioxane (15 mL) were added DIPEA (0.9 mL, 5.2 mmol) and 4-amino-2,5-dimethylbenzonitrile **7** (500 mg, 3.4 mmol) sequentially at 25 °C. The mixture was heated to 50 °C for 18 h. The volatiles were evaporated, and the residue was purified by flash chromatography (120 g of SiO_2_, 0–30% cyclohexane/EtOAc) to give nitrile **9** (568 mg, 54.7%) as a yellow solid. ^**1**^**H NMR (500 MHz, DMSO-*d***_**6**_**): δ** 10.41 (bs, 1H, NH), 9.17 (s, 1H, H6), 7.68 (s, 2H, H3′), 2.17 (s, 6H, CH_3_). ^**13**^**C NMR (126 MHz, DMSO-*d***_**6**_**): δ** 162.62 (C2 or C4), 157.96 (C6), 154.46 (C2 or C4), 139.59 (C1′), 138.02 (C2′), 131.82 (C3′), 127.97 (C5), 118.91 (CN), 110.54 (C4′), 18.02 (CH_3_). **HRMS-CI**^**+**^**[M** + **H]**^**+**^**:** calcd, 304.0601; found, 304.0599. Note: compound **9** hydrolyzed in DMSO upon standing, and NMR spectra were recorded immediately after dissolution.

##### Ethyl *N*-(2-Chloro-5-nitropyrimidin-4-yl)-*N*-(4-cyano-2,6-dimethylphenyl)glycinate (**10**)

Amine **9** (2.14 g, 7.0 mmol) was dissolved in DMF (150 mL). The solution was cooled down to 0 °C, and sodium hydride (705 mg, 17.5 mmol, 60% in mineral oil) was added in one portion. After stirring the mixture for 15 min at 0 °C, ethyl bromoacetate (3.89 mL, 35 mmol) was added, and the resulting mixture was heated to 50 °C overnight. The solvent was evaporated, and the residue was purified by flash chromatography (120 g SiO_2_, 20–50% *iso*-hexane/ethyl acetate) to afford compound **10** (578 mg, 21%). ^**1**^**H NMR (600 MHz, DMSO-*d***_**6**_**): δ** 8.87 (s, 1H, H6), 7.69–7.65 (m, 2H, H3′), 4.59 (s, 2H, NCH_2_), 4.20 (q, *J* = 7.1 Hz, 2H, OCH_2_), 2.20 (t, *J* = 0.7 Hz, 6H, 2′-CH_3_), 1.25 (t, *J* = 7.1 Hz, 3H, CH_2_**CH**_**3**_). ^**13**^**C NMR (151 MHz, DMSO-*d***_**6**_**): δ** 167.86 (CO), 159.57 (C2), 156.55 (C6), 153.00 (C4), 142.96 (C1′), 137.50 (C2′), 133.17 (C3′), 132.59 (C5), 118.25 (CN), 111.50 (C4′), 61.11 (OCH_2_), 53.71 (NCH_2_), 17.86 (2′-CH_3_), 14.30 (CH_2_**CH**_**3**_). **HRMS-ESI**^**+**^**[M** + **H]**^**+**^**:** calcd, 390.0964; found, 390.0966.

##### Ethyl *N*-(4-Cyano-2,6-dimethylphenyl)-*N*-(2-((4-cyanophenyl)amino)-5-nitropyrimidin-4-yl)glycinate (**11**)

Glycinate **10** (150 mg, 0.39 mmol) and 4-aminobenzonitrile (46 mg, 0.39 mmol) were placed into a microwave vial (10 mL) and flushed with argon. Isopropyl alcohol (1 mL) was added, and the vial was sealed with a Teflon septum. The vial was heated to 150 °C for 30 min. The reaction mixture was evaporated, and the crude product was suspended in MeOH. Solids were filtered off and washed with hexane (1 mL) and diethyl ether (0.5 mL) to afford compound **11** (99 mg, 54%). ^**1**^**H NMR (500 MHz, DMSO-*d***_**6**_**): δ** 10.74 (bs, 1H, NH), 8.78 (s, 1H, H6), 7.88–7.82 (m, 2H, H2′), 7.80–7.73 (m, 2H, H3′), 7.64 (s, 2H, H3″), 4.66 (s, 2H, NCH_2_), 3.92 (q, *J* = 7.1 Hz, 2H, OCH_2_), 2.20 (s, 6H, 2″-CH_3_), 1.05 (t, *J* = 7.1 Hz, 3H, CH_2_**CH**_**3**_). ^**13**^**C NMR (126 MHz, DMSO-*d***_**6**_**): δ** 168.28 (CO), 158.25 (C2), 156.88 (C6), 154.33 (C4), 144.71 (C1″), 143.26 (C1′), 137.21 (C2″), 133.15 (C3″ or C3′), 133.11 (C3″ or C3′), 127.44 (C5), 120.38 (C2′), 119.35 (4′-CN), 118.49 (4″-CN), 110.51 (C4″), 104.75 (C4′), 60.95 (OCH_2_), 52.98 (NCH_2_), 18.12 (2″-CH_3_), 13.91 (CH_2_**CH**_**3**_). **HRMS-ESI**^**+**^**[M** + **H]**^**+**^**:** calcd, 472.1728; found, 472.1725.

##### Ethyl 2-[(4-Bromo-2,6-dimethylphenyl)(2-chloro-5-nitropyrimidin-4-yl)amino]acetate (**13**)

Aniline **12** (2.0 g, 10.0 mmol) and DIPEA (2.60 mL, 15.0 mmol) were added to compound **8** (1.94 g, 10.0 mmol) in dioxane (20 mL). The reaction mixture was heated to 60 °C for 3 h. The volatiles were evaporated, and the residue was suspended in MeCN (10 mL) under sonication. Solids were filtered off and washed with hexanes (10 mL) and Et_2_O (10 mL) to give the first crop of *N*-(4-bromo-2,6-dimethylphenyl)-2-chloro-5-nitropyrimidin-4-amine (**S1**, 1.40 g) as a yellow solid. The mother liquor was absorbed on silica gel and purified by flash chromatography (40 g SiO_2_, 0–20% cyclohexane/EtOAc) to give a second crop of the product. Combined yield: 1.70 g, 87%. Intermediate **S1:**^**1**^**H NMR (400 MHz, DMSO-*d***_**6**_**)**: **δ** 10.25 (s, 1H, NH), 9.14 (s, 1H, H6), 7.40 (s, 2H, H3′), 2.12 (s, 6H, CH_3_). ^**13**^**C NMR (101 MHz, DMSO-*d***_**6**_**): δ** 162.56 (C2), 157.77 (C6), 154.55 (C4), 138.52 (C2′ or C1′), 134.11 (C2′ or C1′), 130.56 (C3′), 127.77 (C5), 120.43 (C4′), 17.85 (CH_3_). **HRMS-ESI**^**+**^**[M** + **H]**^**+**^**:** calcd, 356.9748; found, 356.9748.

NaH (313 mg, 7.89 mmol, 60% in mineral oil) was added to the obtained amine **S1** (1.40 g, 3.9 mmol) in DMF (50 mL) at 0 °C. The mixture was stirred for 20 min, ethyl bromoacetate (2.20 mL, 20 mmol) was added, and the mixture was warmed to 25 °C overnight. The reaction mixture was then heated to 60 °C for 5 h. The reaction was cooled to 25 °C and ethyl bromoacetate (0.88 mL, 7.8 mmol) was added, and the reaction mixture was heated to 60 °C overnight. The reaction mixture was diluted with EtOAc (100 mL) and washed with NH_4_Cl (sat. aq, 3 × 100 mL). The organic layer was dried with MgSO_4_. The residue was purified by flash chromatography (120 g SiO_2_, 0–40% cyclohexane/EtOAc) to give compound **13** (1.15 g, 66%, 57% over two steps) as a yellow solid. ^**1**^**H NMR (500 MHz, DMSO-*d***_**6**_**): δ** 8.82 (s, 1H, H6), 7.40–7.36 (m, 2H, H3′), 4.53 (s, 2H, NCH_2_), 4.19 (q, *J* = 7.1 Hz, 2H, OCH_2_), 2.14 (t, *J* = 0.7 Hz, 6H, 2′-CH_3_), 1.24 (t, *J* = 7.1 Hz, 3H, CH_2_C**H**_**3**_). ^**13**^**C NMR (126 MHz, DMSO-*d***_**6**_**): δ** 167.90 (CO), 159.37 (C4), 156.19 (C6), 153.20 (C2), 138.32 (C1′), 138.21 (C2′), 132.63 (C5), 131.96 (C3′), 121.84 (C4′), 61.04 (OCH_2_), 53.97 (NCH_2_), 17.78 (2′-CH_3_), 14.32 (CH_2_**CH**_**3**_). **HRMS-ESI**^**+**^**[M** + **H]**^**+**^**:** calcd, 443.0116; found, 443.0116.

##### 4-{[8-(4-Bromo-2,6-dimethylphenyl)-6-oxo-5,6,7,8-tetrahydropteridin-2-yl]amino}benzonitrile (**14**)

From compound **13** (222 mg, 0.50 mmol) according to Method A. The reaction was then repeated five more times on a 1 mmol-scale, all reaction mixtures were joint before purification, thus, 5.5 mmol of **13** as starting material were used in total. Reaction time: 30 min. Instead of usual chromatography, the reaction was purified as follows: the combined reaction mixtures were cooled to room temperature and filtered. The solids were washed with minimal amount of ice cold *i*-PrOH, MeOH and Et_2_O, respectively, to give ethyl 2-[(4-bromo-2,6-dimethylphenyl)({2-[(4-cyanophenyl)amino]-5-nitropyrimidin-4-yl})amino]acetate **S2** (2.19 g, 76%) as yellow solid. ^**1**^**H NMR (400 MHz, DMSO-*d***_**6**_**): δ** 10.66 (bs, 1H, NH), 8.73 (s, 1H, H6), 7.89–7.79 (m, 2H, H2′), 7.78–7.71 (m, 2H, H3′), 7.33 (s, 2H, H3″), 4.60 (s, 2H, NCH_2_), 3.93 (q, *J* = 7.1 Hz, 2H, OCH_2_), 2.15 (s, 6H, 2″-CH_3_), 1.06 (t, *J* = 7.1 Hz, 3H, CH_2_**CH**_**3**_). ^**13**^**C NMR (101 MHz, DMSO-*d***_**6**_**): δ** 168.28 (CO), 158.12 (C2), 156.55 (C6), 154.49 (C4), 143.37 (C1′), 139.96 (C1″), 137.94 (C2″), 133.06 (C3′), 131.87 (C3″), 127.51 (C5), 120.73 (CN), 120.22 (C2′), 119.34 (C4″), 104.56 (C4′), 60.86 (OCH_2_), 53.24 (NCH_2_), 18.01 (2″-CH_3_), 13.90 (CH_2_**CH**_**3**_). **HRMS-ESI**^**+**^**[M** + **H]**^**+**^**:** calcd, 525.0880; found, 525.0880.

##### Method 1 (Reduction with Fe/HCl)

Ethyl 2-[(4-bromo-2,6-dimethylphenyl)({2-[(4-cyanophenyl)amino]-5-nitropyrimidin-4-yl})amino]acetate **S2** (185 mg, 0.35 mmol) and iron powder (197 mg, 3.5 mmol) were suspended in MeOH/water 4:1 (20 mL). One drop of concentrated hydrochloric acid was added, and the mixture was heated to 50 °C overnight. The mixture was filtered, volatiles were evaporated, and the crude residue was purified by HPLC (Phenomenex Gemini 10u, C18, 250 × 21.2 mm, 10 mL/min, 50–100% MeCN/H_2_O) to afford nitrile **14** (16.8 mg, 8% over two steps).

##### Method 2 (Reduction with H_2_/Ra–Ni)

To ethyl 2-[(4-bromo-2,6-dimethylphenyl)({2-[(4-cyanophenyl)amino]-5-nitropyrimidin-4-yl})amino]acetate **S2** (720 mg, 1.4 mmol) in MeOH/H_2_O (4/1, 100 mL) was added Raney nickel (50% w/w suspension in H_2_O, tip-full of 1 mL Pasteur pipette). The atmosphere was exchanged for H_2_, and the mixture was heated to 50 °C for 20 h. Further Raney nickel (50% w/w suspension in H_2_O, tip-full of a 1 mL Pasteur pipette) was added at 25 °C, and the mixture was heated to 60 °C for 16 h. The reaction mixture was cooled to 25 °C and filtered through diatomaceous earth. The volatiles were evaporated, and the residue was purified by repeated flash chromatography (40 g C18-SiO_2_, 0–100% MeOH/H_2_O) to give nitrile **14** (110 mg, 14% over two steps) as a yellow solid. ^**1**^**H NMR (401 MHz, DMSO-*d***_**6**_**): δ** 10.74 (bs, 1H, 5-NH), 9.55 (s, 1H, 1′-NH), 7.69 (s, 1H, H6), 7.52–7.47 (m, 4H, H3″, H2′), 7.38–7.33 (m, 2H, H3′), 4.28 (s, 2H, CH_2_), 2.15 (s, 6H, CH_3_). ^**13**^**C NMR (101 MHz, DMSO-*d***_**6**_**): δ** 161.65 (CON), 154.81 (C2), 149.23 (C4), 145.76 (C1′), 139.48 (C2″), 138.84 (C6), 137.78 (C1″), 132.53 (C3′), 131.42 (C3″), 121.07 (C4″), 119.92 (CN), 117.43 (C2′), 113.01 (C5), 101.02 (C4′), 50.47 (CH_2_), 17.40 (CH_3_). **HRMS-ESI**^**+**^**[M** + **H]**^**+**^**:** calcd, 449.0720; found, 449.0717.

##### 4-({2-[(4-Cyanophenyl)amino]-5-nitropyrimidin-4-yl}amino)-3,5-dimethylbenzonitrile (**15**)

According to Method A. From chloro-pyrimidine **9** (300 mg, 0.99 mmol). Reaction time 1 h. Flash chromatography (50 g C18-SiO_2_, 0–100% MeOH/H_2_O, min 20 CV 100% MeOH) gave compound **15** (236 mg, 62.0%) as a pale orange solid. ^**1**^**H NMR (500 MHz, DMSO-*d***_**6**_**): δ** 10.70 (bs, 1H, NH), 10.33 (bs, 1H, NH), 9.12 (s, 1H, H6), 7.78 (s, 2H, H3″), 7.39 (bs, 4H, H2′and H3′), 2.18 (s, 6H, CH_3_). ^**13**^**C NMR (126 MHz, DMSO-*d***_**6**_**): δ** 159.32 (C2 or C4), 157.88 (C6), 154.73 (C2 or C4), 143.57 (C1′), 141.74 (C1″), 137.96 (C2″), 132.66 (C3′), 131.83 (C3″), 121.88 (C5), 119.30 (4′-**C**N), 119.14 (C2′), 119.04 (4″-**C**N), 109.88 (C4″), 104.34 (C4′), 17.99 (CH_3_). **HRMS-ESI**^**+**^**[M** + **Na]**^**+**^**:** calcd, 408.1179; found, 408.1176. Note: the product elutes in 100% MeOH during chromatography as a risen UV-absorption baseline (ca 0.15 AU at 250 nm). About 50 × 20 mL fractions were collected. The solid crude product can be more conveniently purified by recrystallization from MeOH, resulting in a slightly lower yield.

##### 4-({5-Amino-2-[(4-cyanophenyl)amino]pyrimidin-4-yl}amino)-3,5-dimethylbenzonitrile (**16**)

From 5-nitropyrimidine **15** (75 mg, 0.20 mmol) according to Method C, afforded compound **16** (57 mg, 82.4%) as off-white solid. ^**1**^**H NMR (500 MHz, DMSO-*d***_**6**_**): δ** 9.23 (bs, 1H, 2-NH), 8.19 (bs, 1H, 4-NH), 7.70 (s, 2H, H3″), 7.64 (s, 1H, H6), 7.51 (m, 2H, H2′), 7.36 (m, 2H, H3′), 4.59 (bs, 2H, NH_2_), 2.17 (s, 6H, CH_3_). ^**13**^**C NMR (126 MHz, DMSO-*d***_**6**_**): δ** 151.57 and 151.49 (C2 and C4), 146.34 (C1′), 142.41 (C1″), 139.28 (C6), 137.72 (C2″), 132.62 (C3′), 131.79 (C3″), 122.25 (C5), 120.23 (4′-**C**N), 119.27 (4″-**C**N), 116.60 (C2′), 108.62 (C4″), 99.86 (C4′), 18.28 (CH_3_). **HRMS-ESI**^**+**^**[M** + **H]**^**+**^**:** calcd, 356.1618; found, 356.1615.

##### Ethyl 3-[(4-Bromo-2,6-dimethylphenyl)amino]propanoate (**17**)

To 4-bromo-2,5-dimethylaniline **12** (8.0 g, 40 mmol), potassium iodide (6.64 g, 40 mmol), and potassium carbonate (8.28 g, 60 mmol) in MeCN (80 mL) in a round-bottom flask equipped with a reflux condenser was added ethyl 3-bromopropionate (15.4 mL, 120 mmol), and the heterogeneous mixture was heated to 65 °C for 3 days. Then further ethyl 3-bromopropionate (8.0 mL, 62 mmol) was added, and the mixture was heated to 55 °C for an additional 2 days. After cooling to 25 °C, the solids were removed by filtration (washing with EtOAc). The filtrate was diluted with sat. Na_2_S_2_O_3_ (200 mL) and EtOAc (200 mL). The organic phase was separated, and the aqueous phase was extracted with EtOAc (2 × 150 mL). The combined organic phases were washed with sat. NaHCO_3_ (150 mL) and brine (150 mL) and dried over MgSO_4_. The volatiles were evaporated and the residue purified by flash chromatography (700 g SiO_2_, 0–20% cyclohexane/EtOAc) to yield compound **17** (5.54 g, 46.2% uncorrected yield), which was 90% pure (NMR analysis, contaminated by the product of double alkylation) as yellow oil. ^**1**^**H NMR (401 MHz, CDCl**_**3**_**): δ** 7.12 (s, 2H, H3), 4.17 (q, *J* = 7.1, C**H**_**2**_CH_3_), 3.54 (bs, 1H, NH), 3.19 (t, *J* = 6.1, 2H, NCH_2_), 2.54 (t, *J* = 6.0, 2H, C**H**_**2**_CO_2_Et), 2.25 (s, 6H, 2-CH_3_), 1.28 (t, *J* = 7.1, 3H, CH_2_C**H**_**3**_). ^**13**^**C NMR (101 MHz, CDCl**_**3**_**): δ** 172.88 (CO), 144.69 (C1), 132.26 (C2), 131.40 (C3), 114.69 (C4), 60.82 (**C**H_2_CH_3_), 43.51 (NCH_2_), 35.15 (**C**H_2_CO_2_Et), 18.32 (2-**C**H_3_), 14.36 (CH_2_**C**H_3_). **HRMS-EI**^**+**^**[M]**^**+**^**:** calcd, 299.0521; found, 299.0520.

##### Ethyl 3-[(4-Bromo-2,6-dimethylphenyl)(2-chloro-5-nitropyrimidin-4-yl)amino]propanoate (**18**)

To 2,6-dichloro-5-nitropyrimidine **8** (970 mg, 5.0 mmol) dissolved in dioxane (7 mL) were added DIPEA (0.87 mL, 5.0 mmol) and propanoate **17** (1.00 g, 3.3 mmol) sequentially at 25 °C. The mixture was heated to 70 °C for 8 h. The volatiles were evaporated, and the residue was purified by flash chromatography (120 g SiO_2_, 0–20% cyclohexane/EtOAc), followed by another flash chromatography (50 g C18-SiO_2_, 50–100% MeCN/H_2_O) to give propanoate **18** (254 mg, 16.7%) as a thick yellow oil which solidified on standing. ^**1**^**H NMR (500 MHz, DMSO-*d***_**6**_**): δ** 8.75 (s, 1H, H6), 7.39 (s, 2H, H3′), 4.09 (t, *J* = 7.2, 2H, NCH_2_), 3.98 (q, *J* = 7.1, 2H, OCH_2_), 2.70 (t, *J* = 7.2, 2H, NCH_2_C**H**_**2**_), 2.07 (s, 6H, 2′-CH_3_), 1.12 (t, *J* = 7.1, 3H, CH_2_C**H**_**3**_). ^**13**^**C NMR (126 MHz, DMSO-*d***_**6**_**): δ** 170.78 (COO), 159.82 (C2), 156.07 (C6), 153.18 (C4), 138.09 (C2′), 137.13 (C1′), 132.77 (C5), 132.08 (C3′), 121.90 (C4′), 60.51 (OCH_2_), 48.15 (NCH_2_), 31.39 (NCH_2_**C**H_2_), 17.89 (2′-CH_3_), 14.12 (CH_2_**C**H_3_). **HRMS-ESI**^**+**^**[M** + **H]**^**+**^**:** calcd, 457.0273; found, 457.0273. Note: compound **18** was hydrolyzed in DMSO upon standing, and NMR spectra were recorded immediately after dissolution.

##### Ethyl 3-[(4-Bromo-2,6-dimethylphenyl)({2-[(4-cyanophenyl)amino]-5-nitropyrimidin-4-yl})amino]propanoate (**19**)

According to Method A. From chloro-pyrimidine **18** (70 mg, 0.15 mmol). Reaction time 1.5 h. Flash chromatography (50 g of C18-SiO_2_, 50–100% MeCN/H_2_O) gave compound **19** (45 mg, 54.6%) as a yellow solid. ^**1**^**H NMR (500 MHz, DMSO-*d***_**6**_**): δ** 10.71 (bs, 1H, NH), 8.69 (s, 1H, H6), 7.96 (m, 2H, H2′), 7.74 (m, 2H, H3′), 7.36 (s, 2H, H3″), 4.12 (m, 2H, NCH_2_), 3.98 (q, *J* = 7.1, 2H, OCH_2_), 2.74 (m, 2H, NCH_2_C**H**_**2**_), 2.08 (s, 6H, 2″-CH_3_), 1.10 (t, *J* = 7.1, CH_2_C**H**_**3**_). ^**13**^**C NMR (126 MHz, DMSO-*d***_**6**_**): δ** 170.93 (COO), 158.45 (C2), 156.69 (C6), 154.35 (C4), 143.83 (C1′), 138.66 (C1″), 137.78 (C2″), 133.22 (C3′), 132.05 (C3″), 127.6* (C5), 120.89 (C4″), 119.96 (C2′), 119.44 (CN), 104.33 (C4′), 60.42 (OCH_2_), 47.99 (NCH_2_), 31.14 (NCH_2_**C**H_2_), 18.08 (2″-**C**H_3_), 14.13 (CH_2_**C**H_3_). **HRMS-ESI**^**+**^**[M** + **H]**^**+**^**:** calcd, 539.1037; found, 539.1028. *Very weak resonance, assigned by HMBC.

##### *N*′-(4-Bromo-2,6-dimethylphenyl)-*N*,*N*-dimethylmethanimidamide (**21**)

Methanimidamide derivative **21** was prepared by a slight alteration of a known literature procedure.^45^ 4-Bromo-2,5-dimethylaniline **12** (50 g, 250 mmol) in DCM (160 mL) was added dropwise to a suspension of Arnold’s reagent (38.4 g, 300 mmol) in DCM (320 mL) at 25 °C. The mixture was vigorously stirred at 25 °C for 60 min before being cooled by an ice/water bath and quenched with 2 M aqueous NaOH (300 mL, internal temperature was maintained below 15 °C). The organic layer was separated, washed with H_2_O (250 mL), dried over MgSO_4_, and evaporated. The residue was co-evaporated with cyclohexane (300 mL) to give 62.38 g (97.8%) of the title compound as brown oil. The ^1^H NMR spectra of **21** in DMSO-*d*_6_ and CDCl_3_ were in agreement with the reported data.^45,50^^**13**^**C NMR (101 MHz, CDCl**_**3**_**): δ** 153.32 (N=CH), 149.37 (C1), 132.07 (C2 or C4), 130.37 (C3), 114.38 (C2 or C4), 39.99 (N–CH_3_), 34.17 (N–CH_3_), 18.62 (2-**C**H_3_). **HRMS-ESI**^**+**^**[M** + **H]**^**+**^**:** calcd, 255.0491; found, 255.0491.

##### *N*′-(4-Formyl-2,6-dimethylphenyl)-*N*,*N*-dimethylmethanimidamide (**22**)

Methanimidamide **22** was prepared by a slight modification of a literature procedure.^44^*n*-BuLi (92 mL, 2.5 M in hexanes, 230 mmol) was added dropwise to a solution of methanimidamide **21** (48.5 g, 190 mmol) in THF (500 mL) at −78 °C over 15 min. The reaction mixture was stirred at −78 °C for further 45 min, and DMF (147 mL, 1.9 mol) was added *via* syringe over 20 min. The mixture was stirred at the same temperature for further 1 h, then it was warmed to 0 °C, and quenched with H_2_O (350 mL). The THF was evaporated, and the aqueous layer was extracted with EtOAc (3 × 300 mL). The combined organic layers were washed with brine (300 mL) and dried over MgSO_4_ to give the crude methanimidamide **22** (40.70 g) as brown oil, which was used in the next step without further purification. ^**1**^**H NMR (401 MHz, DMSO-*d***_**6**_**): δ** 9.77 (s, 1H, CHO), 7.51 (s, 2H, H3), 7.43 (s, 1H, N=CH), 2.96 (s, 6H, NCH_3_), 2.12 (s, 6H, 2-CH_3_). ^**13**^**C NMR (101 MHz, DMSO-*d***_**6**_**): δ** 191.84 (CHO), 157.18 (C1), 153.05 (N=CH), 129.96 and 129.94 (C2 and C4), 129.52 (C3), 18.60 (2-**C**H_3_), (N–CH_3_ not observed). **HRMS-EI**^**+**^**[M]**^**+**^**:** calcd, 204.1263; found, 204.1262.

##### *N*′-[4-(Hydroxymethyl)-2,6-dimethylphenyl]-*N*,*N*-dimethylmethanimidamide (**23**)

NaBH_4_ (6.70 g, 176 mmol) was added to a solution of crude **22** (30 g) in EtOH (300 mL) portionwise at 0 °C (ice/water bath). The cooling bath was removed, and the reaction mixture was stirred at 25 °C for 1.5 h. The reaction mixture was cooled to 0 °C, and carefully quenched with saturated aqueous NH_4_Cl (200 mL), and extracted with EtOAc (3 × 300 mL). The combined organic layers were dried over MgSO_4_ and evaporated to give **23** (23.29 g, 80.6% over two steps) as brownish oil. ^**1**^**H NMR (401 MHz, DMSO-*d***_**6**_**): δ** 7.27 (s, 1H, N=CH), 6.88 (s, 2H, H3), 4.89 (t, *J* = 5.6, 1H, OH), 4.33 (d, *J* = 5.6, 2H, CH_2_), 2.92 (s, 6H, NCH_3_), 2.02 (s, 6H, 2-CH_3_). ^**13**^**C NMR (101 MHz, DMSO-*d***_**6**_**): δ** 153.59 (N=CH), 149.19 (C1), 134.84 and 128.38 (C2 and C4), 126.36 (C3), 63.19 (CH_2_), 18.71 (2-**C**H_3_), (N–CH_3_ not observed). **HRMS-ESI**^**+**^**[M** + **H]**^**+**^**:** calcd, 207.1492; found, 207.1490.

##### (4-Amino-3,5-dimethylphenyl)methanol (**24**)

Methanimidamide **23** (23.3 g, 113 mmol), LiOH·H_2_O (23.8 g, 566 mmol), and ethanolamine (20 mL, 335 mmol) were suspended in H_2_O:*i*-PrOH (230 mL, 4:1). The mixture was refluxed for 20 h, then allowed to cool to 25 °C and extracted with EtOAc (3 × 200 mL). The combined organic layers were washed with brine (200 mL), dried over MgSO_4_, and evaporated. The residue was recrystallized twice from chlorobenzene to yield **24** (12.8 g, 75.0%) as brownish needles. ^1^H NMR spectrum in CDCl_3_ is in agreement with the literature.^51^^**1**^**H NMR (401 MHz, DMSO-*d***_**6**_**): δ** 6.75 (s, 2H, H3), 4.71 (t, *J* = 5.6, 1H, OH), 4.40 (bs, 2H, NH_2_), 4.25 (d, *J* = 5.6, 2H, CH_2_), 2.06 (s, 6H, CH_3_). ^**13**^**C NMR (101 MHz, DMSO-*d***_**6**_**): δ** 143.12 (C1), 129.71 (C2 or C4), 127.06 (C3), 120.35 (C2 or C4), 63.39 (CH_2_), 18.03 (CH_3_). **HRMS-ESI**^**+**^**[M** + **Na]**^**+**^**:** calcd, 174.0889; found, 174.0889.

##### 2,6-Dimethyl-4-({[tris(propan-2-yl)silyl]oxy}methyl)aniline (**20**)

Compound **24** (5.32 g, 35 mmol) and imidazole (2.52 g, 37 mmol) were dissolved in DMF (90 mL), and TIPSCl (7.9 mL, 37 mmol) was added dropwise at 25 °C. The greenish solution was stirred at 25 °C for 6 h and then diluted with EtOAc (450 mL). The organic layer was washed with water (250 mL) and brine (200 mL), dried with MgSO_4_, and evaporated. The residue was purified by flash chromatography (120 g SiO_2_, 0–30% cyclohexane/EtOAc) to give aniline **20** (9.58 g, 88.6%) as a slightly off-white solid. ^**1**^**H NMR (400 MHz, DMSO-*d***_**6**_**): δ** 6.76 (s, 2H, H3), 4.56 (s, 2H, CH_2_), 4.43 (bs, 2H, NH_2_), 2.06 (s, 6H, 2-CH_3_), 1.15–1.01 (m, 21H, TIPS). ^**13**^**C NMR (101 MHz, DMSO-*d***_**6**_**): δ** 143.26 (C1), 128.40 (C4), 126.35 (C3), 120.41 (C2), 64.98 (CH_2_), 18.09 (2-**C**H_3_), 18.00 (CH_3_-TIPS), 11.66 (CH-TIPS). **HRMS-ESI**^**+**^**[M** + **Na]**^**+**^**:** calcd, 330.2224; found, 330.2223.

##### 2-Chloro-*N*-[2,6-dimethyl-4-({[tris(propan-2-yl)silyl]oxy}methyl)phenyl]-5-nitropyrimidin-4-amine (**25**)

To a solution of 2,6-dichloro-5-nitropyrimidine **8** (1.55 g, 8.0 mmol) in dioxane (16 mL) were added DIPEA (2.09 mL, 12 mmol) and aniline **20** (2.46 g, 8.0 mmol), sequentially at 25 °C. The mixture was heated to 60 °C for 90 min. The volatiles were evaporated, and the residue was purified by flash chromatography (120 g SiO_2_, 0–20% cyclohexane/EtOAc) to give amine **25** (3.29 g, 88.4%) as a yellow waxy solid. ^**1**^**H NMR (500 MHz, DMSO-*d***_**6**_**): δ** 10.26 (bs, 1H, NH), 9.12 (s, 1H, H6), 7.11 (s, 2H, H3′), 4.78 (s, 2H, CH_2_), 2.11 (s, 6H, 2′-CH_3_), 1.16 (m, 3H, CH-TIPS), 1.07 (d, *J* = 7.1, 18H, CH_3_-TIPS). ^**13**^**C NMR (126 MHz, DMSO-*d***_**6**_**): δ** 162.72 (C2 or C4), 157.89 (C6), 154.78 (C2 or C4), 140.85 (C4′), 135.46 (C2′), 133.10 (C1′), 127.69 (C5), 125.46 (C3′), 64.34 (CH_2_), 18.47 (2′-**C**H_3_), 18.22 (CH_3_-TIPS), 11.73 (CH-TIPS). **HRMS-ESI**^**+**^**[M** + **Na]**^**+**^**:** calcd, 487.1903; found, 487.1899. Note: compound **25** was hydrolyzed in DMSO upon standing, and NMR spectra were recorded immediately after dissolution.

##### Ethyl 2-[(2-Chloro-5-nitropyrimidin-4-yl)[2,6-dimethyl-4-({[tris(propan-2-yl)silyl]oxy}methyl)phenyl]amino]acetate (**26**)

To a solution of amine **25** (750 mg, 1.6 mmol) in DMF (22 mL) was added NaH (129 mg, 3.2 mmol, 60% in mineral oil) in 2 portions at 0 °C, and the mixture was stirred at 0 °C for 20 min. Ethyl bromoacetate (0.358 mL, 3.2 mmol) was added, and the mixture was heated to 55 °C for 16 h. The mixture was cooled to 0 °C and further NaH (60 mg, 1.50 mmol, 60% in mineral oil) was added, the mixture was stirred at 0 °C for 20 min, and ethyl bromoacetate (0.175 mL, 1.58 mmol) was added. The mixture was heated to 60 °C for 6 h. The volatiles were removed. The residue was twice co-evaporated with toluene and purified by flash chromatography (120 g SiO_2_, 0–15% cyclohexane/EtOAc) to give glycinate **26** (587 mg, 66.0%) as a yellow solid. ^**1**^**H NMR (500 MHz, DMSO-*d***_**6**_**): δ** 8.77 (s, 1H, H6), 7.05 (s, 2H, H3′), 4.74 (s, 2H, 4′-CH_2_), 4.51 (s, 2H, NCH_2_), 4.19 (q, *J* = 7.1, C**H**_**2**_CH_3_), 2.14 (s, 6H, 2′-CH_3_), 1.25 (t, *J* = 7.1, 3H, CH_2_C**H**_**3**_), 1.19–1.05 (m, 21H, TIPS). ^**13**^**C NMR (126 MHz, DMSO-*d***_**6**_**): δ** 167.89 (COO), 159.18 (C2), 155.88 (C6), 153.50 (C4), 142.03 (C4′), 137.46 (C1′), 135.04 (C2′), 132.70 (C5), 126.46 (C3′), 63.87 (4′-**C**H_2_), 60.94 (**C**H_2_CH_3_), 54.20 (NCH_2_), 18.19 (2′-CH_3_), 18.04 (CH_3_-TIPS), 14.28 (**C**H_3_CH_2_), 11.58 (CH-TIPS). **HRMS-ESI**^**+**^**[M** + **Na]**^**+**^**:** calcd, 573.2271; found, 573.2267. Note: compound **26** was hydrolyzed in DMSO upon standing, and NMR spectra were recorded immediately after dissolution.

##### 4-({8-[2,6-Dimethyl-4-({[tris(propan-2-yl)silyl]oxy}methyl)phenyl]-6-oxo-5,6,7,8-tetrahydropteridin-2-yl}amino)benzonitrile (**27**) and 4-({8-[4-(hydroxymethyl)-2,6-dimethylphenyl]-6-oxo-5,6,7,8-tetrahydropteridin-2-yl}amino)benzonitrile (**28**)

According to Method B. From nitro-pyrimidine **26** (750 mg, 1.4 mmol). Flash chromatography (50 g of C18-SiO_2_, 0–100% MeOH/H_2_O, min. 5 column volumes (CV) 100% MeOH) gave fractions containing the nitriles (UPLC–MS: desilylated *t* = 4.60 min, [M + H]^+^: 477.16; silylated *t* = 6.50 min, [M + H]^+^: 633.74), which were combined, evaporated, and used directly in the next step. From the above-obtained nitriles (max. 1.36 mmol). Reaction time: 18 h. Flash chromatography (50 g C18-SiO_2_, 0–100% MeOH/H_2_O, min. 5 CV 100% MeOH) gave **28** (70 mg, 13%) as a white solid and crude **27**, which was further purified by flash chromatography (50 g of SiO_2_, 0–15% CHCl_3_/MeOH) that gave pure **27** (228 mg, 30%) as a beige solid. Combined (**27** and **28**) yield 43.0% over two steps. Furthermore, treatment of silyl ether **27** (152 mg, 0.273 mmol) according to Method E afforded alcohol **28** (90 mg, 82%) as a white solid. Compound **27:**^**1**^**H NMR (500 MHz, DMSO-*d***_**6**_**): δ** 10.72 (bs, 1H, 5-NH), 9.55 (bs, 1H, 1′-NH), 7.67 (s, 1H, H6), 7.44 (m, 2H, H2′), 7.27 (m, 2H, H3′), 7.20 (s, 2H, H3″), 4.84 (s, 2H, 4″-CH_2_), 4.28 (s, 2H, NCH_2_), 2.16 (s, 6H, 2″-CH_3_), 1.25–1.09 (m, 21H, TIPS). ^**13**^**C NMR (126 MHz, DMSO-*d***_**6**_**): δ** 161.68 (CON), 154.89 (C2), 149.43 (C4), 145.74 (C1′), 141.26 (C4″), 138.76 (C6), 136.87 (C1″), 136.08 (C2″), 132.43 (C3′), 125.97 (C3″), 119.67 (CN), 117.25 (C2′), 112.79 (C5), 100.88 (C4′), 64.21 (4″-**C**H_2_), 50.81 (NCH_2_), 18.11 (CH_3_-TIPS), 17.83 (2″-**C**H_3_), 11.62 (CH-TIPS). **HRMS-ESI**^**+**^**[M** + **H]**^**+**^**:** calcd, 557.3055; found, 557.3051. Compound **28:**^**1**^**H NMR (500 MHz, DMSO-*d***_**6**_**): δ** 10.71 (bs, 1H, 5-NH), 9.51 (bs, 1H, 1′-NH), 7.67 (s, 1H, H6), 7.48 (m, 2H, H2′), 7.35 (m, 2H, H3′), 7.17 (s, 2H, H3″), 5.28 (bt, *J* = 5.2, 1H, OH), 4.52 (d, *J* = 4.2, 2H, 4″-CH_2_), 4.26 (s, 2H, NCH_2_), 2.15 (s, 6H, 2″-CH_3_). ^**13**^**C NMR (126 MHz, DMSO-*d***_**6**_**): δ** 161.70 (CON), 154.92 (C2), 149.51 (C4), 145.76 (C1′), 142.48 (C4″), 138.61 (C6), 136.76 (C1″), 135.90 (C2″), 132.63 (C3′), 126.99 (C3″), 119.94 (CN), 117.34 (C2′), 112.86 (C5), 100.92 (C4′), 62.73 (4″-**C**H_2_), 50.88 (NCH_2_), 17.70 (CH_3_). **HRMS-ESI**^**+**^**[M** + **H]**^**+**^**:** calcd, 401.1721; found, 401.1718.

##### 4-[(4-{[2,6-Dimethyl-4-({[tris(propan-2-yl)silyl]oxy}methyl)phenyl]amino}-5-nitropyrimidin-2-yl)amino]benzonitrile (**29**)

According to Method A. From chloro-pyrimidine **25** (400 mg, 0.86 mmol). Reaction time: 1 h. First flash chromatography (50 g of C18-SiO_2_, 0–100% MeOH/H_2_O) was followed by another chromatography (50 g of SiO_2_, 1–2% CHCl_3_/MeOH) to give nitrile **29** (268 mg, 57.0%) as a yellow solid. ^**1**^**H NMR (500 MHz, DMSO-*d***_**6**_**): δ** 10.71 (bs, 1H, NH), 10.13 (bs, 1H, NH), 9.11 (s, 1H, H6), 7.43 (m, 2H, H2′), 7.33 (m, 2H, H3′), 7.22 (s, 2H, H3″), 4.88 (s, 2H, CH_2_), 2.13 (s, 6H, 2″-C**H**_**3**_), 1.23 (m, 3H, CH-TIPS), 1.11 (d, *J* = 7.3, 18H, CH_3_-TIPS). ^**13**^**C NMR (126 MHz, DMSO-*d***_**6**_**): δ** 159.37 (C2 or C4), 157.76 (C6), 155.24 (C2 or C4), 143.63 (C1′), 140.54 (C4″), 135.59 (C2″), 134.48 (C1″), 132.58 (C3′), 125.36 (C3″), 121.57 (C5), 119.09 (C2′), 119.04 (CN), 104.17 (C4′), 64.33 (CH_2_), 18.40 (2″-**C**H_3_), 18.14 (CH_3_-TIPS), 11.64 (CH-TIPS). **HRMS-ESI**^**+**^**[M** + **Na]**^**+**^**:** calcd, 569.2667; found, 569.2661.

##### 4-[(5-Amino-4-{[2,6-dimethyl-4-({[tris(propan-2-yl)silyl]oxy}methyl)phenyl]amino}pyrimidin-2-yl)amino]benzonitrile (**30**)

From 5-nitropyrimidine **29** (250 mg, 0.46 mmol) according to Method C, afforded compound **30** (209 mg, 89%) as a beige solid. ^**1**^**H NMR (500 MHz, DMSO-*d***_**6**_**): δ** 9.20 (bs, 1H, 2-NH), 7.95 (bs, 1H, 4-NH), 7.56 (s, H, H6), 7.50 (m, 2H, H2′), 7.27 (m, 2H, H3′), 7.15 (s, 2H, H3″), 4.81 (s, 2H, CH_2_), 4.51 (bs, 2H, NH_2_), 2.13 (s, 6H, 2″-C**H**_**3**_), 1.20 (m, 3H, CH-TIPS), 1.10 (d, *J* = 7.2, 18H, CH_3_-TIPS). ^**13**^**C NMR (126 MHz, DMSO-*d***_**6**_**): δ** 152.22 and 151.81 (C2 and C4), 146.49 (C1′), 139.27 (C4″), 138.52 (C6), 135.80 (C2″), 135.63 (C1″), 132.48 (C3′), 125.43 (C3″), 121.81 (C5), 120.10 (CN), 116.61 (C2′), 99.56 (C4′), 64.45 (CH_2_), 18.67 (2″-**C**H_3_), 18.17 (CH_3_-TIPS), 11.67 (CH-TIPS). **HRMS-ESI**^**+**^**[M** + **H]**^**+**^**:** calcd, 517.3106; found, 517.3100.

##### 4-({9-[2,6-Dimethyl-4-({[tris(propan-2-yl)silyl]oxy}methyl)phenyl]-8-oxo-8,9-dihydro-7*H*-purin-2-yl}amino)benzonitrile (**31**)

From amine **30** (125 mg, 0.24 mmol) according to Method D. Sat. NaHCO_3_ (10 mL) and CHCl_3_ (30 mL) were added. The organic layer was separated, and the aqueous layer was extracted with CHCl_3_ (2 × 30 mL). The combined organic layers were dried over MgSO_4_, and the volatiles were evaporated. The residue was purified by flash chromatography (20 g of SiO_2_, 0–10% CHCl_3_/MeOH) to give compound **31** (119 mg, 90.6%) as a red solid. ^**1**^**H NMR (500 MHz, DMSO-*d***_**6**_**): δ** 11.43 (bs, 1H, 5-NH), 9.98 (s, 1H, 2-NH), 8.17 (s, 1H, H6), 7.91 (m, 2H, H2′), 7.63 (m, 2H, H3′), 7.21 (s, 2H, H3″), 4.81 (s, 2H, CH_2_), 2.04 (s, 6H, 2″-CH_3_), 1.19 (m, 3H, CH-TIPS), 1.09 (d, *J* = 7.2, 18H, CH_3_-TIPS). ^**13**^**C NMR (126 MHz, DMSO-*d***_**6**_**): δ** 154.25 (C2 or C4), 152.13 (C=O), 151.21 (C2 or C4), 145.60 (C1′), 142.74 (C4″), 136.92 (C2″), 133.72 (C6), 133.08 (C3′), 129.32 (C1″), 125.92 (C3″), 119.96 (CN), 117.61 (C2′), 116.80 (C5), 101.55 (C4′), 64.26 (CH_2_), 18.16 (CH_3_-TIPS), 17.90 (2″-**C**H_3_), 11.64 (CH-TIPS). **HRMS-ESI**^**+**^**[M** + **H]**^**+**^**:** calcd, 543.2898; found, 543.2895.

##### 4-({9-[4-(Hydroxymethyl)-2,6-dimethylphenyl]-8-oxo-8,9-dihydro-7*H*-purin-2-yl}amino)benzonitrile (**32**)

From silyl ether **31** (116 mg, 0.21 mmol) according to Method E, afforded alcohol **32** (72 mg, 87.2%) as a white solid. ^**1**^**H NMR (500 MHz, DMSO-*d***_**6**_**): δ** 11.37 (bs, 1H, 5-NH), 9.98 (s, 1H, 2-NH), 8.16 (s, 1H, H6), 7.92 (m, 2H, H2′), 7.64 (m, 2H, H3′), 7.18 (s, 2H, H3″), 5.25 (bs, 1H, OH), 4.51 (s, 2H, CH_2_), 2.04 (s, 6H, CH_3_). ^**13**^**C NMR (126 MHz, DMSO-*d***_**6**_**): δ** 154.22 (C2 or C4), 152.15 (C=O), 151.28 (C2 or C4), 145.59 (C1′), 143.99 (C4″), 136.71 (C2″), 133.56 (C6), 133.04 (C3′), 129.11 (C1″), 126.51 (C3″), 119.91 (CN), 117.58 (C2′), 116.83 (C5), 101.52 (C4′), 62.61 (CH_2_), 17.72 (CH_3_). **HRMS-ESI**^**+**^**[M** + **Na]**^**+**^**:** calcd, 409.1384; found, 409.1381.

##### Ethyl 3-[(4-Cyano-2,6-dimethylphenyl)amino]propanoate (**33**)

Pd(*t*-Bu_3_P)_2_ (200 mg, 0.39 mmol) was added to propanoate **17** (3.26 g, 10.9 mmol) and zinc cyanide (3.83 g, 32.6 mmol) in DMF (70 mL), and the heterogeneous mixture was heated to 100 °C for 1 h. Then another portion of the catalyst (200 mg) was added, and heating was continued for further 1 h, after which the addition/heating cycle was repeated. Then the last (150 mg) portion of the catalyst was added, and the mixture was heated at 100 °C for 2 h (3 × 200 mg and 1 × 150 mg catalyst additions in total, 1.47 mmol). The mixture was allowed to cool to 25 °C and filtered. The filtrate was diluted with sat. NaHCO_3_ (150 mL) and the aqueous layer was extracted with EtOAc (3 × 150 mL). The combined organic extracts were washed with brine (4 × 100 mL), dried over MgSO_4_, and evaporated. The residue was purified by flash chromatography (120 g SiO_2_, 0–20% cyclohexane/EtOAc) to give **33** (2.32 g, 86.7%) as white crystals. ^**1**^**H NMR (401 MHz, CDCl**_**3**_**): δ** 7.24 (s, 2H, H3), 4.16 (q, *J* = 7.1, 2H, C**H**_**2**_CH_3_), 4.06 (bs, 1H, NH), 3.40 (t, *J* = 6.0, 2H, NCH_2_), 2.54 (t, *J* = 6.0, C**H**_**2**_CO_2_Et), 2.27 (s, 6H, 2-CH_3_), 1.26 (t, *J* = 7.1, 3H, CH_2_C**H**_**3**_). ^**13**^**C NMR (101 MHz, CDCl**_**3**_**): δ** 172.61 (CO), 150.14 (C1), 132.89 (C3), 128.68 (C2), 119.99 (CN), 103.59 (C4), 60.98 (**C**H_2_CH_3_), 42.82 (NCH_2_), 35.15 (**C**H_2_CO_2_Et), 18.88 (2-**C**H_3_), 14.32 (CH_2_**C**H_3_). **HRMS-CI**^**+**^**[M** + **H]**^**+**^**:** calcd, 247.1447; found, 247.1445.

##### Ethyl 3-[(4-Formyl-2,6-dimethylphenyl)amino]propanoate (**34**)

Raney nickel (250 mg, wet, Sigma-Aldrich) was added to propanoate **33** (196 mg, 0.80 mmol) in aqueous formic acid (85%, 4 mL), and the mixture was heated to 80 °C for 15 min. The reaction mixture was quickly cooled to 0 °C and poured into sat. NaHCO_3_/ice (1/1, 50 mL), pH was adjusted to 8, the aqueous solution was filtered through diatomaceous earth (washing with EtOAc) and extracted with EtOAc (3 × 50 mL). The combined organic layers were washed with brine (50 mL), dried over MgSO_4_, and evaporated. Flash chromatography (20 g SiO_2_, 0–30% cyclohexane/EtOAc) gave aldehyde **34** (148 mg, 74.6%) as yellow oil. ^**1**^**H NMR (401 MHz, CDCl**_**3**_**): δ** 9.78 (s, 1H, CHO), 7.49 (s, 2H, H3), 4.19 (bs, 1H, NH, overlapped), 4.16 (q, *J* = 7.2, 2H, C**H**_**2**_CH_3_), 3.49 (t, *J* = 6.0, 2H, NCH_2_), 2.55 (t, *J* = 6.0, 2H, C**H**_**2**_CO_2_Et), 2.33 (s, 6H, 2-CH_3_), 1.26 (t, *J* = 7.2, CH_2_C**H**_**3**_). ^**13**^**C NMR (101 MHz, CDCl**_**3**_**): δ** 191.42 (CHO), 172.59 (**C**O_2_Et), 151.93 (C1), 131.41 (C3), 129.50 (C4), 127.52 (C2), 60.97 (**C**H_2_CH_3_), 42.72 (NCH_2_), 35.29 (**C**H_2_CO_2_Et), 19.19 (2-**C**H_3_), 14.33 (CH_2_**C**H_3_). **HRMS-CI**^**+**^**[M** + **H]**^**+**^**:** calcd, 250.1443; found, 250.1442.

##### Ethyl 3-{[4-(Hydroxymethyl)-2,6-dimethylphenyl]amino}propanoate (**35**)

Aldehyde **34** (207 mg, 0.83 mmol) was dissolved in EtOH (7 mL) and sodium borohydride (38 mg, 1.0 mmol) was added portionwise at 25 °C, and the mixture was stirred at 25 °C for 45 min. Then H_2_O (10 mL) was added, and the aqueous layer was extracted with EtOAc (3 × 30 mL). The combined organic layers were dried over MgSO_4_, and the volatiles were evaporated. The residue was purified by flash chromatography (20 g SiO_2_, 20–60% cyclohexane/EtOAc) to give alcohol **35** (177 mg, 84.8%) as pale yellow oil. ^**1**^**H NMR (401 MHz, CDCl**_**3**_**): δ** 7.00 (s, 2H, H3), 4.55 (s, 2H, C**H**_**2**_OH), 4.17 (q, *J* = 7.2, 2H, C**H**_**2**_CH_3_), 3.56 (bs, 1H, NH or OH), 3.23 (t, *J* = 6.1, 2H, NCH_2_), 2.56 (t, *J* = 6.0, 2H, C**H**_**2**_CO_2_Et), 2.29 (s, 6H, 2-CH_3_), 1.28 (t, *J* = 7.2, 3H, CH_2_C**H**_**3**_), NH or OH not detected. ^**13**^**C NMR (101 MHz, CDCl**_**3**_**): δ** 172.95 (CO), 145.18 (C1), 134.63 (C4), 130.29 (C2), 128.07 (C3), 65.45 (CH_2_OH), 60.78 (**C**H_2_CH_3_), 43.64 (NCH_2_), 35.27 (**C**H_2_CO_2_Et), 18.51 (2-**C**H_3_), 14.38 (CH_2_**C**H_3_). **HRMS-ESI**^**+**^**[M** + **Na]**^**+**^**:** calcd, 274.1414; found, 274.1412.

##### Ethyl 3-{[2,6-Dimethyl-4-({[tris(propan-2-yl)silyl]oxy}methyl)phenyl]amino}propanoate (**36**)

To alcohol **35** (365 mg, 1.45 mmol) dissolved in pyridine (18 mL) were added AgNO_3_ (296 mg, 1.74 mmol) and TIPSCl (0.33 mL, 1.52 mmol) sequentially at 25 °C. The mixture was stirred at 25 °C for 4.5 h. Then, H_2_O (40 mL) and EtOAc (50 mL) were added, and the resulting heterogeneous mixture was filtered through diatomaceous earth (washed with EtOAc). The organic phase was separated, and the aqueous phase was extracted with EtOAc (2 × 60 mL). The combined organic phases were washed with brine (50 mL), dried over MgSO_4_, and evaporated. The residue was purified by flash chromatography (80 g of SiO_2_, 0–20% cyclohexane/EtOAc) to give silyl ether **36** (529 mg, 89.4%) as pale yellow oil. ^**1**^**H NMR (401 MHz, CDCl**_**3**_**): δ** 6.98 (s, 2H, H3), 4.71 (s, 2H, C**H**_**2**_OTIPS), 4.17 (q, *J* = 7.1, 2H, C**H**_**2**_CH_3_), 3.50 (bs, 1H, NH), 3.21 (t, *J* = 6.1, 2H, NCH_2_), 2.56 (t, *J* = 6.1, 2H, C**H**_**2**_CO_2_Et), 2.29 (s, 6H, 2-CH_3_), 1.28 (t, *J* = 7.1, 3H, CH_2_C**H**_**3**_), 1.21–1.12 (m, 3H, CH-TIPS), 1.12–1.07 (m, 18H, CH_3_-TIPS). ^**13**^**C NMR (101 MHz, CDCl**_**3**_**): δ** 173.01 (**C**O_2_Et), 144.19 (C1), 135.47 (C4), 130.04 (C2), 126.64 (C3), 64.98 (**C**H_2_OTIPS), 60.73 (**C**H_2_CH_3_), 43.79 (NCH_2_), 35.34 (**C**H_2_CO_2_Et), 18.56 (2-CH_3_), 18.23 (CH_3_-TIPS), 14.38 (CH_2_**C**H_3_), 12.22 (CH-TIPS). **HRMS-ESI**^**+**^**[M** + **H]**^**+**^**:** calcd, 408.2929; found, 408.2922. Note: UPLC–MS method B and TLC were used to monitor reaction progress.

##### Ethyl 3-[(2-Chloro-5-nitropyrimidin-4-yl)[2,6-dimethyl-4-({[tris(propan-2-yl)silyl]oxy}methyl)phenyl]amino]propanoate (**37**)

2,6-Dichloro-5-nitropyrimidine **8** (329 mg, 1.7 mmol) was charged into a vial, and **36** (520 mg, 1.3 mmol) in dioxane (11 mL) and DIPEA (0.34 mL, 1.9 mmol) were added sequentially. The resulting mixture was heated to 55 °C for 3 h. Further 2,6-dichloro-5-nitropyrimidine **8** (110 mg, 0.56 mmol) was added, and the heating was continued for another 2 h. The volatiles were evaporated, and the residue was purified by flash chromatography (120 g of SiO_2_, 0–20% cyclohexane/EtOAc) to give propanoate **37** (373 mg, 51.7%) as yellow oil. **1H NMR (500 MHz, DMSO-*d***_**6**_**): δ** 8.70 (s, 1H, H6), 7.06 (s, 2H, H3′), 4.74 (s, 2H, 4′-CH_2_), 4.10 (t, *J* = 7.2, 2H, NCH_2_), 3.96 (q, *J* = 7.2, 2H, C**H**_**2**_CH_3_), 2.69 (t, *J* = 7.2, 2H, NCH_2_C**H**_**2**_), 2.06 (s, 6H, 2′-CH_3_), 1.19–1.03 (m, 24H, C**H**_**3**_CH_2_, CH- and CH_3_-TIPS). ^**13**^**C NMR (126 MHz, DMSO-*d***_**6**_**): δ** 170.79 (COO), 159.71 (C2), 155.88 (C6), 153.54 (C4), 142.11 (C4′), 136.16 (C1′), 134.95 (C2′), 132.85 (C5), 126.59 (C3′), 63.91 (4′-**C**H_2_), 60.48 (**C**H_2_CH_3_), 48.25 (NCH_2_), 31.41 (NCH_2_**C**H_2_), 18.34 (2′-CH_3_), 18.12 (CH_3_-TIPS), 14.12 (**C**H_3_CH_2_), 11.64 (CH-TIPS). **HRMS-ESI**^**+**^**[M** + **H]**^**+**^**:** calcd, 565.2608; found, 565.2598. Note: UPLC–MS method B was used to monitor the reaction progress. Compound **37** hydrolyzed in DMSO upon standing, and NMR spectra were recorded immediately after dissolution.

##### 4-({9-[2,6-Dimethyl-4-({[tris(propan-2-yl)silyl]oxy}methyl)phenyl]-6-oxo-5*H*,6*H*,7*H*,8*H*,9*H*-pyrimido[4,5-*b*][1,4]diazepin-2-yl}amino)benzonitrile (**38**) and 4-({9-[4-(hydroxymethyl)-2,6-dimethylphenyl]-6-oxo-5*H*,6*H*,7*H*,8*H*,9*H*-pyrimido[4,5-*b*][1,4]diazepin-2-yl}amino)benzonitrile (**39**)

According to Method B. From nitro-pyrimidine **37** (350 mg, 0.62 mmol). Flash chromatography (50 g C18-SiO_2_, 0–100% MeOH/H_2_O, min. 5 column volumes 100% MeOH) gave fractions containing the nitriles (desilylated *t* = 4.67 min, [M + H]^+^: 491.09; silylated *t* = 6.54 min, [M + H]^+^: 647.32), which were combined, evaporated and used directly in the next step. From the obtained nitriles (0.62 mmol). Reaction time: 20 h. Flash chromatography (50 g of C18-SiO_2_, 0–100% MeOH/H_2_O, min. 5 CV 100% MeOH) gave alcohol **39** (51 mg, 20%) as a white solid and crude **38**, which was further purified by preparative TLC (divided onto 2 plates, 8% MeOH/CHCl_3_) to give pure silyl ether **38** (59 mg, 17%) as a beige solid. Combined (**38** and **39**) yield 37% over two steps. Furthermore, treatment of silyl ether **38** (53 mg, 0.09 mmol) according to Method E afforded alcohol **39** (33 mg, 86%) as a white solid. Compound **38:**^**1**^**H NMR (500 MHz, DMSO-*d***_**6**_**): δ** 9.67 (bs, 1H, 2-NH), 9.65 (bs, 1H, 5-NH), 7.89 (s, 1H, H6), 7.22 (s, 2H, H3″), 7.17 (m, 2H, H3′), 7.13 (m, 2H, H2′), 4.87 (s, 2H, C**H**_**2**_OTIPS), 3.82 (m, 2H, NCH_2_), 2.89 (m, 2H, COCH_2_), 2.09 (s, 6H, CH_3_), 1.23 (m, 3H, CH-TIPS), 1.11 (d, *J* = 7.3, 18H, CH_3_-TIPS). ^**13**^**C NMR (126 MHz, DMSO-*d***_**6**_**): δ** 172.49 (CON), 154.85 (C2), 152.98 (C4), 149.33 (C6), 145.47 (C1′), 142.53 (C1″), 140.42 (C4″), 135.32 (C2″), 132.40 (C3′), 125.87 (C3″), 119.65 (CN), 117.07 (C2′), 111.32 (C5), 101.06 (C4′), 64.24 (**C**H_2_OTIPS), 48.27 (NCH_2_), 36.70 (CO**C**H_2_), 18.19 (CH-TIPS), 18.10 (2′-CH_3_) 11.65 (CH_3_-TIPS). **HRMS-ESI**^**+**^**[M** + **H]**^**+**^**:** calcd, 571.3211; found, 571.3207. Compound **39:**^**1**^**H NMR (500 MHz, DMSO-*d***_**6**_**): δ** 9.65 (bs, 2H, 2-NH and 5-NH), 7.89 (s, 1H, H6), 7.25 (m, 2H, H3′), 7.19 (s, 2H, H3″), 7.13 (m, 2H, H2′), 5.38 (bs, 1H, OH), 4.54 (s, 2H, C**H**_**2**_OH), 3.81 (m, 2H, NCH_2_), 2.89 (m, 2H, COCH_2_), 2.09 (s, 6H, CH_3_). ^**13**^**C NMR (126 MHz, DMSO-*d***_**6**_**): δ** 172.49 (CON), 154.85 (C2), 152.97 (C4), 149.27 (C6), 145.45 (C1′), 142.48 (C1″), 141.54 (C4″), 135.16 (C2″), 132.67 (C3′), 127.15 (C3″), 119.98 (CN), 117.07 (C2′), 111.28 (C5), 101.05 (C4′), 62.86 (CH_2_OH), 48.26 (NCH_2_), 36.74 (COCH_2_), 17.97 (CH_3_). **HRMS-ESI**^**+**^**[M** + **H]**^**+**^**:** calcd, 415.1878; found, 415.1874. Note: UPLC–MS method B was used to monitor the reaction progress.

##### 4-{[9-(4-Formyl-2,6-dimethylphenyl)-8-oxo-8,9-dihydro-7*H*-purin-2-yl]amino}benzonitrile (**40**)

From alcohol **32** (47 mg, 0.12 mmol), according to Method F, afforded aldehyde **40** (34 mg, 73%) as a white solid. ^**1**^**H NMR (500 MHz, DMSO-*d***_**6**_**): δ** 11.52 (bs, 1H, 5-NH), 10.03 (s, 1H, CHO), 9.98 (bs, 1H, 2-NH), 8.21 (s, 1H, H6), 7.91 (m, 2H, H2′), 7.82 (s, 2H, H3″), 7.64 (m, 2H, H3′), 2.16 (s, 6H, CH_3_). ^**13**^**C NMR (126 MHz, DMSO-*d***_**6**_**): δ** 192.90 (CHO), 154.23 (C2 or C4), 151.54 (CON), 150.78 (C2 or C4), 145.49 (C1′), 138.61 (C2″), 136.79 (C4″), 136.01 (C1″), 134.12 (C6), 133.08 (C3′), 129.42 (C3″), 119.90 (CN), 117.66 (C2′), 116.83 (C5), 101.67 (C4′), 17.72 (CH_3_). **HRMS-ESI**^**+**^**[M** + **Na]**^**+**^**:** calcd, 407.1227; found, 407.1225.

##### 4-{[8-(4-Formyl-2,6-dimethylphenyl)-6-oxo-5,6,7,8-tetrahydropteridin-2-yl]amino}benzonitrile (**41**)

From alcohol **28** (72 mg, 0.18 mmol) according to Method F, afforded aldehyde **41** (49 mg, 68%) as a white solid. ^**1**^**H NMR (500 MHz, DMSO-*d***_**6**_**): δ** 10.76 (bs, 1H, 5-NH), 10.06 (s, 1H, CHO), 9.54 (bs, 1H, 1′-NH), 7.80 (s, 2H, H3″), 7.71 (s, 1H, H6), 7.45 (m, 2H, H2′), 7.30 (m, 2H, H3′), 4.32 (s, 2H, NCH_2_), 2.26 (s, 6H, CH_3_). ^**13**^**C NMR (126 MHz, DMSO-*d***_**6**_**): δ** 193.06 (CHO), 161.56 (CON), 154.78 (C2), 149.13 (C4), 145.63 (C1′), 143.76 (C1″), 138.98 (C6), 138.03 (C2″), 135.82 (C4″), 132.56 (C3′), 129.91 (C3″), 119.83 (CN), 117.28 (C2′), 112.98 (C5), 101.06 (C4′), 50.30 (NCH_2_), 17.69 (CH_3_). **HRMS-ESI**^**+**^**[M** + **Na]**^**+**^**:** calcd, 421.1384; found, 421.1385.

##### 4-{[9-(4-Formyl-2,6-dimethylphenyl)-6-oxo-5*H*,6*H*,7*H*,8*H*,9*H*-pyrimido[4,5-*b*][1,4]diazepin-2-yl]amino}benzonitrile (**42**)

From alcohol **39** (50 mg, 0.12 mmol), according to Method F. afforded aldehyde **42** (37 mg, 74%) as a white solid. ^**1**^**H NMR (500 MHz, DMSO-*d***_**6**_**): δ** 10.09 (s, 1H, CHO), 9.71 (s, 2H, 2-NH and 5-NH), 7.94 (s, 1H, H6), 7.83 (s, 2H, H3″), 7.10 (s, 4H, H3′ and H2′), 3.85 (m, 2H, NCH_2_), 2.93 (m, 2H, COCH_2_), 2.20 (s, 6H, CH_3_). ^**13**^**C NMR (126 MHz, DMSO-*d***_**6**_**): δ** 193.21 (CHO), 172.41 (CON), 154.72 (C2), 152.67 (C4), 149.57 (C6), 149.45 (C1″), 145.35 (C1′), 137.30 (C2″), 135.29 (C4″), 132.38 (C3′), 130.09 (C3″), 119.79 (CN), 116.97 (C2′), 111.44 (C5), 101.14 (C4′), 47.63 (NCH_2_), 36.73 (COCH_2_), 17.89 (CH_3_). **HRMS-ESI**^**+**^**[M** + **H]**^**+**^**:** calcd, 413.1721; found, 413.1718.

### Biology

#### MT-4 Antiviral and Cytotoxicity Assays

Compounds were tested in a high-throughput 384-well assay format for their ability to inhibit the virus replication-induced cytopathic effect in MT-4 cell cultures acutely infected with HIV-1 (IIIB strain), HXB2, HXB2-K103N, and HXB2-Y181C. Compounds were serially diluted (1:3) in DMSO on 384-well polypropylene plates and further diluted 200-fold into complete RPMI media (10% FBS, 1% P/S) using Biotek Micro Flow and an Agilent ECHO acoustic dispenser. Each plate contained up to eight test compounds, with negative (no drug control) and 5 μM AZT positive controls. MT-4 cells were further diluted in complete RPMI media and added to each plate using a Micro-Flo dispenser. After five days of incubation in a humidified and temperature-controlled incubator (37 °C), Cell Titer Glo (Promega) was added to the assay plates to quantify the amount of luciferase. EC_50_ and CC_50_ values were defined as the compound concentration that causes a 50% decrease in luminescence signal and were calculated using a sigmoidal dose-response model to generate curve fits.

#### MDCK Assay

cMdr1-KO cells were maintained in Dulbecco’s modified Eagle’s medium (DMEM) with sodium pyruvate and GlutaMax, supplemented with 1% Pen/Strep and 10% fetal bovine serum in an incubator set at 37 °C, 90% humidity, and 5% CO_2_. MDCKII- cMdr1-KO cells were grown to confluence over 3 days on 96-well PET plates with 1 μm pore size, polyester membrane (Corning 3392). Experiments were run using HBSS donor buffer from Invitrogen containing an additional 10 mM HEPES, 15 mM glucose, and 0.1% BSA adjusted to pH 7.4. The receiver well had HBSS buffer supplemented with 1% BSA, 10 mM HEPES, 15 mM glucose, and the pH was adjusted to 7.4. TEER values were read to test membrane integrity at the beginning of the assay. The experiment was started by the addition of dosing solutions containing test compounds. Samples were taken from the donor compartment at 0/120 min and from the receiver compartment at 120 min. Each compound was tested in two separate replicate wells. All samples were immediately collected in a 72:8:20 MeCN/MeOH:H_2_O mix to precipitate protein and stabilize the test compounds. Cells were dosed on the apical side to determine forward (A to B) permeability. To test for non-specific binding and compound instability, the total amount of drug was quantified at the end of the experiment and compared to the material present in the original dosing solution as a percentage of recovery. Samples were analyzed by RF-QToF.

#### Measurement of Predicted Clearance Values in HLM

Metabolic stability of compounds was assessed using HLM (Corning cat. 457117).^52^ In this assay, 10 nL compounds at a concentration of 1 mM in 100% DMSO are dispensed into 384-well polypropylene plates using the Echo 550 acoustic liquid dispenser (Labcyte). Each plate contains 384 wells with a single test compound in each well.

A solution of HLM at 2 mg/mL in 100 mM K_2_HPO_4_/KH_2_PO_4_ pH 7.4 with 0.0225 mg/mL Alamethicin from *Trichoderma viride* (Sigma-Aldrich cat. A4665-10MG) was incubated on ice for 15 min. 5 μL of this solution was added to individual wells following 15 min incubation at room temperature; and supplemented with 5 μL NADPH Regenerating Solution of cofactors (Corning Gentest UGT Reaction Mix solutions A and B, cat. 451200 and 451220) containing 100 mM K_2_HPO_4_/KH_2_PO_4_ pH 7.4, 2.6 mM NADP^+^, 6.6 mM glucose-6-phosphate, 6.6 mM MgCl_2_, 0.8 U/mL glucose-6-phosphate dehydrogenase, 0.1 mM sodium citrate, and 6.8 mM uridine diphosphate-glucuronic acid. The final concentration of analyte compounds at the beginning of the reactions was 1 μM. The reactions were incubated at 37 °C, and time points of 0, 5, 15, 30, 40, 50, 60, and 70 min were collected for further analysis. Background data were collected using reactions without analyte compounds.

Upon collection of the reaction time points, samples were quenched at the set time points with 30 μL of a solution of 72% acetonitrile, 8% methanol, 0.1% formic acid, 19.9% water, and labetalol as an internal standard (IS) at a concentration of 650 nM. Reaction plates were span in a centrifuge at a speed of 4000 rcf for 30 min and 4 °C, following a dilution of the 10 μL quenched reaction into 40 μL de-ionized water, yielding assay plates.

Samples were analyzed by a solid-phase extraction coupled with a quadrupole time-of-flight mass spectrometer, using an Agilent QToF 6530 RapidFire 360 system with C4 type A solid state cartridges. Analysis was performed in either positive or negative ionization modes. Mobile phases contained 0.1% formic acid in water for loading analytes onto solid state extraction cartridges, and 0.1% formic acid in acetonitrile for elution into the mass spectrometer in positive ionization mode, or 0.1% acetic acid in water for loading and 0.1% acetic acid in acetonitrile for extraction in negative ionization mode. Peak-area ratios of integrated counts for individual compounds to IS were plotted as a semi-logarithmic chart of log versus time. Initial, linear portion of decay was fitted to a linear regression equation to derive the half-time of a compound decay.

Pharmacological parameters for an analyte compound metabolism were calculated using the equations described in Table S2.

#### Solubility Assessment

Compound solubility was measured at Analiza (Cleveland, OH) by the standard shake-flask method at pH 1.2 and 7.4 and quantitated by HPLC-UV.

#### Protein Crystallization

HIV-1 Reverse Transcriptase (RT) was expressed and purified as described previously.^15^ For crystallization trials, RT was concentrated to 20 mg/mL in a final buffer containing 10 mM Tris pH 7.0, 25 mM KCl, and 1 mM DTT. The compounds were dissolved into DMSO to make a stock solution at 10 mM and then mixed with RT for a final concentration of 400 μM. Crystals were obtained in 0.8–0.96 M K/Na tartrate and MES buffer pH 6.0 by hanging drop vapor diffusion at 20 °C. Crystals were introduced to a cryoprotectant solution containing 20% glycerol in addition to the above concentrations of the mother liquor components. The crystals were then cooled in a bath of liquid nitrogen.

#### Data Collection, Model Building, and Refinement

Data was collected at The Advanced Light Source on BL5.0.2 at a temperature of 100 K. Diffraction data was processed with XDS.^53^ The structure was determined by the molecular replacement method using the program Phenix^54^ with PDB code 3MEE as a search model. Additionally, simulated annealing, energy minimization, and *B*-factor refinement were carried out in Phenix.^54^ Model building was performed with the molecular graphics program Coot.^55^

## Supporting Information Available

^1^H NMR and ^13^C NMR spectra of prepared compounds, HPLC chromatograms of *in vitro* evaluated compounds, data collection and refinement statistics for X-ray structures, and pharmacological parameters for an analyte compound metabolism (PDF)
Molecular formula strings (CSV)

## Accession Codes

The atomic coordinates and structure factors have been deposited into the Protein Data Bank. The accession numbers are 8FCC, 8FCD, and 8FCE for compounds **2, 4,** and **6,** respectively. Authors will release the atomic coordinates upon article publication.

## Abbreviations Used

CDI: carbonyldiimidazole
DAPY: diarylpyrimidine
DIPEA: *N*,*N*-diisopropylethylamine
ETV: etravirine
HAART: highly active antiretroviral therapy
HIV: human immunodeficiency virus
NNRTIs: non-nucleoside reverse transcriptase inhibitors
RPV: rilpivirine
RT: reverse transcriptase
TEA: triethylamine
WT: wild type
