# Supplementary material for: Design and Synthesis of Novel HIV-1 NNRTIs with Bicyclic Cores and with Improved Physicochemical Properties
Source: J Med Chem. 2023 Jan 18;66(3):1761–77. doi: 10.1021/acs.jmedchem.2c01574 (PMC10017027; doi:10.1021/acs.jmedchem.2c01574)
Supplement: Supplementary file 1 — jm2c01574_si_001.pdf [file jm2c01574_si_001.pdf]

SUPPORTING INFORMATION

Design and Synthesis of Novel HIV-1 NNRTIs with Bicyclic Cores and with Improved Physicochemical Properties

Ladislav Prener[a], Ondřej Baszczyňski[a,b], Martin M. Kaiser[a], Martin Dračínský[a], George Stepan[c], Yu-Jen Lee[c], Boris Brumshtein[c], Helen Yu[c], Petr Jansa[c], Eric B. Lansdon*[,c], Zlatko Janeba*[,a]

[a]*Institute of Organic Chemistry and Biochemistry of the Czech Academy of Sciences, Flemingovo nám. 2, 160 00 Prague 6, Czech Republic*

[b]*Department of Organic Chemistry, Faculty of Science, Charles University, Hlavova 8, 128 43, Prague 2, Czech Republic*

[c]*Gilead Sciences Inc., 333 Lakeside Drive, Foster City, California 94404, United States*

*Address correspondence to: janeba@uochb.cas.cz (Z.J.) and Eric.Lansdon@gilead.com (E.B.L.)

Table of contents:

1. Copies of spectra of prepared compounds S2

2. Copies of HPLC chromatograms of compounds with *in vitro* data S43

3. Data collection and refinement statistics for X-ray structures (Table S1) …………. S50

4. Pharmacological parameters for an analyte compound metabolism (Table S2) …... S51

1. Copies of spectra of prepared compounds

4-{2-[(4-Cyanophenyl)amino]-8-oxo-8,9-dihydro-7H-purin-9-yl}-3,5-dimethylbenzonitrile (1):

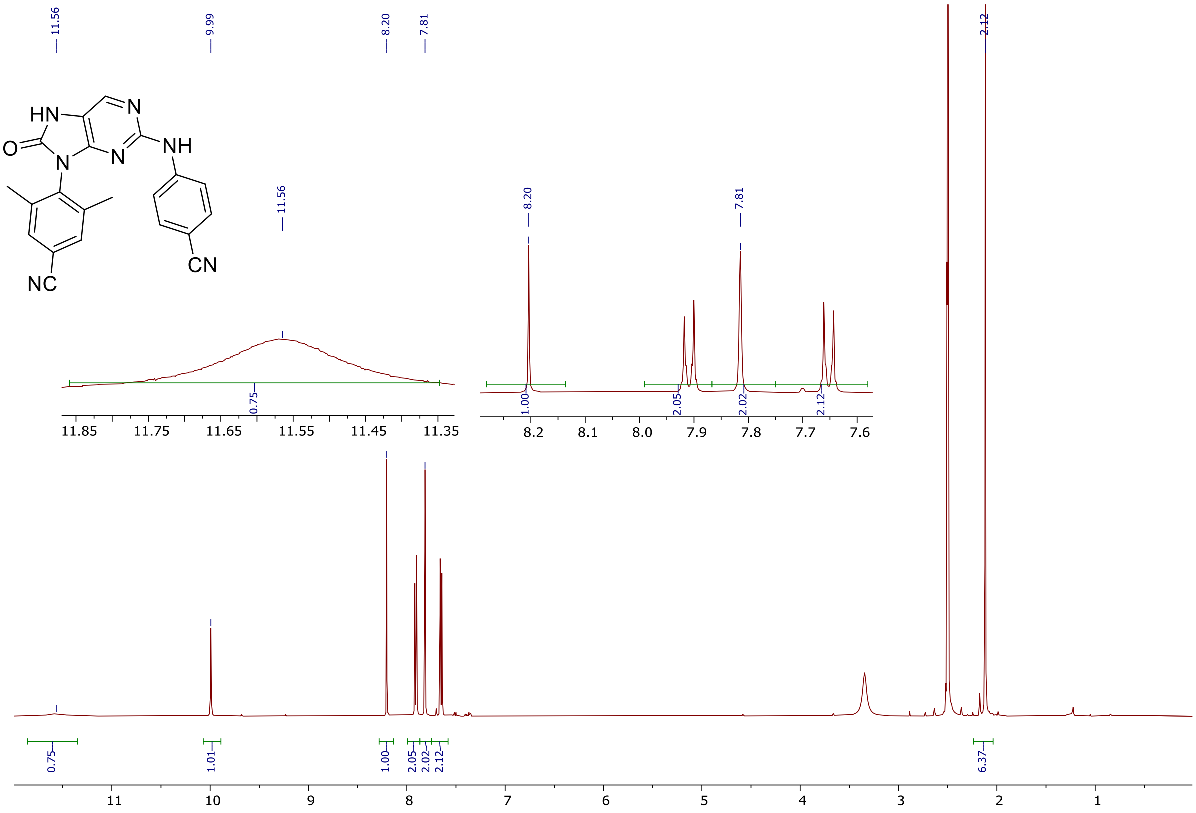

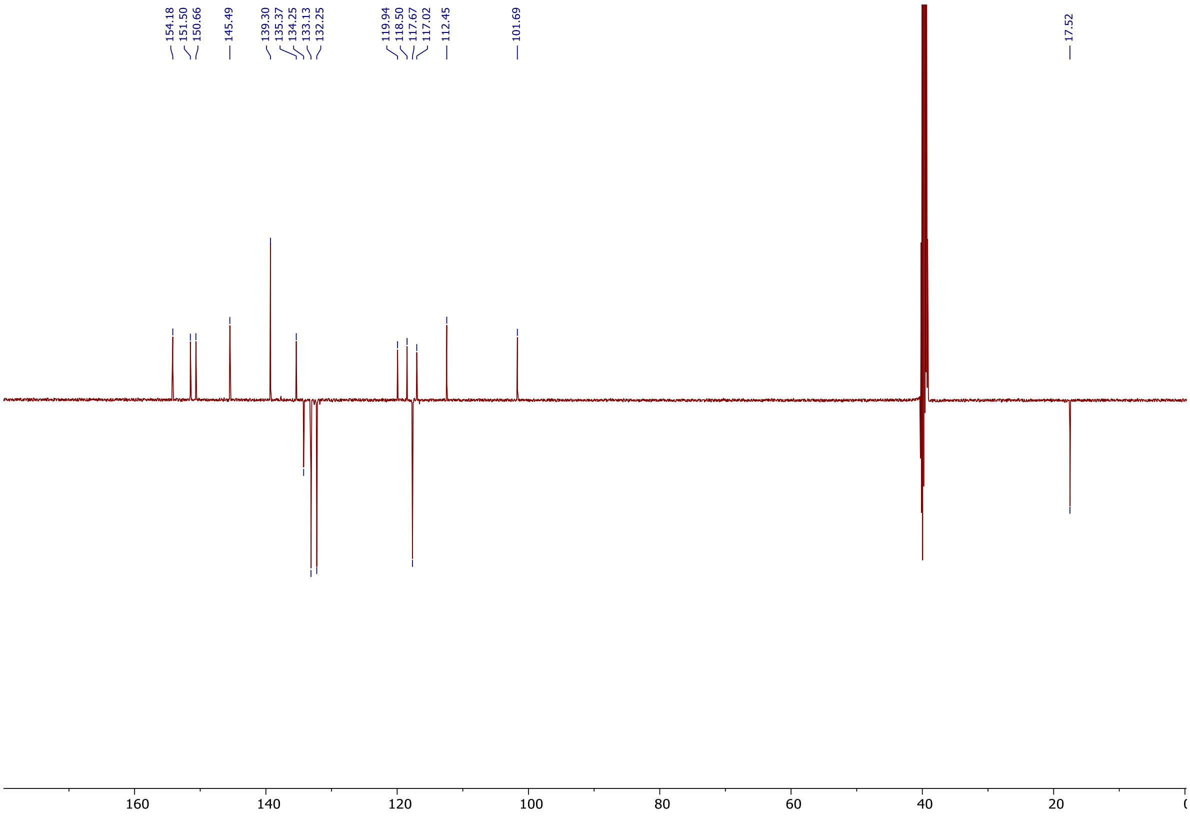

4-[(9-{4-[(1*E*)-2-Cyanoeth-1-en-1-yl]-2,6-dimethylphenyl}-8-oxo-8,9-dihydro-7*H*-purin-2-yl)amino]benzonitrile (2):

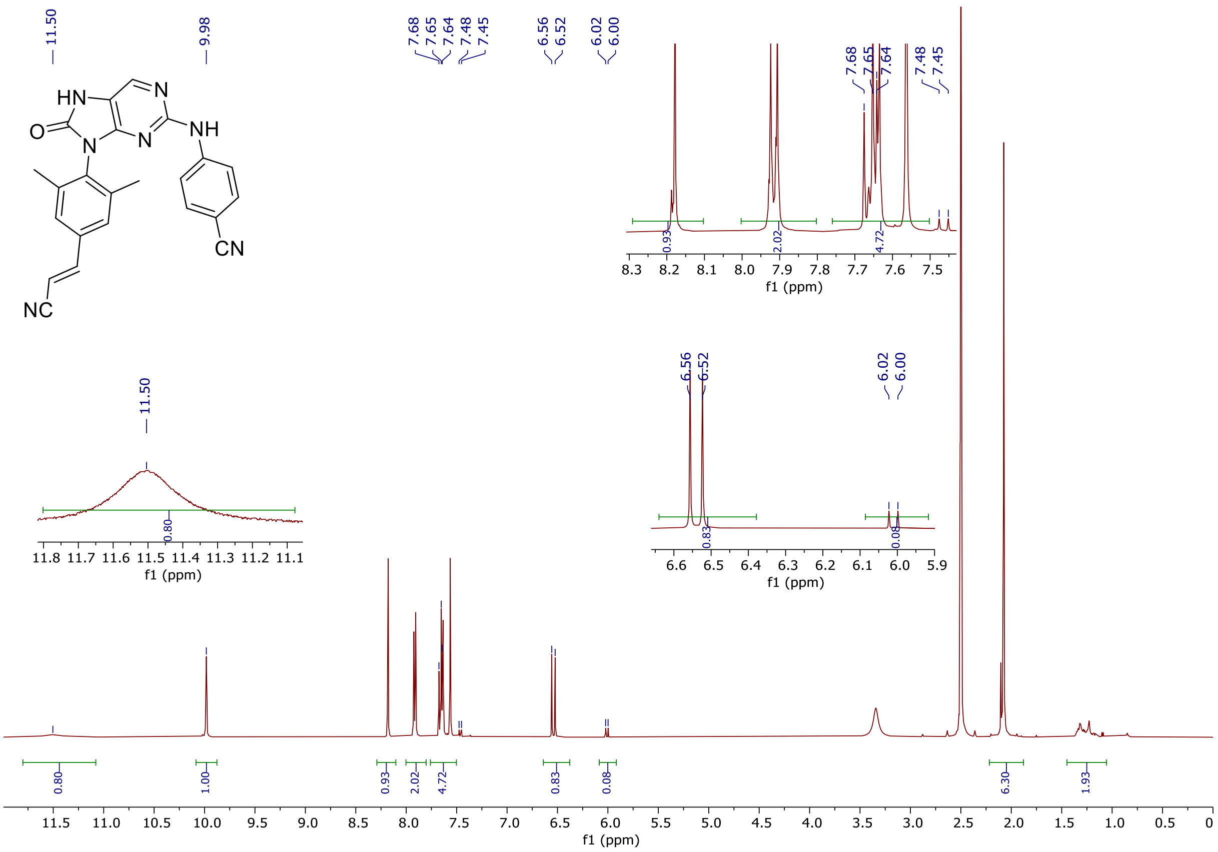

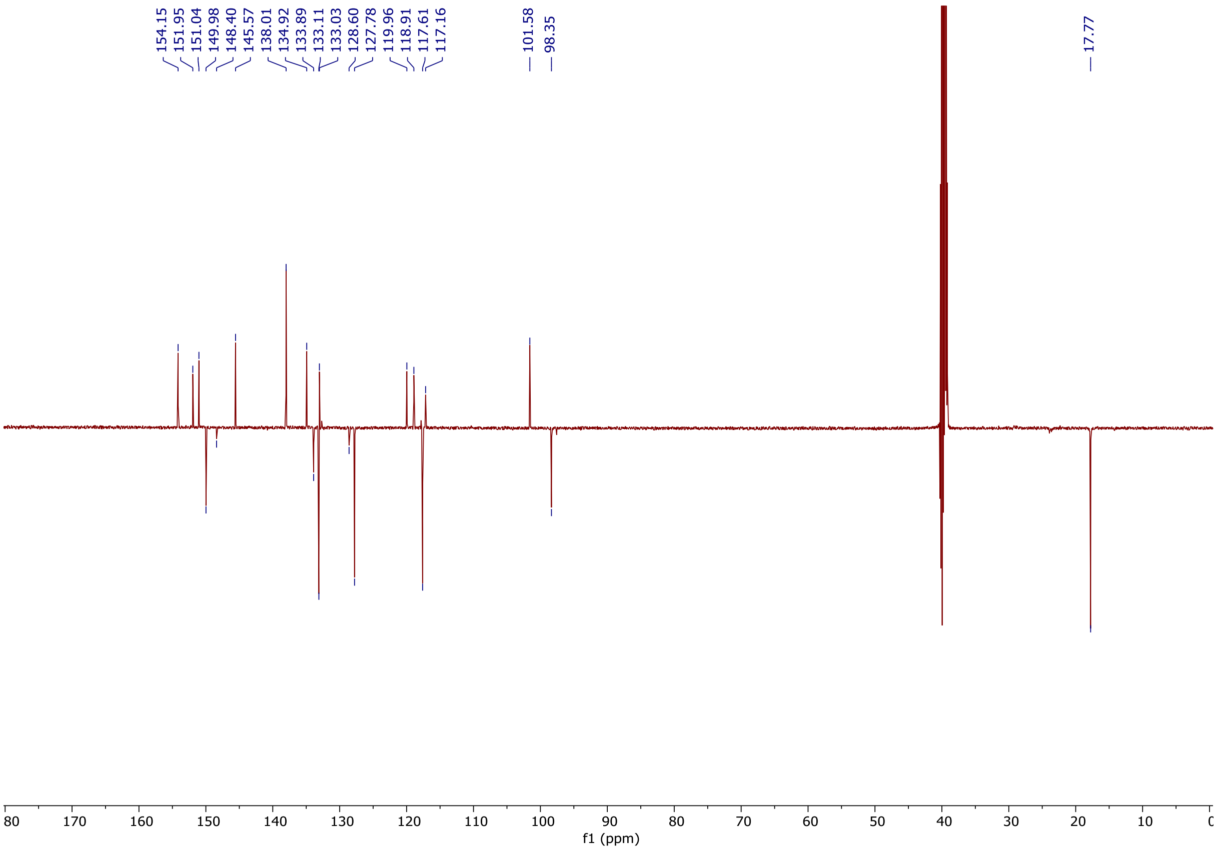

4-{2-[(4-cyanophenyl)amino]-6-oxo-6,7-dihydropteridin-8(5H)-yl}-3,5-dimethylbenzonitrile (3):

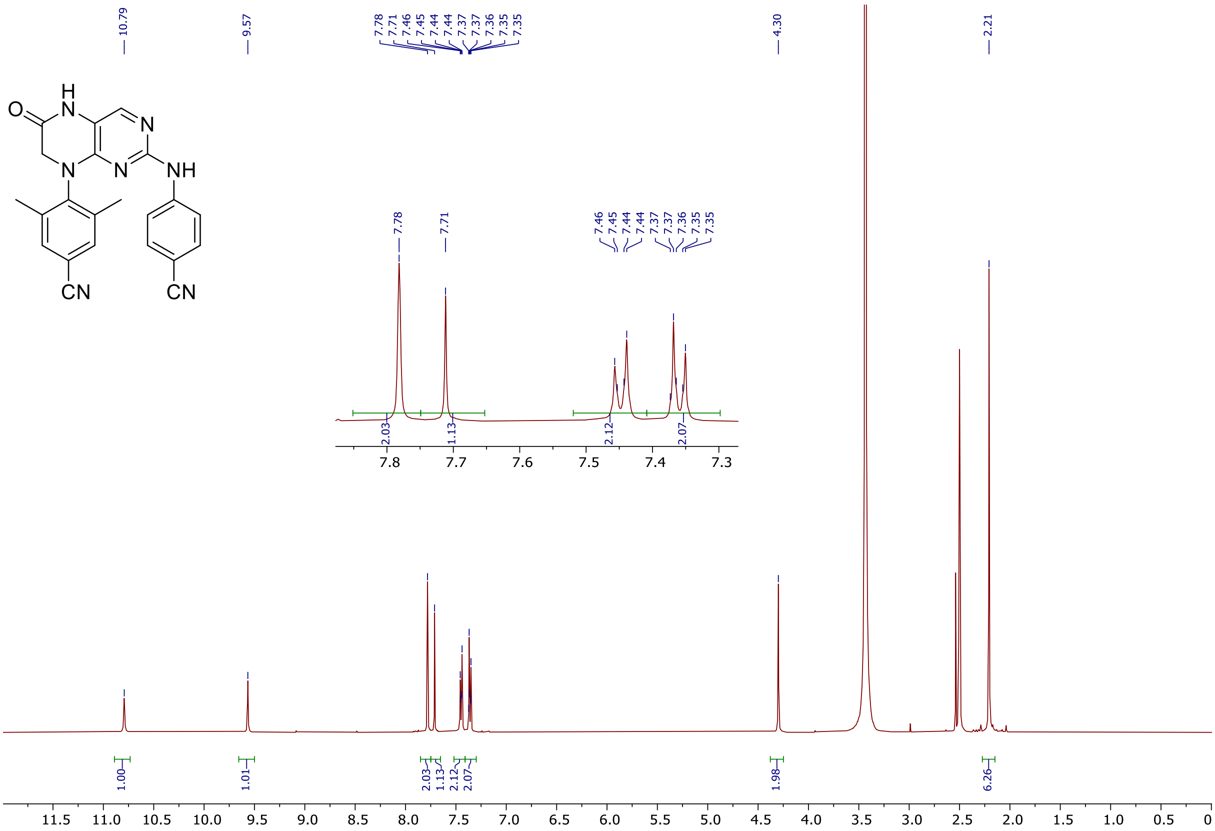

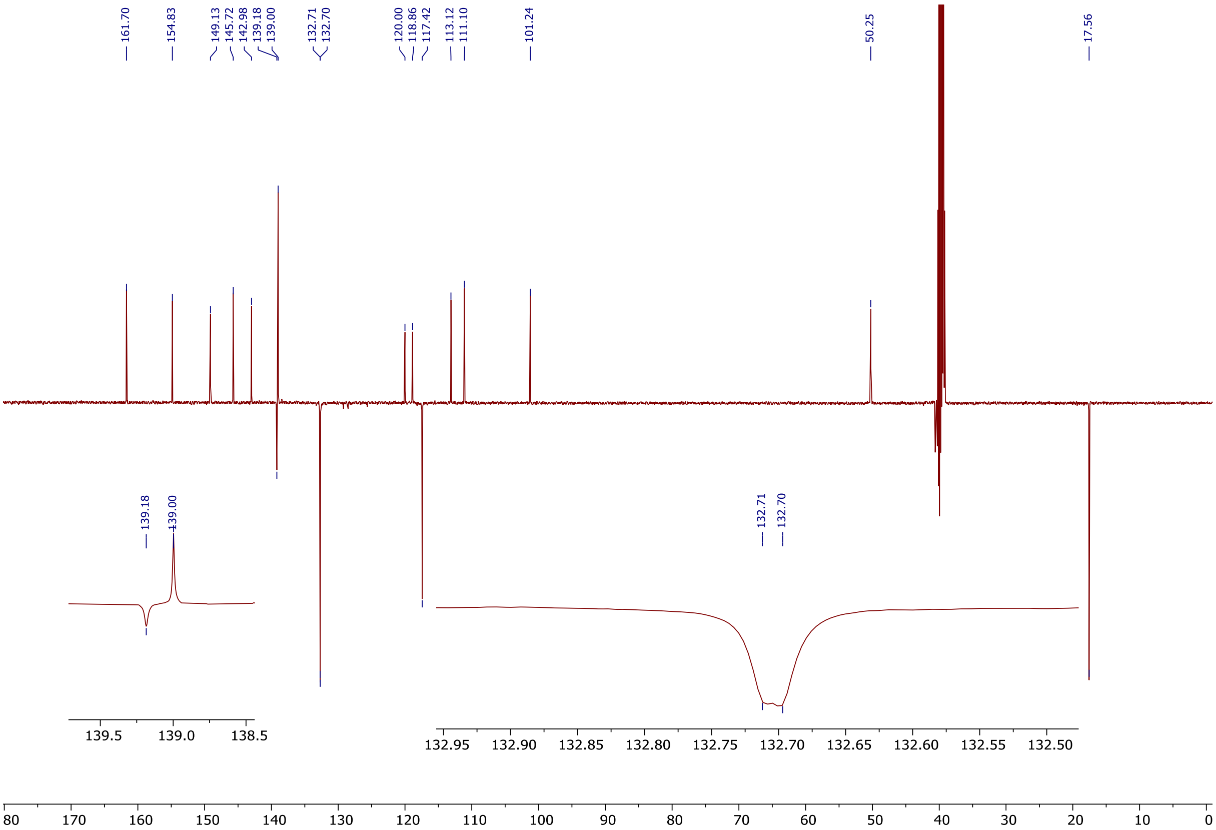

4-[(8-{4-[(1*E*)-2-Cyanoeth-1-en-1-yl]-2,6-dimethylphenyl}-6-oxo-5,6,7,8-tetrahydropteridin-2-yl)amino]benzonitrile (4):

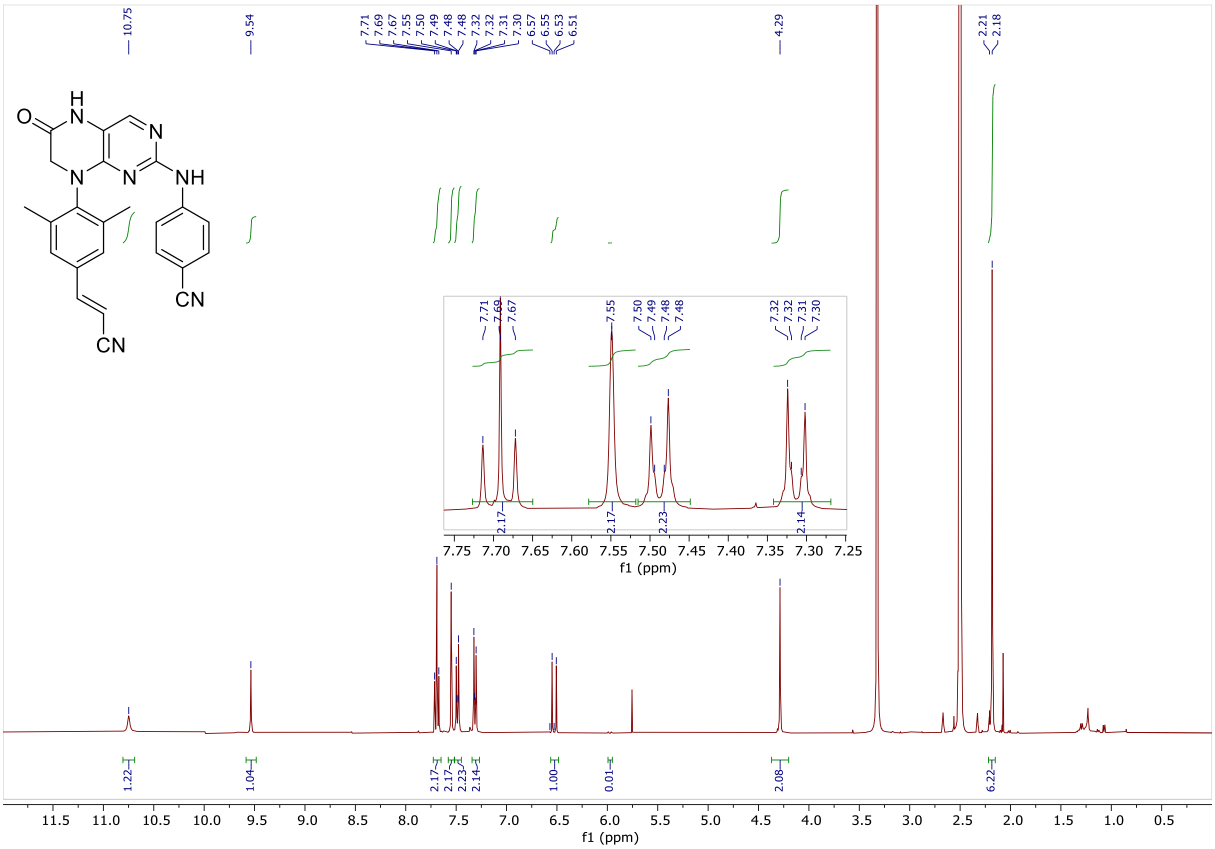

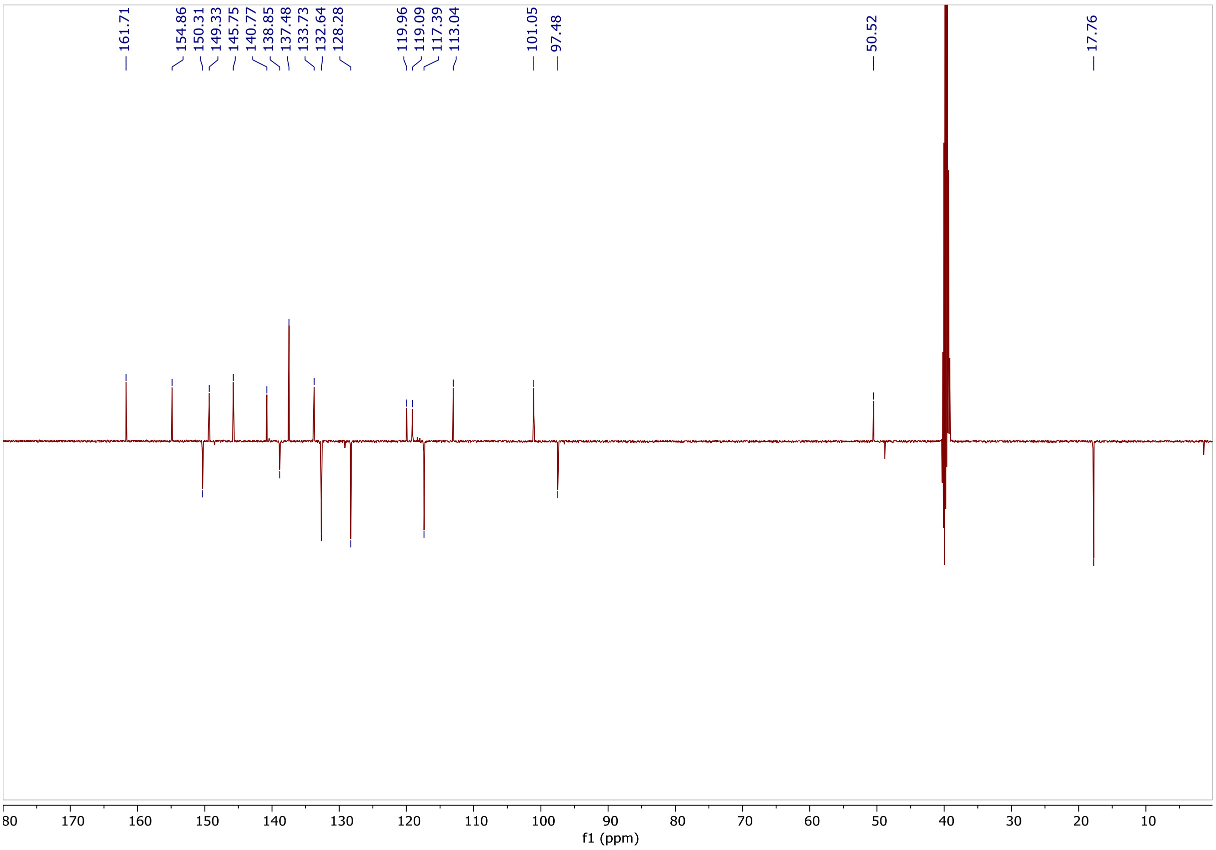

4-{2-[(4-Cyanophenyl)amino]-6-oxo-5*H*,6*H*,7*H*,8*H*,9*H*-pyrimido[4,5-b][1,4]diazepin-9-yl}-3,5-dimethylbenzonitrile (5):

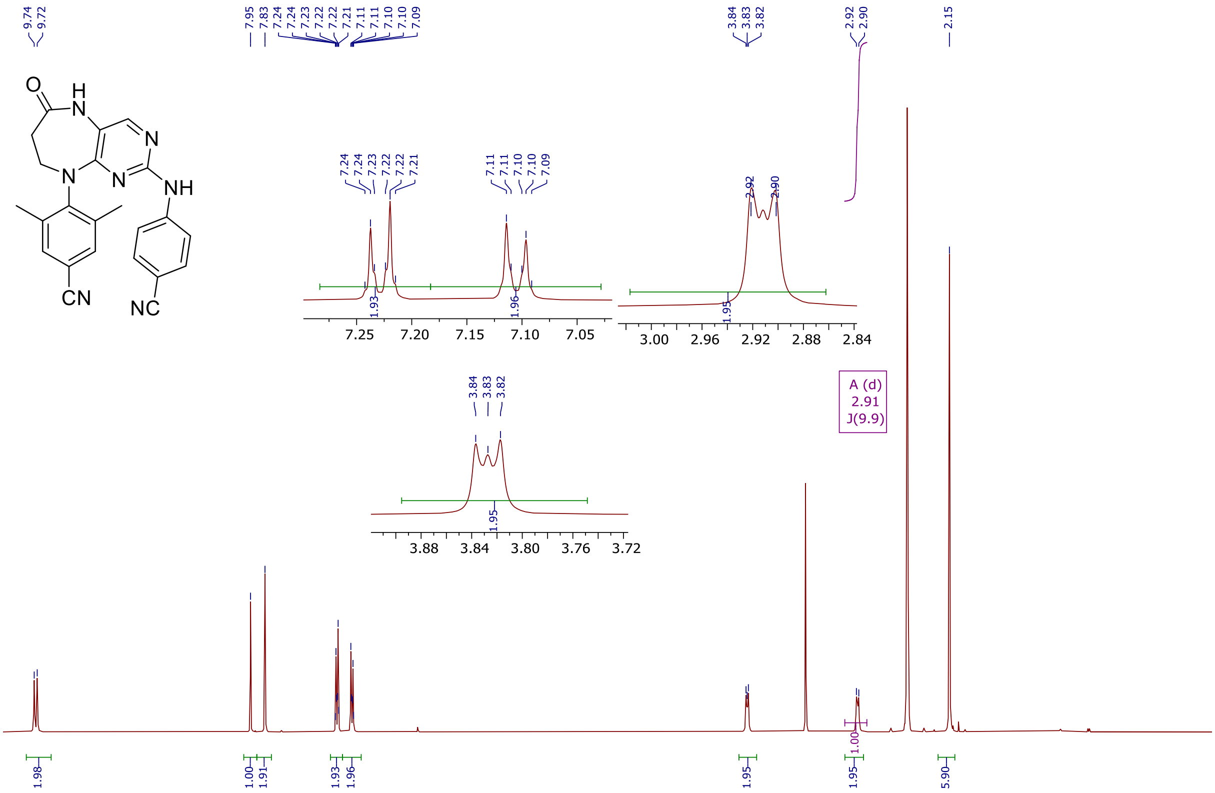

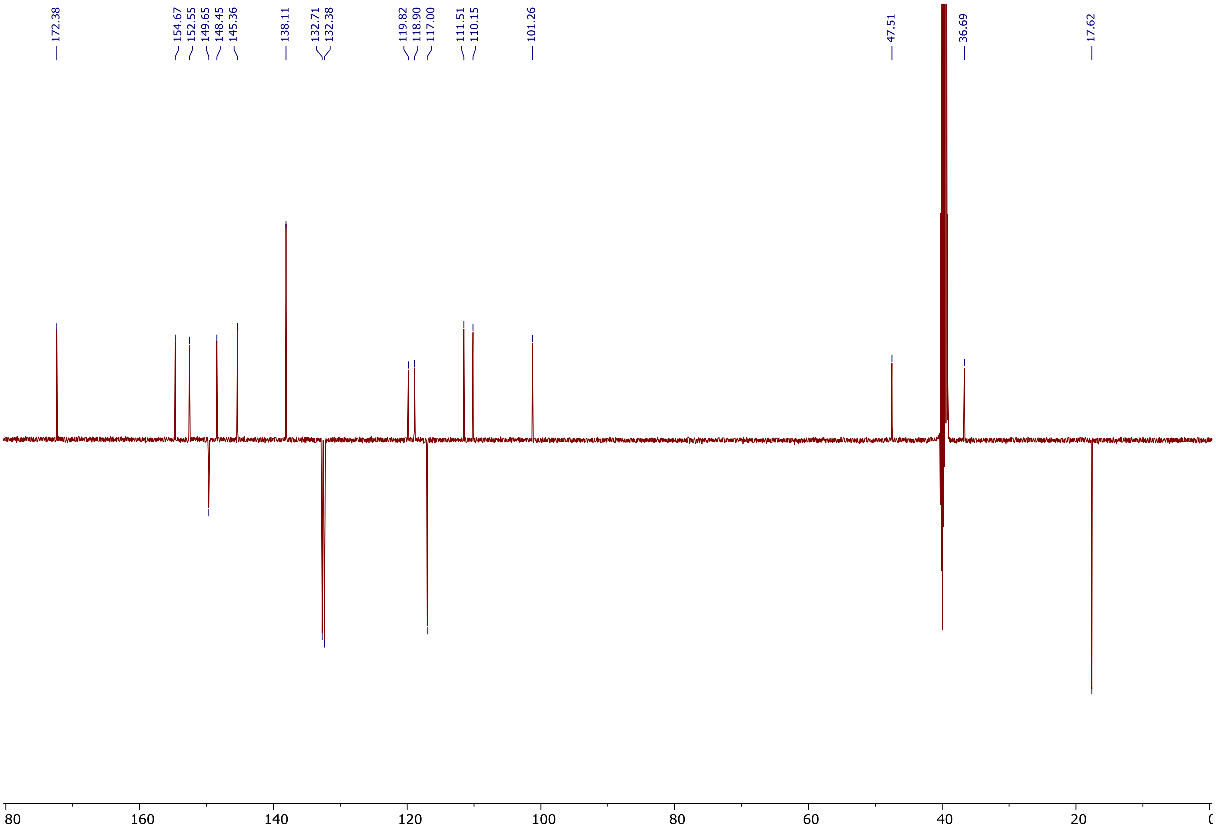

4-[(9-{4-[(1*E*)-2-Cyanoeth-1-en-1-yl]-2,6-dimethylphenyl}-6-oxo-5*H*,6*H*,7*H*,8*H*,9*H*-pyrimido[4,5-*b*][1,4]diazepin-2-yl)amino]benzonitrile (6):

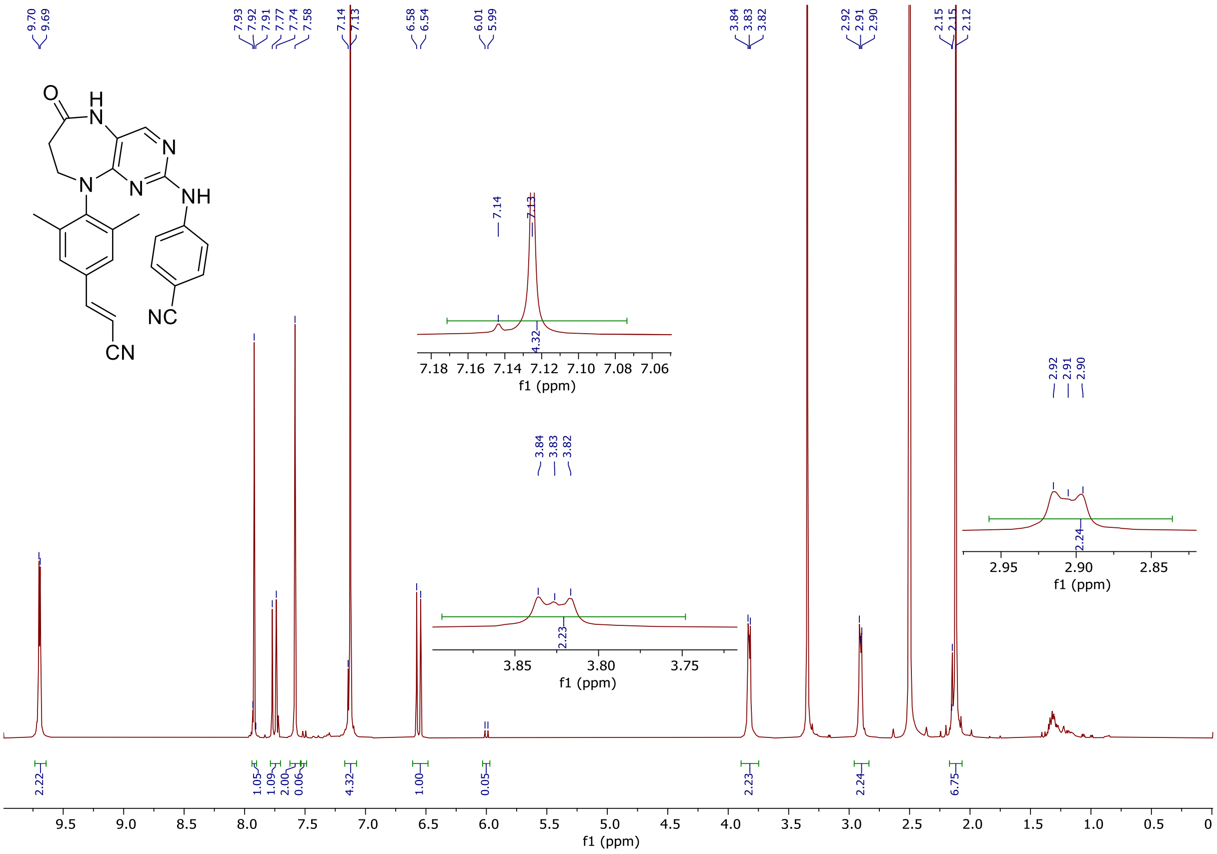

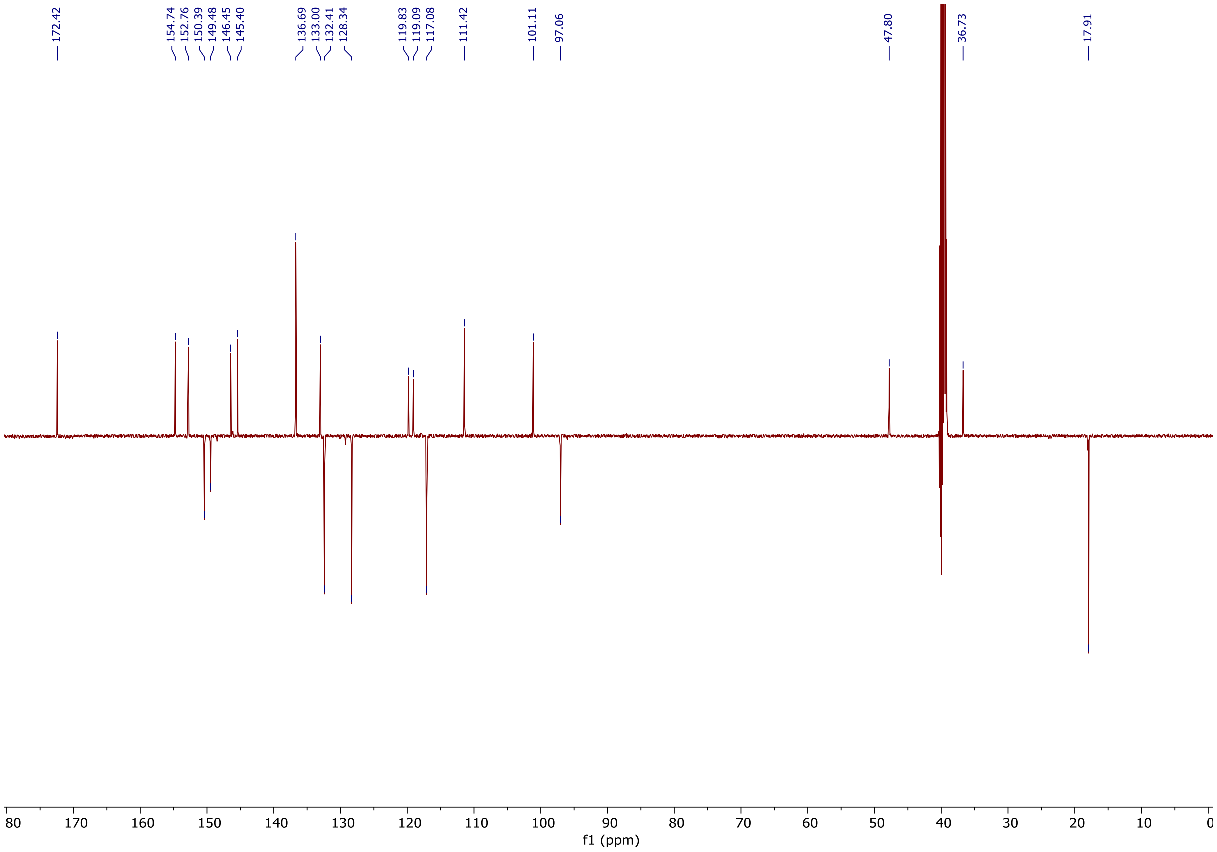

4-[(2-Chloro-5-nitropyrimidin-4-yl)amino]-3,5-dimethylbenzonitrile (9):

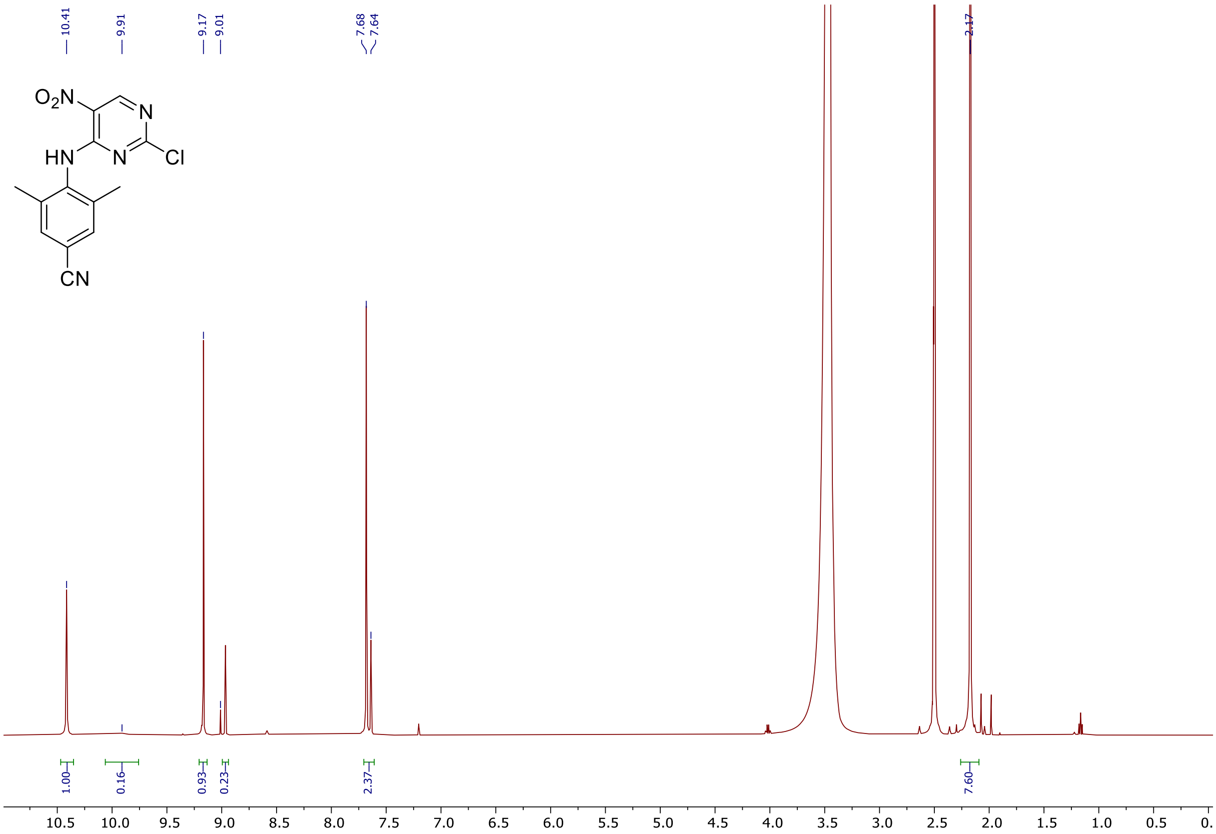

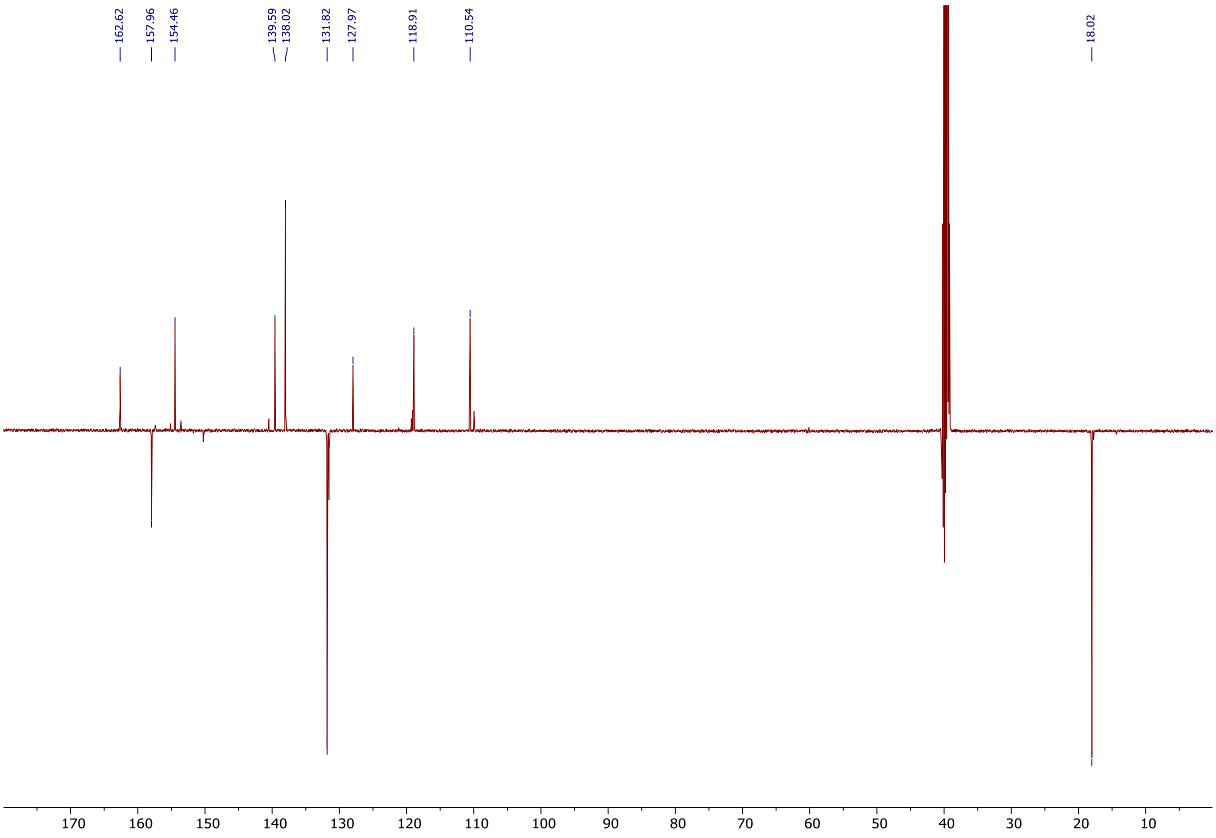

Ethyl *N*-(2-chloro-5-nitropyrimidin-4-yl)-*N*-(4-cyano-2,6-dimethylphenyl)glycinate (10):

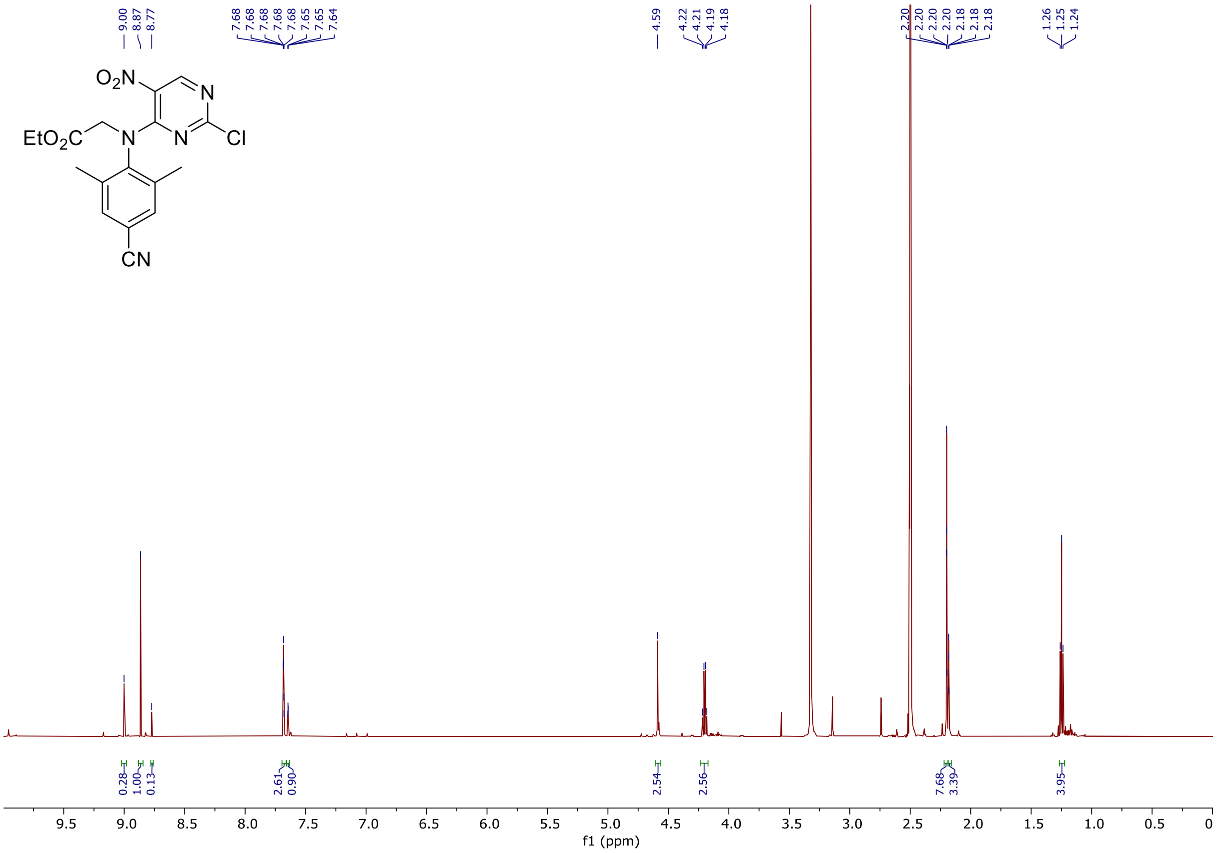

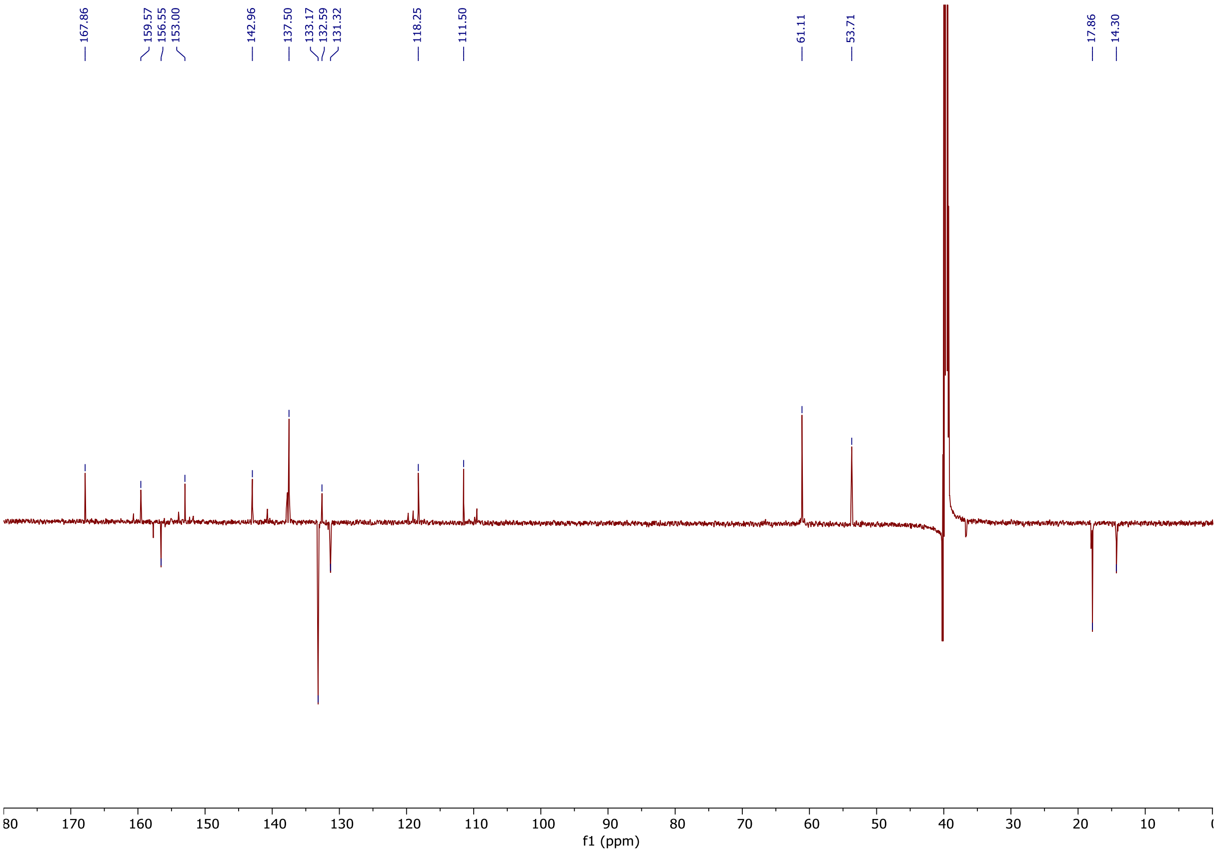

Ethyl *N*-(4-cyano-2,6-dimethylphenyl)-*N*-(2-((4-cyanophenyl)amino)-5-nitropyrimidin-4-yl)glycinate (11):

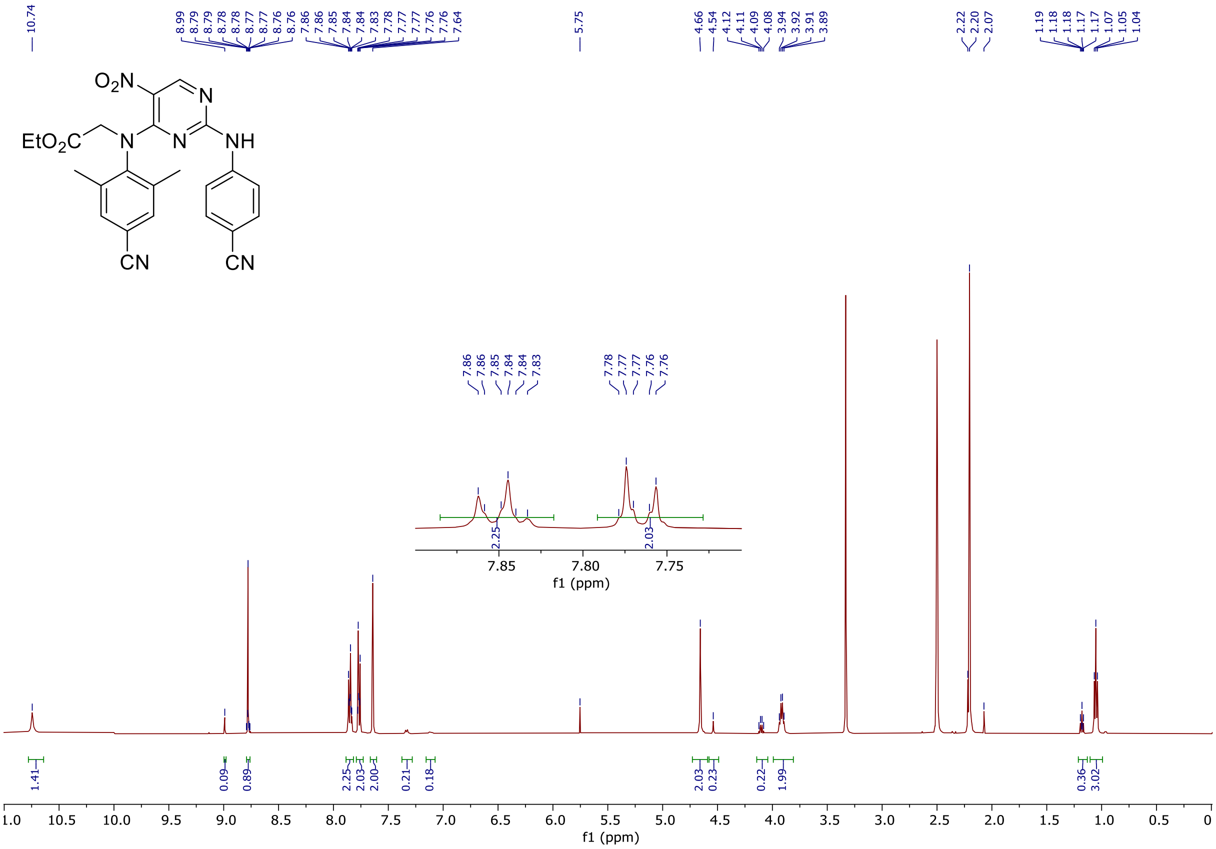

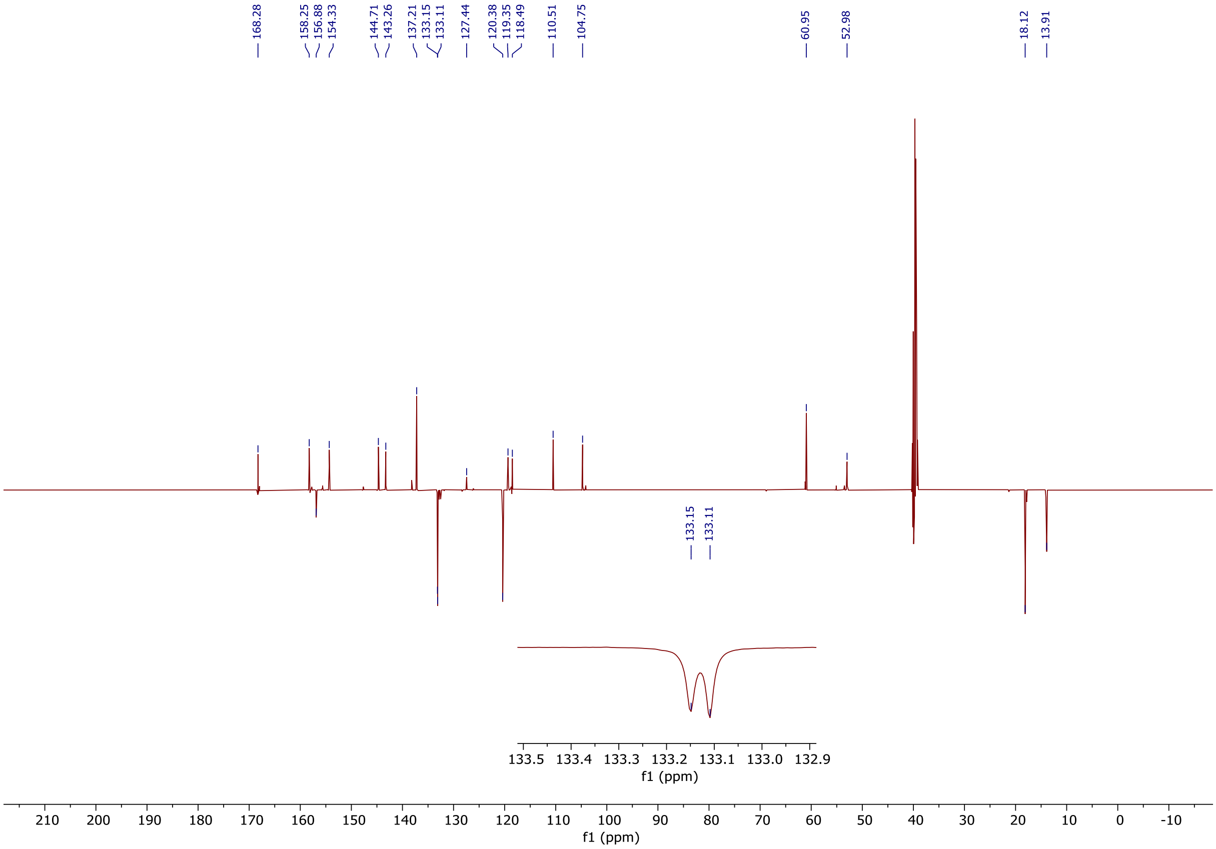

N-(4-Bromo-2,6-dimethylphenyl)-2-chloro-5-nitropyrimidin-4-amine (S1):

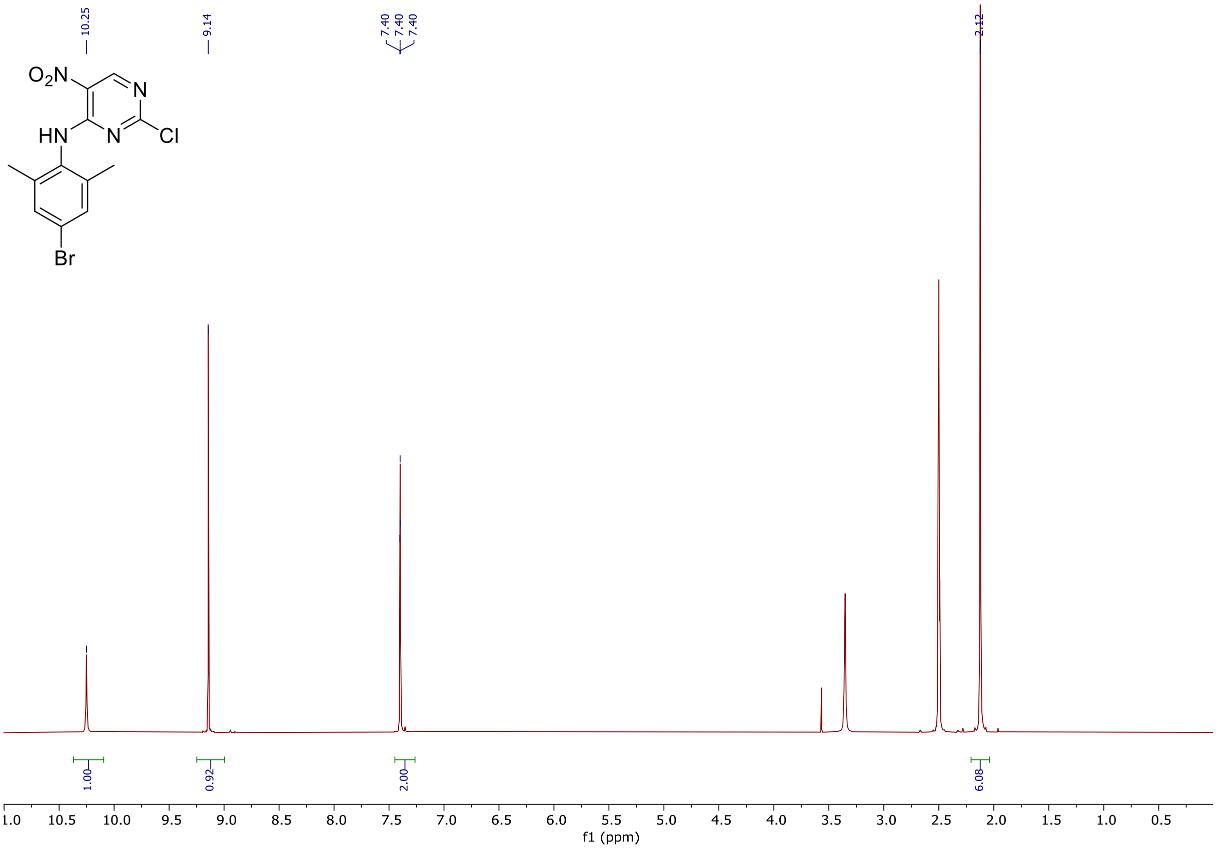

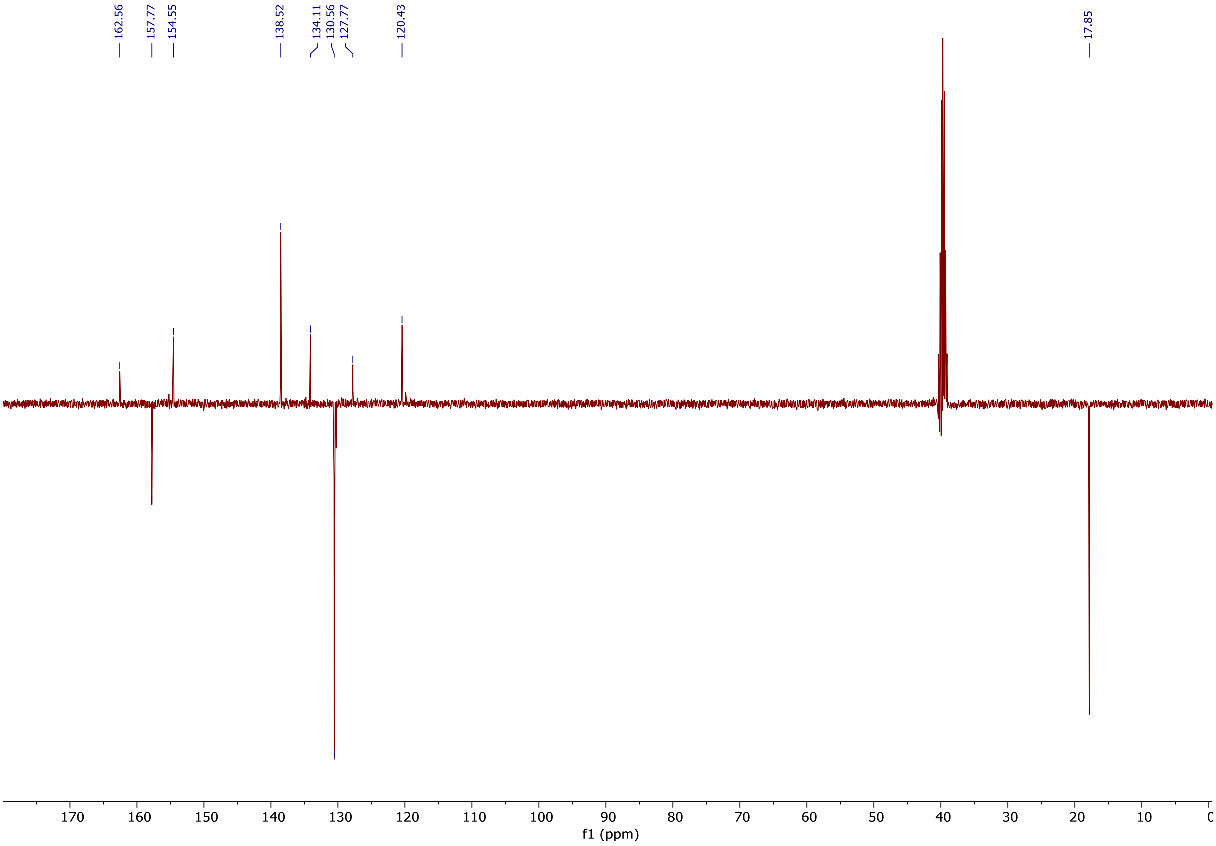

Ethyl 2-[(4-bromo-2,6-dimethylphenyl)(2-chloro-5-nitropyrimidin-4-yl)amino]acetate (13):

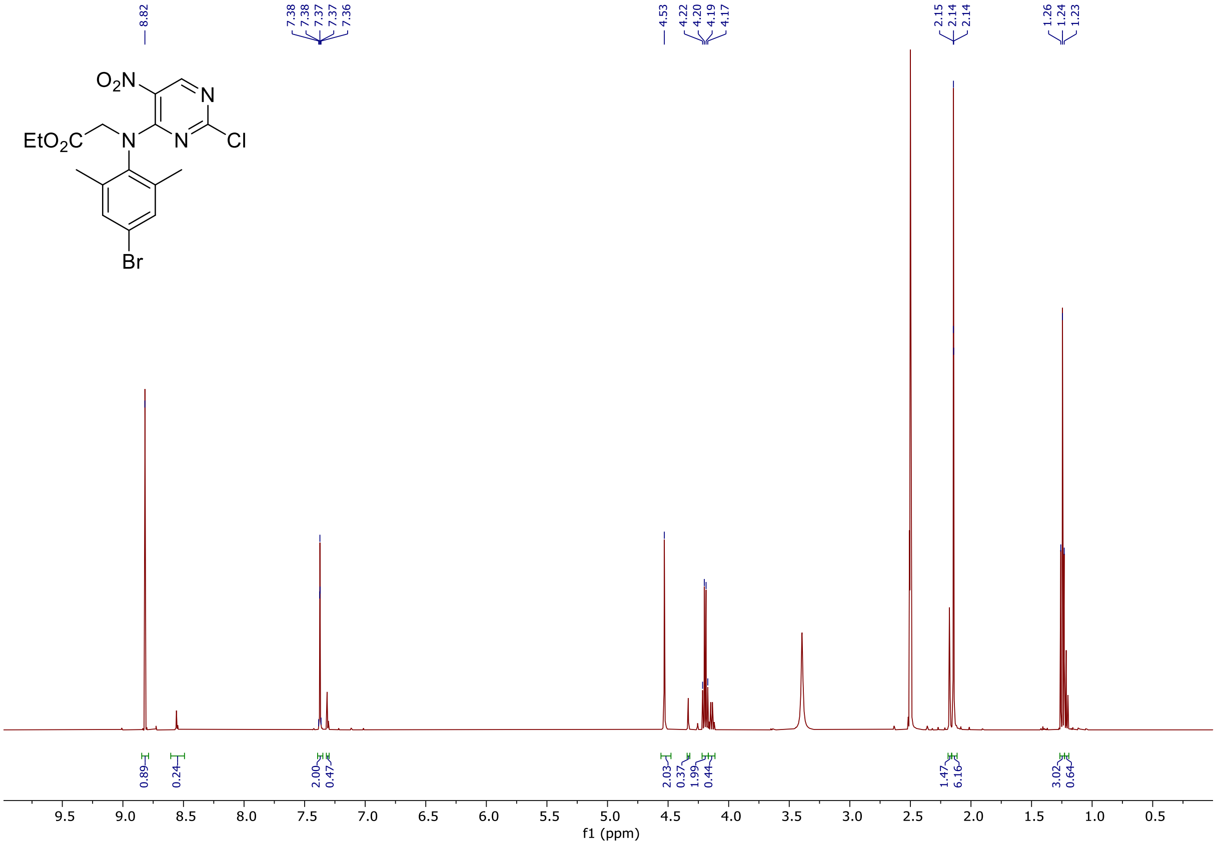

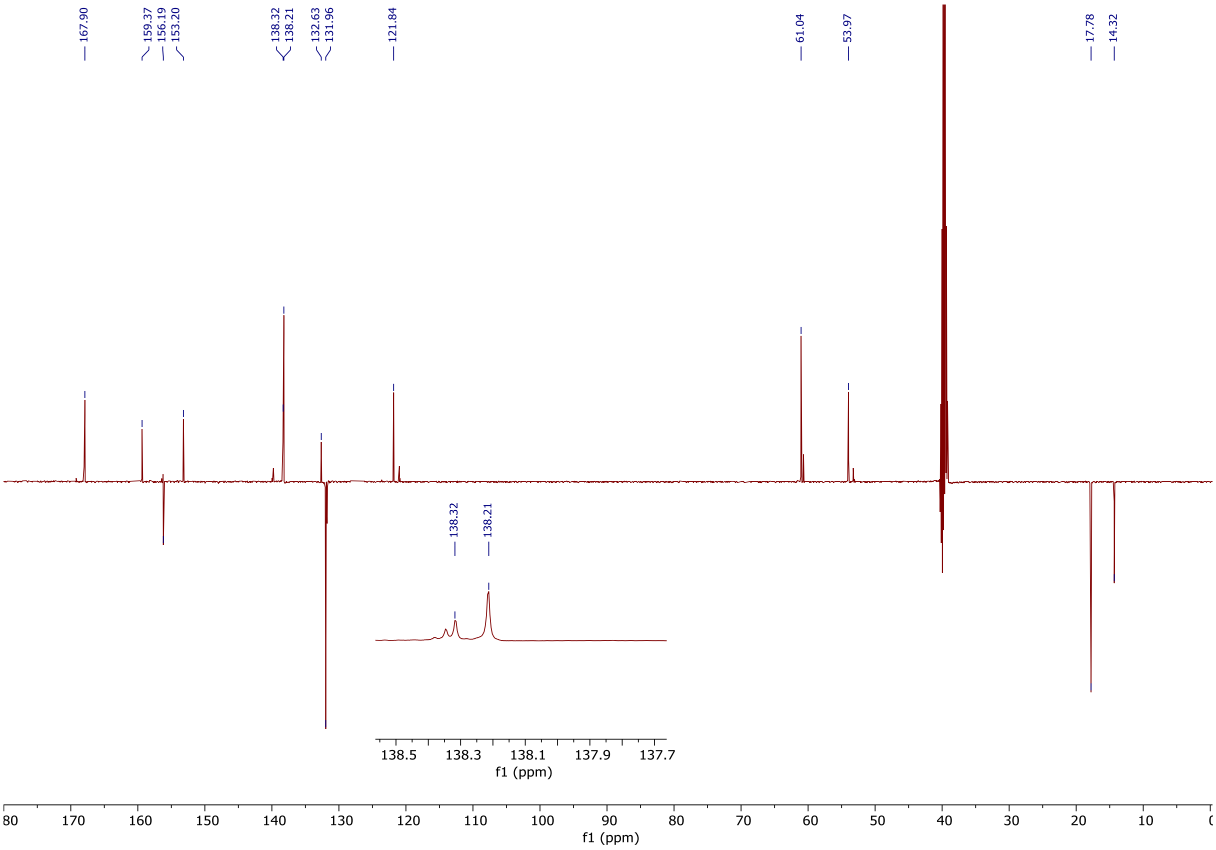

Ethyl 2-[(4-bromo-2,6-dimethylphenyl)({2-[(4-cyanophenyl)amino]-5-nitropyrimidin-4-yl})amino]acetate (S2):

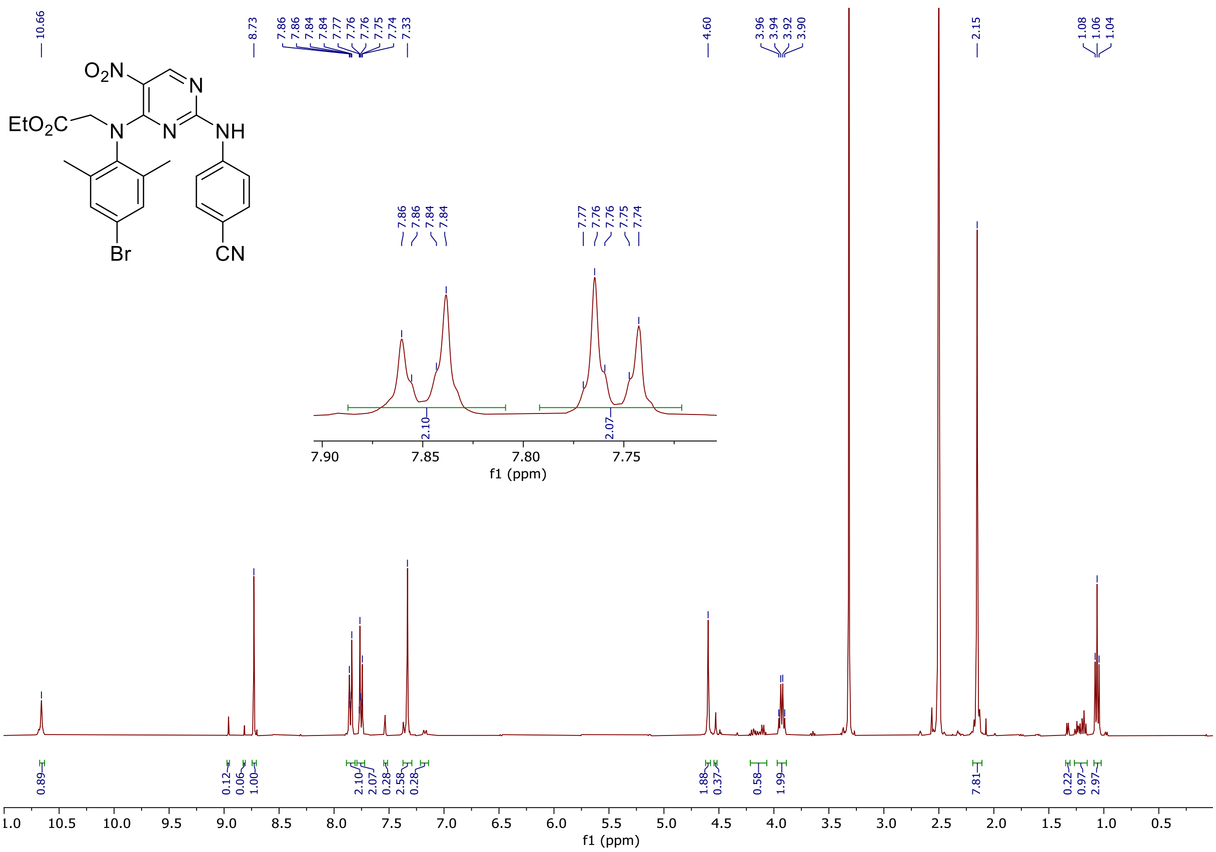

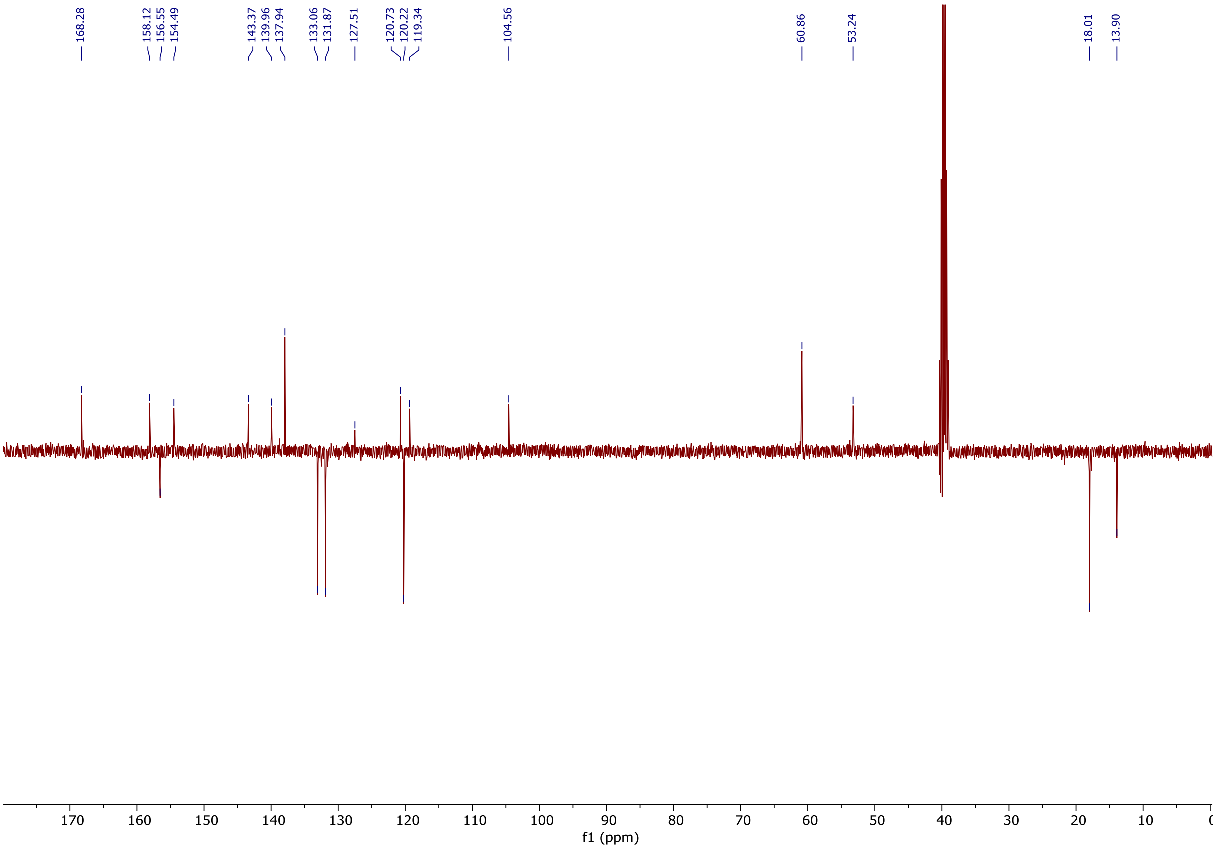

4-{[8-(4-Bromo-2,6-dimethylphenyl)-6-oxo-5,6,7,8-tetrahydropteridin-2-yl]amino}benzonitrile (14):

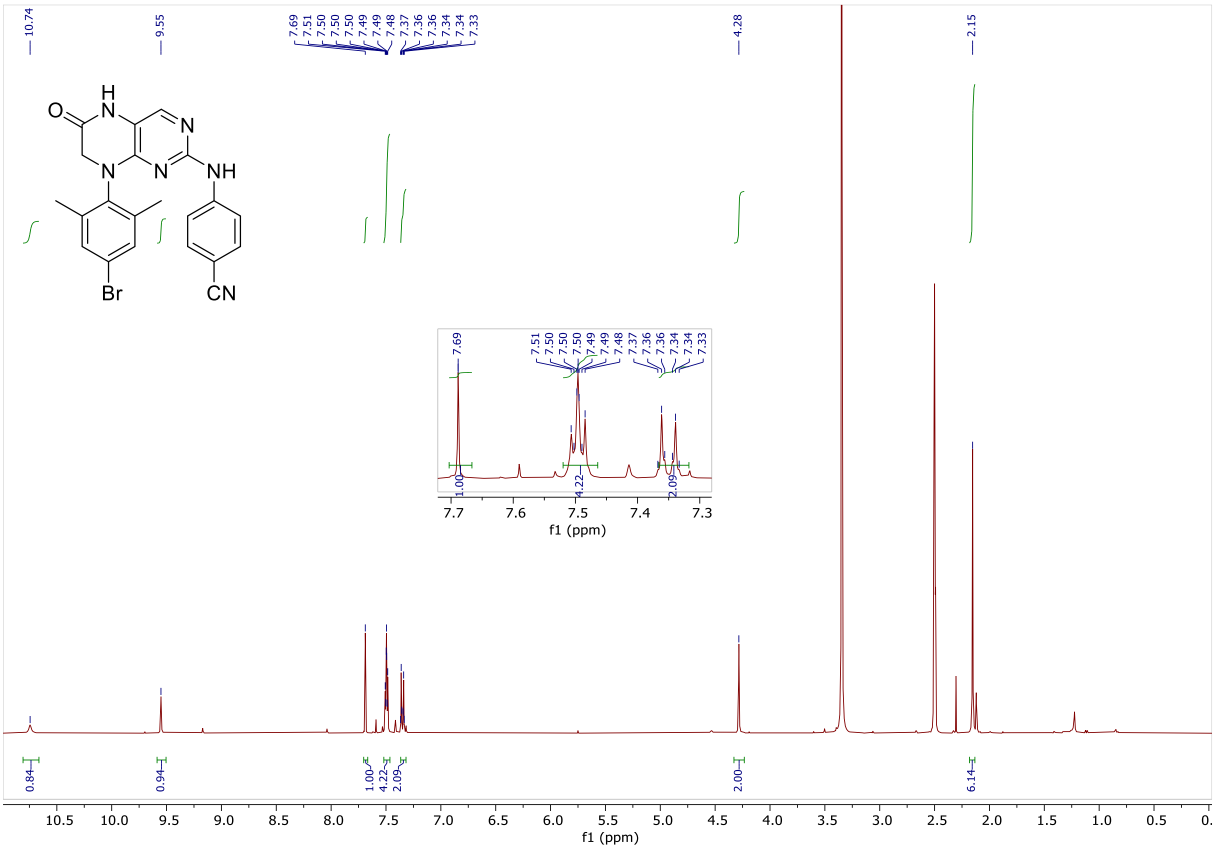

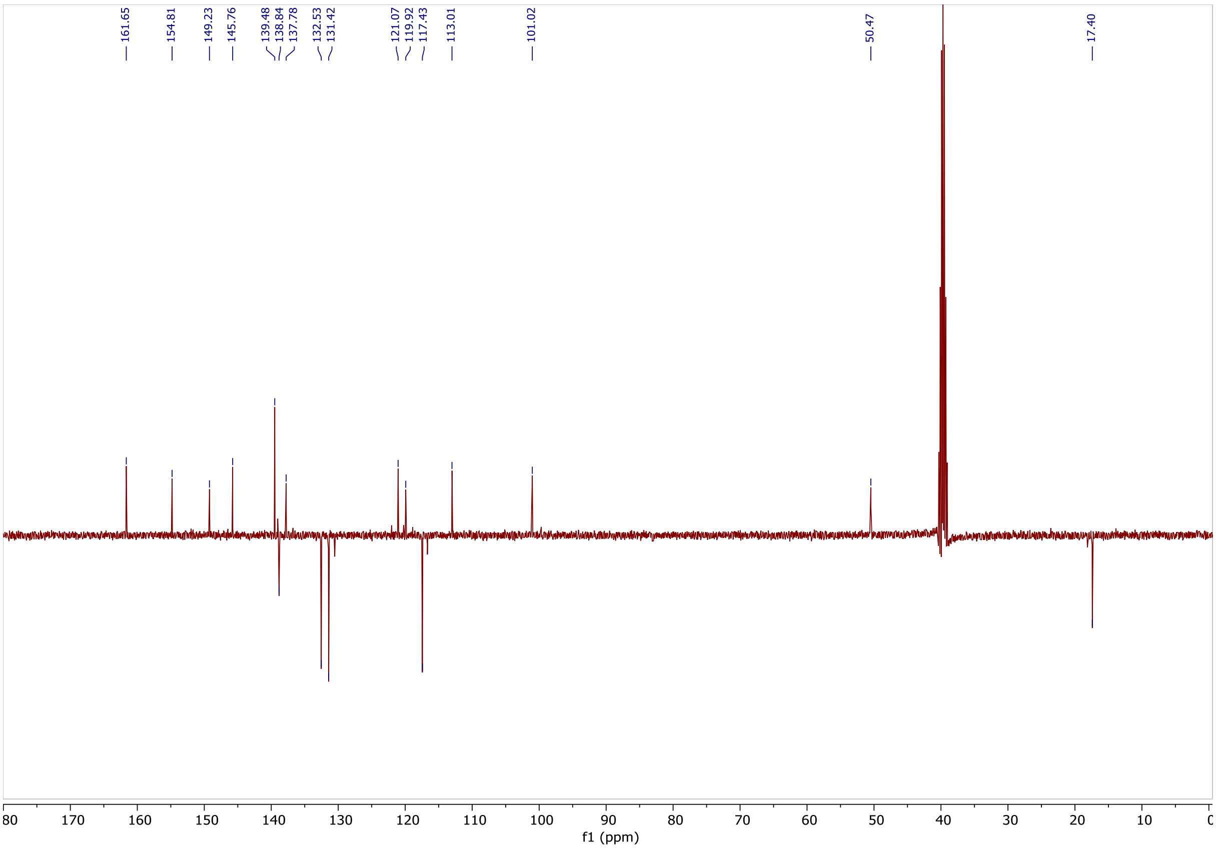

4-({2-[(4-Cyanophenyl)amino]-5-nitropyrimidin-4-yl}amino)-3,5-dimethylbenzonitrile (15):

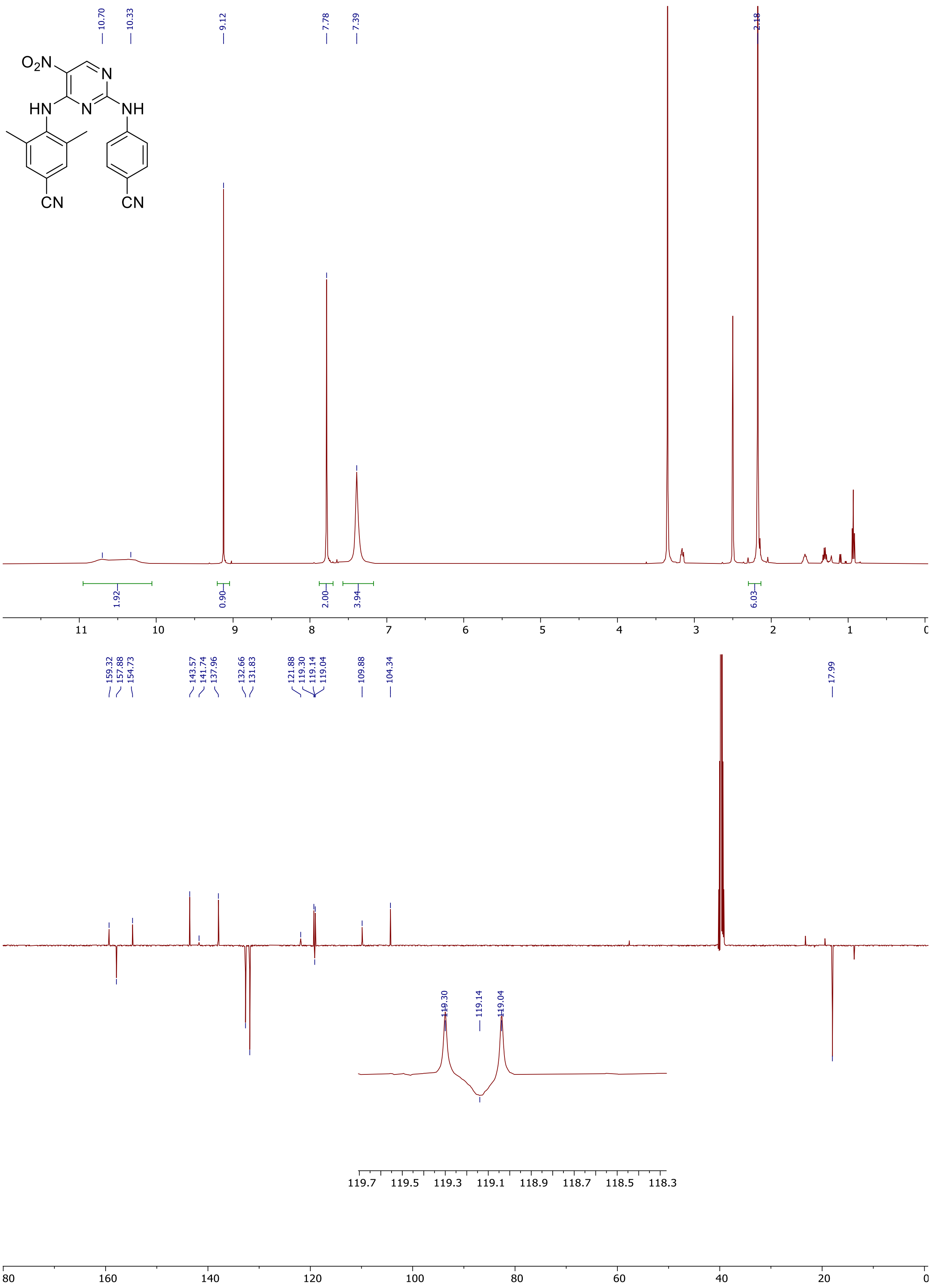

4-({5-Amino-2-[(4-cyanophenyl)amino]pyrimidin-4-yl}amino)-3,5-dimethylbenzonitrile (16):

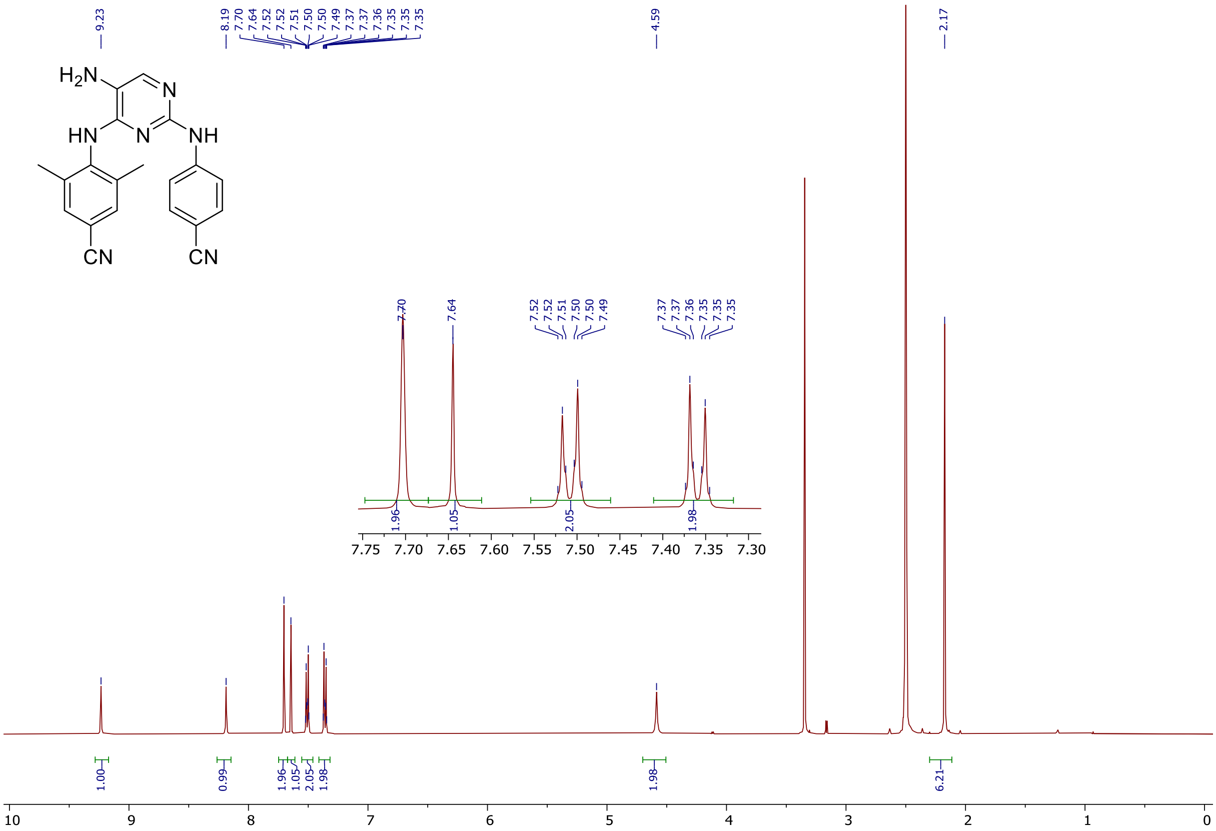

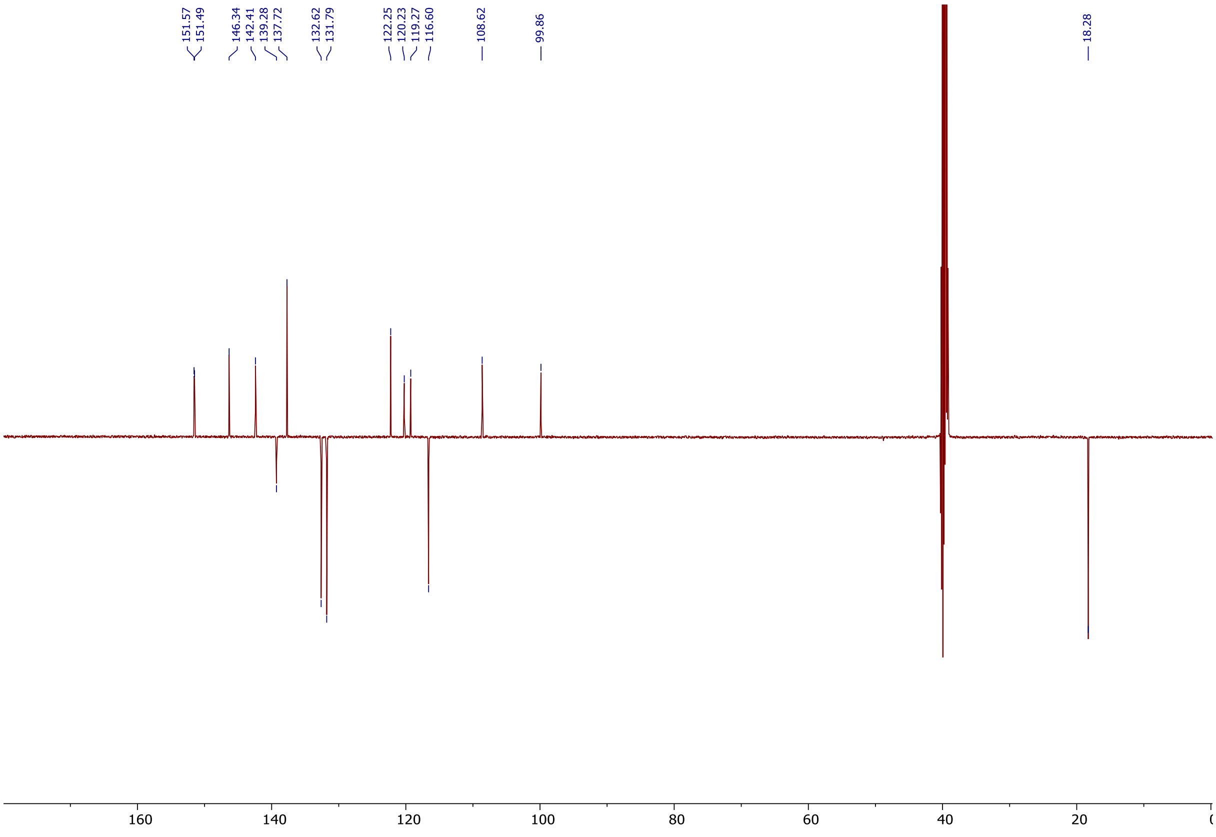

Ethyl 3-[(4-bromo-2,6-dimethylphenyl)amino]propanoate (17):

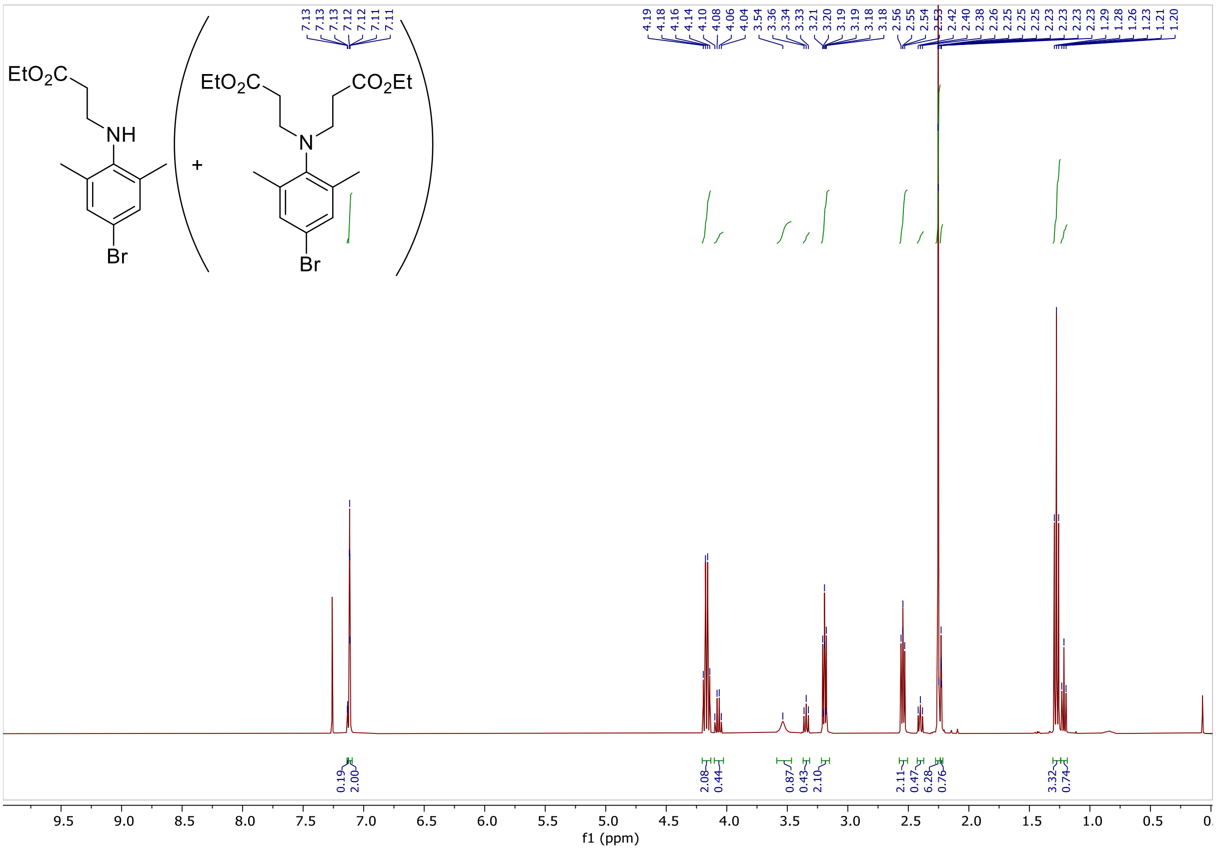

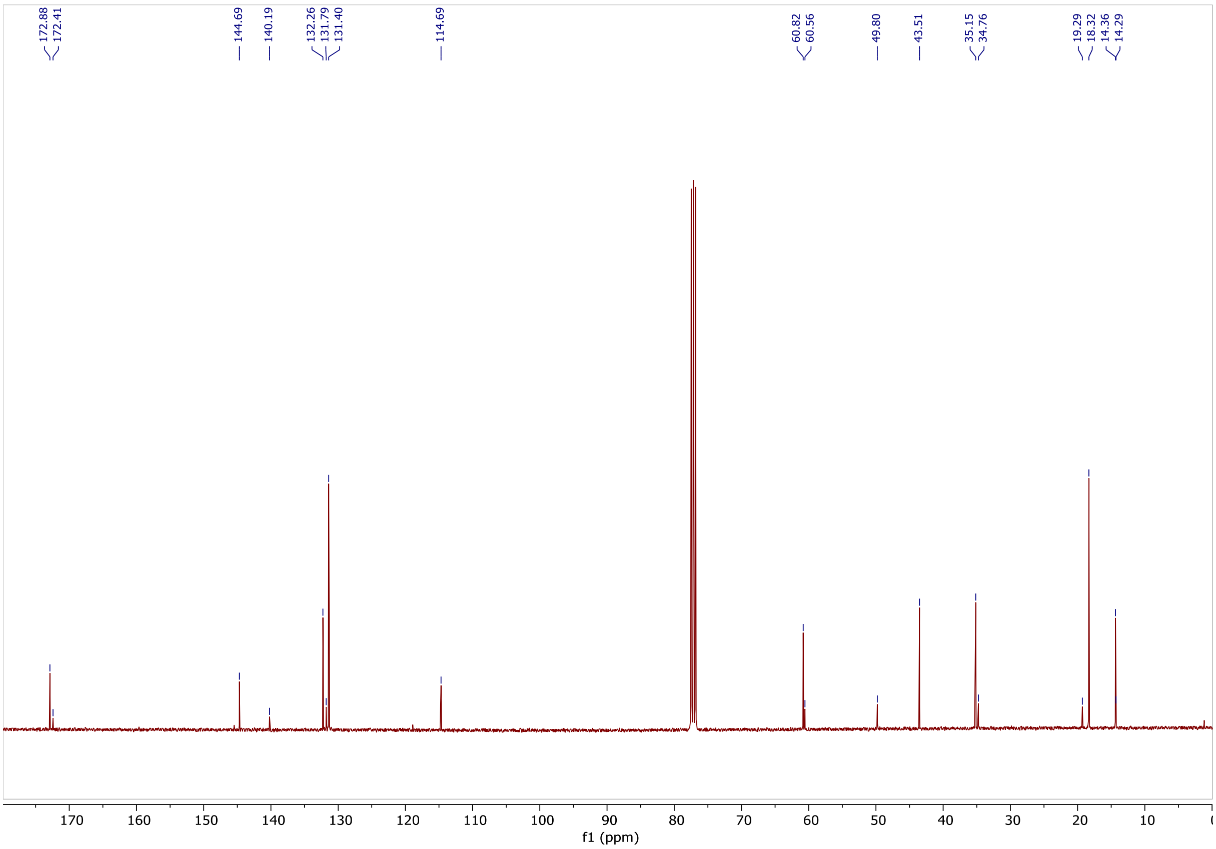

Ethyl 3-[(4-bromo-2,6-dimethylphenyl)(2-chloro-5-nitropyrimidin-4-yl)amino]propanoate (18):

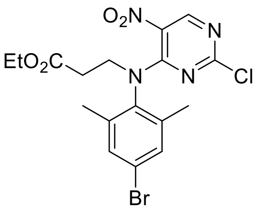

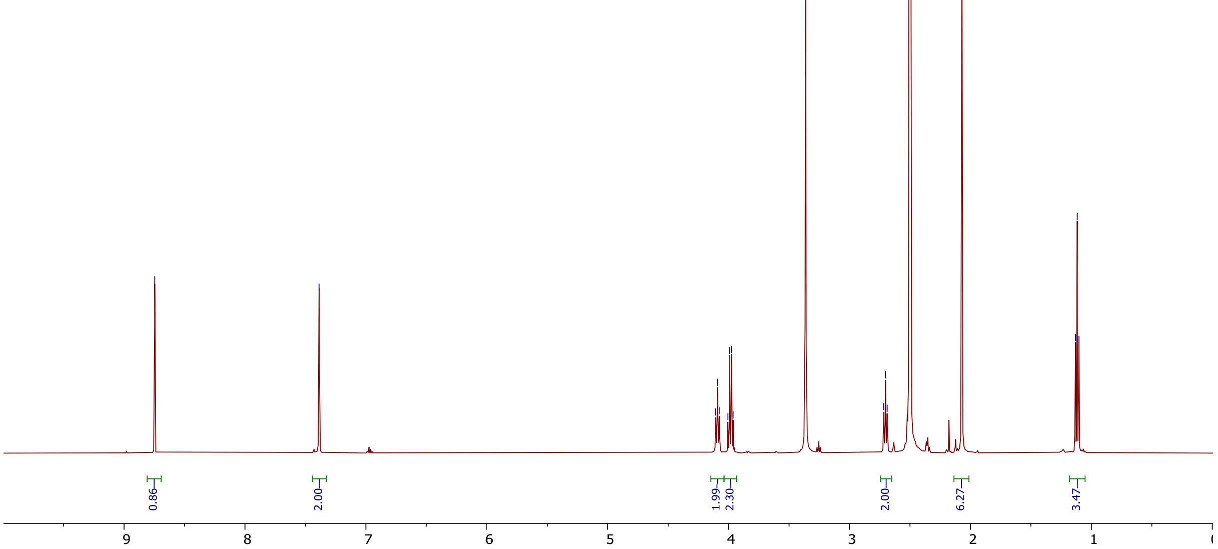

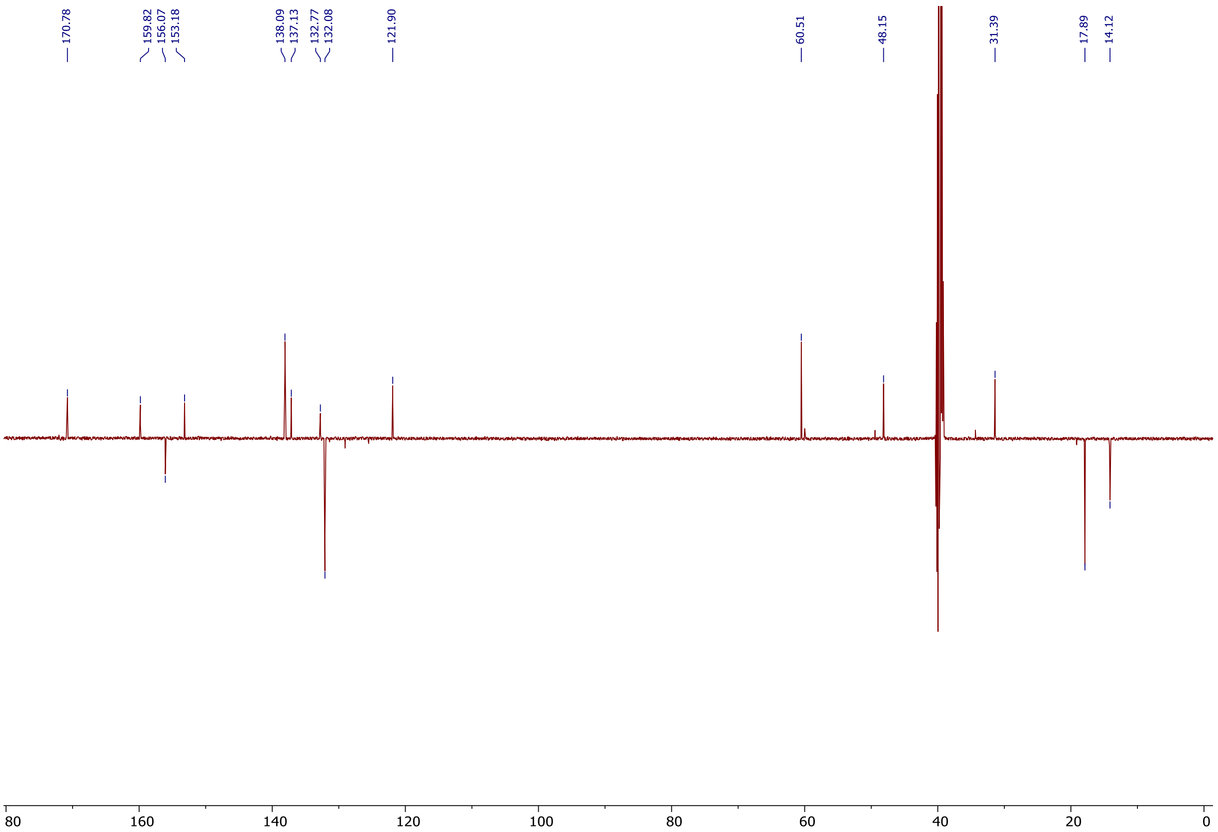

Ethyl 3-[(4-bromo-2,6-dimethylphenyl)({2-[(4-cyanophenyl)amino]-5-nitropyrimidin-4-yl})amino]propanoate (19):

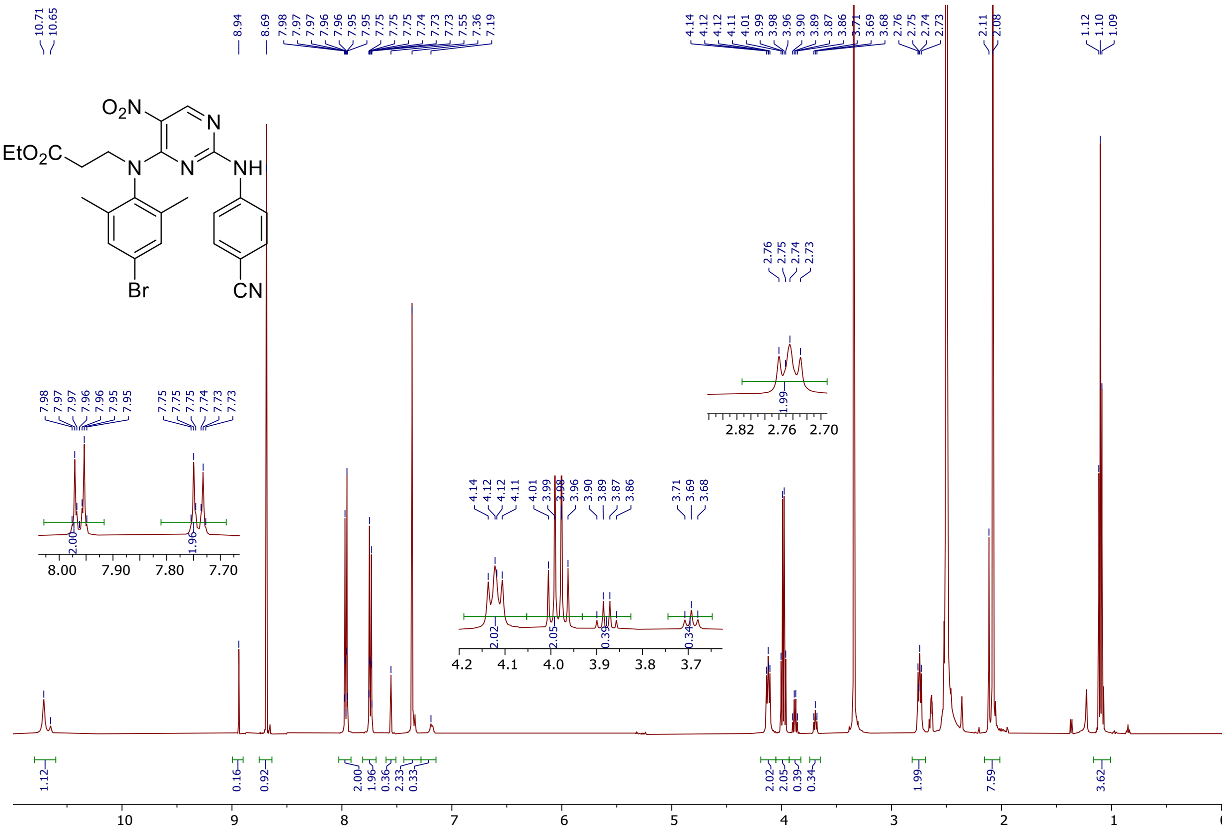

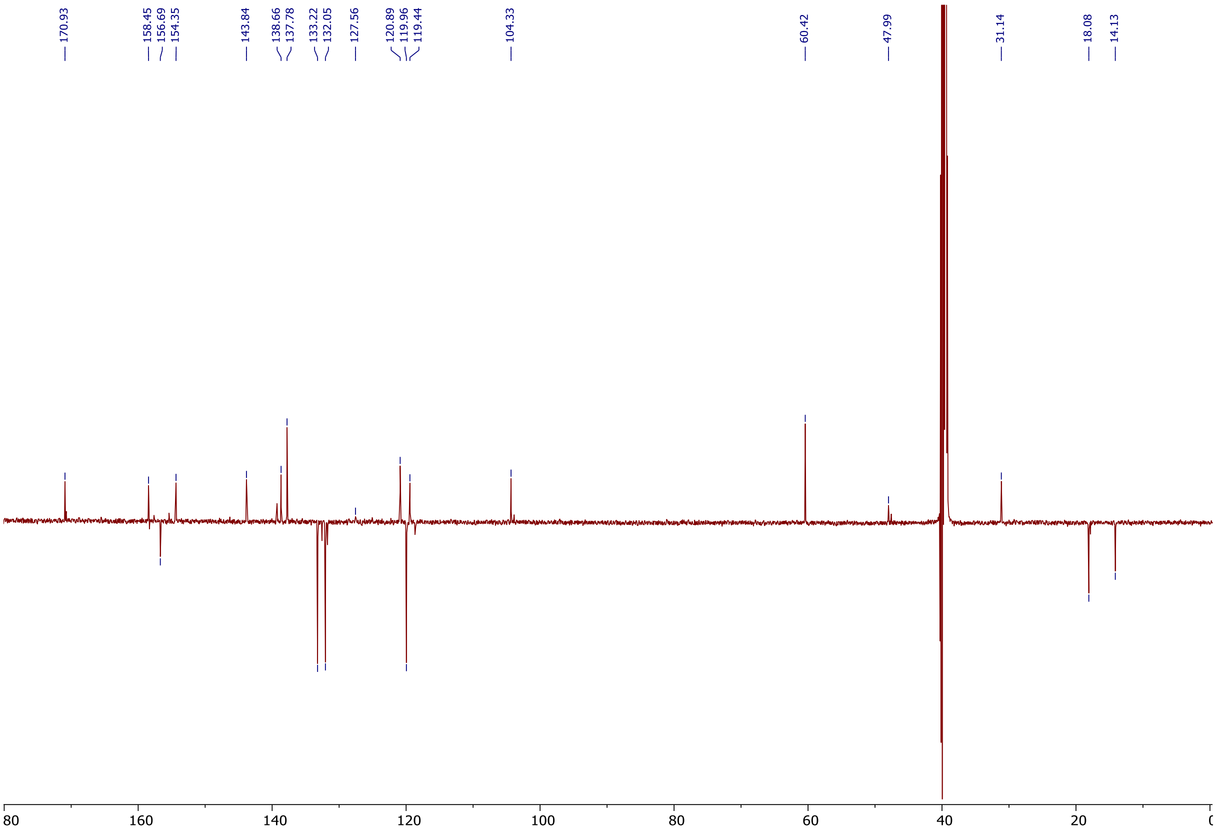

N'-(4-bromo-2,6-dimethylphenyl)-*N*,*N*-dimethylmethanimidamide (21):

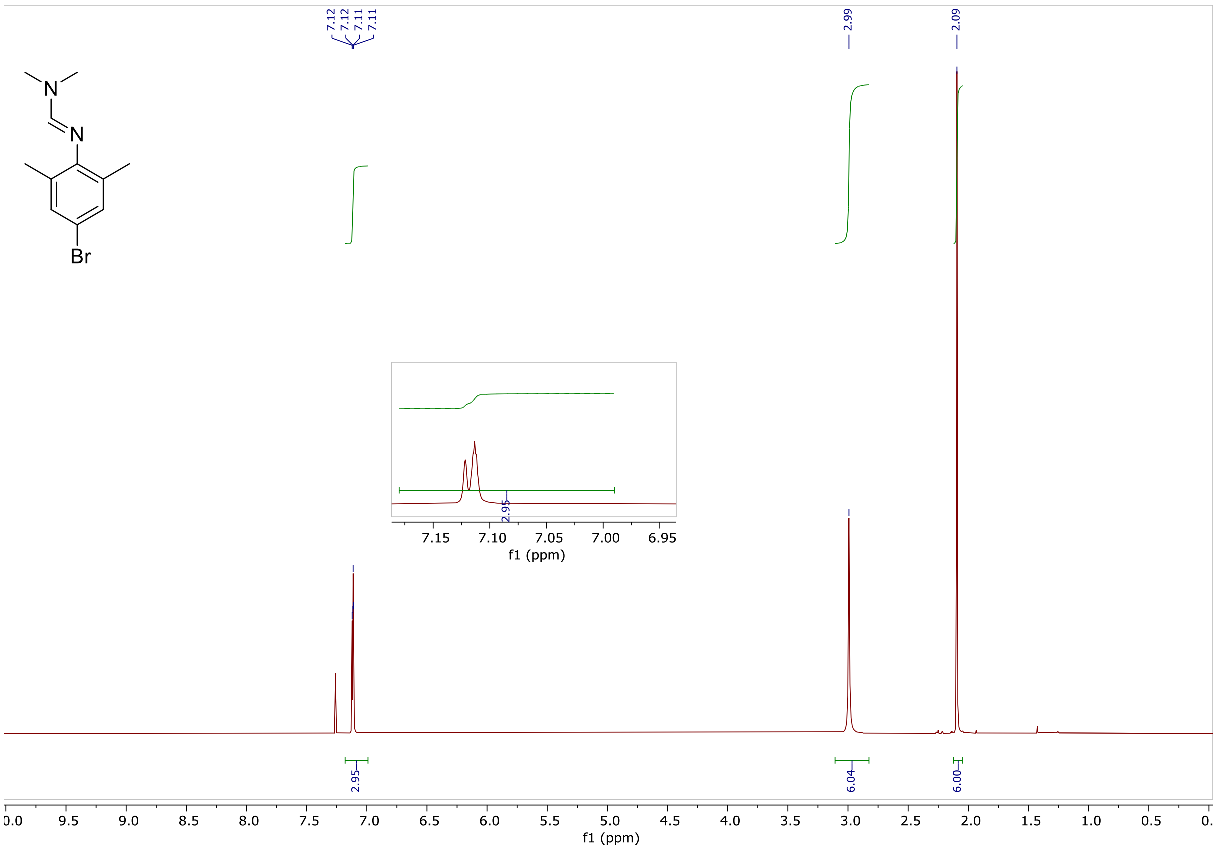

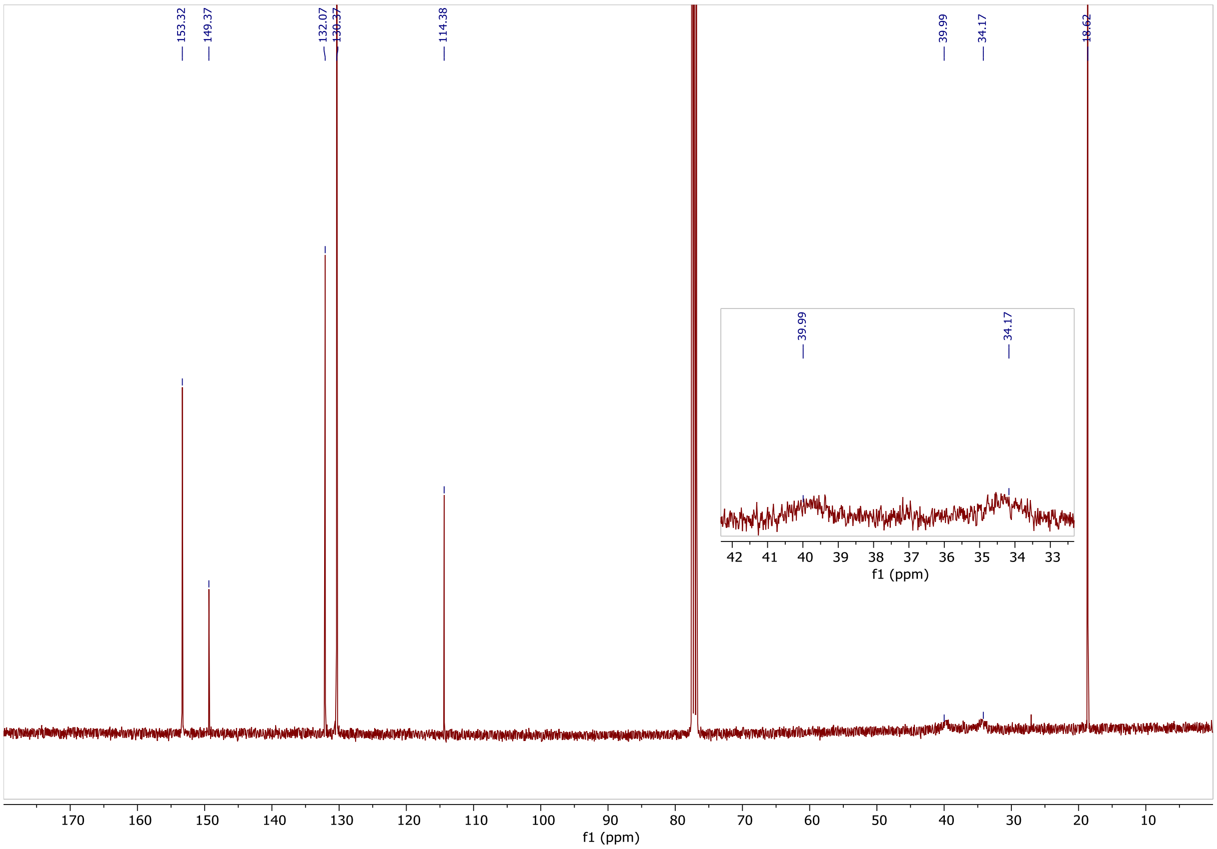

N'-(4-formyl-2,6-dimethylphenyl)-*N*,*N*-dimethylmethanimidamide (22):

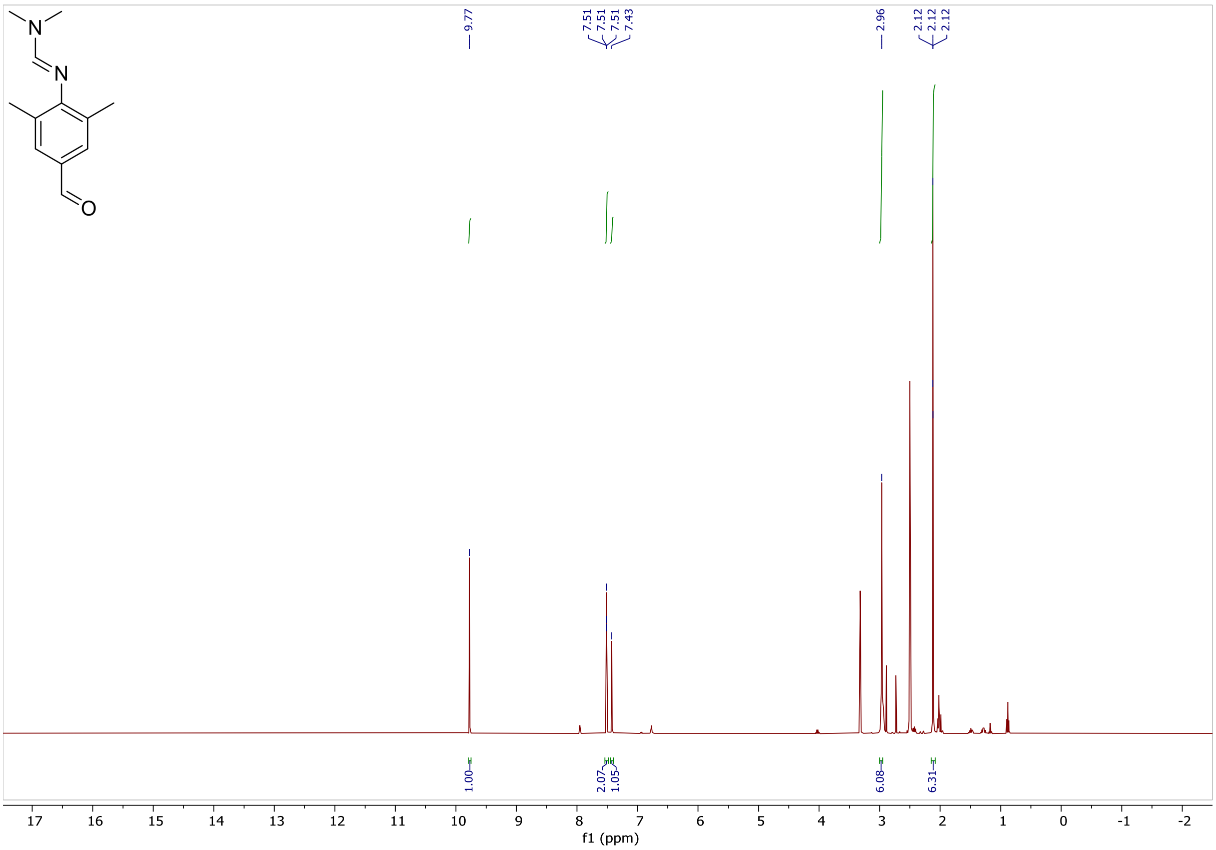

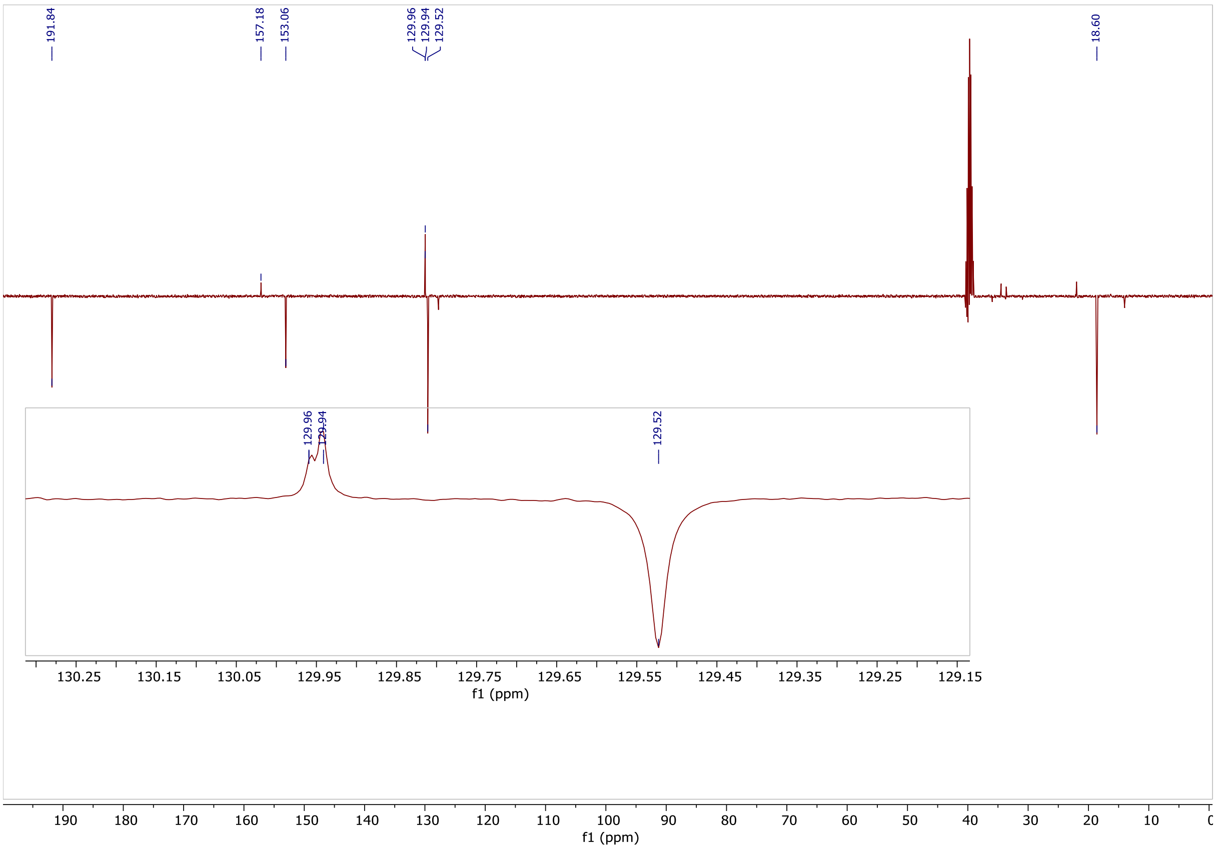

N'-[4-(hydroxymethyl)-2,6-dimethylphenyl]-*N*,*N*-dimethylmethanimidamide (23):

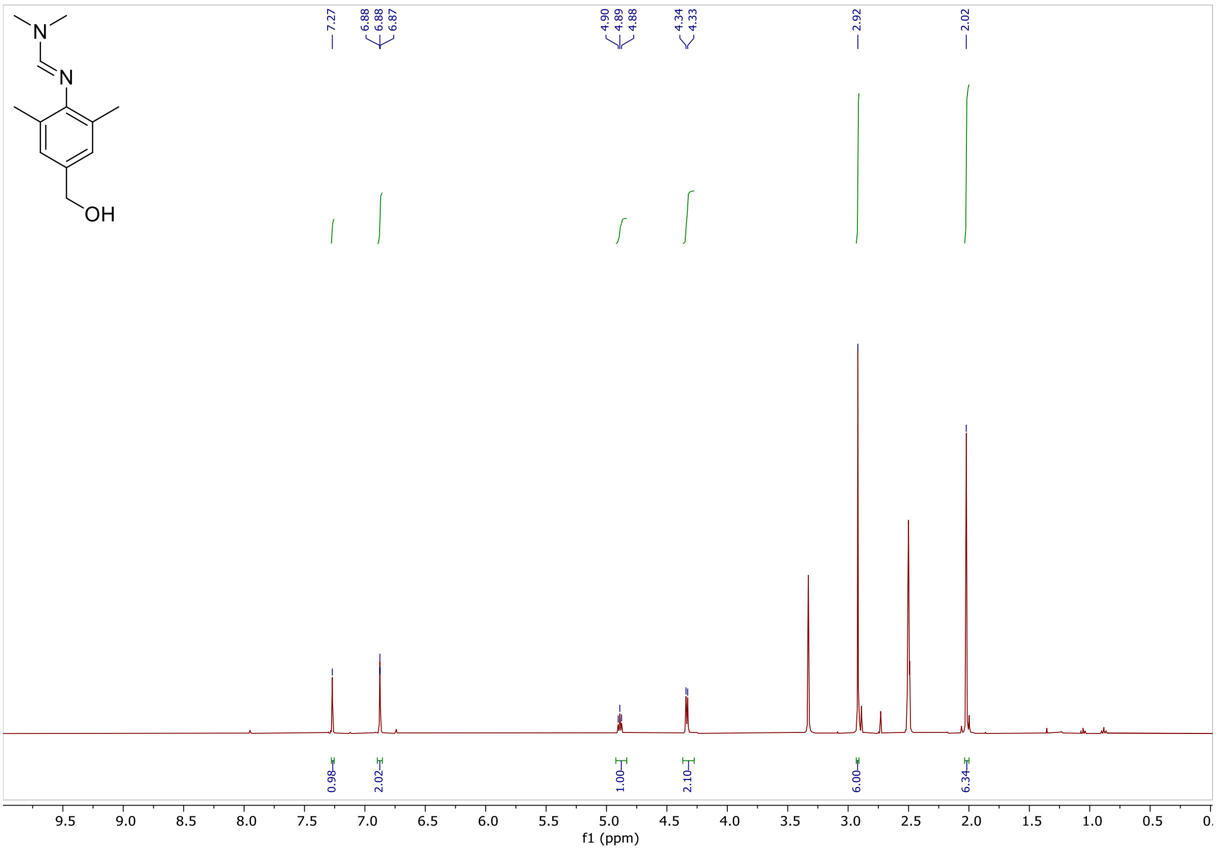

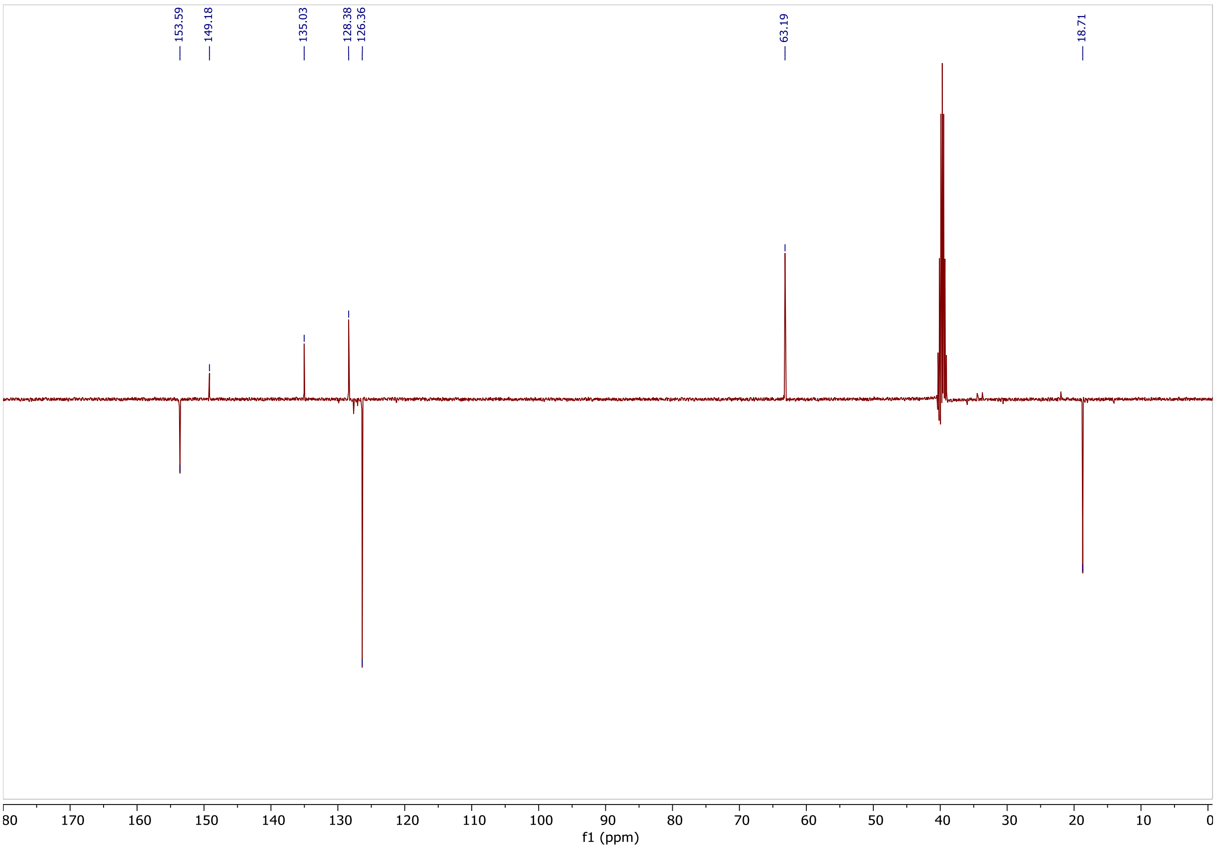

(4-Amino-3,5-dimethylphenyl)methanol (24):

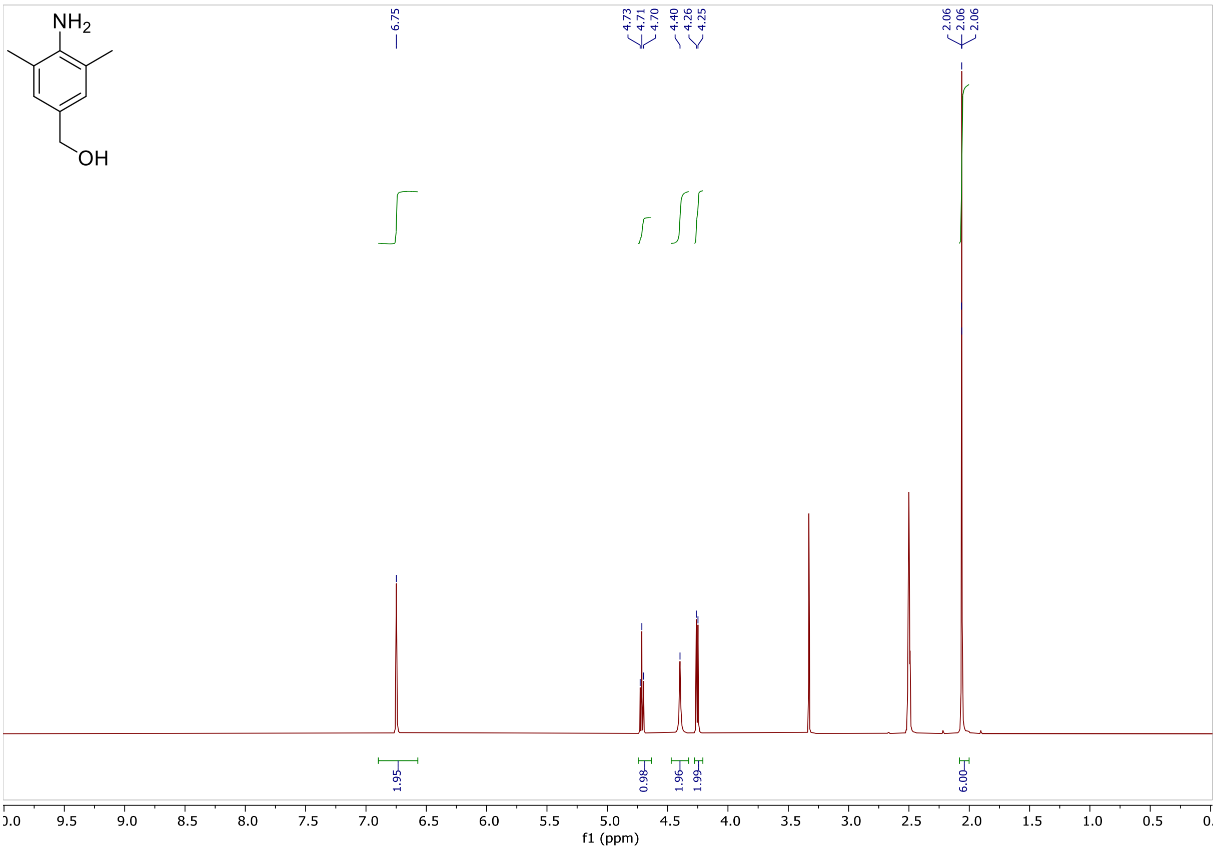

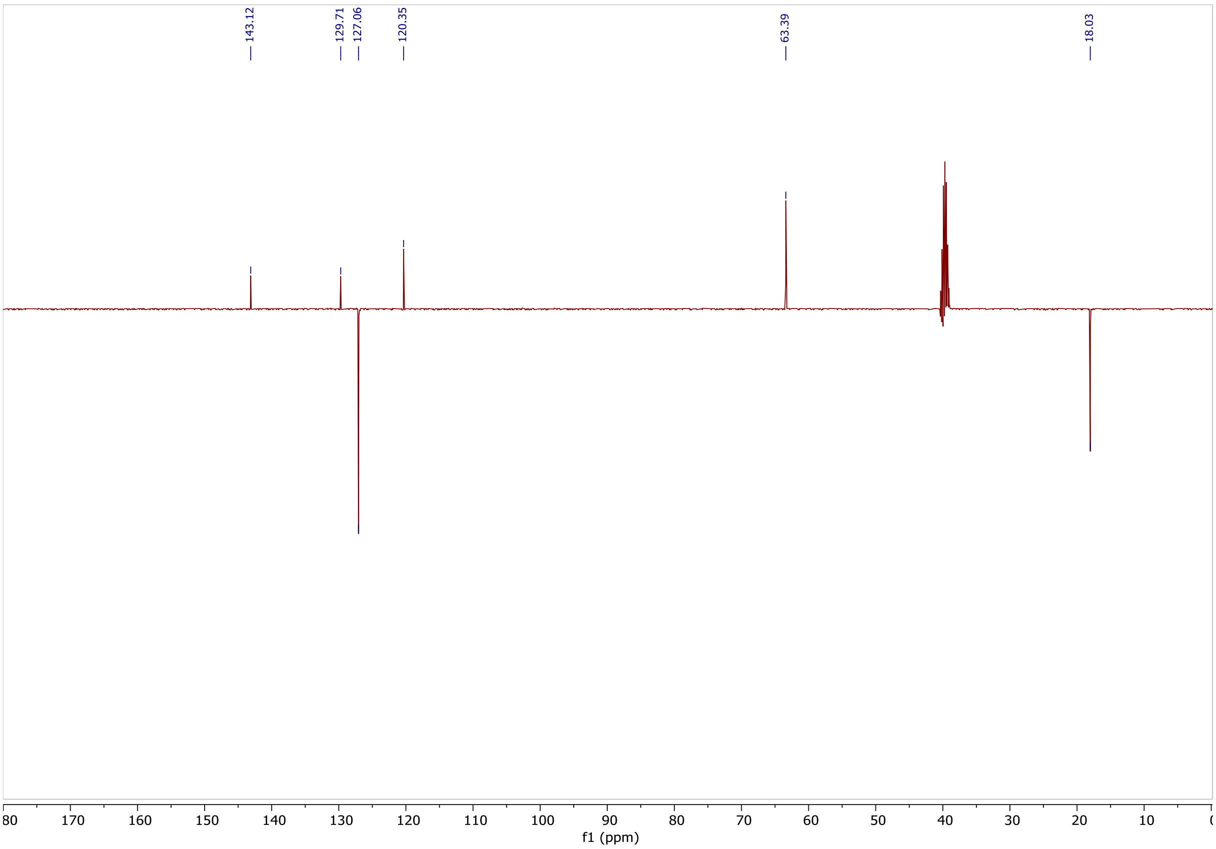

2,6-Dimethyl-4-({[tris(propan-2-yl)silyl]oxy}methyl)aniline (20):

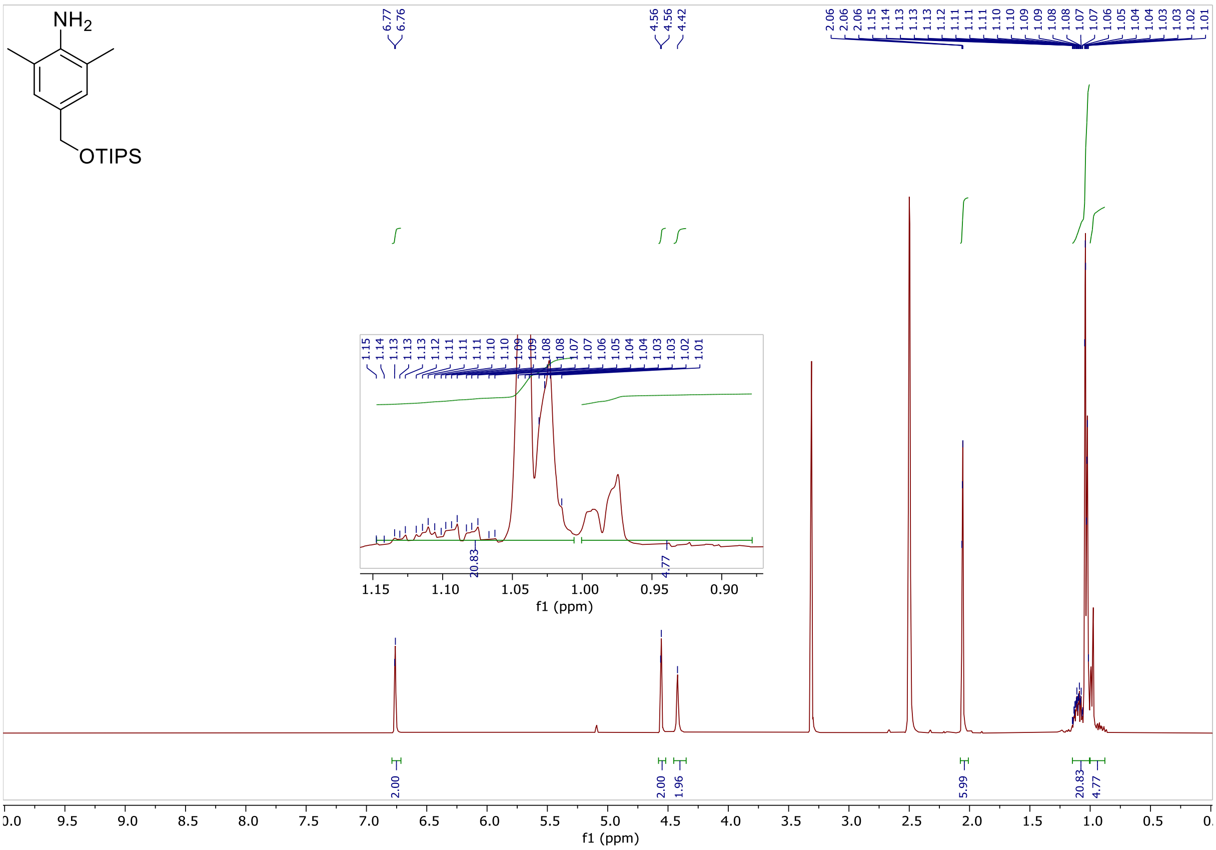

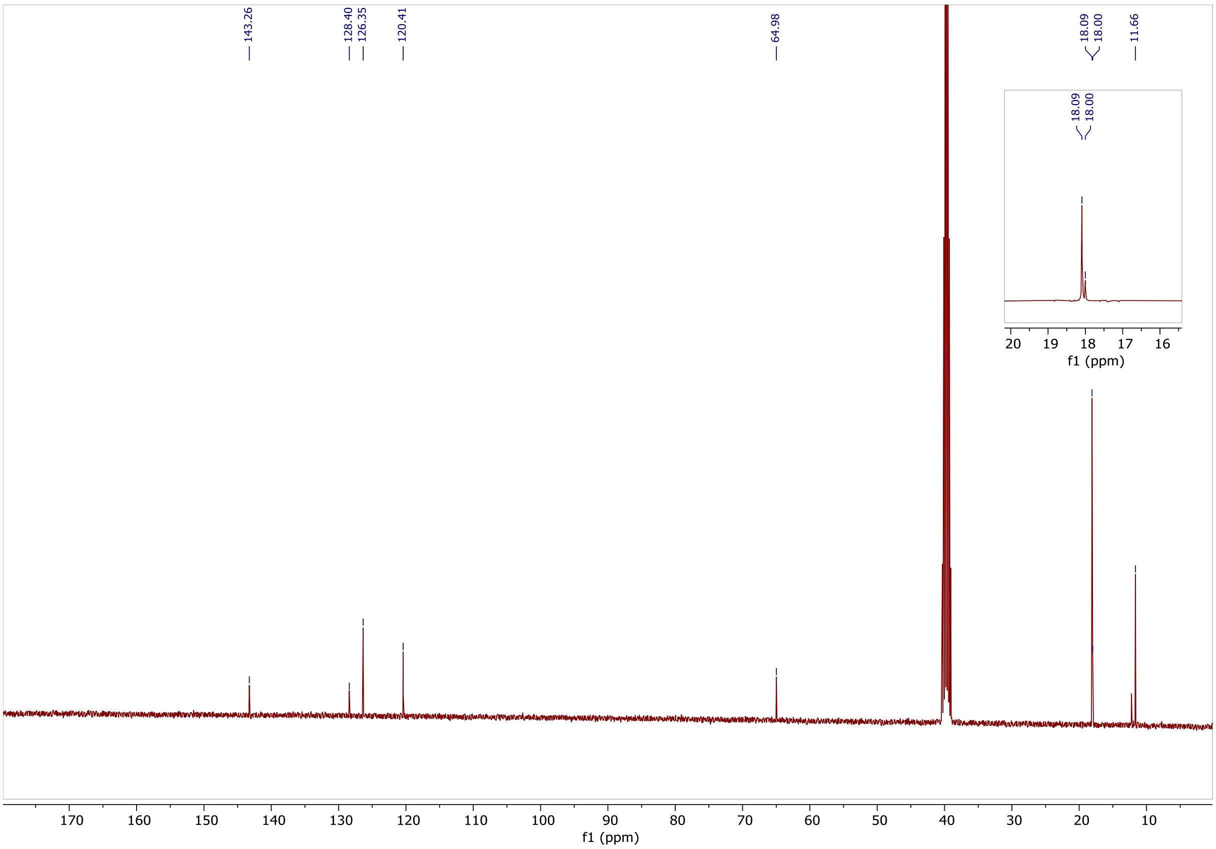

2-Chloro-*N*-[2,6-dimethyl-4-({[tris(propan-2-yl)silyl]oxy}methyl)phenyl]-5-nitropyrimidin-4-amine (25):

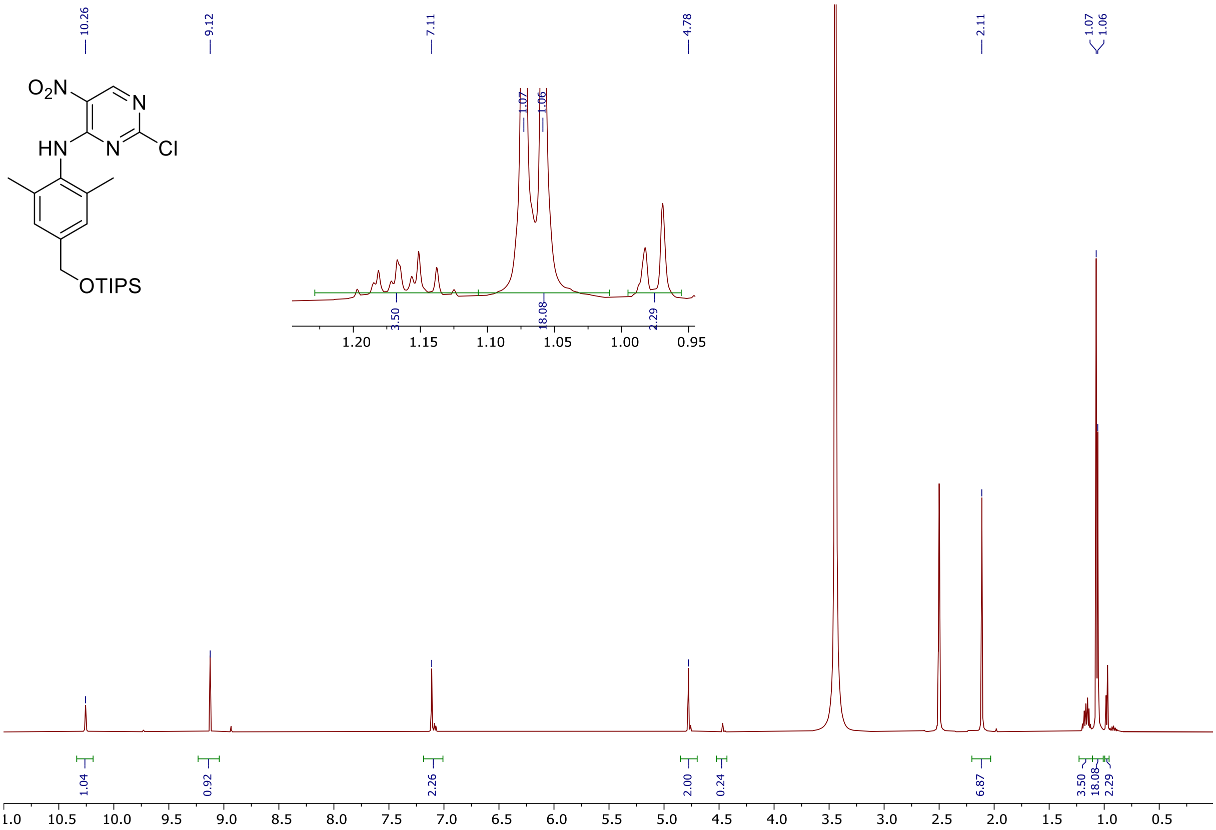

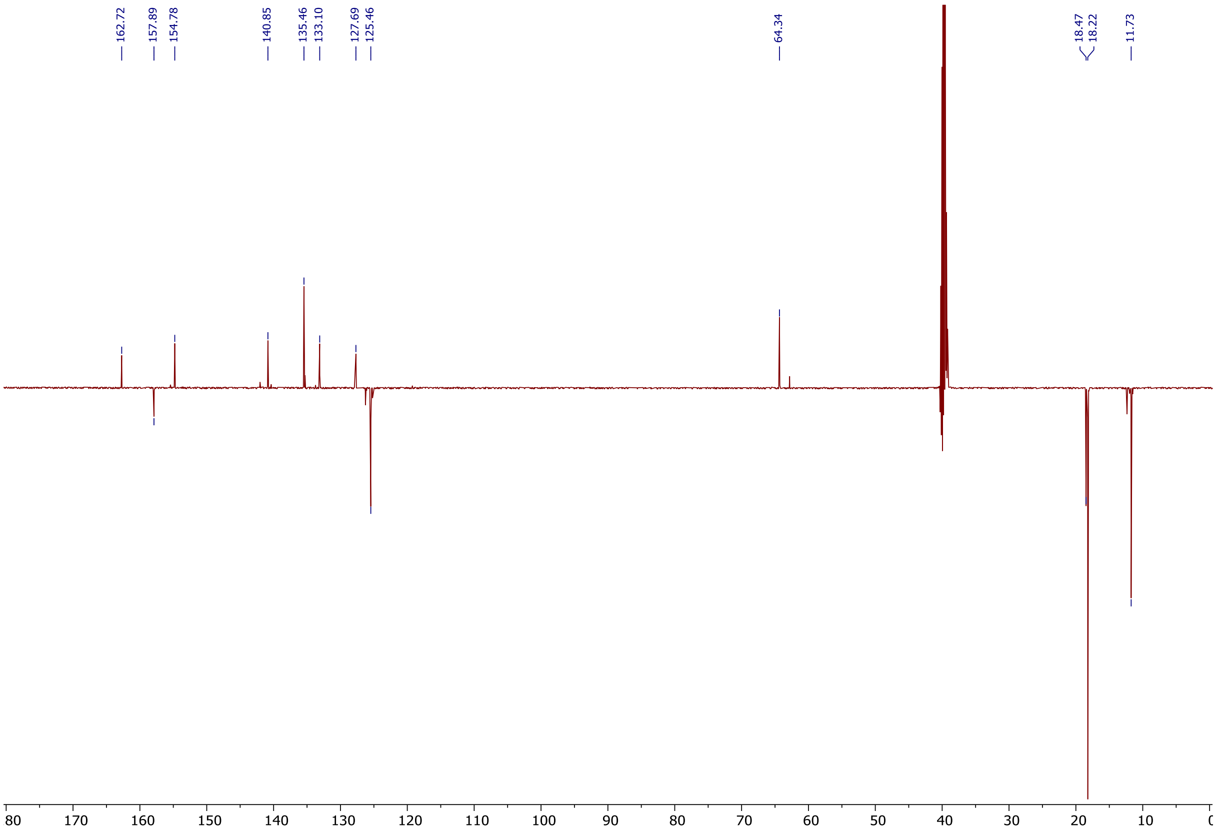

Ethyl 2-[(2-chloro-5-nitropyrimidin-4-yl)[2,6-dimethyl-4-({[tris(propan-2-yl)silyl]oxy}methyl)phenyl]amino]acetate (26):

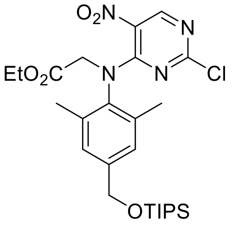

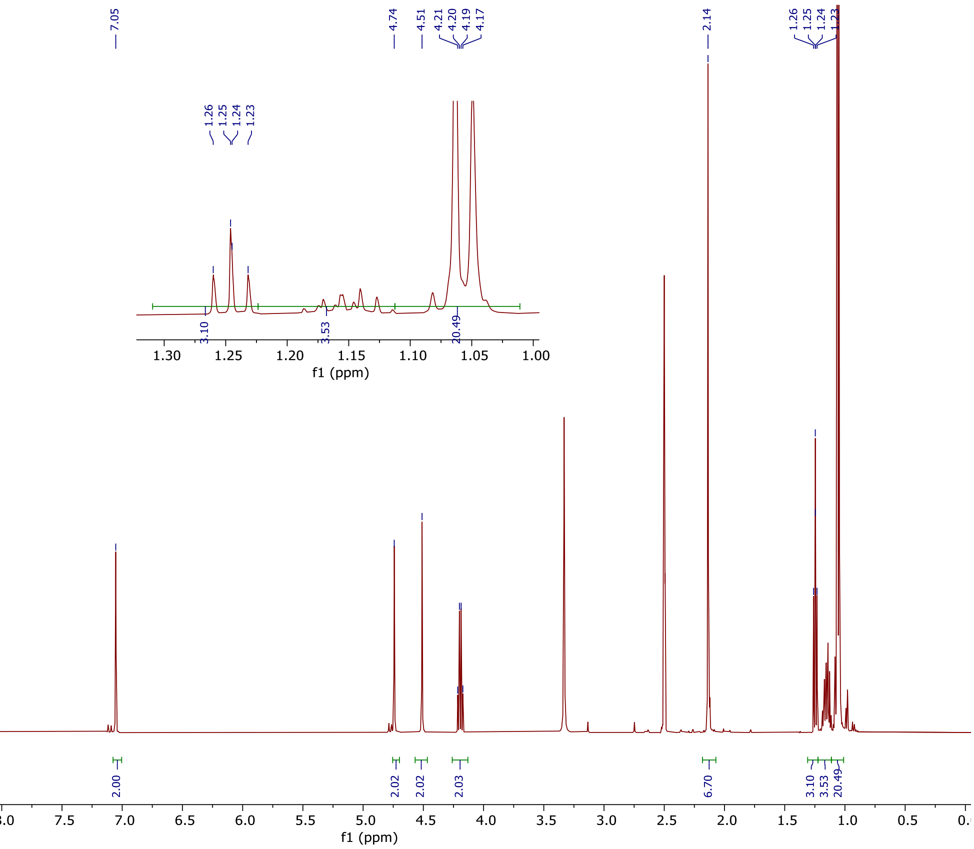

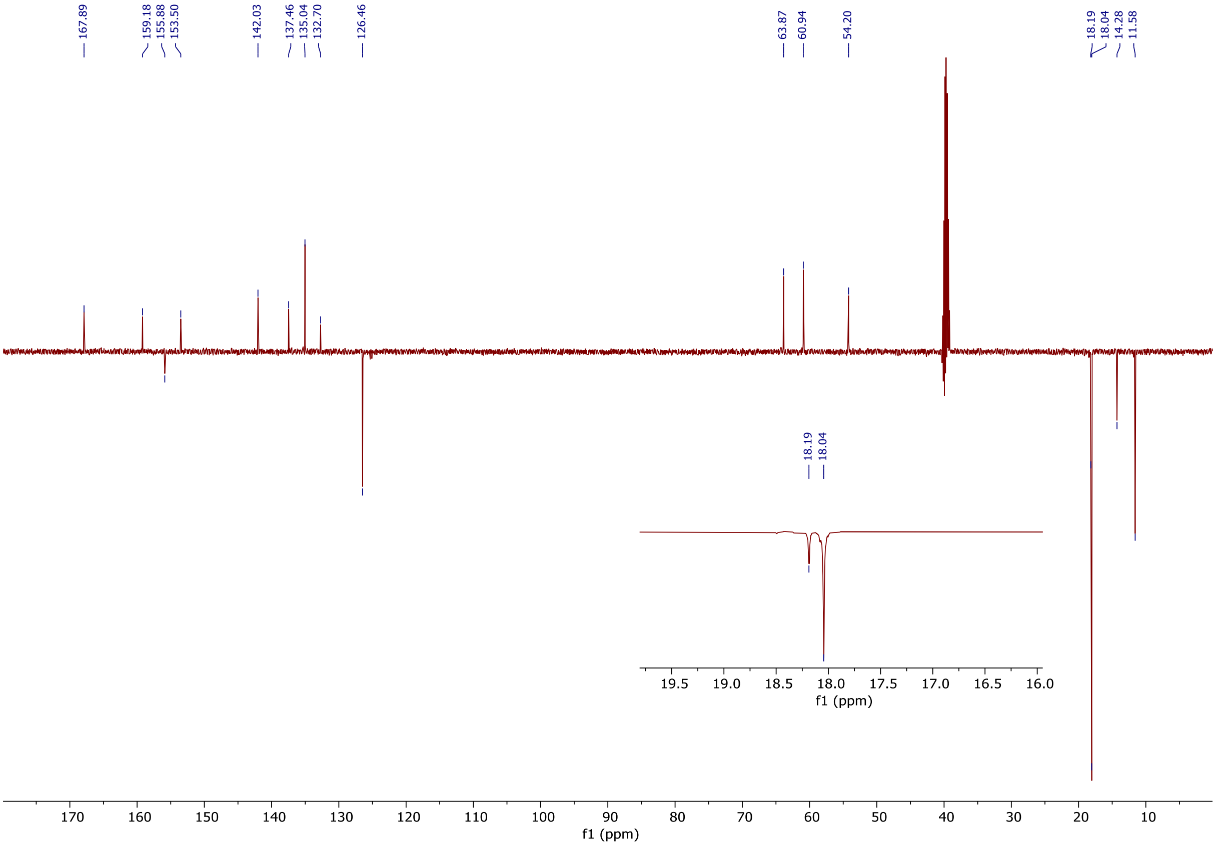

4-({8-[2,6-Dimethyl-4-({[tris(propan-2-yl)silyl]oxy}methyl)phenyl]-6-oxo-5,6,7,8-tetrahydropteridin-2-yl}amino)benzonitrile (27):

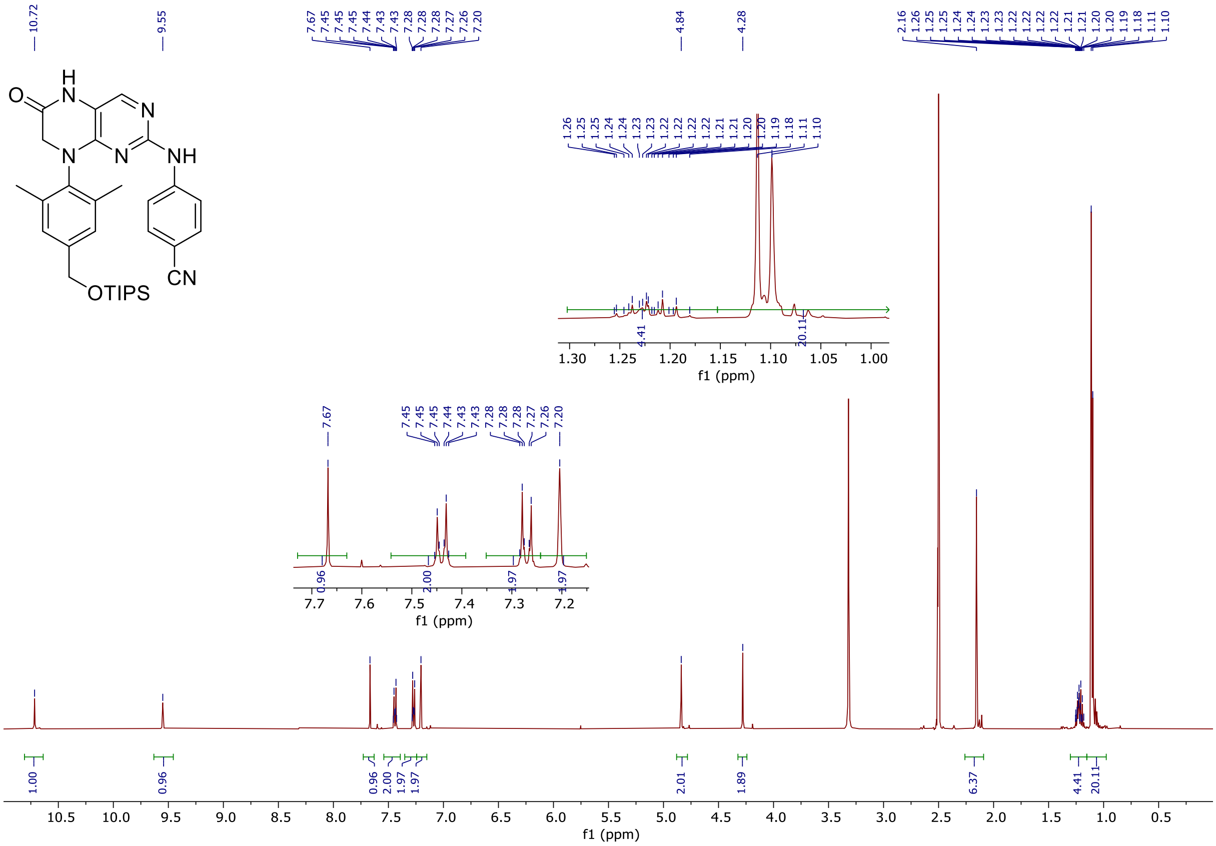

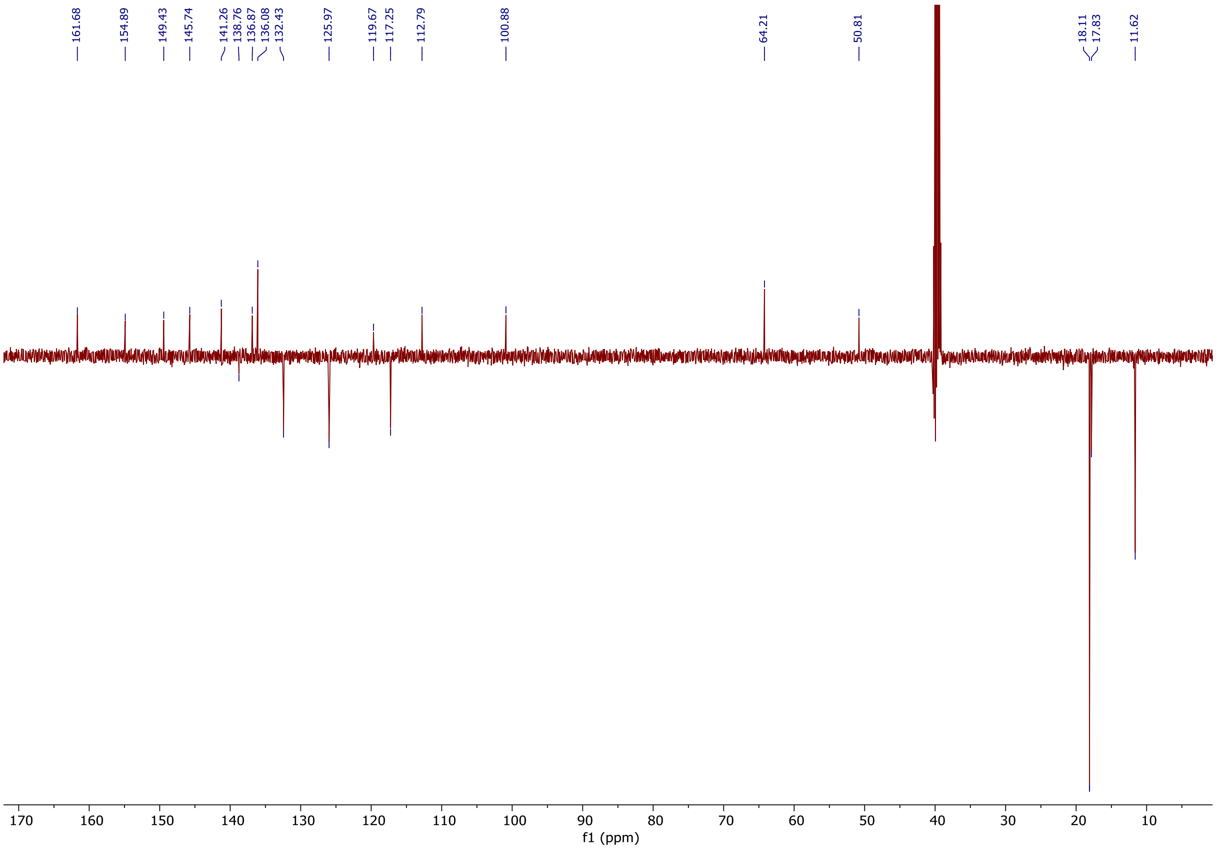

4-({8-[4-(hydroxymethyl)-2,6-dimethylphenyl]-6-oxo-5,6,7,8-tetrahydropteridin-2-yl}amino)benzonitrile (28):

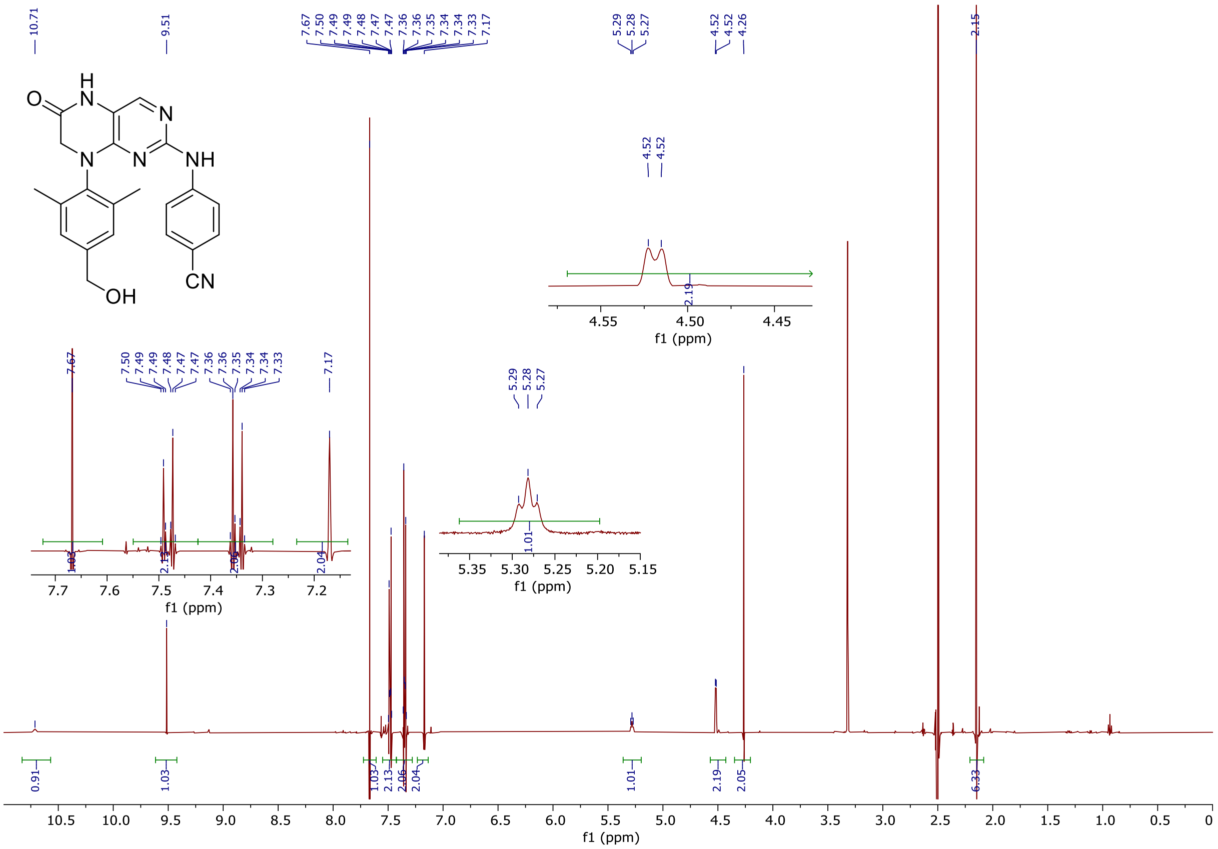

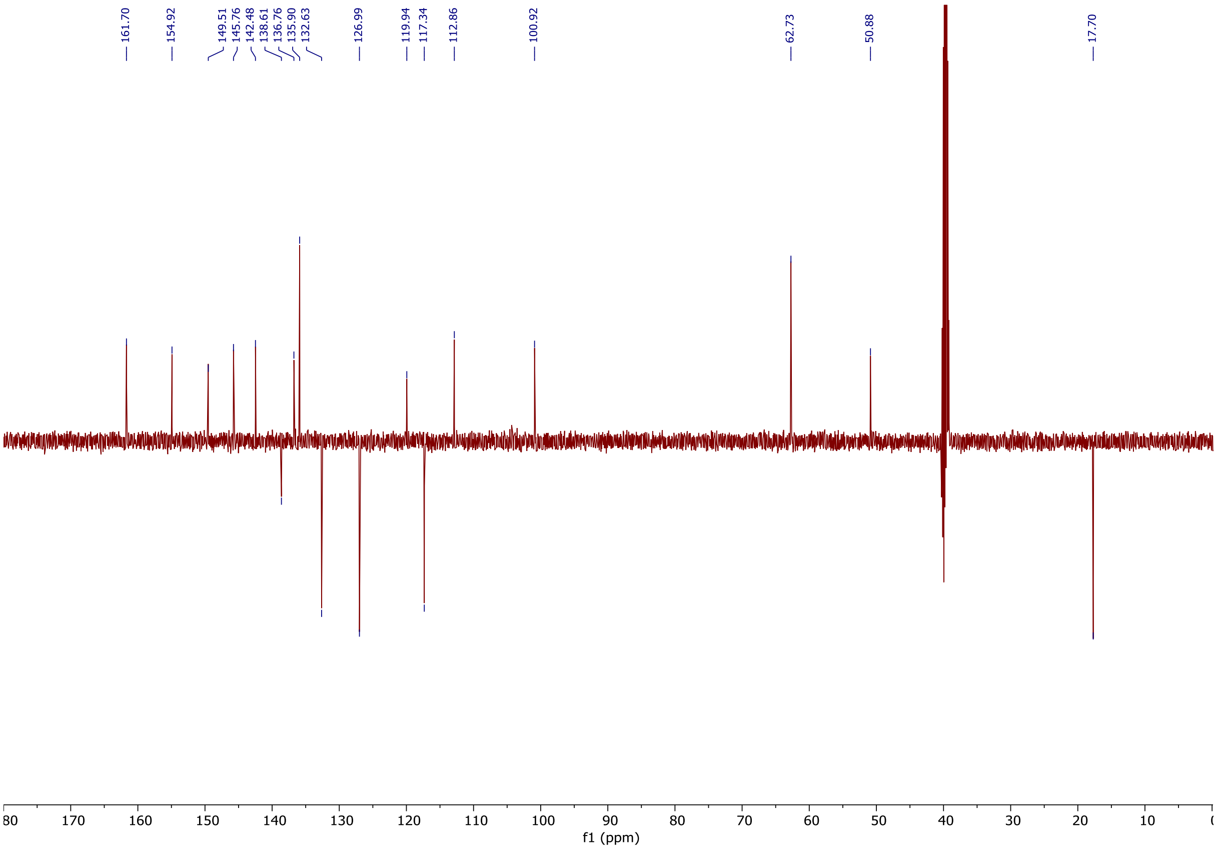

4-[(4-{[2,6-Dimethyl-4-({[tris(propan-2-yl)silyl]oxy}methyl)phenyl]amino}-5-nitropyrimidin-2-yl)amino]benzonitrile (29):

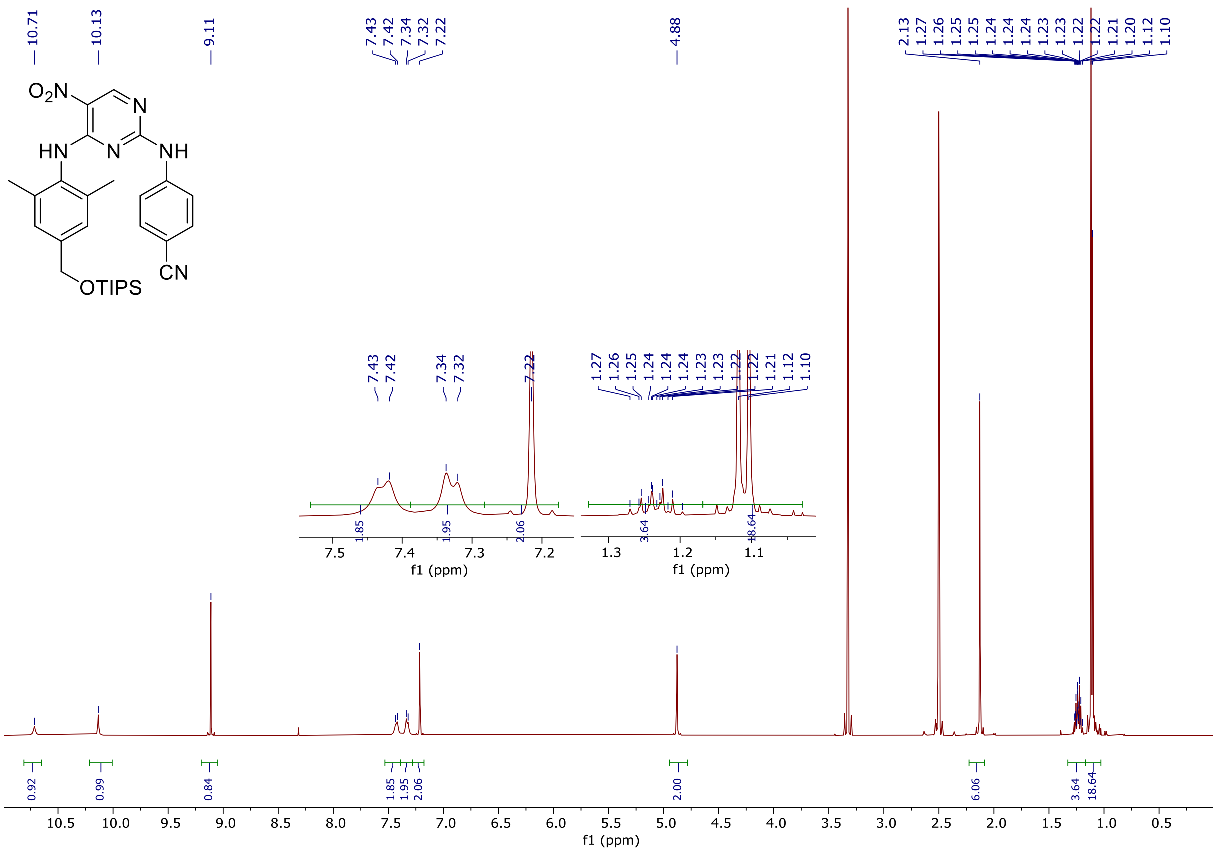

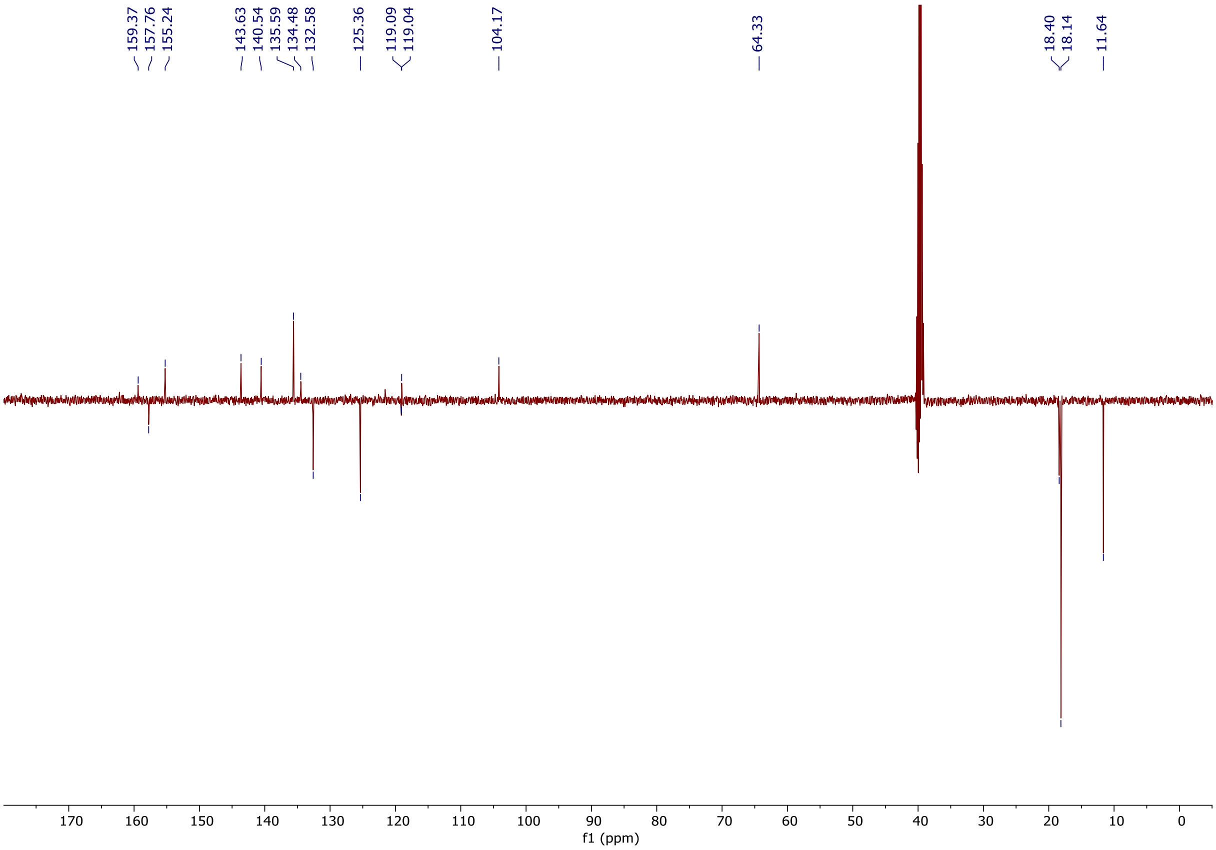

4-[(5-Amino-4-{[2,6-dimethyl-4-({[tris(propan-2-yl)silyl]oxy}methyl)phenyl]amino}pyrimidin-2-yl)amino]benzonitrile (30):

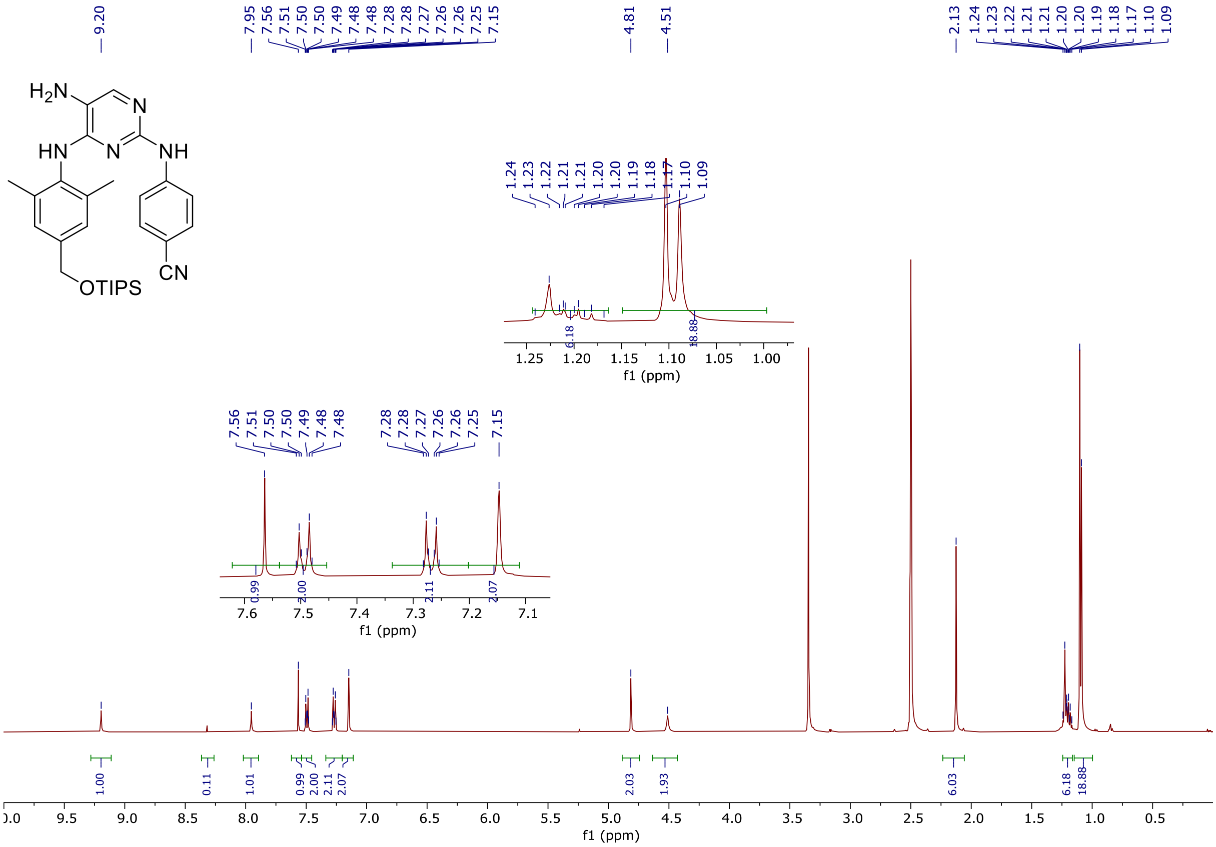

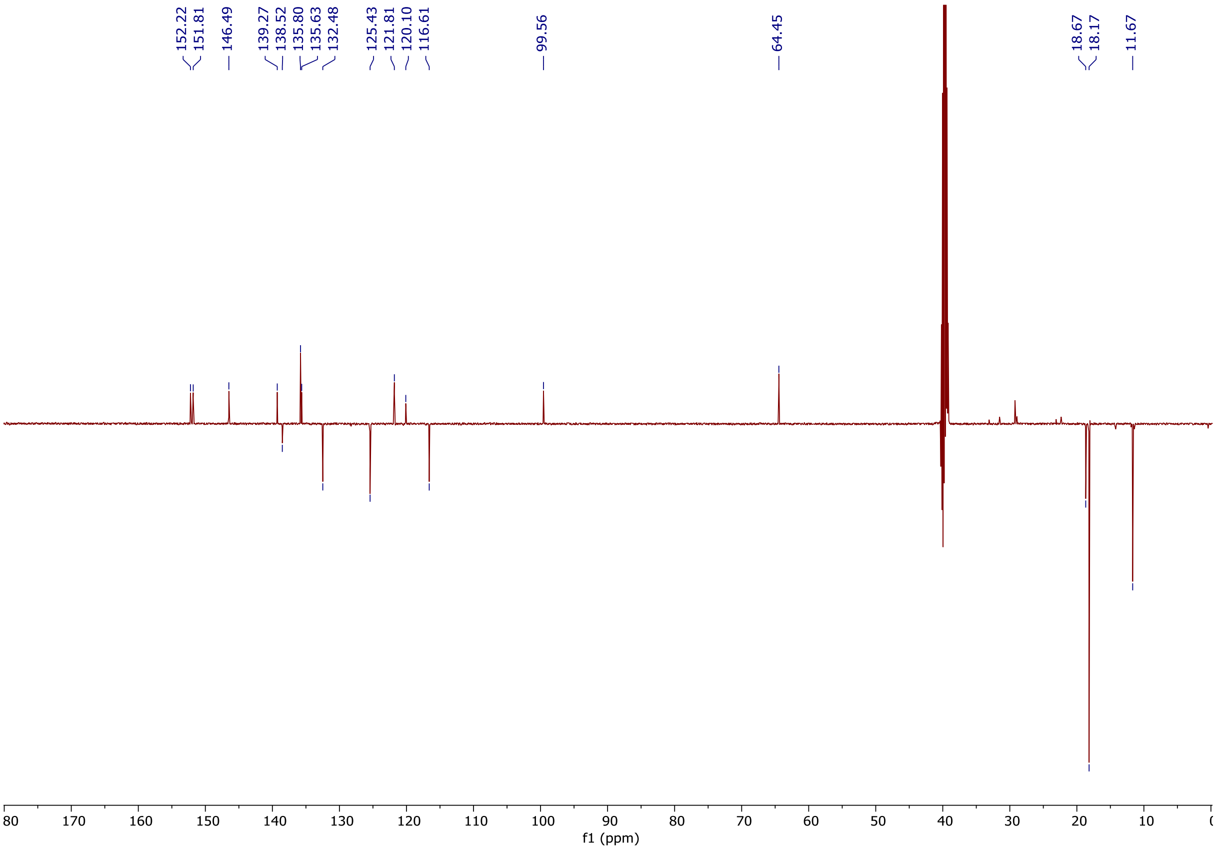

4-({9-[2,6-Dimethyl-4-({[tris(propan-2-yl)silyl]oxy}methyl)phenyl]-8-oxo-8,9-dihydro-7*H*-purin-2-yl}amino)benzonitrile (31):

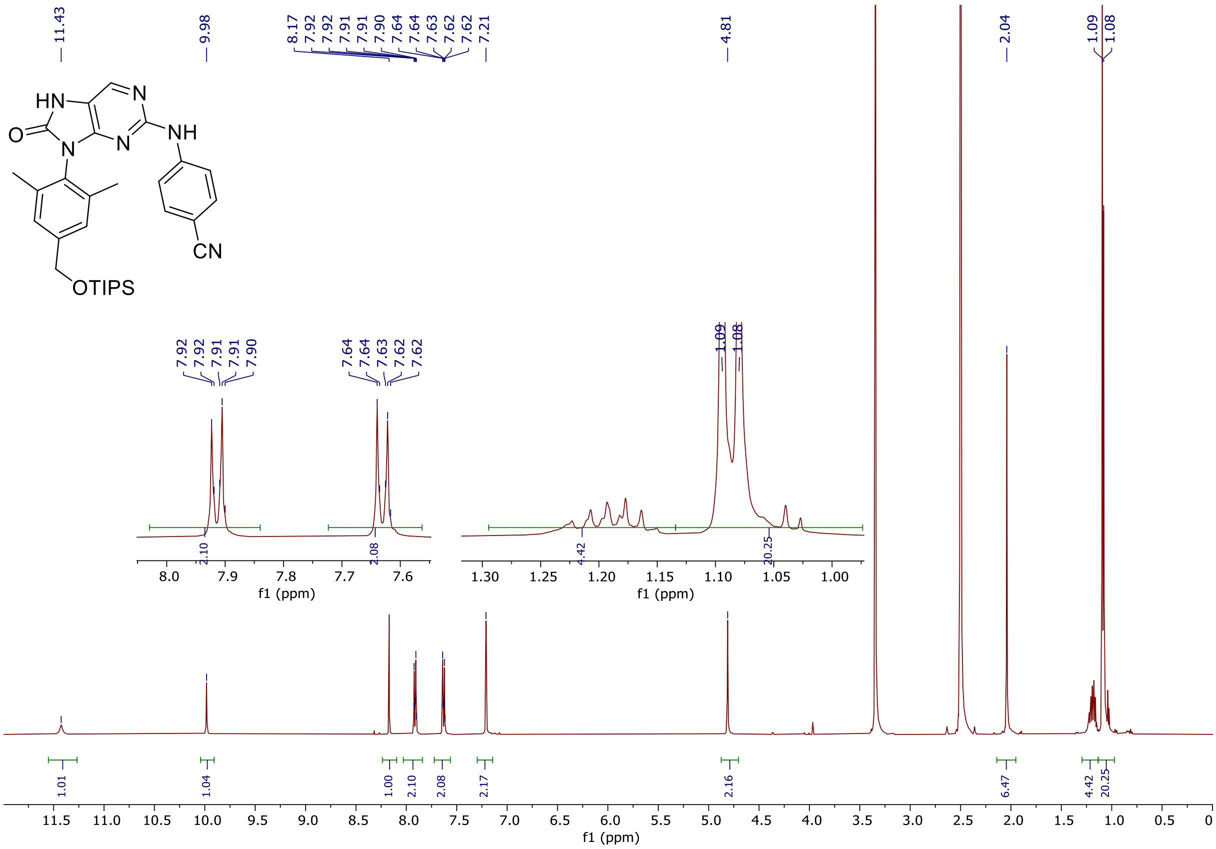

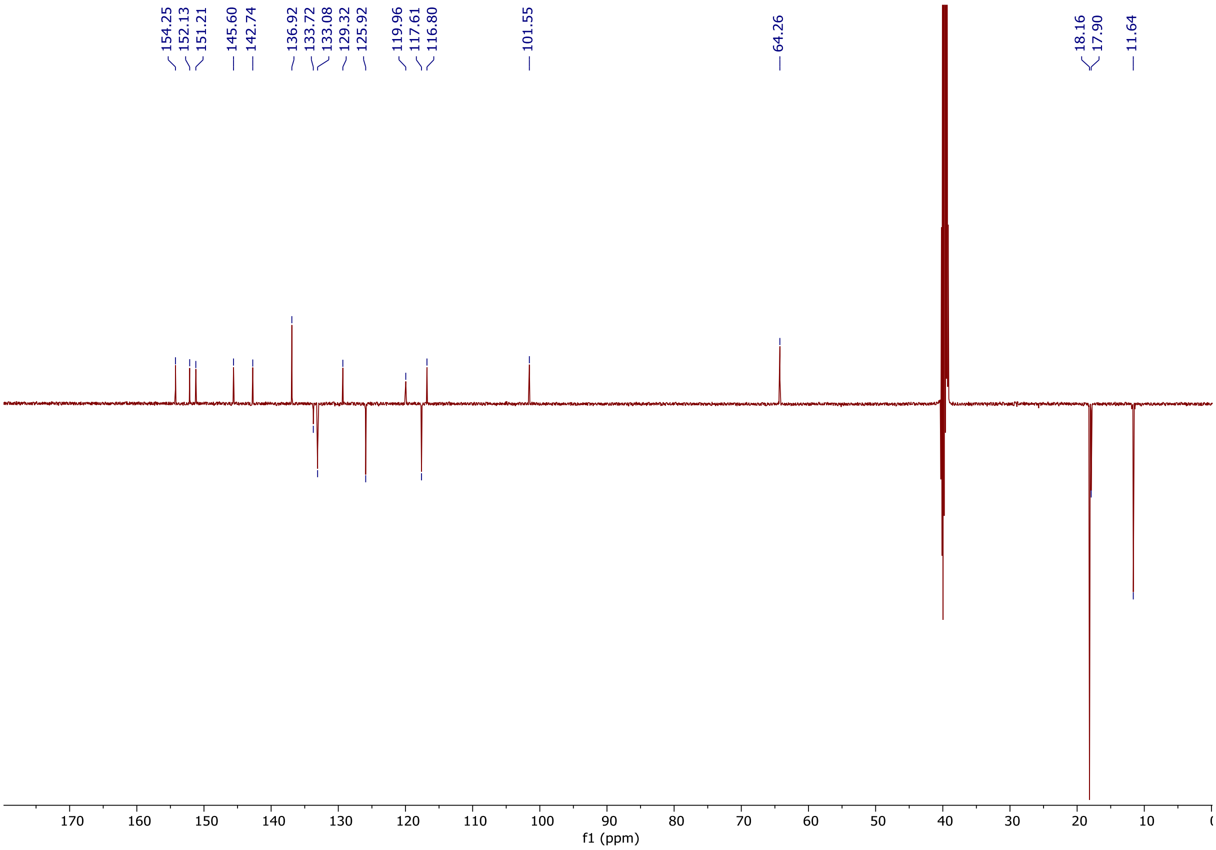

4-({9-[4-(Hydroxymethyl)-2,6-dimethylphenyl]-8-oxo-8,9-dihydro-7*H*-purin-2-yl}amino)benzonitrile (32):

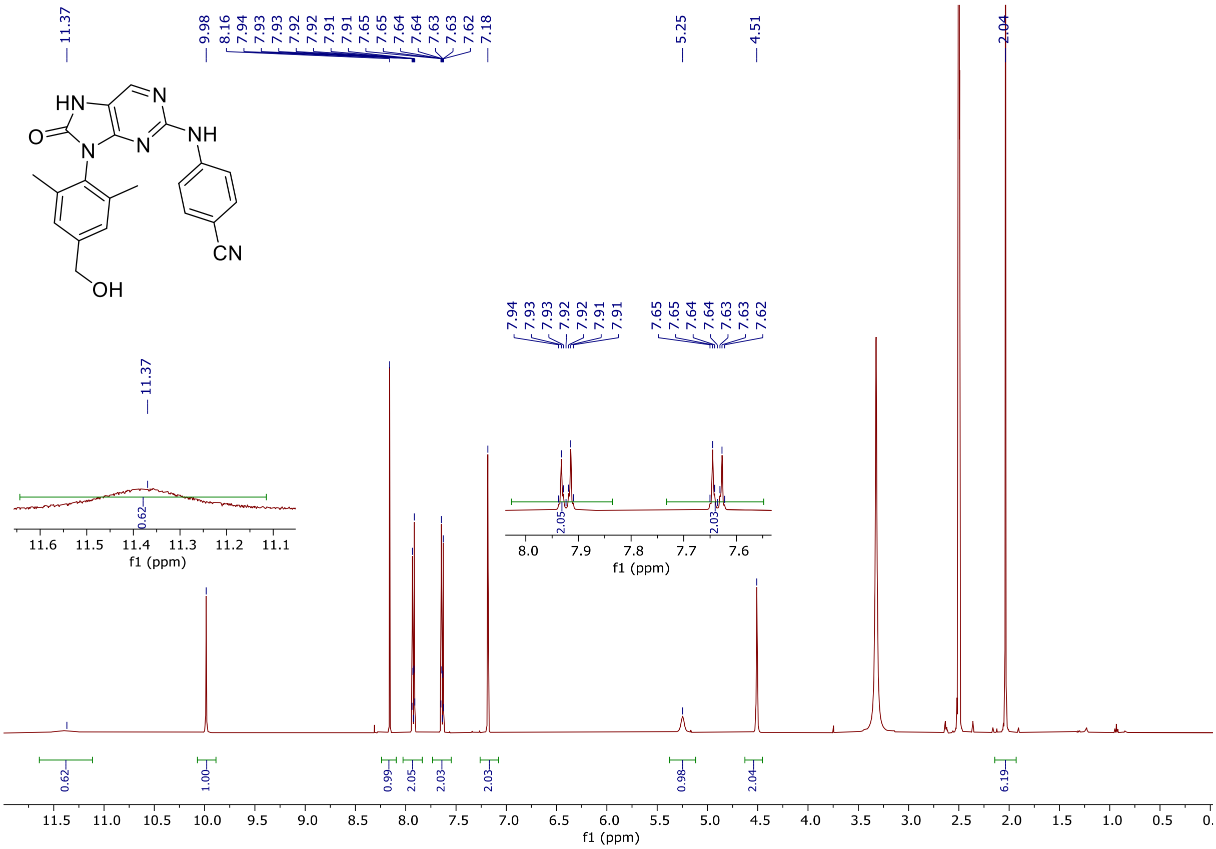

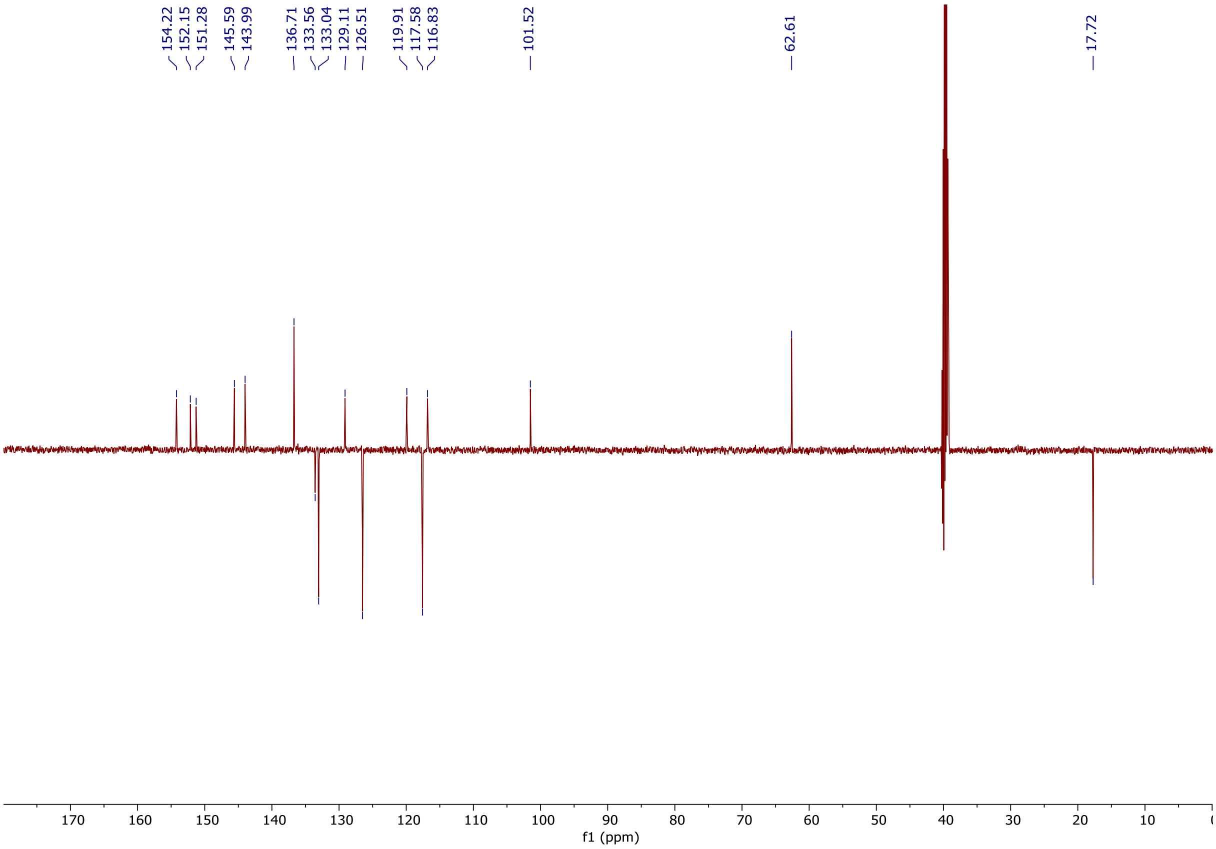

Ethyl 3-[(4-cyano-2,6-dimethylphenyl)amino]propanoate (33):

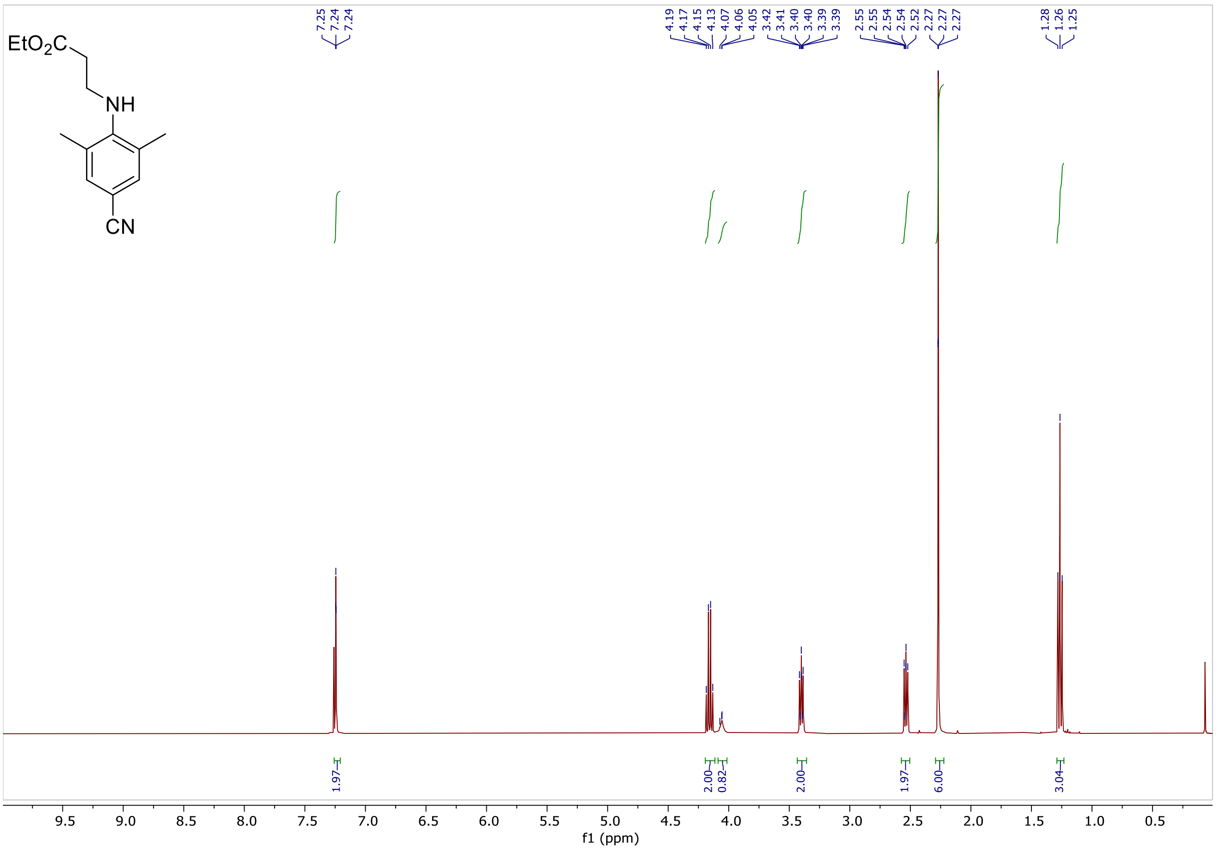

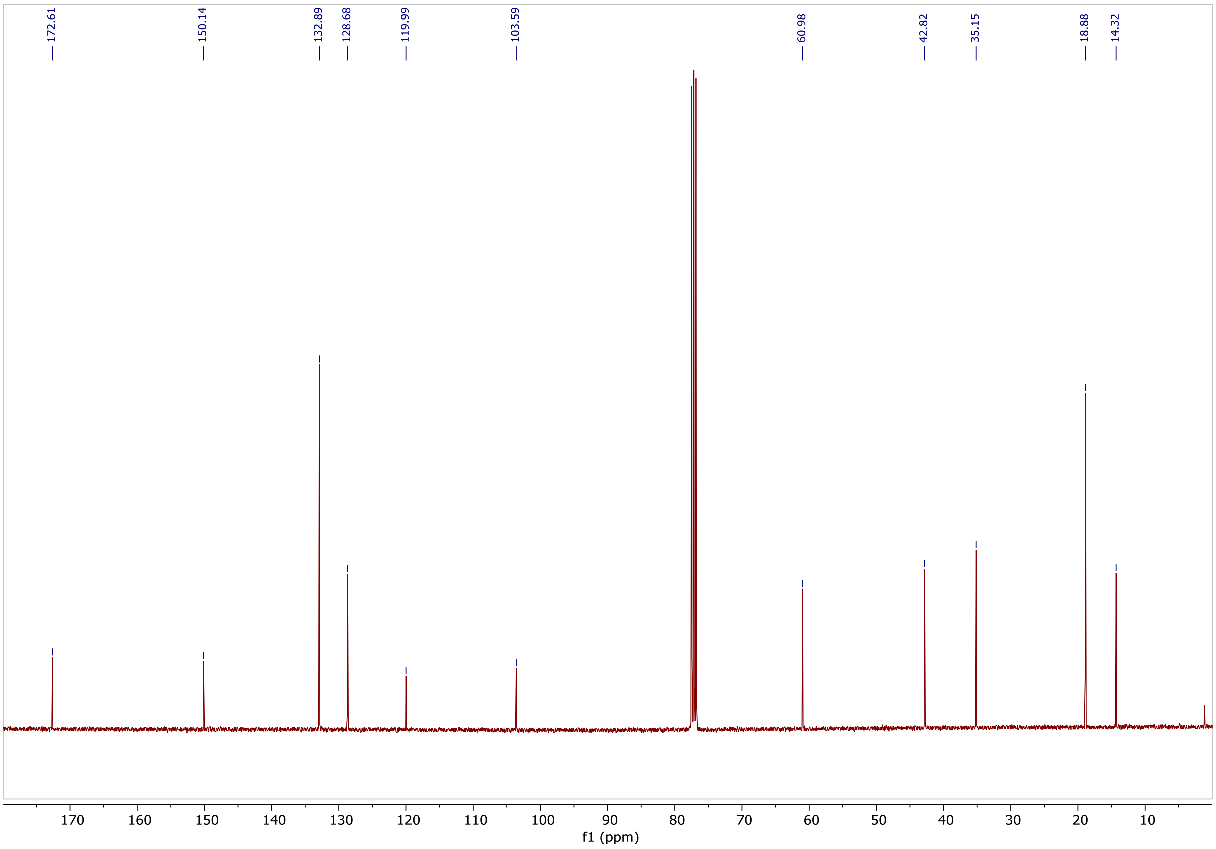

Ethyl 3-[(4-formyl-2,6-dimethylphenyl)amino]propanoate (34):

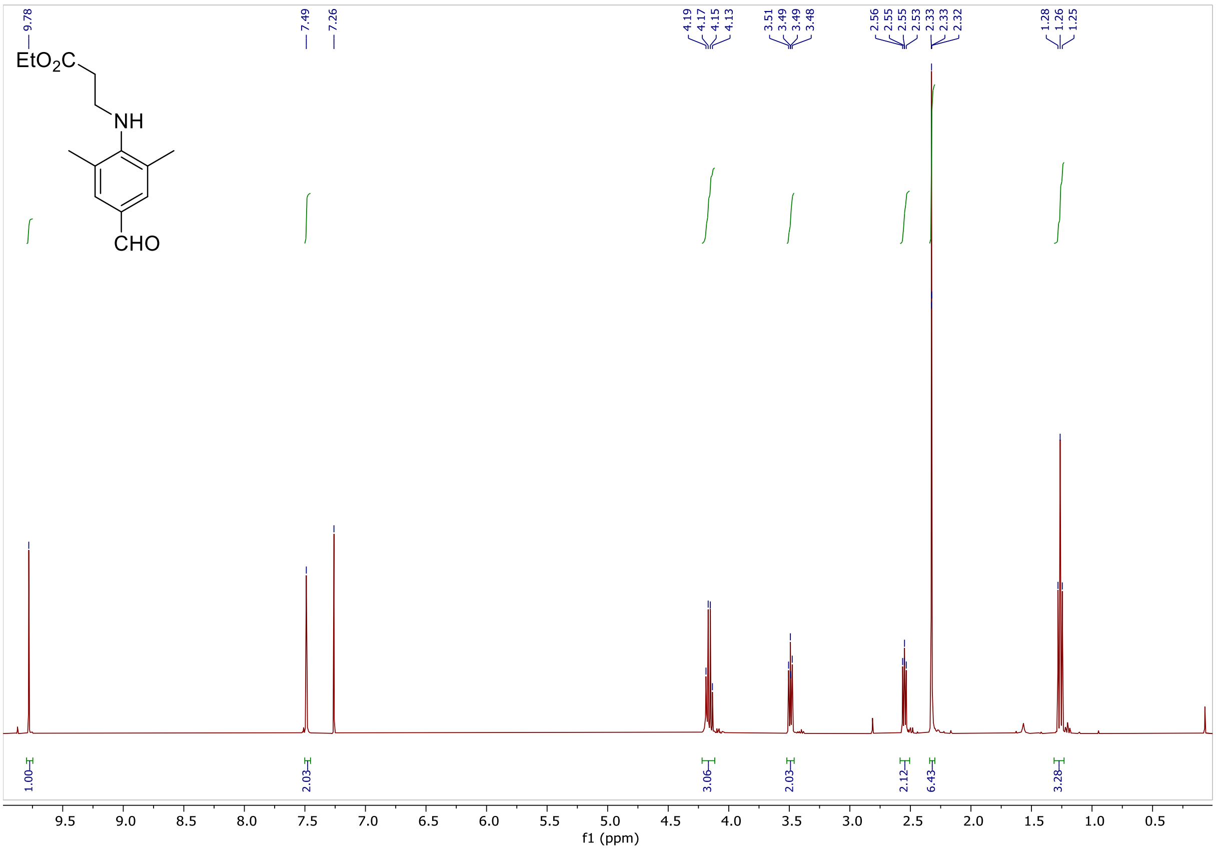

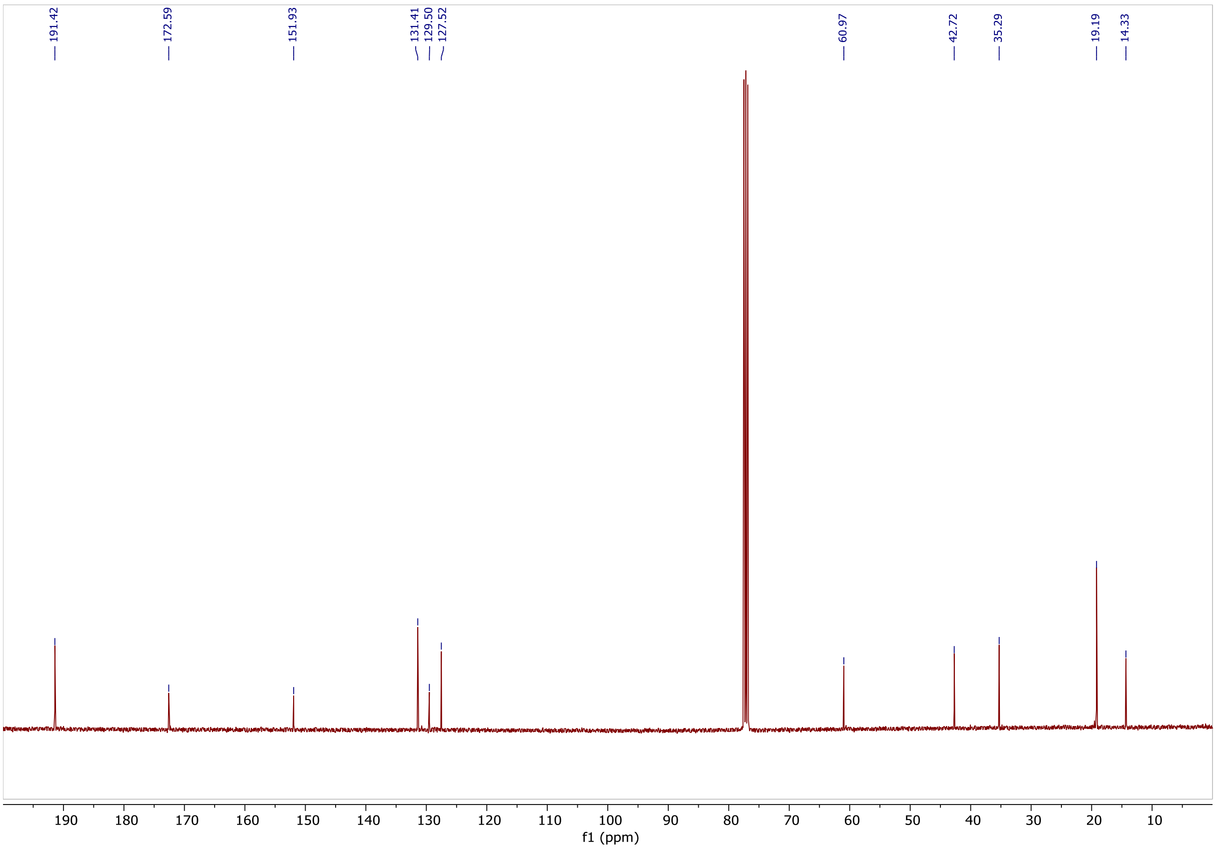

Ethyl 3-{[4-(hydroxymethyl)-2,6-dimethylphenyl]amino}propanoate (35):

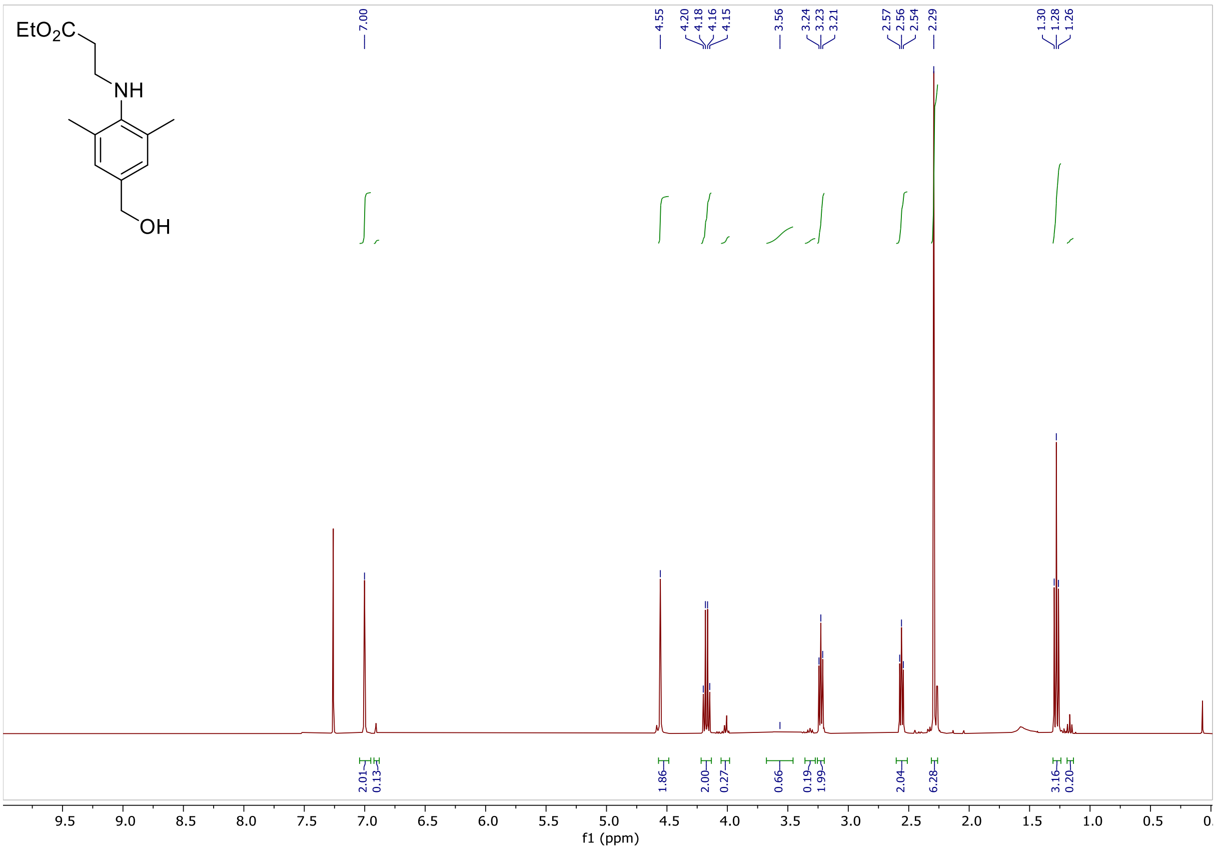

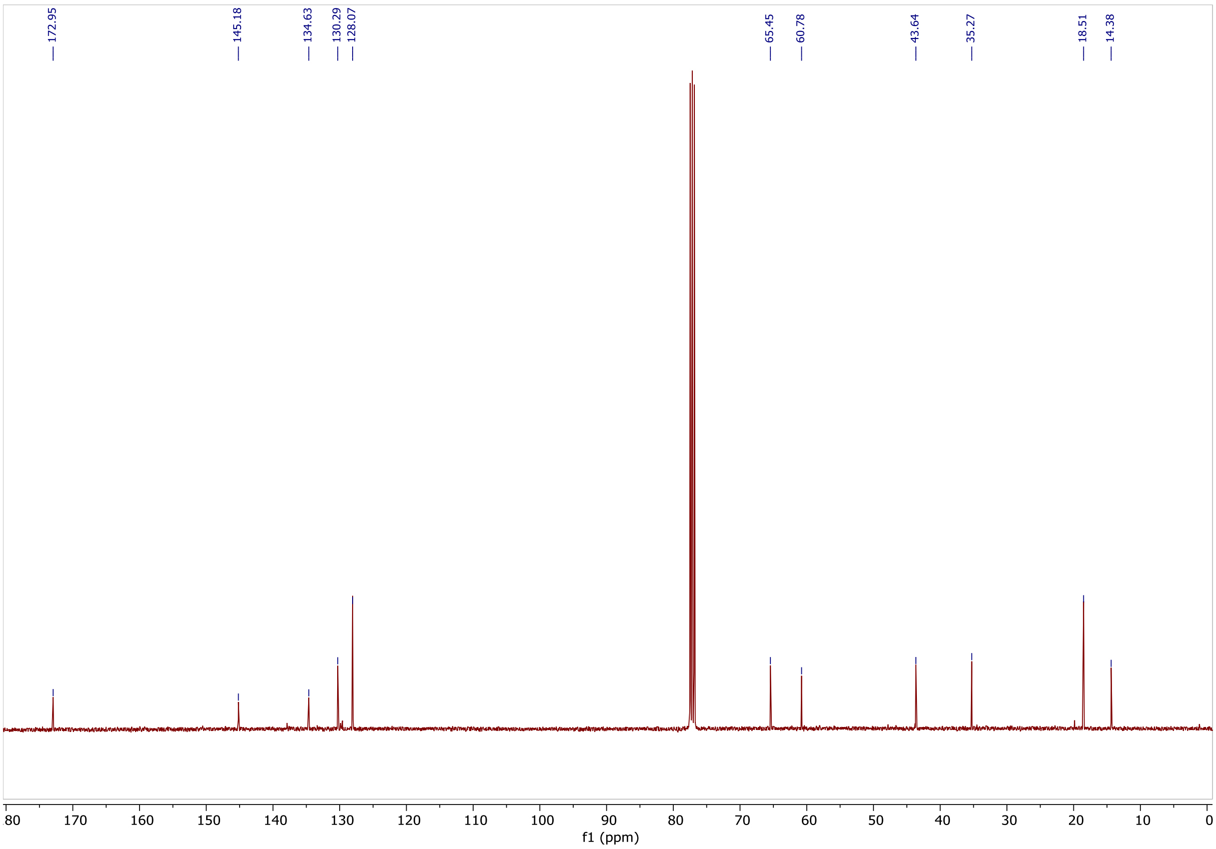

Ethyl 3-{[2,6-dimethyl-4-({[tris(propan-2-yl)silyl]oxy}methyl)phenyl]amino}propanoate (36):

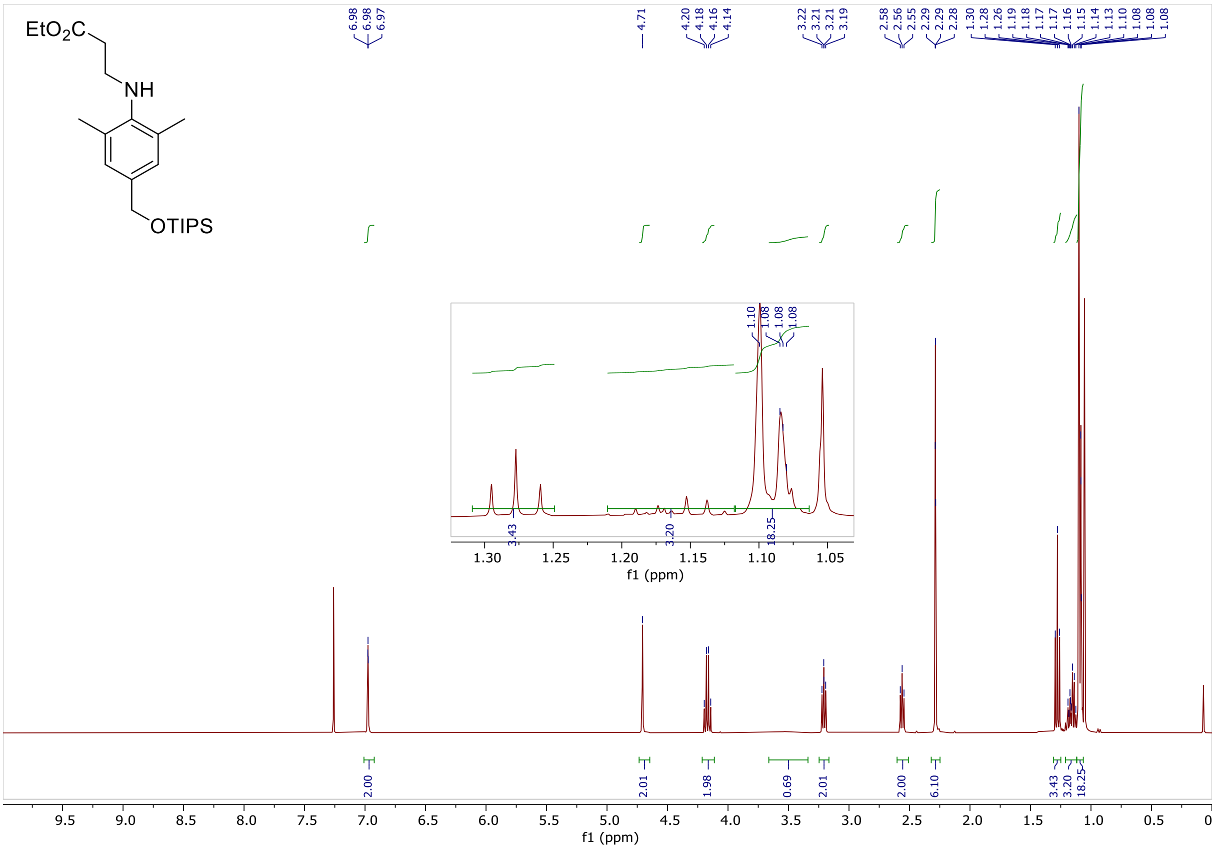

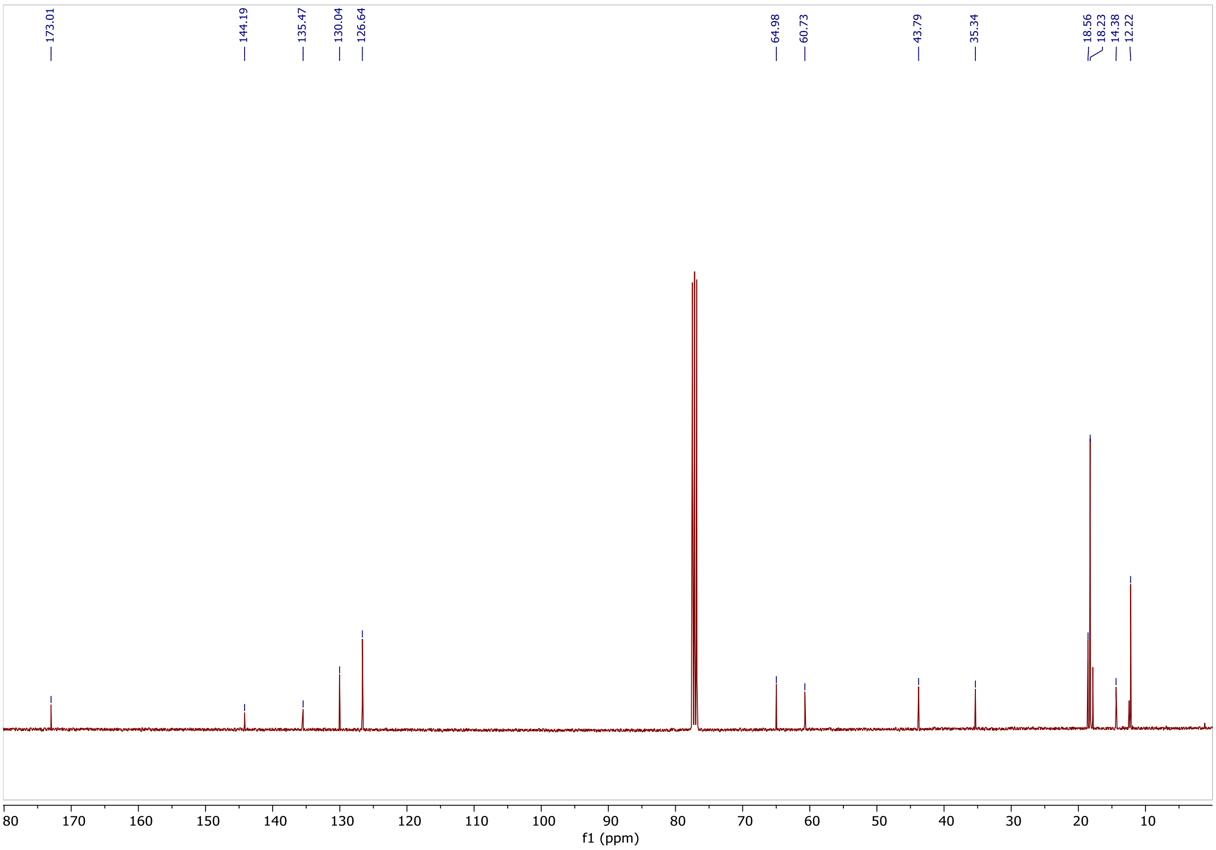

Ethyl 3-{[2,6-dimethyl-4-({[tris(propan-2-yl)silyl]oxy}methyl)phenyl]amino}propanoate (37):

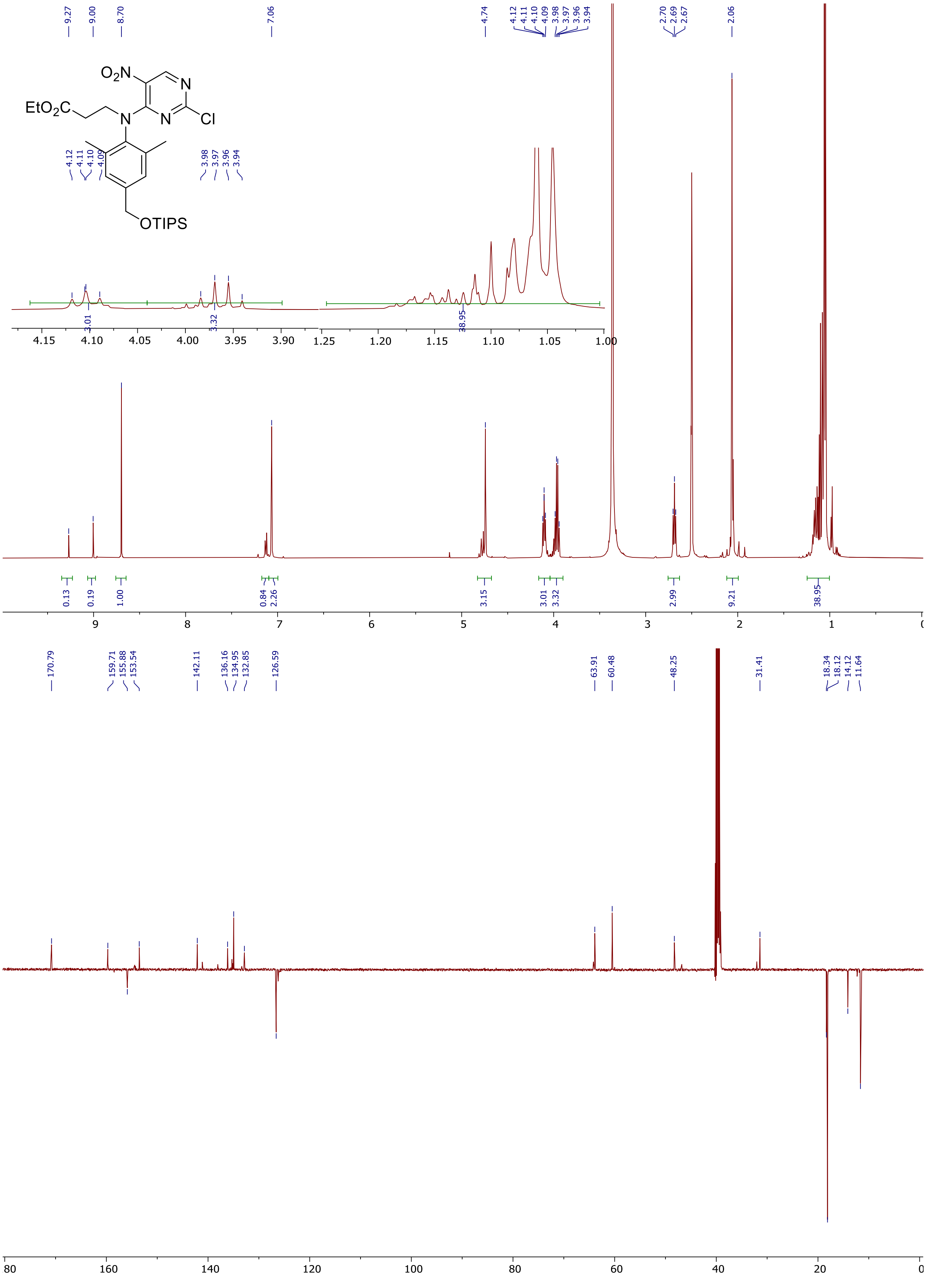

4-({9-[2,6-Dimethyl-4-({[tris(propan-2-yl)silyl]oxy}methyl)phenyl]-6-oxo-5*H*,6*H*,7*H*,8*H*,9*H*-pyrimido[4,5-*b*][1,4]diazepin-2-yl}amino)benzonitrile (38):

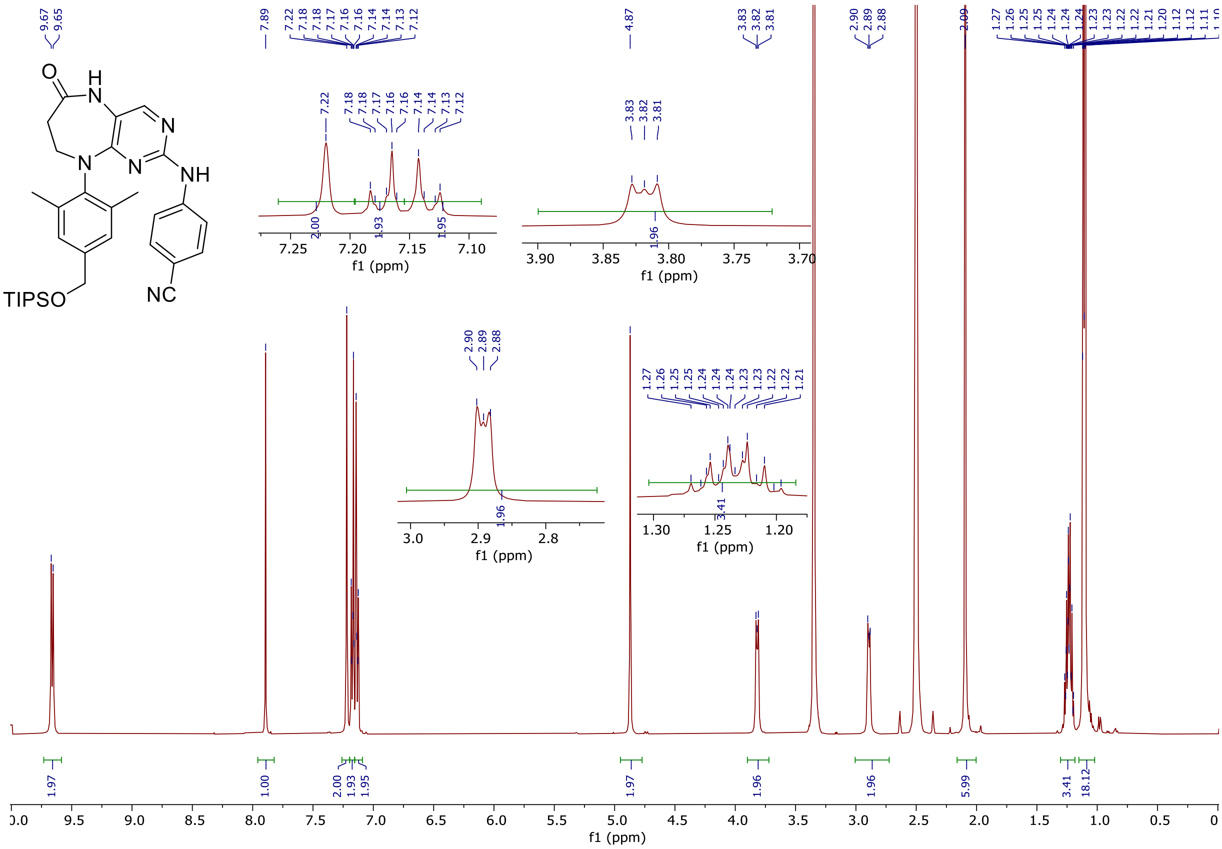

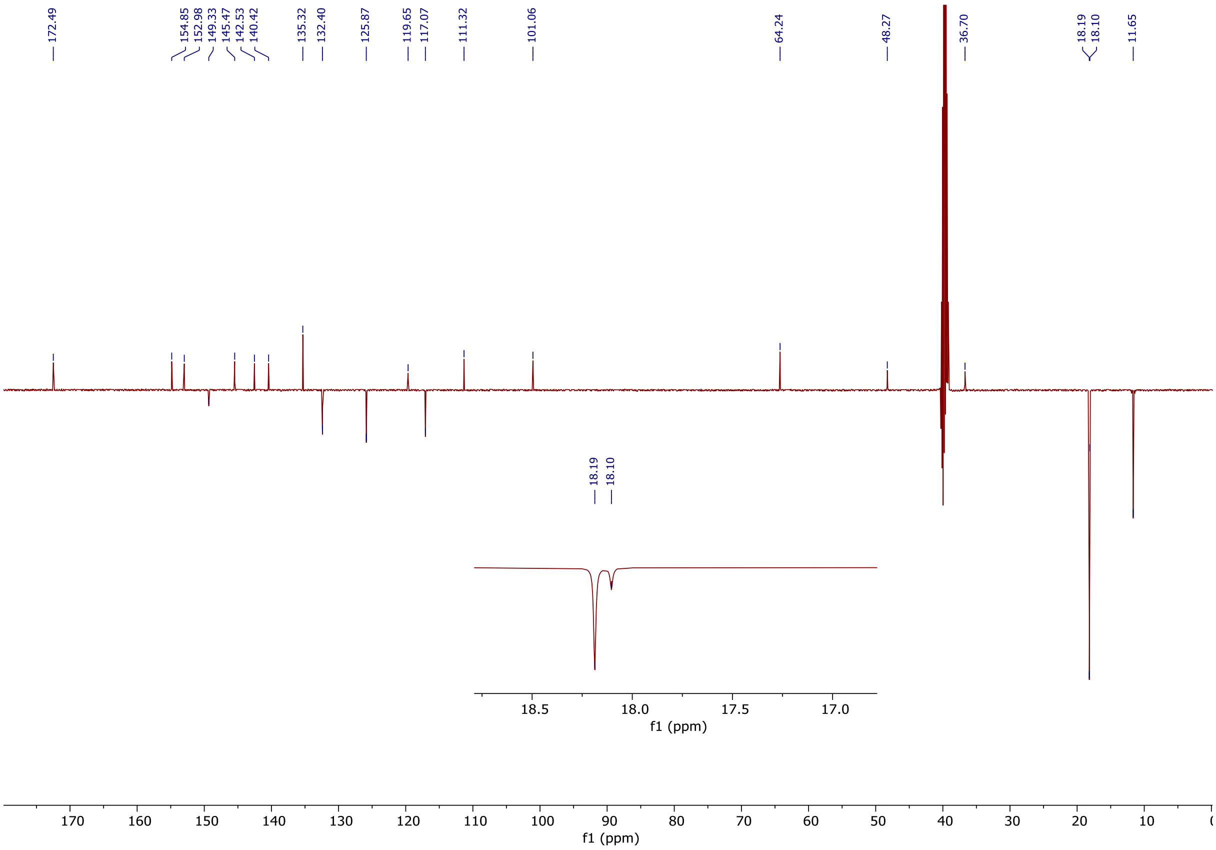

4-({9-[4-(hydroxymethyl)-2,6-dimethylphenyl]-6-oxo-5*H*,6*H*,7*H*,8*H*,9*H*-pyrimido[4,5-*b*][1,4]diazepin-2-yl}amino)benzonitrile (39):

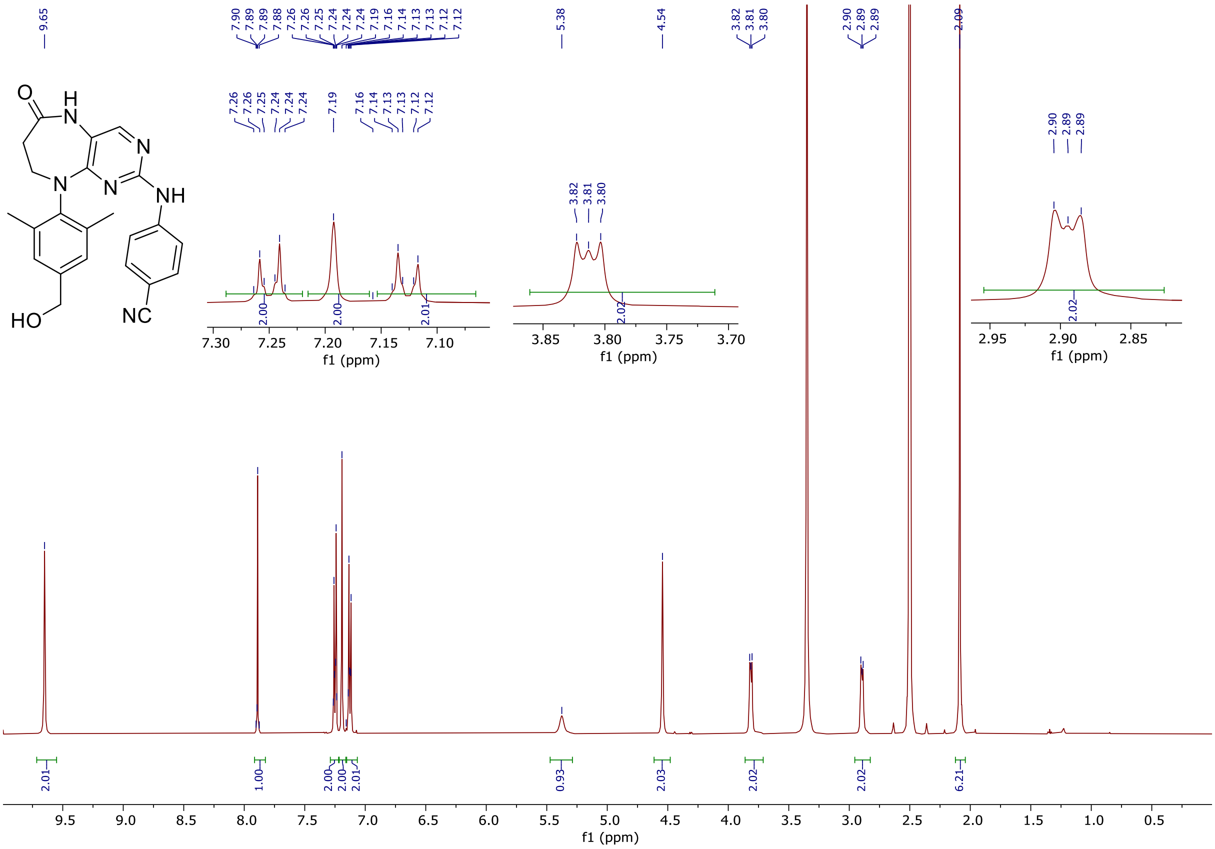

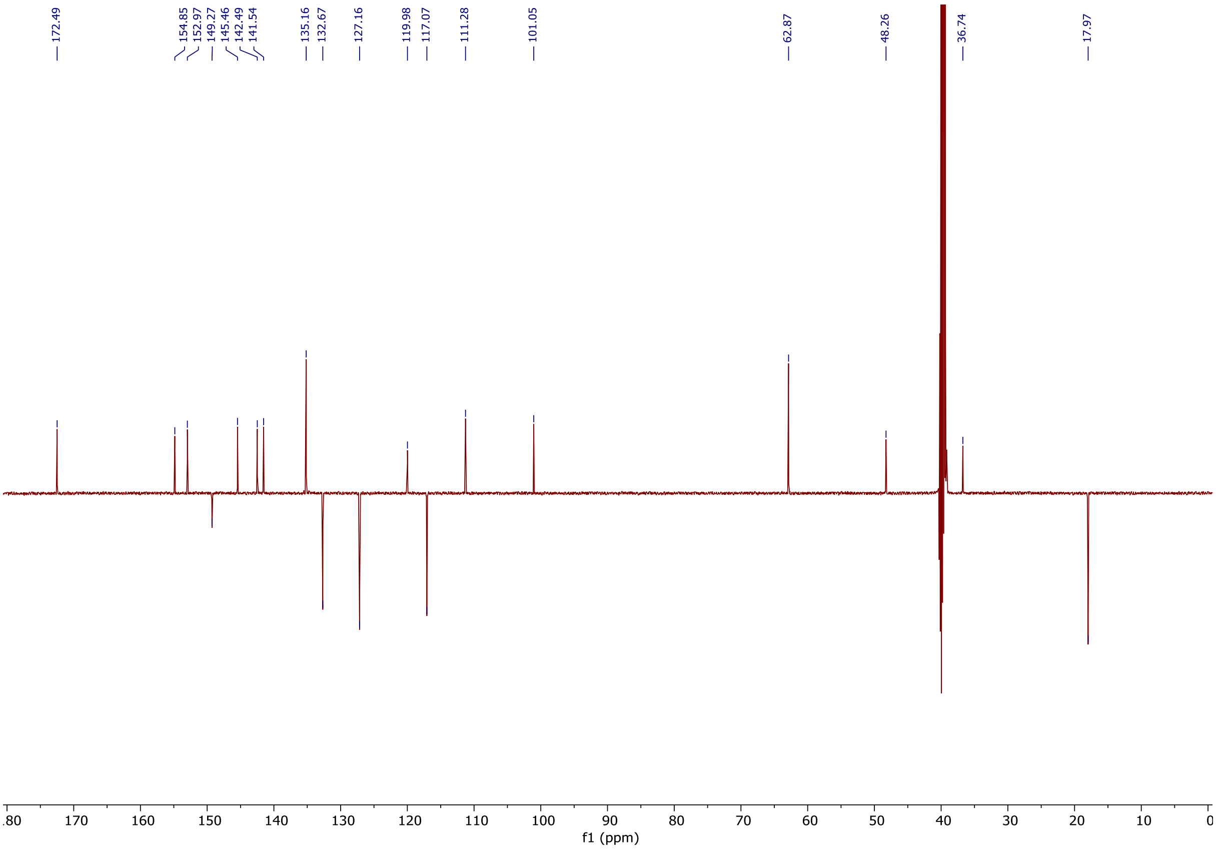

4-{[9-(4-Formyl-2,6-dimethylphenyl)-8-oxo-8,9-dihydro-7*H*-purin-2-yl]amino}benzonitrile (40):

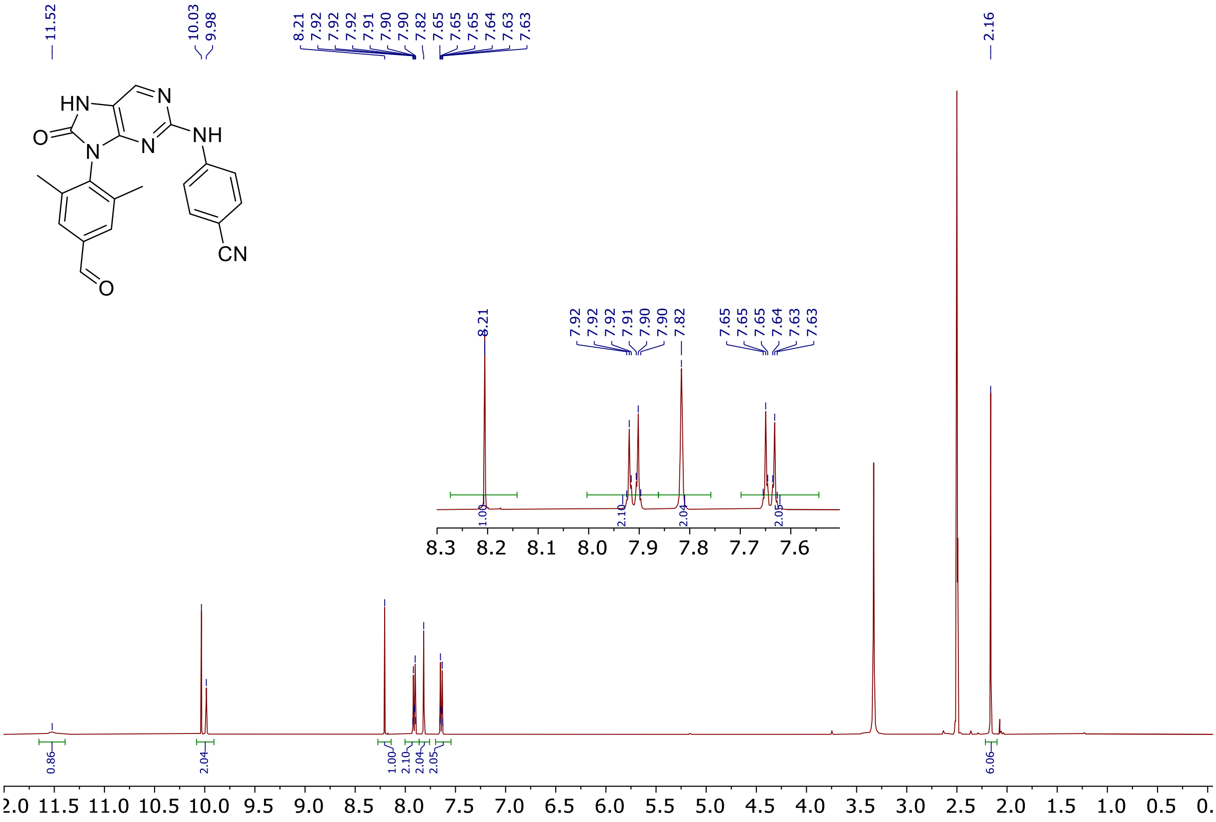

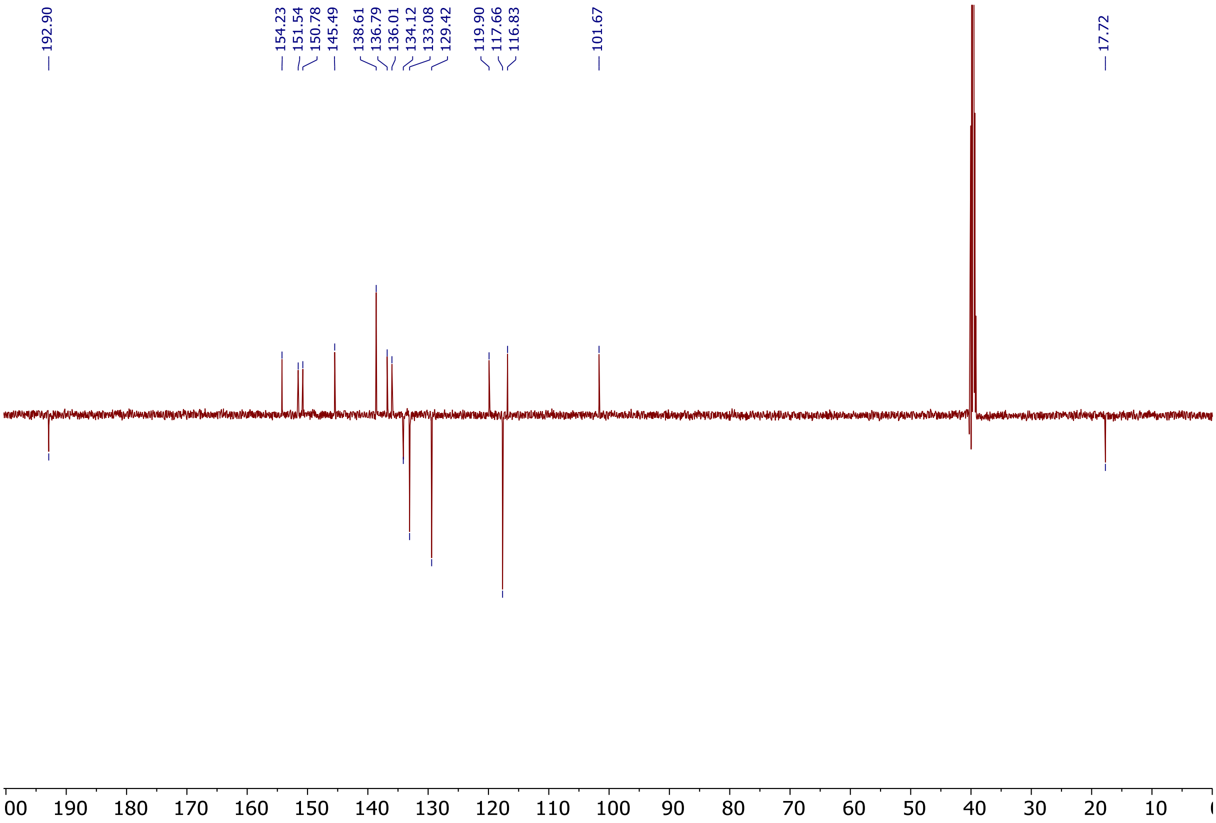

4-{[8-(4-Formyl-2,6-dimethylphenyl)-6-oxo-5,6,7,8-tetrahydropteridin-2-yl]amino}benzonitrile (41):

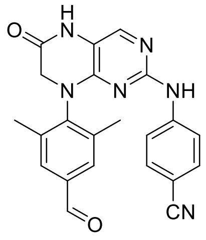

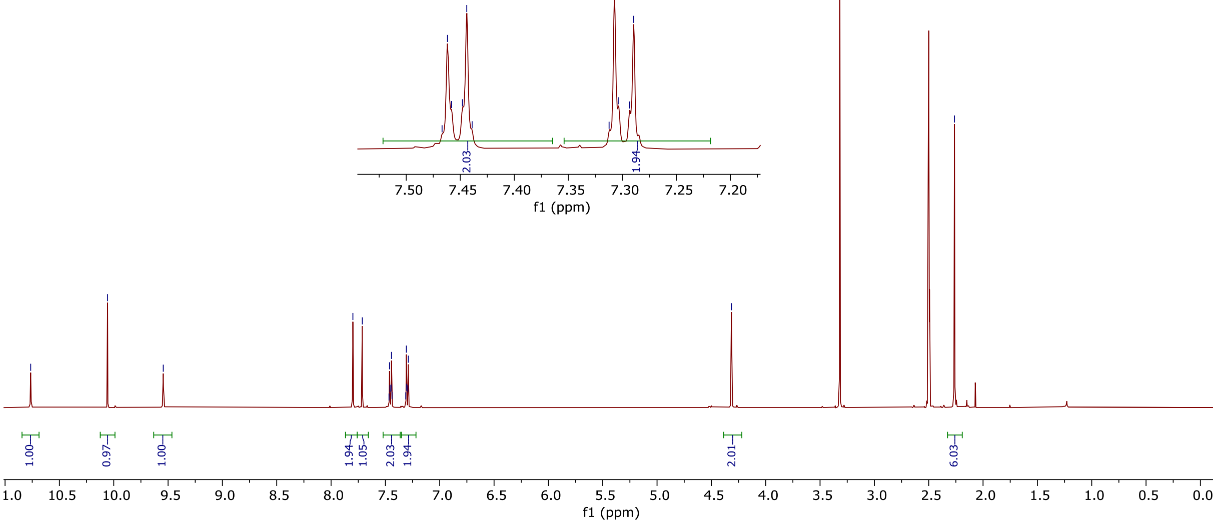

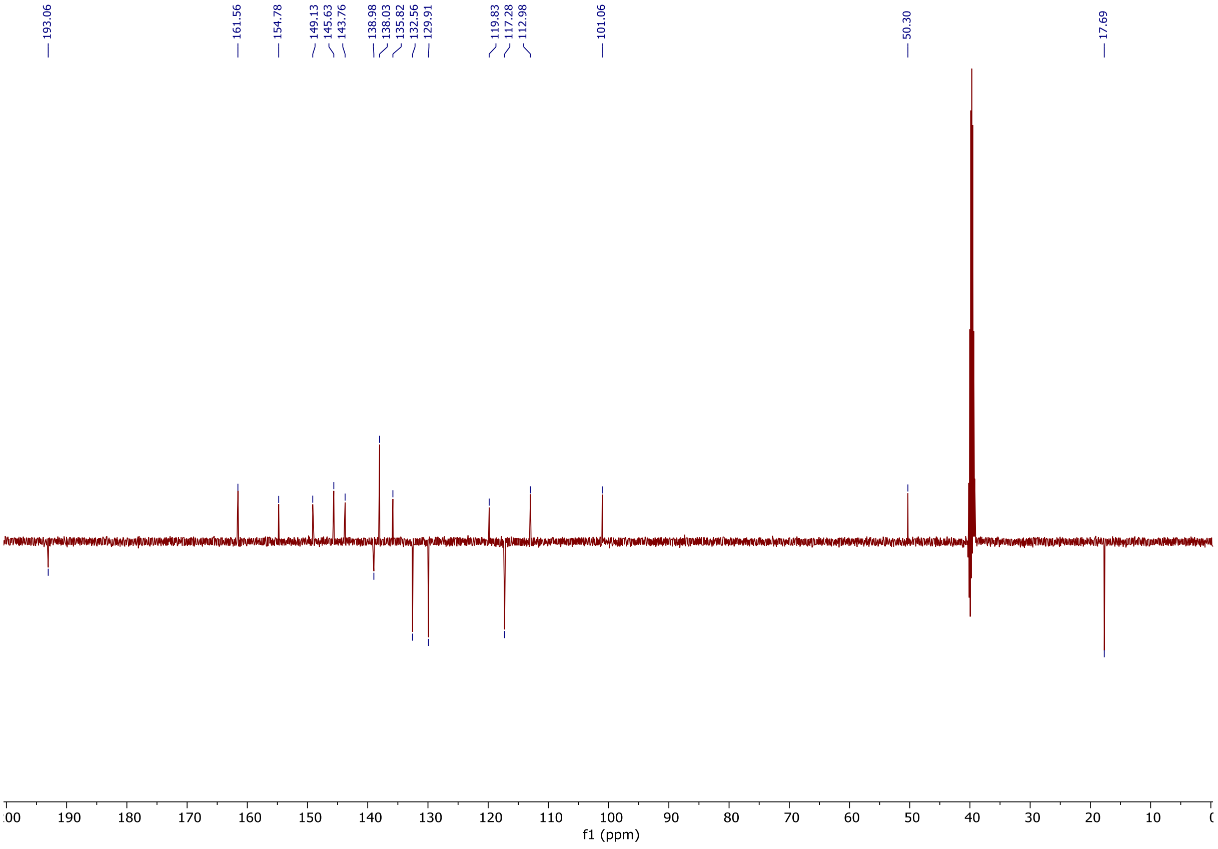

4-{[9-(4-Formyl-2,6-dimethylphenyl)-6-oxo-5*H*,6*H*,7*H*,8*H*,9*H*-pyrimido[4,5-*b*][1,4]diazepin-2-yl]amino}benzonitrile (42):

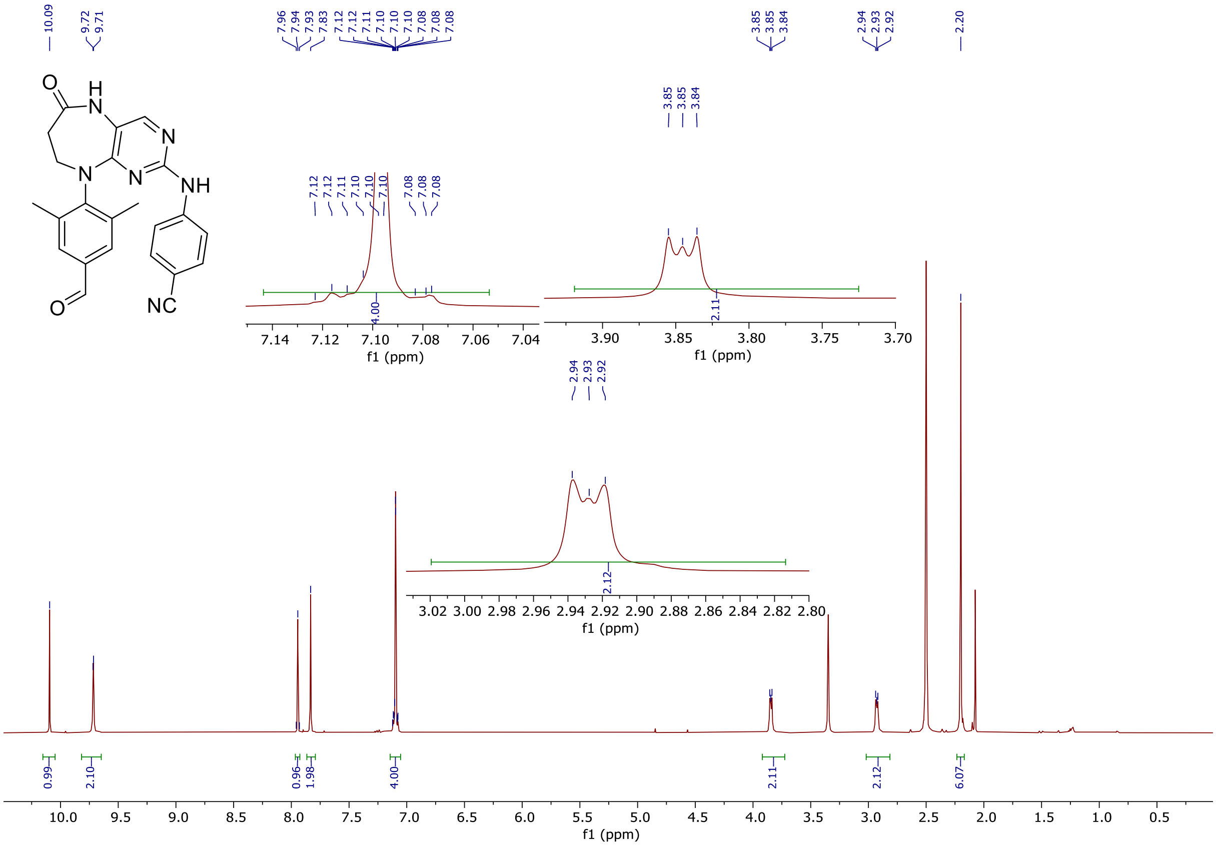

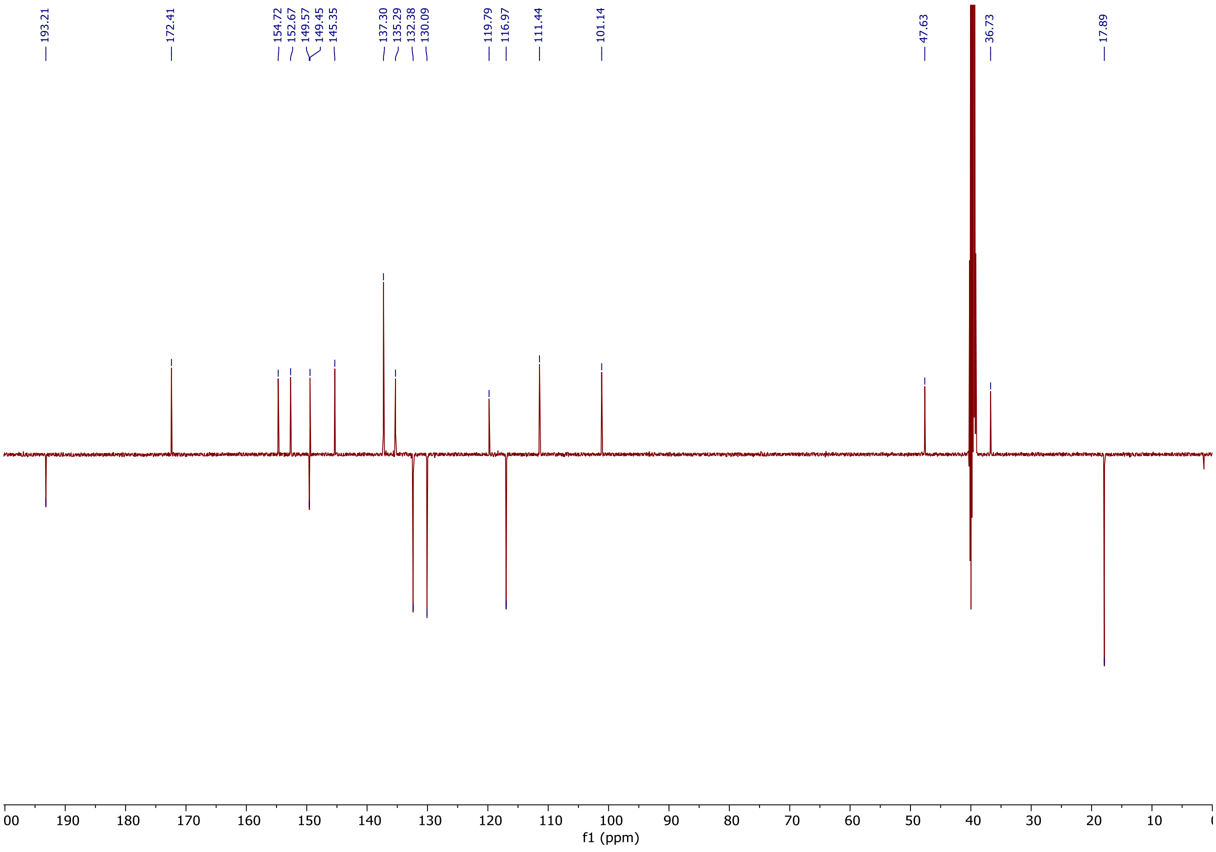

2. Copies of HPLC chromatograms of compounds with *in vitro* data

Blank spectrum

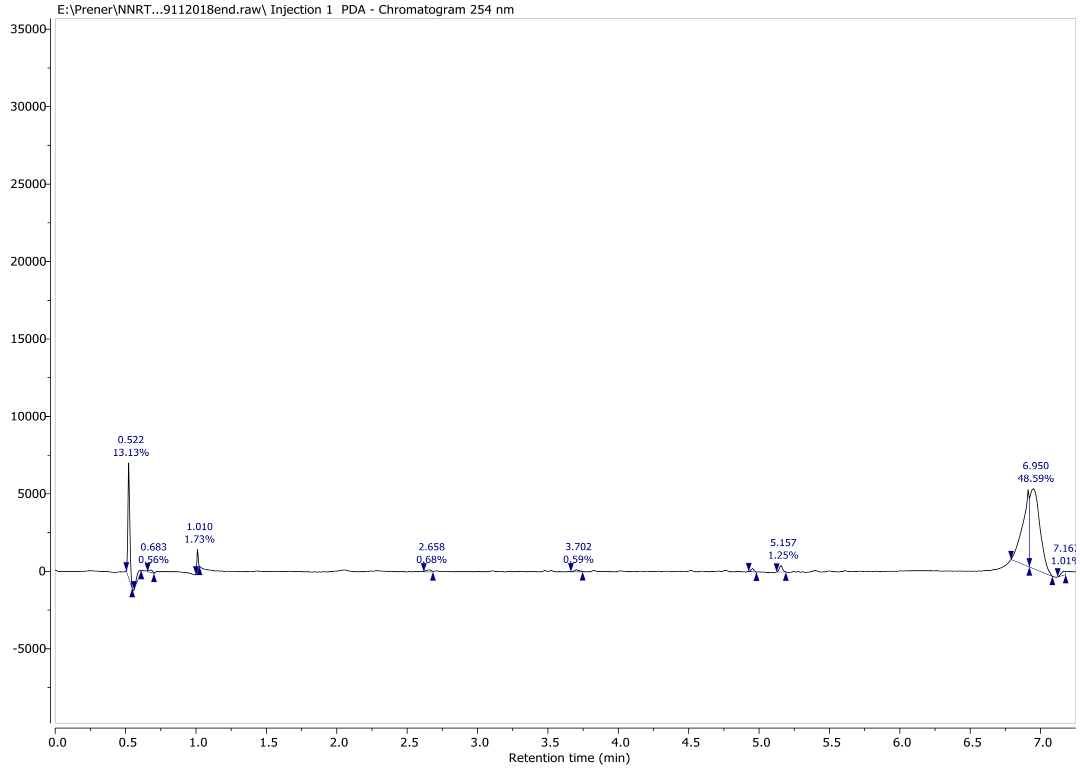

4-{2-[(4-Cyanophenyl)amino]-8-oxo-8,9-dihydro-7H-purin-9-yl}-3,5-dimethylbenzonitrile (1):

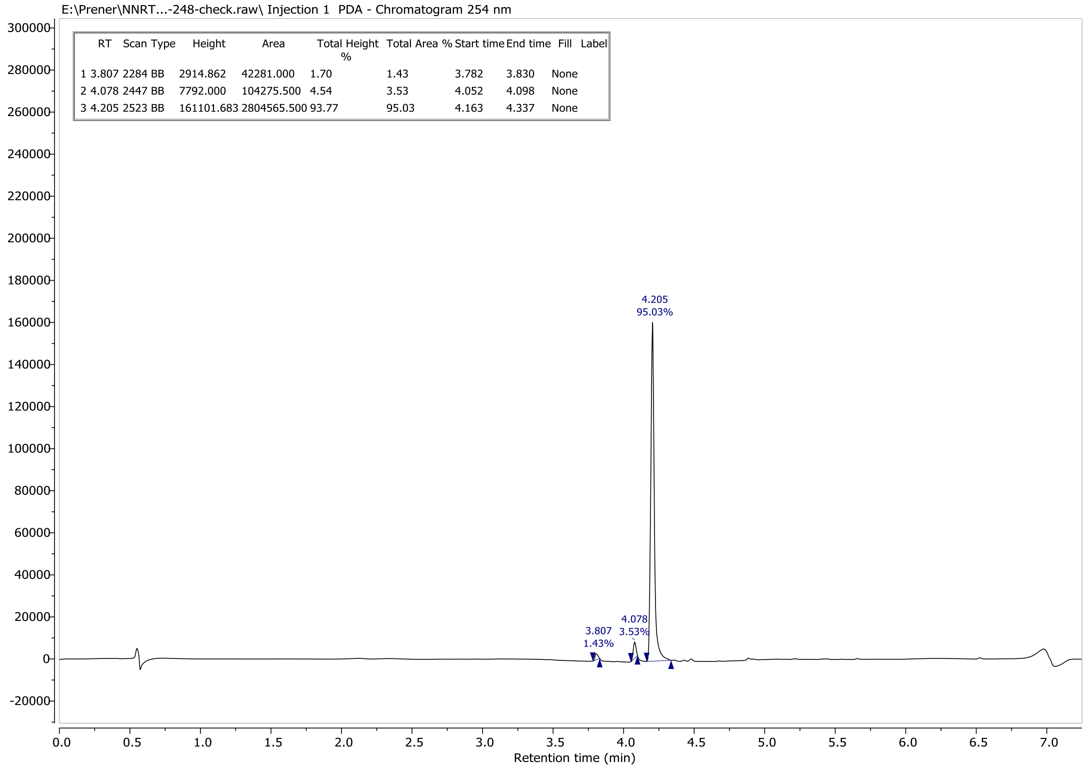

4-[(9-{4-[(1*E*)-2-Cyanoeth-1-en-1-yl]-2,6-dimethylphenyl}-8-oxo-8,9-dihydro-7*H*-purin-2-yl)amino]benzonitrile (2):

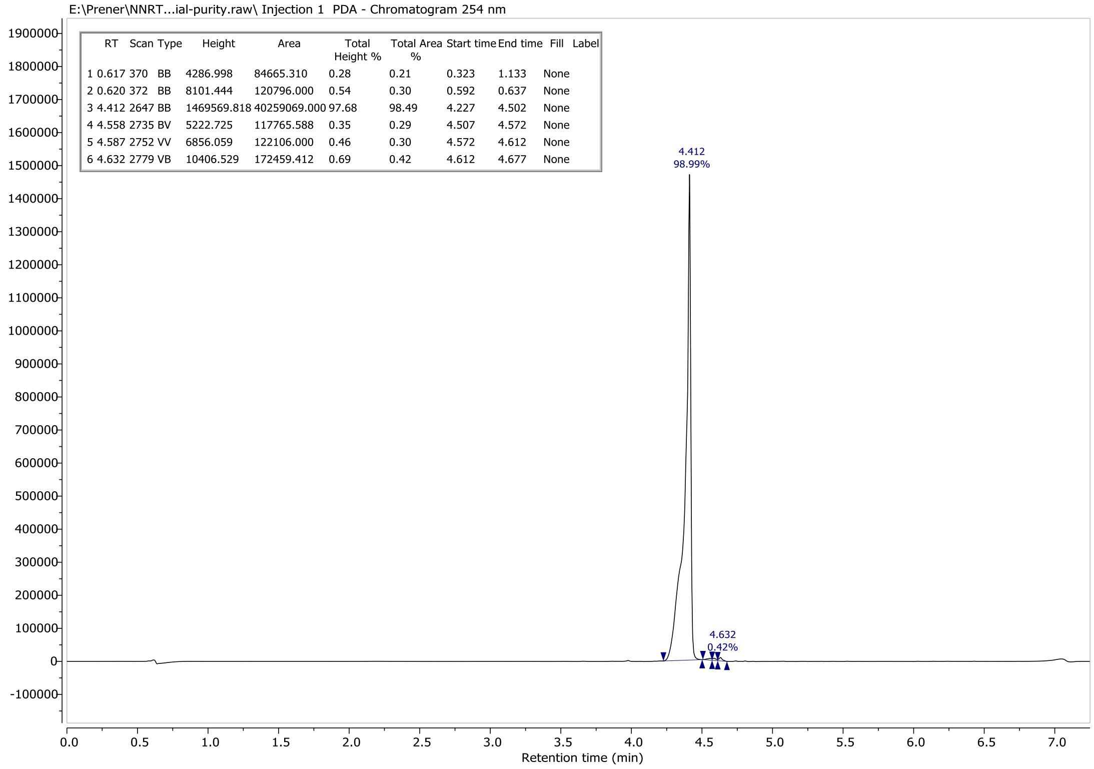

	RT	Scan	Type	Height	Area	Total Height %	Total Area %	Start time	End time	Fill	Label
1	0.617	370	BB	4286.998	84665.310	0.28	0.21	0.323	1.133	None	
2	0.620	372	BB	8101.444	120796.000	0.54	0.30	0.592	0.637	None	
3	4.412	2647	BB	1469569.818	40259069.000	97.68	98.49	4.227	4.502	None	
4	4.558	2735	BV	5222.725	117765.588	0.35	0.29	4.507	4.572	None	
5	4.587	2752	VV	6856.059	122106.000	0.46	0.30	4.572	4.612	None	
6	4.632	2779	VB	10406.529	172459.412	0.69	0.42	4.612	4.677	None	

4-{2-[(4-cyanophenyl)amino]-6-oxo-6,7-dihydropteridin-8(5H)-yl}-3,5-dimethylbenzonitrile (3):

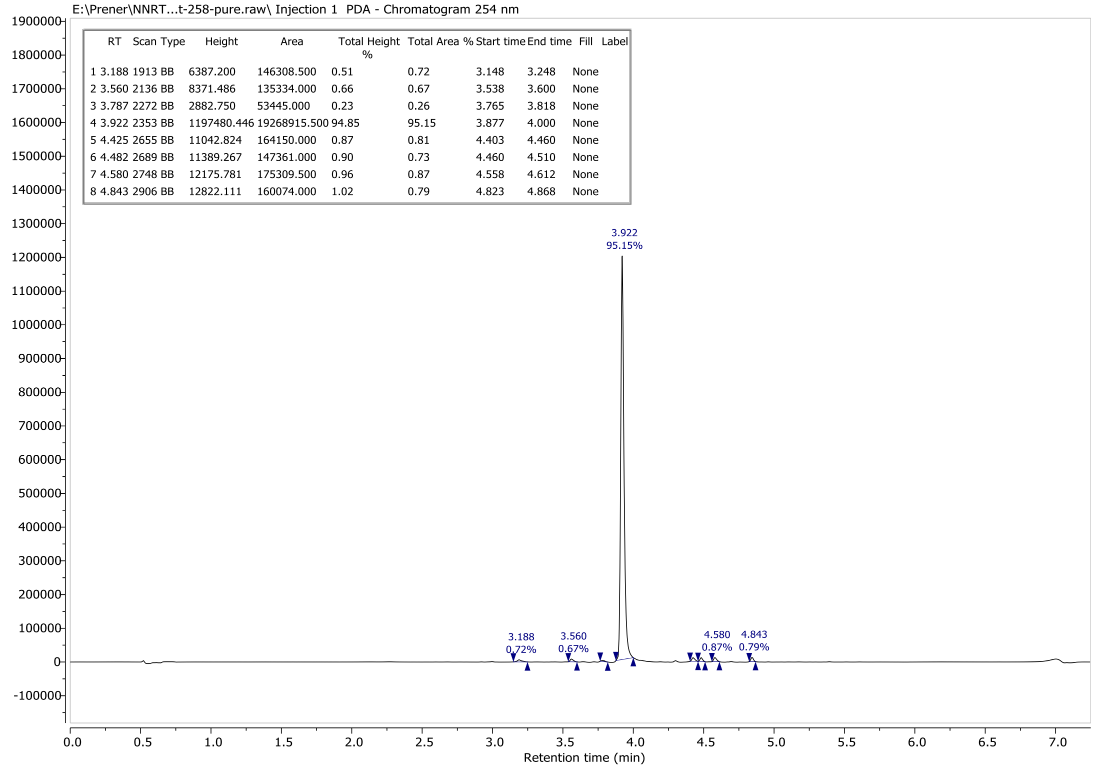

	RT	Scan	Type	Height	Area	Total Height %	Total Area %	Start time	End time	Fill	Label
1	3.188	1913	BB	6387.200	146308.500	0.51	0.72	3.148	3.248	None	
2	3.560	2136	BB	8371.486	135334.000	0.66	0.67	3.538	3.600	None	
3	3.787	2272	BB	2882.750	53445.000	0.23	0.26	3.765	3.818	None	
4	3.922	2353	BB	1197480.446	19268915.500	94.85	95.15	3.877	4.000	None	
5	4.425	2655	BB	11042.824	164150.000	0.87	0.81	4.403	4.460	None	
6	4.482	2689	BB	11389.267	147361.000	0.90	0.73	4.460	4.510	None	
7	4.580	2748	BB	12175.781	175309.500	0.96	0.87	4.558	4.612	None	
8	4.843	2906	BB	12822.111	160074.000	1.02	0.79	4.823	4.868	None	

4-[(8-{4-[(1*E*)-2-Cyanoeth-1-en-1-yl]-2,6-dimethylphenyl}-6-oxo-5,6,7,8-tetrahydropteridin-2-yl)amino]benzonitrile (4):

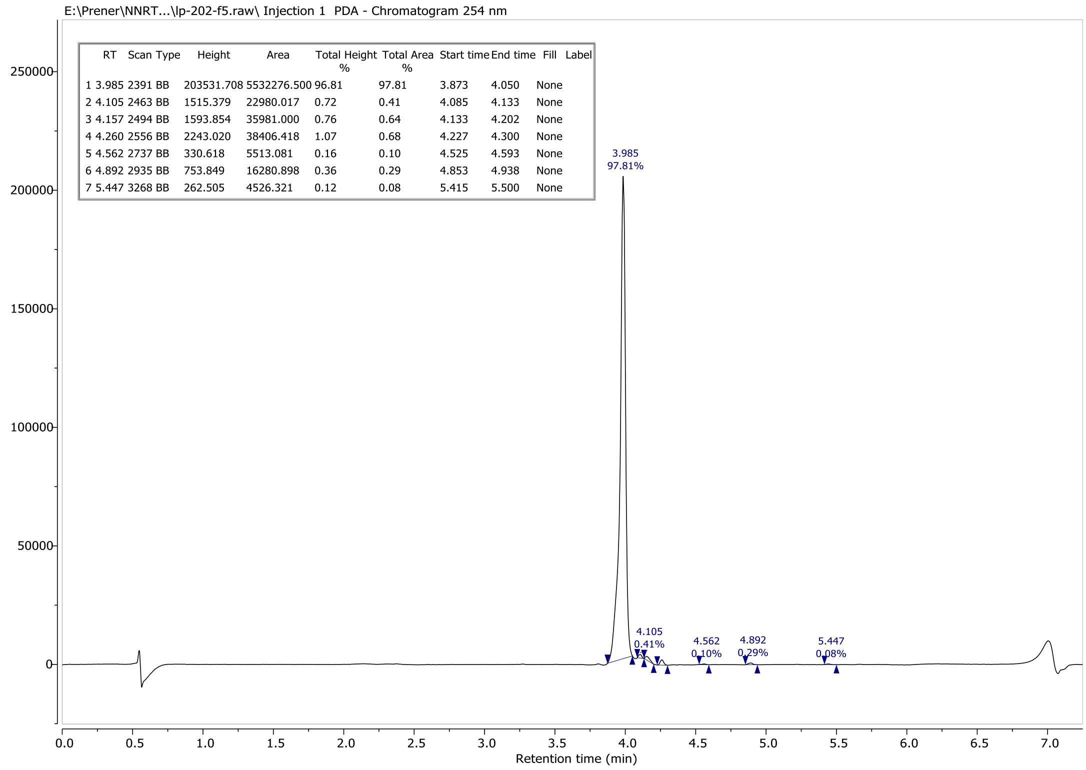

	RT	Scan	Type	Height	Area	Total Height %	Total Area %	Start time	End time	Fill	Label
1	3.985	2391	BB	203531.708	5532276.500	96.81	97.81	3.873	4.050	None	
2	4.105	2463	BB	1515.379	22980.017	0.72	0.41	4.085	4.133	None	
3	4.157	2494	BB	1593.854	35981.000	0.76	0.64	4.133	4.202	None	
4	4.260	2556	BB	2243.020	38406.418	1.07	0.68	4.227	4.300	None	
5	4.562	2737	BB	330.618	5513.081	0.16	0.10	4.525	4.593	None	
6	4.892	2935	BB	753.849	16280.898	0.36	0.29	4.853	4.938	None	
7	5.447	3268	BB	262.505	4526.321	0.12	0.08	5.415	5.500	None	

4-{2-[(4-Cyanophenyl)amino]-6-oxo-5*H*,6*H*,7*H*,8*H*,9*H*-pyrimido[4,5-b][1,4]diazepin-9-yl}-3,5-dimethylbenzonitrile (5):

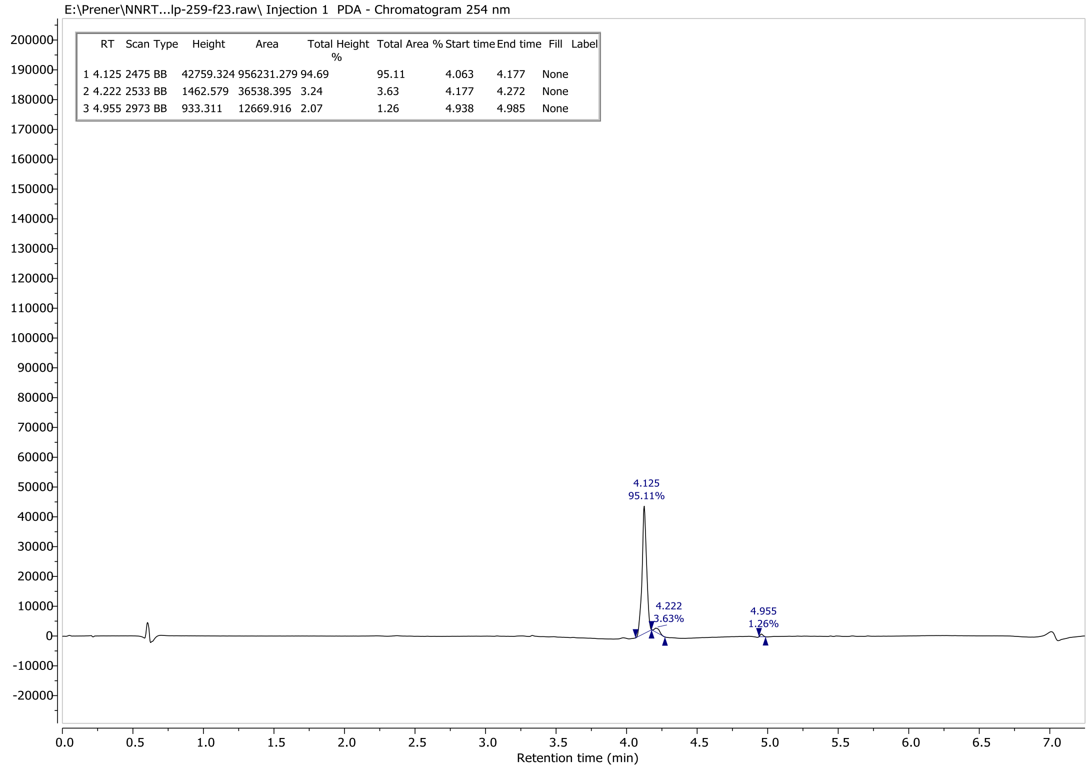

	RT	Scan	Type	Height	Area	Total Height %	Total Area %	Start time	End time	Fill	Label
1	4.125	2475	BB	42759.324	956231.279	94.69	95.11	4.063	4.177	None	
2	4.222	2533	BB	1462.579	36538.395	3.24	3.63	4.177	4.272	None	
3	4.955	2973	BB	933.311	12669.916	2.07	1.26	4.938	4.985	None	

4-[(9-{4-[(1*E*)-2-Cyanoeth-1-en-1-yl]-2,6-dimethylphenyl}-6-oxo-5*H*,6*H*,7*H*,8*H*,9*H*-pyrimido[4,5-*b*][1,4]diazepin-2-yl)amino]benzonitrile (6):

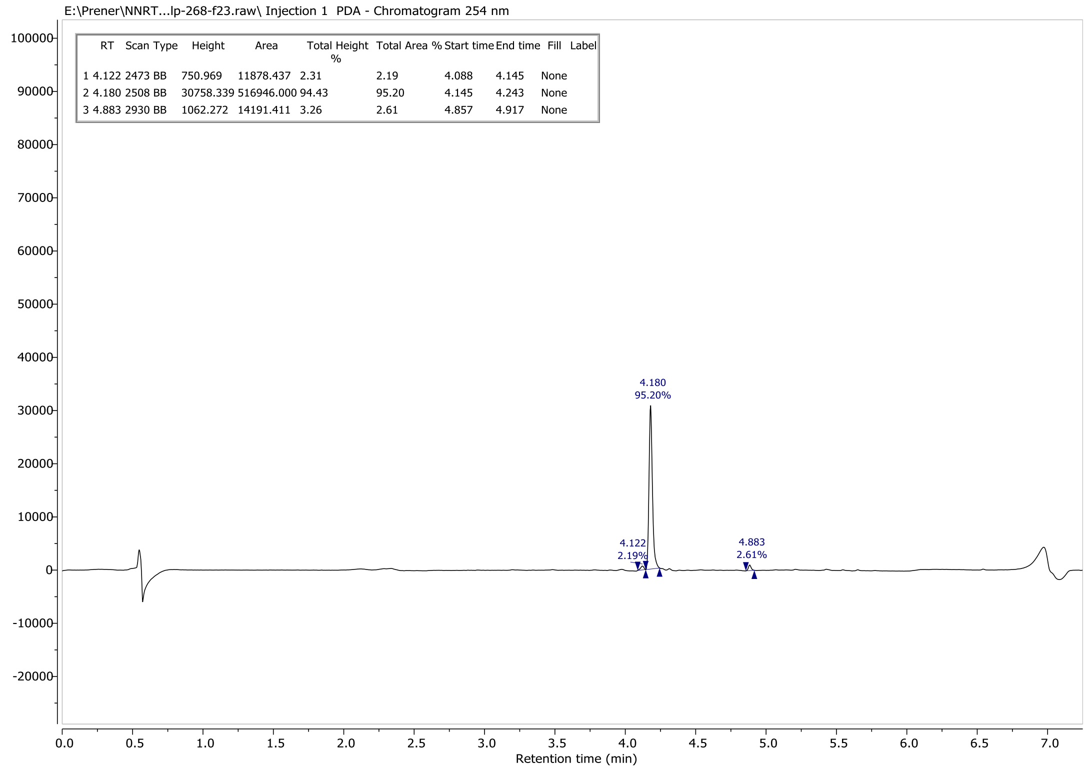

4-({8-[4-(hydroxymethyl)-2,6-dimethylphenyl]-6-oxo-5,6,7,8-tetrahydropteridin-2-yl}amino)benzonitrile (28):

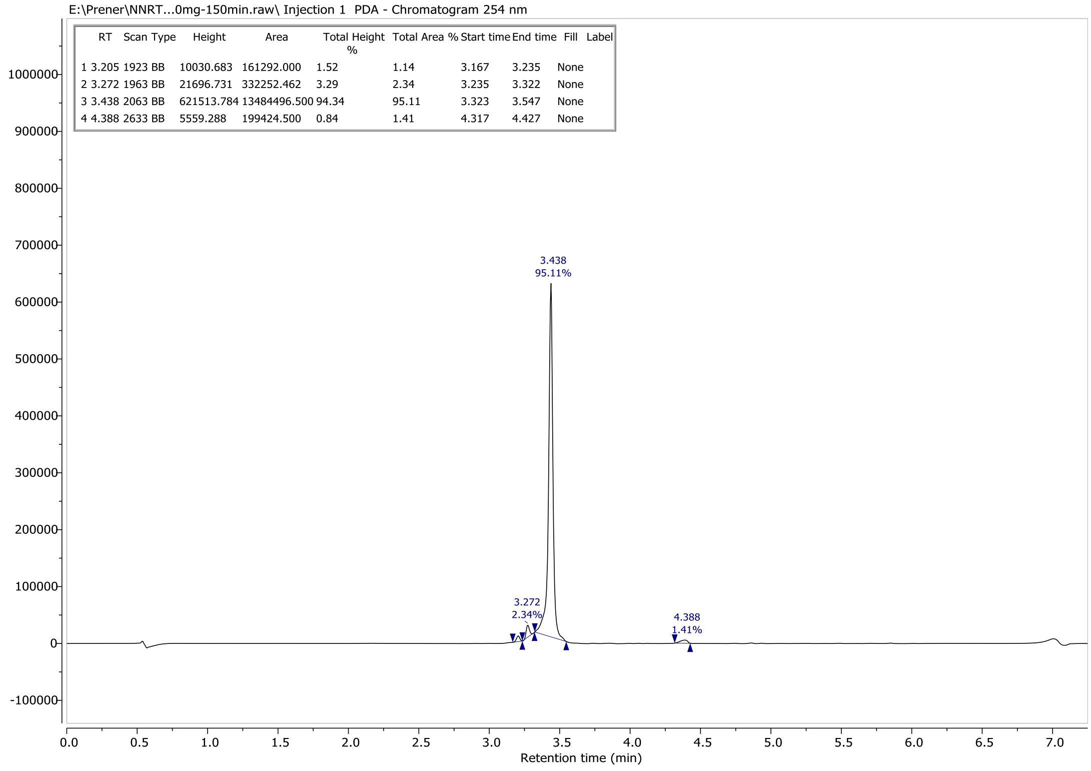

4-({9-[4-(Hydroxymethyl)-2,6-dimethylphenyl]-8-oxo-8,9-dihydro-7*H*-purin-2-yl}amino)benzonitrile (32):

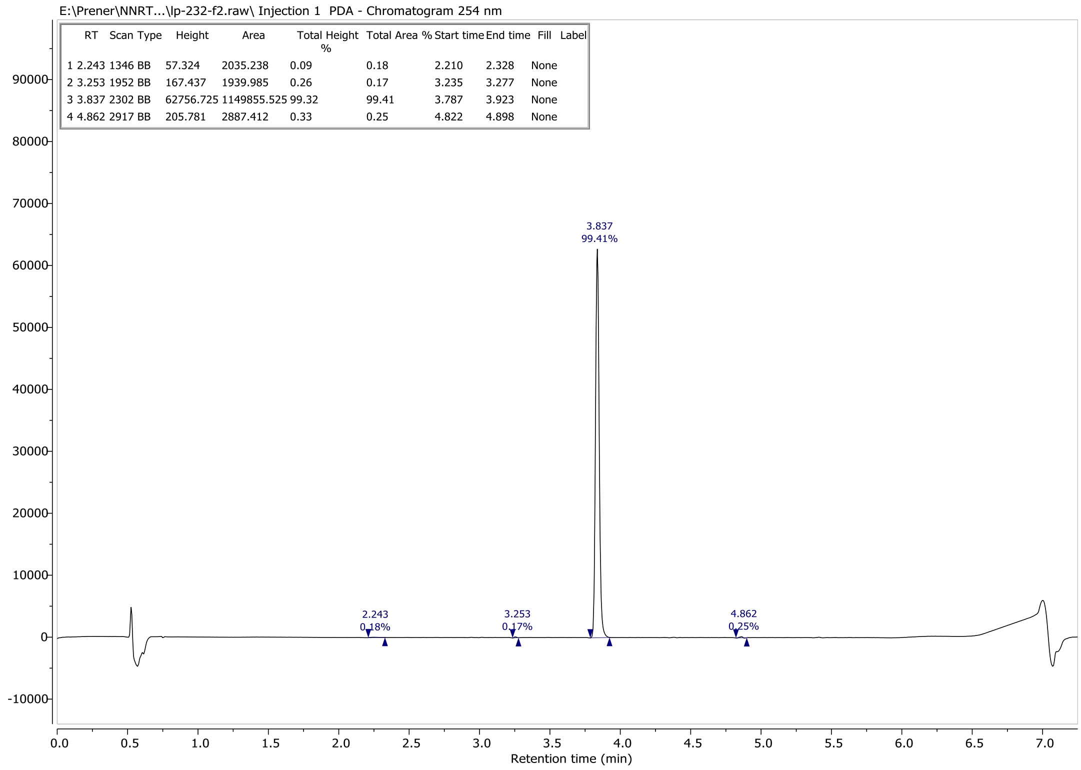

4-({9-[4-(hydroxymethyl)-2,6-dimethylphenyl]-6-oxo-5*H*,6*H*,7*H*,8*H*,9*H*-pyrimido[4,5-*b*][1,4]diazepin-2-yl}amino)benzonitrile (39):

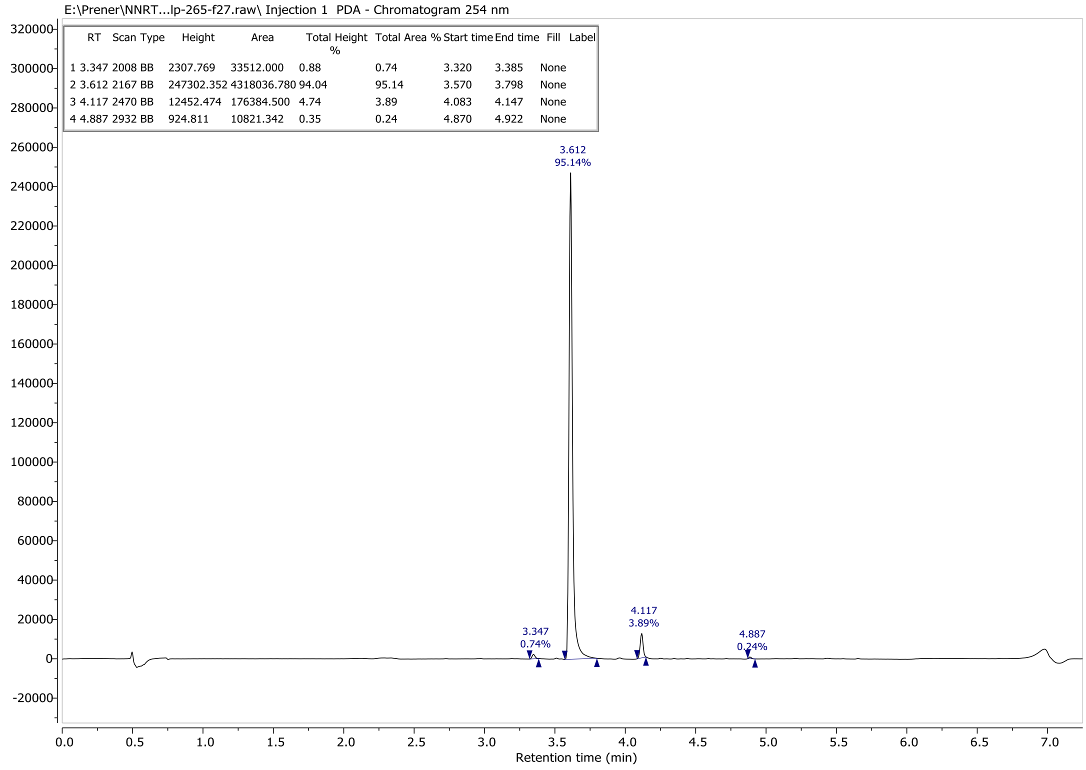

4-{[9-(4-Formyl-2,6-dimethylphenyl)-8-oxo-8,9-dihydro-7*H*-purin-2-yl]amino}benzonitrile (40):

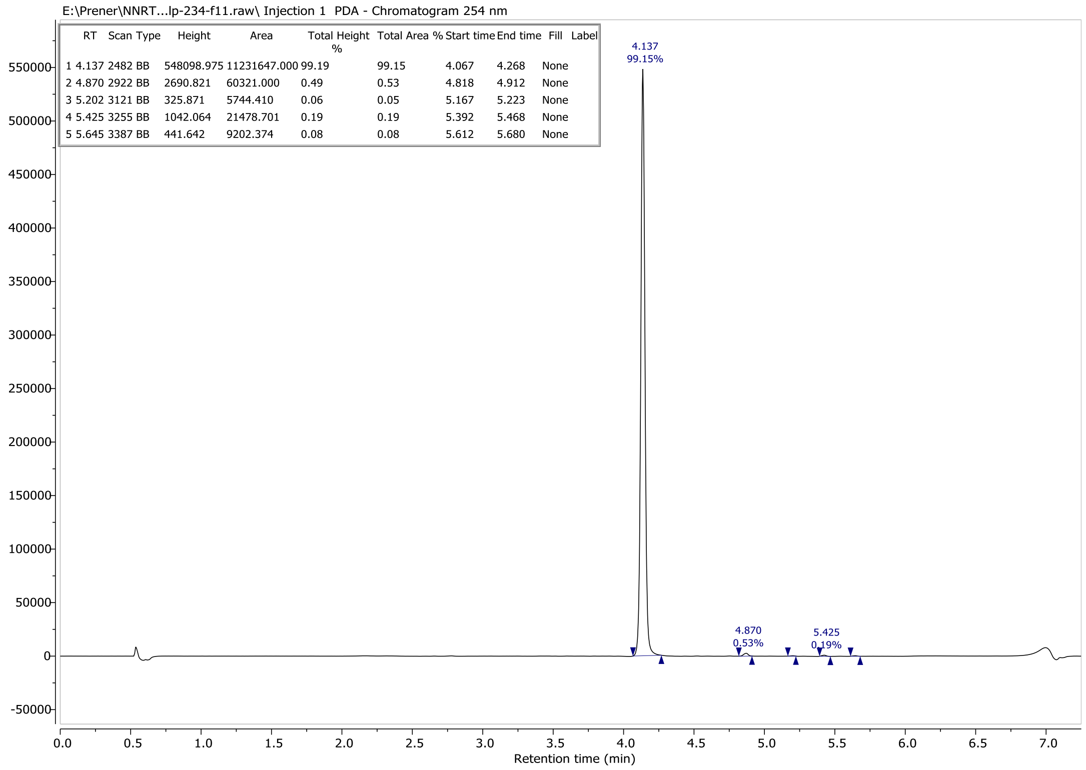

	RT	Scan	Type	Height	Area	Total Height %	Total Area %	Start time	End time	Fill	Label
1	4.137	2482	BB	548098.975	11231647.000	99.19	99.15	4.067	4.268	None	
2	4.870	2922	BB	2690.821	60321.000	0.49	0.53	4.818	4.912	None	
3	5.202	3121	BB	325.871	5744.410	0.06	0.05	5.167	5.223	None	
4	5.425	3255	BB	1042.064	21478.701	0.19	0.19	5.392	5.468	None	
5	5.645	3387	BB	441.642	9202.374	0.08	0.08	5.612	5.680	None	

4-{[8-(4-Formyl-2,6-dimethylphenyl)-6-oxo-5,6,7,8-tetrahydropteridin-2-yl]amino}benzonitrile (41):

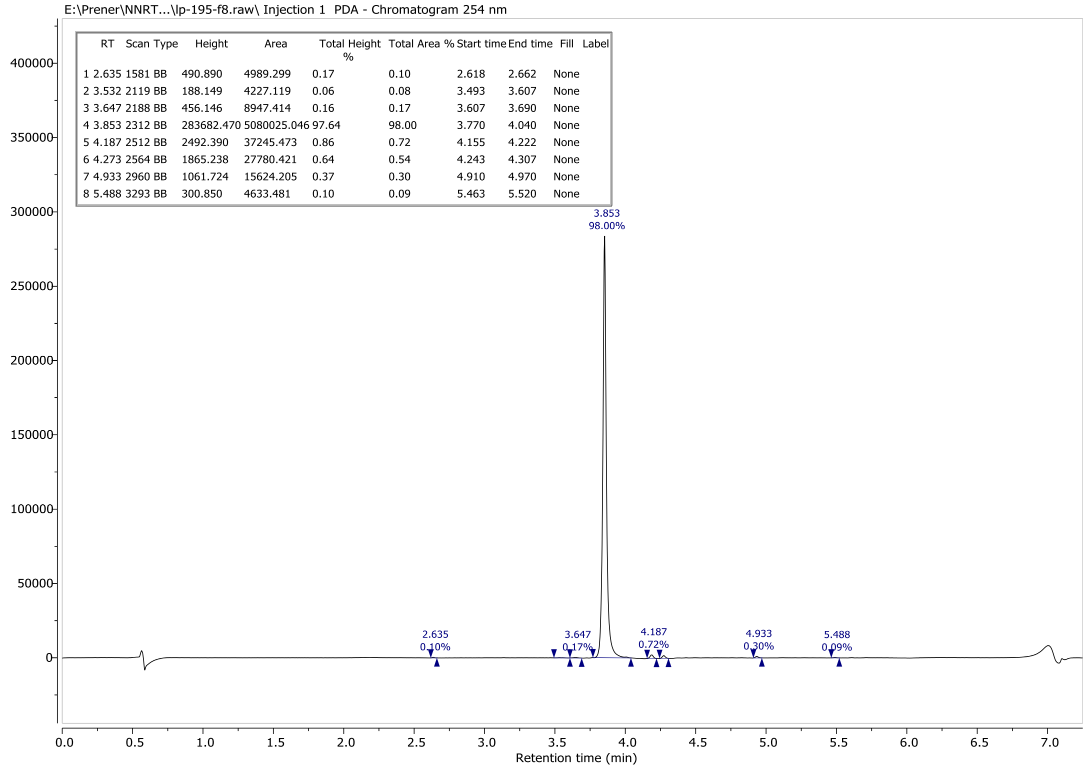

	RT	Scan	Type	Height	Area	Total Height %	Total Area %	Start time	End time	Fill	Label
1	2.635	1581	BB	490.890	4989.299	0.17	0.10	2.618	2.662	None	
2	3.532	2119	BB	188.149	4227.119	0.06	0.08	3.493	3.607	None	
3	3.647	2188	BB	456.146	8947.414	0.16	0.17	3.607	3.690	None	
4	3.853	2312	BB	283682.470	5080025.046	97.64	98.00	3.770	4.040	None	
5	4.187	2512	BB	2492.390	37245.473	0.86	0.72	4.155	4.222	None	
6	4.273	2564	BB	1865.238	27780.421	0.64	0.54	4.243	4.307	None	
7	4.933	2960	BB	1061.724	15624.205	0.37	0.30	4.910	4.970	None	
8	5.488	3293	BB	300.850	4633.481	0.10	0.09	5.463	5.520	None	

4-{[9-(4-Formyl-2,6-dimethylphenyl)-6-oxo-5*H*,6*H*,7*H*,8*H*,9*H*-pyrimido[4,5-*b*][1,4]diazepin-2-yl]amino}benzonitrile (42):

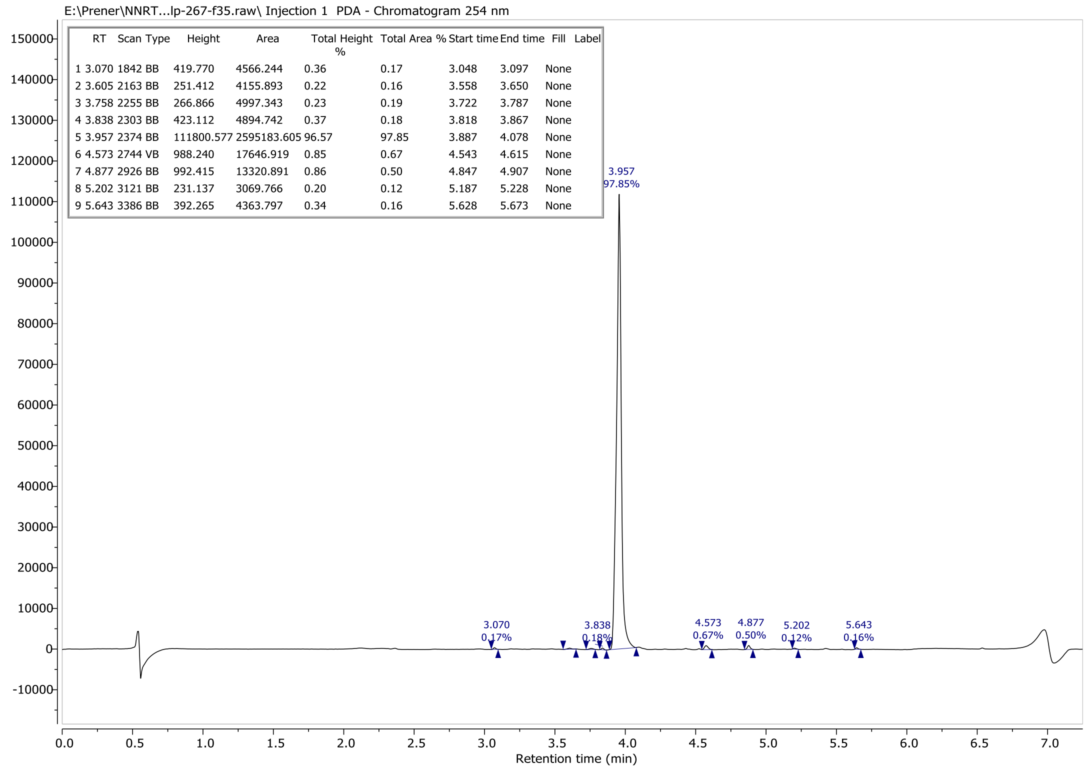

	RT	Scan	Type	Height	Area	Total Height %	Total Area %	Start time	End time	Fill	Label
1	3.070	1842	BB	419.770	4566.244	0.36	0.17	3.048	3.097	None	
2	3.605	2163	BB	251.412	4155.893	0.22	0.16	3.558	3.650	None	
3	3.758	2255	BB	266.866	4997.343	0.23	0.19	3.722	3.787	None	
4	3.838	2303	BB	423.112	4894.742	0.37	0.18	3.818	3.867	None	
5	3.957	2374	BB	111800.577	2595183.605	96.57	97.85	3.887	4.078	None	
6	4.573	2744	VB	988.240	17646.919	0.85	0.67	4.543	4.615	None	
7	4.877	2926	BB	992.415	13320.891	0.86	0.50	4.847	4.907	None	
8	5.202	3121	BB	231.137	3069.766	0.20	0.12	5.187	5.228	None	
9	5.643	3386	BB	392.265	4363.797	0.34	0.16	5.628	5.673	None	

Table S1. Data collection and refinement statistics for X-ray structures.

	Compound **2**	Compound **4**	Compound **6**
PDB code	**8FCC**	**8FCD**	**8FCE**
Wavelength (Å)	1.000	1.000	1.000
Space Group	$C222_1$	$C222_1$	$C222_1$
Unit Cell (a, b, c in Å)	120.1, 154.8, 154.67	119.8, 155.2, 155.0	118.8, 154.6, 153.5
Resolution (Å)	47.44-2.57 (2.66-2.57)	47.50-2.57 (2.66-2.57)	47.3-2.77 (2.89-2.77)
No. of reflections	617,410 (63,944)	612,704 (64,258)	472,745 (61,248)
No. unique	46,163 (4,483)	46,280 (4,511)	36,293 (4,371)
I/σ	13.4 (2.3)	14.5 (2.2)	13.4 (2.3)
CC (1/2)	0.998 (0.748)	0.998 (0.774)	0.998 (0.761)
Completeness (%)	100.0 (100.0)	100.0 (100.0)	100.0 (100.0)
**Refinement Statistics**			
Resolution (Å)	47.44-2.57	47.41-2.57	47.11-2.77
No. reflections (F≥0)	46,110	46,229	36,249
R-factor[a]	0.200	0.188	0.204
R-free[a]	0.257	0.231	0.262
RMS bond lengths (Å)	0.009	0.010	0.010
RMS bond angles (°)	1.07	1.22	1.07

[a] *R*-factor and *R*-free = $\sum||F_{obs}| - |F_{calc}||/\sum|F_{obs}|$ for 95% of recorded data (*R*-factor) or 5% of data (*R*-free).

Table S2. Pharmacological parameters for an analyte compound metabolism were calculated using the following equations:

**Parameter**	**Equation**
Half Life	$$T_{1/2} = \frac{Ln(2)}{-1 * Slope}$$
Intrinsic Clearance (*in vitro*)	$$Cl_{int,\, in\, vitro} = \frac{\ln 2}{T_{1/2} * Conc}$$ *Conc* = 1.0 mg protein/mL
Intrinsic Clearance	$$Cl_{int} = \frac{Cl_{int,\, in\, vitro} * Liver\ Mass * Yield}{Body\ Weight}$$ *Liver Mass* = 1,800 gr *Yield* = 45 mg/g liver *Body Weight* = 70 kg
Predicted Hepatic Clearance	$$Cl_{int,pred} = \frac{Cl_{int} * Q_H}{Cl_{int} + Q_H}$$ Q_H *(Hepatic Blood Flow)* = 1.3 L/hr/kg
